# Alterations in microRNA expression associated with alcohol consumption in rectal cancer subjects

**DOI:** 10.1007/s10552-017-0882-2

**Published:** 2017-03-16

**Authors:** Lila E. Mullany, Jennifer S. Herrick, Roger K. Wolff, John R. Stevens, Martha L. Slattery

**Affiliations:** 1 Department of Internal Medicine, University of Utah, 383 Colorow Bldg., Salt Lake City, UT 84108 USA; 2 Department of Mathematics and Statistics, Utah State University, 3900 Old Main Hill, Logan, UT 84322 USA

**Keywords:** MiRNA, Rectal, Colorectal, Cancer, Alcohol

## Abstract

**Purpose:**

Alcohol consumption has been purported to influence many diseases. MicroRNAs (miRNAs) may be influenced by compounds found in alcohol. In this investigation, we test the hypothesis that total alcohol, beer, wine, and hard liquor influence miRNA expression.

**Methods:**

We studied 1447 colorectal (CR) cancer cases with normal CR mucosa and carcinoma miRNA expression data along with alcohol consumption data. We analyzed long-term and long-term and current (LTC) alcohol use for beer, liquor, and wine with miRNA expression between paired carcinoma and normal colon and rectal tissues, adjusting for multiple comparisons using the positive false discovery rate *q*-value. MiRNAs associated significantly with alcohol were examined with all-cause mortality (ACM). MiRNAs associated significantly with ACM were examined with RNA-Seq data.

**Results:**

Expression of 84 miRNAs was associated significantly with LTC wine use in normal rectal mucosa. Higher expression of two of these miRNAs significantly worsened ACM: hsa-miR-210 (Hazard Ratio [HR] 1.12, 95% CI (1.03, 1.21), *p*-value = 0.004), and hsa-miR-92a-1-5p (HR 1.20, 95% CI (1.04, 1.38), *p*-value = 0.013). These miRNAs were downregulated across levels of LTC wine consumption.

**Conclusions:**

Our results suggest that wine influences miRNA expression in rectal cancer, supporting the hypothesis that components in alcohol influence miRNA expression.

**Electronic supplementary material:**

The online version of this article (doi:10.1007/s10552-017-0882-2) contains supplementary material, which is available to authorized users.

## Background

Alcohol intake has been associated with all-cause mortality (ACM) as well as with several diseases, including certain types of cancer, cardiovascular disease, diabetes mellitus, liver diseases, and obesity [1–5]. Furthermore, specific types of alcohol have been shown to influence disease risk uniquely [6, 7]. Some studies have shown an increase in ACM at both high and low levels of intake and others have shown that moderate alcohol consumption does not increase disease risk [3, 8]. Given alcoholic beverage-specific associations and the j-shaped curve associated with ACM, multiple biological mechanisms may operate to influence risk of disease and death from alcohol consumption.

Alcohol consumption has been shown to incite inflammatory responses, and prolonged consumption can induce systemic inflammation [5]. Lipopolysaccharides (LPS) can become circulated systemically in individuals who consume large amounts of alcohol, as alcohol increases gut membrane permeability [5, 9]. When these antigens reach the liver, pro-inflammatory cytokines such as TNF-α, IL-1, and IL-6 are released, creating a potential systemic inflammatory reaction [10]; LPS has been linked to liver metastasis in colorectal cancer (CRC) as a result of this increased inflammation [11]. Chronic inflammation is known to be a salient process contributing to the development and progression of cancer [12], and alcohol consumption has been shown to increase risk of CRC compared to non-drinkers [13]; inflammation due to alcohol consumption may therefore contribute to the pathogenesis of some diseases, such as CRC. Additionally, alcohol has been assessed as a component of DNA methylation pathways and it has been hypothesized that alcohol influences cancer risk by influencing these pathways [14, 15].

In addition to these broad mechanisms associated with alcohol intake, types of alcoholic beverages contain specific components that may influence disease risk. For instance, purines, found most abundantly in beer and in liquor to a lesser extent, may have pro-inflammatory effects through increased serum uric acid production [16, 17]. Polyphenols in wine, such as resveratrol, may have anti-inflammatory properties that reduce risk of various diseases [18, 19]. Polyphenols are known to have multi-target effects and influence many pathways, including anti-inflammatory responses, anti-proliferation, and anti-oxidation [20].

MiRNAs are non-coding RNA molecules, approximately 22 nucleotides long [21], which are endogenously expressed and serve as mRNA repressors, either through mRNA degradation or translation inhibition [22, 23]. MiRNAs have been shown to target thousands of diverse mRNAs [24], and as such they have been implicated as regulators of a multitude of biological responses including apoptosis, cell differentiation, proliferation, and innate and acquired immunity [25]. Additionally, miRNA dysregulation has been associated with cancer and autoimmune diseases, disease states influenced by chronic inflammation [26]. MiRNAs are purported to be regulated themselves by certain exogenous dietary factors, such as vitamins, fatty acids, and polyphenols, which are present in many foods, including wine [20, 27].

In this study, we analyzed total alcohol consumption as well as intake of specific types of alcohol including beer, liquor and wine, with miRNA expression. We assessed associations between alcoholic beverages and miRNA expression in normal colorectal mucosa (NCRM) and with differential miRNA expression between paired carcinoma and normal mucosa, analyzing colon and rectal cancers separately. We hypothesize that alcohol consumption will be associated with altered miRNA expression and that type of alcoholic beverage, i.e., wine, beer, and liquor, may have unique associations with normal and/or differential miRNA expression due to different mechanisms attributable to different components of alcohol.

## Methods

### Study population

Data come from participants in the population-based Diet, Activity, and Lifestyle study that were recruited from Utah or the Kaiser Permanente Medical Care Program of Northern California (KPMCP). Colon cancer cases were identified as having a primary adenocarcinoma diagnosed between 1 October 1991 and 30 September 1994, while rectal cancer cases were diagnosed between May 1997 and May 2001. Eligible cases were between 30 and 79 years of age at diagnosis, currently living in the study area, spoke English and were able to complete an interview, and had no prior history of CRC, Crohn’s disease, ulcerative colitis, or known familial adenomatous polyposis. More detailed descriptions of proportion of included and excluded cases for colon cancer [28] and rectal cancer [29] may be found in our previous works. This study was approved by the Institutional Review Board at the University of Utah; all participants signed an informed consent form.

### Alcohol data

Data were collected by trained and certified interviewers using laptop computers; all interviews were audio-taped as previously described and reviewed for quality control purposes [30]. The study referent period was 2 years prior to diagnosis for cases. Information was collected on type, amount, and duration of alcohol consumption, which was defined in terms of liquor, including whiskey, rum, gin, vodka, tequila, liqueurs, etc., beer, including malt liquor and non-alcoholic beers, and wine, including champagne, sherry, and wine cooler beverages. Alcohol consumed was measured in number of drinks consumed, as measured by 12-ounces (oz.) for beer, 4 oz. for wine, and 1.5 oz. for liquor, per week or month and respondents must have consumed on average at least one beverage a month to be considered a consumer. Diet history on what cases typically consumed was collected, including alcohol consumption for weekdays and weekend days as well as type, amount, and frequency of alcohol consumed. Data were collected for the referent year, and 10 and 20 years prior. Long-term (LT) alcohol consumption was defined as intake during 10 and 20 years prior to diagnosis, while incorporating current alcohol intake during the referent period with that of 10 and 20 years ago was defined as long-term current (LTC).

### MiRNA processing

A pathologist determined carcinoma and normal mucosa sections on slides from formalin-fixed paraffin embedded tissues stained with hematoxylin and eosin; subsequent cuts from the tissue block stained in aniline blue were then appropriately sectioned according to this delineation. RNA was extracted and processed as previously described [31]. 100 ng total RNA was labeled with Cy3 and hybridized to Agilent Human miRNA Microarrays V19.0 and were scanned on an Agilent SureScan microarray scanner model G2600D using Agilent Feature Extract software v.11.5.1.1. Data were required to pass stringent QC parameters established by Agilent that included tests for excessive background fluorescence, excessive variation among probe sequence replicates on the array, and measures of the total gene signal on the array to assess low signal. Samples that failed to meet QC standards were repeated; samples failing QC assessment a second time were deemed to be of poor quality and were excluded from down-stream analysis. Thirteen samples, eight carcinoma, and five, matched normal, which were run over the course of the study were repeated to test for potential changes and bias in the microarray platform. The Agilent platform was found to be highly reliable (*r* = 0.98) in measuring miRNA expression in repeated samples and to have reasonable agreement with NanoString [32] as well as excellent agreement with qRT-PCR [33]. For unpaired samples due to missing normal scans, we imputed values whenever possible for normal mucosa as previously described [34]; this method has yielded results with high accuracy. In order to minimize differences that could be attributed to the array, amount of RNA, location on array, or other factors that could erroneously influence expression, total gene signal was normalized by multiplying each sample by a scaling factor which was the median of the 75th percentiles of all the samples divided by the 75th percentile of each individual sample [35]. This scaling factor was implemented using SAS 9.4.

### RNA-seq data

Normal CR RNA-Seq was available for 183 subjects for which alcohol data were available. These samples were taken from the study subjects used for miRNA analysis and were extracted, isolated, and purified as previously described [31]. RNA library construction was done with the Illumina TruSeq Stranded Total RNA Sample Preparation Kit with Ribo-Zero. Agencount AMPure XP beads were used to purify amplified cDNA. A detailed description of the methods can be found in our previous work [36]. Illumina TruSeq v3 single-read flow cell and a 50 cycle single-read sequence run was performed on an Illumina HiSeq instrument. Reads were aligned to a sequence database containing the human genome (build GRCh37/hg19, February 2009 from genome.ucsc.edu) and alignment was performed using novoalign v2.08.01. Total gene counts were calculated using coordinates obtained from http://genome.ucsc.edu. We dropped genes that were not expressed in our data or for which the expression was missing for the majority of samples [36].

### Statistical analysis

Our sample consisted of 2,943 miRNA tissue samples: 1,496 carcinoma tissue, and 1,447 normal CR mucosa. Including the imputed data, there were 1,446 subjects with both carcinoma and paired normal CR mucosa miRNA samples available for analyzing differential expression between carcinoma and normal CR mucosa, and 1,447 subjects available for analyzing normal CR mucosa miRNA expression. We included only the miRNAs in which at least 20% of the samples had some level of detectable expression in the tissue(s) of interest. We stratified the data by colon vs. rectal cancer, and the number of included miRNAs varied from 766 to 817. We examined eight alcohol variables: LT total alcohol use, LTC total alcohol use, LT beer use, LTC beer use, LT wine use, LTC wine use, LT liquor use, and LTC liquor use. These variables were used to determine if there was an association between total alcohol and specific type of alcohol and miRNA expression in both normal colonic or rectal mucosa and for the difference in expression between carcinoma tissue and normal colonic or rectal mucosa. We fit a least-squares linear regression model to the log base two transformed miRNA expression levels and adjusting for age at diagnosis, study center, sex, and current cigarette smoking (defined as smoking during the referent period). *p*-values were generated using the bootstrap method by creating a distribution of 10,000 F statistics derived by resampling the residuals from the null hypothesis model of no association between the alcohol variables and the miRNAs [37], using the boot package in R. Associations were considered significant if the positive false discovery rate, also known as a q-value, was less than 0.1 [38]. We transformed the miRNAs to standard normal to calculate standardized the regression slopes in order to compare the results across the miRNA and lifestyle factors and present the trend of the miRNA expression across the levels of each alcohol variable. Alcohol consumption levels were classified as ‘none’ if subjects consumed <1 drink per month on the long-term alcohol intake questionnaire and no alcoholic beverages reported on the food history questionnaire, ‘moderate’ for males who consumed >0–<20 mg/day and females who consumed >0–<10 mg/day, and ‘high’ for males consuming ≥ 20 g/day and females consuming ≥ 10 g/day.

We analyzed expression levels of miRNAs that were significantly associated with alcohol variables with ACM. Using a Cox-Proportional Hazard Model in the R package survival, we calculated *p*-values based upon 10,000 permutations of the likelihood ratio test adjusting for age, sex, center, and AJCC stage. Survival time was calculated as the time between diagnosis, and death or lost to follow-up. We report the interquartile range of the miRNA expression and hazard ratios (HR) and 95% confidence intervals (CI).

We tested associations between miRNAs found to be associated with alcohol consumption and ACM with mRNA expression in NCRM, using the bootstrap method described above.

### Bioinformatics

QIAGEN Ingenuity Pathway Analysis (IPA http://www.qiagen.com/ingenuity) was utilized to perform pathway analysis on mRNAs associated significantly with hsa-miR-210 in NCRM. We performed a core analysis, including direct and indirect relationships, using the ingenuity knowledge base. We restricted to experimentally verified findings and mammalian species, and considered all data sources, tissues, and mutations.

## Results

The study population consisted of 892 colon cancer cases and 555 rectal cancer cases; 54% of cases were male and 46% were female for colon cases, and 57% of cases were male and 43% were female for rectal cases (Table 1). The average age at diagnosis was 64.7 years for colon cancer subjects and 61.8 years for rectal cancer subjects. Eighty-six percent of colon cases and 89 percent of rectal cases did not have a family history of CRC, while approximately 14 percent and 11 percent, respectively, did.

**Table 1:** Study sample descriptions

	Colon		Rectal	
	N/Mean	(%/SD)	N/Mean	(%/SD)
Age at diagnosis	64.7	(9.5)	61.8	(10.8)
Sex				
Male	485	(54.4)	316	(56.9)
Female	407	(45.6)	239	(43.1)
Center				
Kaiser	626	(70.2)	340	(61.3)
Utah	266	(29.8)	215	(38.7)
Family history of colorectal cancer				
No	770	(86.3)	493	(89.0)
Yes	122	(13.7)	61	(11.0)
Long-term alcohol use				
None	390	(43.7)	239	(43.2)
Moderate	288	(32.3)	168	(30.4)
High	214	(24.0)	146	(26.4)
Long-term & current alcohol use				
None	347	(38.9)	213	(38.5)
Moderate	366	(41.0)	200	(36.2)
High	179	(20.1)	140	(25.3)
Long-term beer use				
None	565	(63.3)	329	(59.7)
Moderate	171	(19.2)	92	(16.7)
High	156	(17.5)	130	(23.6)
Long-term & current beer use				
None	538	(60.3)	311	(56.3)
Moderate	222	(24.9)	111	(20.1)
High	132	(14.8)	130	(23.6)
Long-term wine use				
None	588	(65.9)	365	(66.1)
Moderate	136	(15.2)	97	(17.6)
High	168	(18.8)	90	(16.3)
Long-term & current wine use				
None	519	(58.2)	340	(61.6)
Moderate	236	(26.5)	108	(19.6)
High	137	(15.4)	104	(18.8)
Long-term liquor use				
None	513	(57.5)	339	(61.6)
Moderate	218	(24.4)	133	(24.2)
High	161	(18.0)	78	(14.2)
Long-term & current liquor use				
None	490	(54.9)	330	(59.8)
Moderate	244	(27.4)	141	(25.5)
High	158	(17.7)	81	(14.7)

**Fig. 1:**
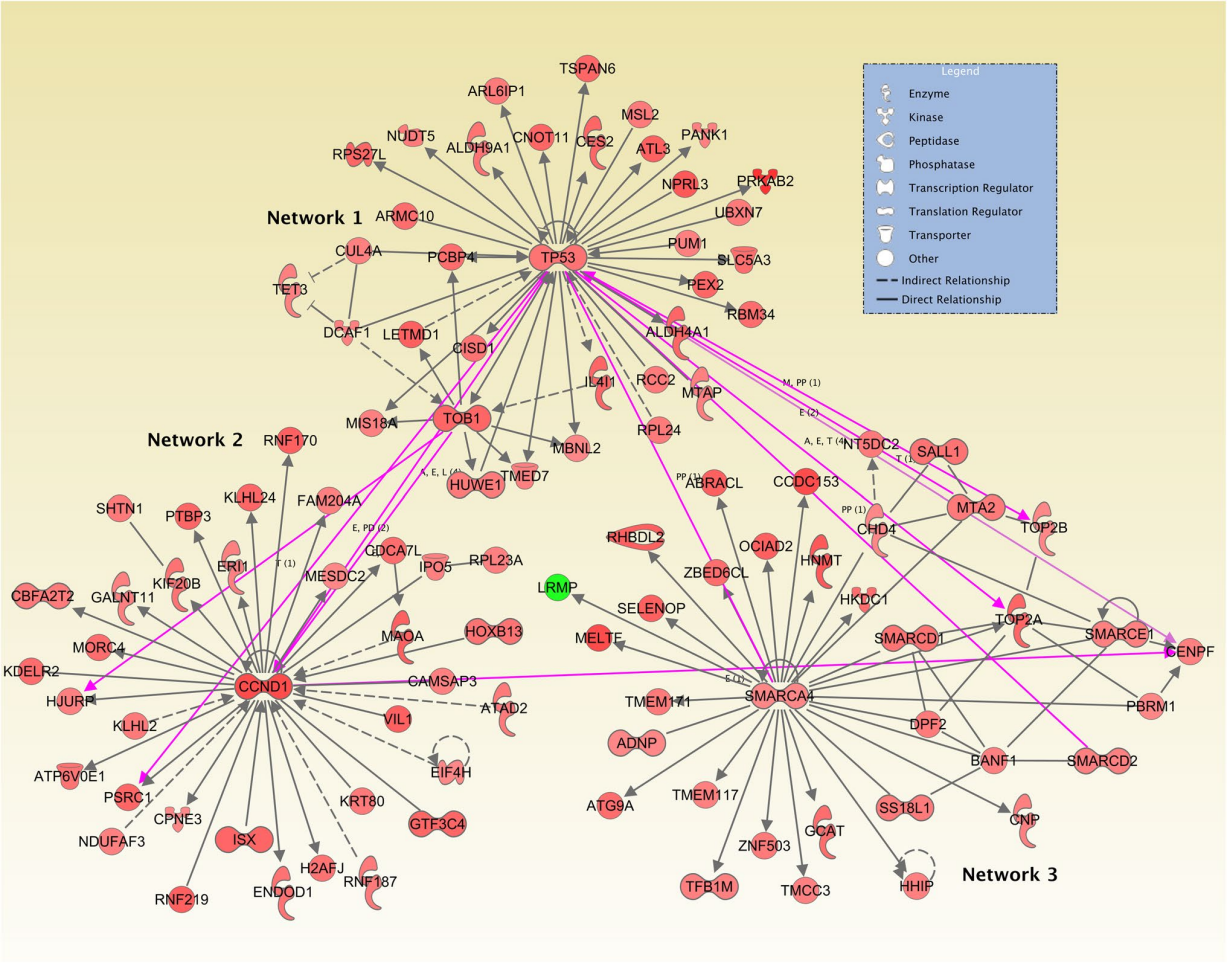
IPA top networks generated for mRNAs associated with hsa-miR-210 with a FC of greater than or equal to 1.5 or less than or equal to 0.67

Eighty-four miRNAs were associated with LTC wine consumption in normal mucosa in rectal cancer subjects (Table 2) after adjusting for cigarette smoking, age, sex, and study center. Five of these miRNAs were upregulated and 79 were downregulated across levels of LTC wine consumption. We analyzed these 84 miRNAs that were different by level of LTC wine consumption with ACM. Higher expression of two of these miRNAs in NCRM was associated significantly with ACM: hsa-miR-210 (HR 1.12, 95% CI (1.03, 1.21), *p*-value = 0.004), and hsa-miR-92a-1-5p (HR 1.20, 95% CI (1.04, 1.38), *p*-value = 0.013) (data shown in Supplemental Table 1). We tested associations between these two miRNAs and mRNAs expressed in NCRM. No mRNAs were associated significantly with hsa-miR-92a-1-5p; 5597 mRNAs associated with hsa-miR-210 when a q-value of <0.05 was applied. Of these, 3169 had a fold change between carcinoma and NCRM of ≥ 1.50 or ≤ 0.67, and were used as input to IPA. The top networks are shown in Fig. 1. The majority of these mRNAs were directly associated with hsa-miR-210. Network 1, centered on *TP53*, has DNA Replication, Recombination, and Repair, Developmental Disorder, and Endocrine Systems Disorder as its top diseases and networks. Network 2, centered on *SMARCA4*, regulates Cellular Development, Hereditary Disorder, and Lipid Metabolism. Network 3, centered on *CCND1*, regulates Cell Cycle, Cellular Assembly and Organization, and DNA Replication, Recombination and Repair. At the center of networks 1 and 3 are *TP53* and *SMARCA4* respectively, which are transcription factors (TFs), and at the center of network 2 is *CCND1*, which encodes for the Cyclin-D1 protein, a key regulator of the cell cycle.

**Table 2:** MicroRNA expression associated with LTC wine consumption in normal rectal mucosa

miRNA	Mean miRNA expression						Beta^a^	*p*-value	*q*-value
	None		Moderate		High				
	Tumor^b^	Normal	Tumor^b^	Normal	Tumor^b^	Normal			
hsa-miR-100-5p	9.77	10.45	10.67	9.70	7.46	6.67	−0.23	<0.0001	0.0808
hsa-miR-106b-5p	12.95	3.36	11.45	3.46	13.06	2.48	−0.19	0.0031	0.0880
hsa-miR-10b-5p	8.95	11.23	8.84	9.68	8.12	8.43	−0.17	0.0096	0.0915
hsa-miR-1203	1.70	2.41	1.96	2.45	2.11	2.25	−0.18	0.0052	0.0915
hsa-miR-127-3p	2.22	0.93	2.47	0.95	2.16	0.22	−0.13	0.0110	0.0915
hsa-miR-1295b-3p	2.63	2.61	3.05	2.46	3.09	2.34	−0.15	0.0213	0.0980
hsa-miR-130a-3p	5.58	4.26	6.09	4.22	5.49	2.67	−0.16	0.0018	0.0808
hsa-miR-132-3p	0.94	1.25	0.97	1.33	1.61	0.35	−0.15	0.0083	0.0915
hsa-miR-133b	1.17	6.90	1.67	6.55	1.30	4.75	−0.21	0.0002	0.0808
hsa-miR-143-3p	5.29	8.36	6.43	7.80	5.46	6.38	−0.16	0.0139	0.0923
hsa-miR-145-5p	127.50	269.68	145.98	249.57	108.42	201.84	−0.15	0.0178	0.0944
hsa-miR-151a-3p	4.31	1.07	3.81	0.98	4.42	0.59	−0.14	0.0148	0.0923
hsa-miR-15a-5p	6.31	3.18	5.31	3.07	6.66	2.05	−0.21	0.0007	0.0808
hsa-miR-15b-5p	35.15	25.56	29.66	22.44	30.11	19.68	−0.17	0.0102	0.0915
hsa-miR-17-5p	56.07	13.55	47.34	13.08	51.50	11.61	−0.16	0.0222	0.0983
hsa-miR-181a-5p	35.81	25.17	33.88	24.18	30.44	22.21	−0.14	0.0170	0.0944
hsa-miR-184	2.27	2.34	2.39	2.65	2.45	1.86	−0.15	0.0120	0.0923
hsa-miR-193b-3p	10.19	6.06	9.51	5.72	7.59	4.45	−0.15	0.0089	0.0915
hsa-miR-195-5p	3.08	10.86	3.12	9.73	2.95	7.79	−0.20	0.0025	0.0808
hsa-miR-199a-3p	41.35	19.32	40.70	17.58	32.62	14.29	−0.15	0.0204	0.0979
hsa-miR-199a-5p	18.56	7.90	18.42	6.88	14.04	5.28	−0.20	0.0006	0.0808
hsa-miR-199b-5p	3.62	1.17	3.90	0.86	3.70	0.50	−0.17	0.0094	0.0915
hsa-miR-204-3p	0.48	1.00	0.80	0.87	0.72	0.94	−0.16	0.0149	0.0923
hsa-miR-210	22.73	16.21	20.75	14.11	18.23	15.48	−0.06	0.0084	0.0915
hsa-miR-2117	1.45	3.23	1.60	3.10	1.76	2.94	−0.17	0.0087	0.0915
hsa-miR-222-3p	16.17	9.19	15.42	8.63	14.94	7.72	−0.16	0.0113	0.0915
hsa-miR-2278	1.72	1.91	1.61	1.83	1.87	1.80	−0.11	0.0211	0.0980
hsa-miR-25-3p	28.71	11.29	23.88	10.05	26.41	8.62	−0.18	0.0056	0.0915
hsa-miR-27b-3p	32.42	24.57	30.10	22.50	27.54	18.91	−0.16	0.0177	0.0944
hsa-miR-28-5p	1.34	1.87	1.39	1.52	1.43	1.10	−0.16	0.0132	0.0923
hsa-miR-302c-5p	2.02	1.65	2.10	1.38	2.54	1.16	−0.18	0.0070	0.0915
hsa-miR-30a-5p	1.51	3.66	1.61	3.06	1.80	2.18	−0.21	0.0008	0.0808
hsa-miR-30c-5p	7.93	10.29	6.52	9.13	7.10	7.63	−0.16	0.0157	0.0923
hsa-miR-30e-5p	2.78	3.12	2.23	2.79	3.15	2.35	−0.17	0.0115	0.0915
hsa-miR-3164	1.20	2.02	1.70	1.89	1.84	1.62	−0.17	0.0097	0.0915
hsa-miR-3187-5p	3.49	2.41	3.95	2.27	4.27	1.99	−0.18	0.0073	0.0915
hsa-miR-324-5p	4.22	1.73	3.05	1.66	4.34	0.96	−0.17	0.0039	0.0915
hsa-miR-3609	2.56	1.79	2.28	1.94	2.92	1.62	−0.13	0.0195	0.0964
hsa-miR-3616-3p	3.32	2.69	3.26	2.69	4.17	1.95	−0.15	0.0113	0.0915
hsa-miR-3617-5p	3.62	4.04	4.11	3.92	4.22	3.26	−0.15	0.0200	0.0976
hsa-miR-3620-3p	7.21	8.12	7.64	8.91	6.86	8.33	0.08	0.0102	0.0915
hsa-miR-362-5p	2.96	0.51	2.33	0.46	3.34	0.13	−0.17	0.0092	0.0915
hsa-miR-365a-3p	8.13	3.95	7.12	3.72	7.15	2.72	−0.13	0.0133	0.0923
hsa-miR-3680-3p	6.68	6.12	7.01	6.43	7.30	5.53	−0.13	0.0157	0.0923
hsa-miR-378d	0.22	1.58	0.20	1.36	0.29	1.36	−0.17	0.0081	0.0915
hsa-miR-378g	1.11	1.87	1.28	1.76	1.67	1.45	−0.19	0.0045	0.0915
hsa-miR-3922-5p	1.45	1.19	1.72	1.34	1.67	1.08	−0.14	0.0229	0.0998
hsa-miR-3976	2.39	0.95	2.54	1.04	2.59	0.59	−0.17	0.0022	0.0808
hsa-miR-425-3p	12.43	14.70	12.66	15.65	11.95	15.83	0.15	0.0217	0.0980
hsa-miR-425-5p	11.55	6.17	9.75	5.17	10.03	4.54	−0.17	0.0053	0.0915
hsa-miR-4280	3.45	2.99	3.82	2.94	4.50	2.94	−0.16	0.0190	0.0952
hsa-miR-4296	1.72	1.13	2.07	1.00	1.94	0.94	−0.16	0.0177	0.0944
hsa-miR-432-5p	2.55	2.15	2.65	2.10	2.74	1.72	−0.16	0.0103	0.0915
hsa-miR-4450	3.83	3.40	4.29	2.97	4.44	2.89	−0.20	0.0018	0.0808
hsa-miR-4469	1.32	2.22	1.52	1.87	1.36	1.88	−0.16	0.0085	0.0915
hsa-miR-4479	1.90	2.15	2.00	1.98	2.09	1.76	−0.20	0.0024	0.0808
hsa-miR-4657	3.01	1.85	3.29	1.89	3.57	1.51	−0.17	0.0101	0.0915
hsa-miR-4659b-3p	4.25	5.32	4.34	5.05	4.95	4.86	−0.15	0.0223	0.0983
hsa-miR-4660	4.84	4.11	5.24	3.69	5.14	3.44	−0.17	0.0060	0.0915
hsa-miR-4684-3p	1.73	2.14	2.10	2.00	2.26	1.90	−0.17	0.0128	0.0923
hsa-miR-4730	8.99	9.06	7.68	11.03	7.80	7.51	−0.07	0.0151	0.0923
hsa-miR-4748	4.79	4.78	4.96	4.62	5.24	4.19	−0.16	0.0147	0.0923
hsa-miR-484	1.67	1.67	1.36	1.42	2.33	0.92	−0.19	0.0012	0.0808
hsa-miR-497-5p	1.13	5.79	1.17	5.46	1.32	4.14	−0.22	0.0004	0.0808
hsa-miR-5008-3p	1.81	0.79	1.96	0.73	2.19	0.39	−0.14	0.0153	0.0923
hsa-miR-518c-5p	1.57	1.91	1.86	1.92	1.85	1.57	−0.16	0.0137	0.0923
hsa-miR-525-5p	1.68	1.71	1.78	1.33	1.82	1.02	−0.15	0.0206	0.0979
hsa-miR-532-5p	4.79	1.01	3.90	0.72	5.23	0.34	−0.17	0.0081	0.0915
hsa-miR-548aa	1.93	2.14	1.68	2.40	1.72	2.08	0.01	0.0042	0.0915
hsa-miR-548am-5p	0.91	1.12	0.69	1.58	1.06	0.75	−0.09	<0.0001	0.0808
hsa-miR-551b-5p	1.31	0.99	1.56	0.95	1.60	0.85	−0.17	0.0110	0.0915
hsa-miR-566	3.08	3.52	3.56	3.86	3.75	3.12	−0.11	0.0157	0.0923
hsa-miR-5685	0.76	2.23	1.08	2.04	1.22	2.16	−0.16	0.0182	0.0951
hsa-miR-595	5.41	5.68	4.83	5.72	4.25	4.67	−0.05	0.0113	0.0915
hsa-miR-615-3p	1.06	1.09	0.98	1.28	1.22	1.06	−0.02	0.0187	0.0952
hsa-miR-636	8.76	10.83	9.98	11.69	9.10	11.91	0.13	0.0215	0.0980
hsa-miR-6500-3p	3.00	3.37	3.44	3.00	3.92	2.96	−0.16	0.0178	0.0944
hsa-miR-652-3p	1.66	0.81	1.72	0.73	2.06	0.38	−0.16	0.0123	0.0923
hsa-miR-6716-3p	5.22	4.23	4.10	7.60	4.79	3.20	−0.04	0.0166	0.0944
hsa-miR-877-3p	24.17	31.59	26.55	34.59	25.26	34.15	0.13	0.0124	0.0923
hsa-miR-92a-1-5p	0.57	0.78	0.55	0.70	0.82	0.60	−0.16	0.0188	0.0952
hsa-miR-93-5p	37.48	12.83	31.04	11.72	35.90	10.33	−0.17	0.0111	0.0915
hsa-miR-99a-5p	6.48	3.59	8.31	3.05	5.56	1.98	−0.15	0.0063	0.0915
hsa-miR-99b-5p	4.65	4.06	5.00	3.99	3.93	2.99	−0.15	0.0159	0.0923

^a^Adjusted for age at diagnosis, study center, sex, and current smoking

^b^Tumor miRNA expression shown for information only; this was not used in the analysis and was not associated with alcohol use

No other alcohol variables, including total alcohol consumption, beer consumption, and hard liquor consumption were associated with miRNA expression level. While hard liquor was associated with expression levels of 21 miRNAs, none of these remained significant after adjusting for cigarette smoking status.

## Discussion

Long-term current wine consumption significantly altered miRNA expression in normal rectal mucosa (NRM) of 84 miRNAs. These findings support the hypothesis that alcohol consumption is able to influence miRNA expression. Furthermore, they suggest that different forms of alcohol are able to impact miRNA expression in a distinctive manner, as we only detected associations for LTC wine after adjusting for multiple comparisons. Two miRNAs associated across LTC wine in NRM, hsa-miR-210 and hsa-miR-92a-1-5p, also significantly worsened ACM when expression levels were higher. Hsa-miR-210 was directly associated with expression of a multitude of mRNAs in NCRM, suggesting miRNAs that are influenced by wine consumption alter mRNAs in NCRM, and this disruption to normal pathways may contribute to a variety of disease processes, affecting ACM.

Previously, we reported associations between alcohol intake and rectal cancer risk in these subjects, and we did not see significant associations for liquor and wine in general; however, we found that, in subjects who were non-users of NSAIDs, high long-term liquor or beer intake significantly increased risk of developing rectal cancer compared to subjects who did use NSAIDs [39]. This finding suggests that liquor and beer consumption have an inherent inflammatory effect that is ameliorated by NSAID use. Alcohol metabolism is known to lead to reactive oxygen species production, which may stimulate nuclear factor kappa-light-chain-enhancer of activated B cells (NF-κB), a pro-inflammatory factor [5]. A German study found that not drinking wine recently and over a lifetime significantly increased chance of death overall and for CRC specifically compared to subjects who drank ≤ 12 g/day of wine [7]. As we saw that NSAIDs moderated risk in liquor users only, it is possible that wine consumption does not trigger the same inflammatory response that liquor consumption does, or instead instigates an anti-inflammatory response. Higher beer and liquor intake, but not wine intake, was associated with greater risk of incidence of gout [40] in a study conducted by Choi et al. Gout, an inflammatory disease, is generally thought to be preceded by hyperuricemia, a condition tied to alcohol consumption, and Choi et al. found that beer and liquor, but not wine, increased serum uric acid levels [16]. Ethanol itself increases serum uric acid, through decreased excretion as well as increased production, and alcoholic beverages that contain more purines, such as beer, which contains high levels of guanosine, and liquor to a lesser extent, exacerbate this effect as these molecules are metabolized into uric acid [16, 41]. As uric acid indicates elevated oxidative stress, Choi et al. proposed anti-oxidant non-alcoholic components of wine, such as polyphenols, may prevent uric acid levels from rising with moderate wine consumption [16].

Resveratrol, an anti-oxidative polyphenol found in grapes, has been shown to mitigate many pathological responses, including chronic inflammation and cancer [26]. One hypothesis as to how resveratrol is able to influence so many disease states, and the multitude of biological processes involved, is by altering the expression of miRNAs [26]. Resveratrol has been linked to the upregulation of hsa-miR-663 [26, 42]; however, we did not replicate this finding. Seven miRNAs associated across LTC wine consumption in NRM have been previously associated with resveratrol presence in human CRC cells in other studies [20, 43]. Hsa-miR-100-5p, hsa-miR-17-5p, hsa-miR-181a-5p, hsa-miR-25-3p, and hsa-miR-30e-5p were all downregulated with increased LTC wine consumption, and were previously shown to have decreased expression with the addition of resveratrol to cells [43]. Hsa-miR-30c-5p was downregulated in our dataset and hsa-miR-615-3p was upregulated between no and moderate LTC wine intake, but decreased between moderate and high consumption in our data; both hsa-miR-30c-5p and hsa-miR-615-3p were shown to increase with resveratrol addition by Tili et al. [43].

Quercetin, another polyphenol found in red wines, influences miRNA expression [20], and has been studied with expression of select miRNAs in mouse models [44], as well as in various human cancers [45–47]. In the mouse model, mmu-miR-181a, 324-5p, 210, and 99b were upregulated, and 30c-1 (stem loop to miR-30c-5p in humans) and 10b were downregulated [44] with the addition of quercetin. We saw significant downregulation across LTC wine consumption for all six miRNAs. In colon [46] and renal cancers [47], quercetin combined with resveratrol and hyperoside respectively decreased expression of hsa-miR-27a; we did not replicate this finding.

As alcohol is known to influence many disease states [1, 2], we analyzed the 84 miRNAs associated significantly with wine LTC for associations with ACM across colon and rectal cases combined. Two miRNAs, hsa-miR-210 and hsa-miR-92a-1-5p, significantly worsened ACM when expression increased in NCRM. These miRNAs were downregulated in NRM across LTC wine consumption levels. This finding suggests that moderate to high wine consumption decreases miRNAs that potentially are oncogenic, as they are shown to worsen CRC survival with greater expression. As these miRNAs were only dysregulated in NRM, it is likely that this altered expression disrupts normal biological pathways, contributing to overall disease states and ACM, rather than CRC pathogenesis specifically.

To identify potentially dysregulated pathways, we tested associations between mRNAs and hsa-miR-210 and hsa-miR-92a-1-5p in NCRM. No mRNAs were associated significantly with hsa-miR-92a-1-5p; 3169 mRNAs were associated with hsa-miR-210 expression when a q-value of <0.5 was applied and a fold change restriction of ≥ 1.5 or <0.67 were applied. Of these, 14 displayed the typical inverse relationship of miRNA::mRNA interactions. While miRNAs are known to upregulate target gene expression in certain conditions [48], it is likely external factors influence the expression of these genes in concert with hsa-miR-210. A multitude of genes upregulate *TP53* and *CCND1*; it is therefore possible that one, or a combination, of these genes is responsible for the positive beta coefficient between hsa-miR-210 and these genes. TFs form feed forward loops (FFLs) with miRNAs and target genes [49, 50]. In certain FFLs, a TF will upregulate a miRNA and its target gene, creating an ‘incoherent’ FFL. Both *SMARCA4* and *TP53* display numerous ‘direct activation’ connections, that are either bidirectional or leading away from the center, indicating their positive regulatory capacity. Conversely, *CCND1* is the target of regulation by a larger majority of genes in its network. *GTF3C4, HOXB13, ATAD2* are TFs that may activate *CCND1*. Given the interconnectedness of the networks and the regulatory ability of so many of the genes in the network, it is possible other genes, specifically TFs, influence the direct association between these mRNAs and hsa-miR-210.

Many of the studies cited from the literature used carcinoma tissue; we looked at miRNA expression in both NCRM and differential miRNA expression, between paired carcinoma and NCRM, to determine if alcohol influenced miRNA expression. We did not see any associations between alcohol use and differential miRNA expression between carcinoma and NCRM; however, we did see that LTC wine use influenced the expression of 84 miRNAs in NRM only. As these miRNAs were not associated with differential expression, we can infer that these miRNAs’ expression, while influenced by alcohol consumption, does not differ within the individual’s carcinoma and normal mucosa, and therefore is behaving similarly to carcinoma tissue miRNA expression reported in other studies. As these miRNAs are not differentially expressed, it is possible that alcohol use contributes to the global dysregulation of miRNAs, and is one of many factors influencing disease progression. As we only see associations with LTC wine, it may be the non-alcoholic components unique to wine, including phytochemicals, that are responsible for the altered expression of these miRNAs. As these data used colorectal tissue, other tissue types may have different associations with alcoholic beverages and miRNA expression.

Our study has many strengths, including our large sample size. This enabled us to analyze colon and rectal cases separately; as we only detected significant results for NRM expression, we may not have found significant results if we had combined colon and rectal cancers or examined only colon cancer cases. Additionally, we used a microarray platform to collect miRNA expression data, which enabled us to take a discovery approach. This is valuable, as miRNA expression associated with alcohol use has not been studied extensively. As we have already stated, this method has high reliability, which is another asset, as well as good concordance with other methods [32, 33]. Another strength of this study is our detailed diet history, which enabled us to collect data on specific alcohol consumption. We have shown that liquor and wine were associated with different miRNAs, and we would not have been able to distinguish these miRNAs otherwise. While we did not replicate all of the findings in the literature, it is possible that this is due to our large platform and the fact that we corrected for multiple comparisons. Additionally, many of the studies calculate alcohol consumption using different amounts of consumption, use certain g/day cutoffs, or another classification method, and as such our results may not be directly comparable to others. In a review done by Milenkovic et al. on miRNAs and polyphenols, they report that the direction of dysregulation of specific miRNAs associated with resveratrol in multiple studies varies, and they attribute this to differences in cell lines used, resveratrol concentrations, and time of resveratrol exposure; this finding highlights the potential sensitivity of miRNAs to exogenous factors as well as the potential difficulties in comparing findings across studies [20]. Finally, while there have been studies investigating polyphenol influence on miRNA expression for many cancers, there is most often one or two studies focusing on any given cancer, some using a combination of different polyphenols, which may or may not be present in the same foods, and some consider a select few miRNAs. Additionally, as some studies use animal models, or different human cell lines, these results may not be directly comparable. The study whose findings most closely matched our own was one that used human CRC cells and performed a microarray analysis [43]. We encourage others to continue to investigate polyphenol contribution to miRNA expression patterns in disease states, particularly CRC, in order to better determine how these molecules influence miRNAs and disease states.

In this study, we show that miRNAs are influenced by wine consumption. As significant results were found only for NRM, it is possible that non-alcoholic components of wine alter the normal mucosa and carcinoma tissue of rectal cancer subjects equally and in a manner that contributes to the overall dysregulation of genes in CRC.

## Electronic supplementary material

Below is the link to the electronic supplementary material.

Supplementary material 1 (DOCX 154 KB)

Supplementary material 2 (DOCX 1248 KB)
