# Supplementary material for: Alterations in microRNA expression associated with alcohol consumption in rectal cancer subjects
Source: Cancer Causes Control. 2017 Mar 16;28(6):545–55. doi: 10.1007/s10552-017-0882-2 (PMC5400787; doi:10.1007/s10552-017-0882-2)
Supplement: Supplementary file 1 — Supplementary material 1 (DOCX 154 KB) [file 10552_2017_882_MOESM1_ESM.docx]

| S1. Associations between wine LTC miRNAs and all-cause mortality for colon and rectal cancer subjects. | | | | | | |
| --- | --- | --- | --- | --- | --- | --- |
| **miRNA** | **25th%ile^1^** | **75th%ile^1^** | **HR^2^** | **95% (CI)** | | **p-value** |
| hsa-miR-100-5p | 1.95 | 3.41 | 0.95 | (0.86, | 1.03) | 0.21 |
| hsa-miR-106b-5p | 0.00 | 2.57 | 1.04 | (0.87, | 1.23) | 0.68 |
| hsa-miR-10b-5p | 3.09 | 4.17 | 1.00 | (0.92, | 1.07) | 0.90 |
| hsa-miR-1203 | 0.00 | 2.03 | 1.06 | (0.90, | 1.26) | 0.46 |
| hsa-miR-127-3p | 0.00 | 0.00 | 1.06 | (0.91, | 1.24) | 0.47 |
| hsa-miR-1295b-3p | 0.00 | 2.20 | 1.07 | (0.91, | 1.25) | 0.43 |
| hsa-miR-130a-3p | 0.00 | 2.51 | 1.05 | (0.90, | 1.23) | 0.54 |
| hsa-miR-132-3p | 0.00 | 0.00 | 0.98 | (0.85, | 1.14) | 0.84 |
| hsa-miR-133b | 0.00 | 2.63 | 0.99 | (0.84, | 1.16) | 0.85 |
| hsa-miR-143-3p | 2.06 | 3.30 | 1.02 | (0.94, | 1.10) | 0.70 |
| hsa-miR-145-5p | 6.84 | 7.98 | 1.02 | (0.91, | 1.14) | 0.74 |
| hsa-miR-151a-3p | 0.00 | 0.83 | 1.06 | (0.98, | 1.16) | 0.17 |
| hsa-miR-15a-5p | 0.00 | 2.36 | 1.08 | (0.93, | 1.26) | 0.30 |
| hsa-miR-15b-5p | 3.82 | 4.75 | 1.01 | (0.94, | 1.07) | 0.88 |
| hsa-miR-17-5p | 3.36 | 4.02 | 1.02 | (0.97, | 1.08) | 0.45 |
| hsa-miR-181a-5p | 4.41 | 4.77 | 1.00 | (0.93, | 1.08) | 0.99 |
| hsa-miR-184 | 0.00 | 2.16 | 1.06 | (0.91, | 1.24) | 0.44 |
| hsa-miR-193b-3p | 1.65 | 2.79 | 1.00 | (0.92, | 1.09) | 0.98 |
| hsa-miR-195-5p | 2.40 | 3.65 | 1.01 | (0.93, | 1.09) | 0.85 |
| hsa-miR-199a-3p | 3.56 | 4.53 | 1.01 | (0.95, | 1.08) | 0.71 |
| hsa-miR-199a-5p | 2.27 | 3.26 | 1.01 | (0.94, | 1.08) | 0.79 |
| hsa-miR-199b-5p | 0.00 | 0.57 | 1.03 | (0.97, | 1.09) | 0.35 |
| hsa-miR-204-3p | 0.00 | 0.81 | 1.00 | (0.91, | 1.09) | 0.93 |
| **hsa-miR-210^3^** | **3.61** | **4.20** | **1.12** | **(1.03,** | **1.21)** | **0.00** |
| hsa-miR-2117 | 0.00 | 2.41 | 1.05 | (0.89, | 1.24) | 0.56 |
| hsa-miR-222-3p | 2.87 | 3.50 | 1.03 | (0.96, | 1.10) | 0.43 |
| hsa-miR-2278 | 0.00 | 1.50 | 1.04 | (0.90, | 1.19) | 0.62 |
| hsa-miR-25-3p | 2.58 | 3.74 | 0.99 | (0.92, | 1.07) | 0.80 |
| hsa-miR-27b-3p | 3.95 | 4.80 | 1.01 | (0.95, | 1.08) | 0.72 |
| hsa-miR-28-5p | 0.00 | 0.88 | 1.04 | (0.96, | 1.14) | 0.33 |
| hsa-miR-302c-5p | 0.00 | 1.84 | 0.95 | (0.81, | 1.10) | 0.48 |
| hsa-miR-30a-5p | 0.00 | 2.15 | 1.05 | (0.91, | 1.22) | 0.49 |
| hsa-miR-30c-5p | 2.62 | 3.53 | 1.01 | (0.95, | 1.08) | 0.71 |
| hsa-miR-30e-5p | 0.00 | 2.27 | 1.05 | (0.89, | 1.23) | 0.57 |
| hsa-miR-3164 | 0.00 | 2.03 | 0.89 | (0.76, | 1.03) | 0.12 |
| hsa-miR-3187-5p | 0.00 | 2.31 | 0.98 | (0.83, | 1.15) | 0.78 |
| hsa-miR-324-5p | 0.00 | 1.14 | 1.02 | (0.92, | 1.13) | 0.70 |
| hsa-miR-3609 | 0.00 | 1.80 | 0.92 | (0.79, | 1.07) | 0.26 |
| hsa-miR-3616-3p | 0.00 | 2.30 | 0.98 | (0.84, | 1.15) | 0.80 |
| hsa-miR-3617-5p | 0.00 | 2.80 | 0.96 | (0.81, | 1.14) | 0.64 |
| hsa-miR-3620-3p | 2.90 | 3.33 | 1.00 | (0.95, | 1.04) | 0.84 |
| hsa-miR-362-5p | 0.00 | 0.00 | 0.95 | (0.79, | 1.13) | 0.55 |
| hsa-miR-365a-3p | 0.00 | 2.34 | 0.96 | (0.83, | 1.12) | 0.64 |
| hsa-miR-3680-3p | 2.59 | 3.13 | 1.00 | (0.96, | 1.05) | 0.87 |
| hsa-miR-378d | 0.00 | 0.83 | 1.04 | (0.94, | 1.14) | 0.45 |
| hsa-miR-378g | 0.00 | 1.57 | 1.06 | (0.92, | 1.21) | 0.45 |
| hsa-miR-3922-5p | 0.00 | 1.39 | 1.07 | (0.93, | 1.24) | 0.34 |
| hsa-miR-3976 | 0.00 | 0.34 | 0.98 | (0.94, | 1.02) | 0.27 |
| hsa-miR-425-3p | 3.44 | 4.01 | 1.02 | (0.95, | 1.09) | 0.56 |
| hsa-miR-425-5p | 1.82 | 2.99 | 1.03 | (0.96, | 1.12) | 0.40 |
| hsa-miR-4280 | 0.00 | 2.59 | 1.05 | (0.88, | 1.25) | 0.60 |
| hsa-miR-4296 | 0.00 | 1.37 | 1.05 | (0.91, | 1.21) | 0.46 |
| hsa-miR-432-5p | 0.00 | 2.18 | 0.97 | (0.83, | 1.14) | 0.70 |
| hsa-miR-4450 | 0.00 | 2.68 | 1.06 | (0.89, | 1.27) | 0.52 |
| hsa-miR-4469 | 0.00 | 1.66 | 0.97 | (0.84, | 1.12) | 0.65 |
| hsa-miR-4479 | 0.00 | 1.71 | 0.99 | (0.86, | 1.14) | 0.91 |
| hsa-miR-4657 | 0.00 | 1.98 | 1.04 | (0.90, | 1.21) | 0.57 |
| hsa-miR-4659b-3p | 0.00 | 2.89 | 0.97 | (0.81, | 1.16) | 0.70 |
| hsa-miR-4660 | 0.00 | 2.83 | 1.04 | (0.87, | 1.25) | 0.66 |
| hsa-miR-4684-3p | 0.00 | 2.10 | 0.92 | (0.79, | 1.08) | 0.31 |
| hsa-miR-4730 | 0.00 | 2.92 | 0.99 | (0.85, | 1.14) | 0.85 |
| hsa-miR-4748 | 2.15 | 3.04 | 1.03 | (0.97, | 1.09) | 0.40 |
| hsa-miR-484 | 0.00 | 0.64 | 1.05 | (0.98, | 1.12) | 0.15 |
| hsa-miR-497-5p | 0.00 | 2.76 | 0.96 | (0.80, | 1.14) | 0.62 |
| hsa-miR-5008-3p | 0.00 | 0.00 | 0.97 | (0.84, | 1.11) | 0.62 |
| hsa-miR-518c-5p | 0.00 | 1.82 | 1.10 | (0.96, | 1.27) | 0.18 |
| hsa-miR-525-5p | 0.00 | 1.47 | 1.03 | (0.91, | 1.15) | 0.68 |
| hsa-miR-532-5p | 0.00 | 0.00 | 0.99 | (0.88, | 1.12) | 0.93 |
| hsa-miR-548aa | 0.00 | 1.11 | 1.06 | (0.96, | 1.17) | 0.23 |
| hsa-miR-548am-5p | 0.00 | 0.73 | 0.97 | (0.88, | 1.06) | 0.49 |
| hsa-miR-551b-5p | 0.00 | 1.41 | 0.95 | (0.82, | 1.10) | 0.50 |
| hsa-miR-566 | 1.53 | 2.49 | 1.03 | (0.95, | 1.11) | 0.51 |
| hsa-miR-5685 | 0.00 | 1.65 | 0.92 | (0.81, | 1.06) | 0.25 |
| hsa-miR-595 | 1.81 | 2.85 | 0.94 | (0.88, | 1.01) | 0.11 |
| hsa-miR-615-3p | 0.00 | 1.13 | 0.90 | (0.79, | 1.03) | 0.12 |
| hsa-miR-636 | 3.23 | 3.73 | 0.99 | (0.92, | 1.07) | 0.87 |
| hsa-miR-6500-3p | 0.00 | 2.52 | 0.96 | (0.81, | 1.13) | 0.62 |
| hsa-miR-652-3p | 0.00 | 0.00 | 1.06 | (0.91, | 1.24) | 0.48 |
| hsa-miR-6716-3p | 0.00 | 0.72 | 1.00 | (0.95, | 1.06) | 0.95 |
| hsa-miR-877-3p | 4.62 | 5.19 | 0.97 | (0.88, | 1.07) | 0.51 |
| **hsa-miR-92a-1-5p** | **0.00** | **0.89** | **1.20** | **(1.04,** | **1.38)** | **0.01** |
| hsa-miR-93-5p | 3.09 | 3.97 | 1.02 | (0.95, | 1.10) | 0.52 |
| hsa-miR-99a-5p | 0.00 | 2.03 | 1.03 | (0.89, | 1.19) | 0.66 |
| hsa-miR-99b-5p | 0.00 | 2.22 | 1.04 | (0.89, | 1.22) | 0.62 |
| ^1^Normal expression, adjusted for age, center, sex, and AJCC. | | | | |  |  |
| ^2^The unit of change for the HR is the interquartile range. | | | |  |  |  |
| ^3^Bolded terms are statistically significant. | | |  |  |  |  |
