# Supplementary material for: Alterations in microRNA expression associated with alcohol consumption in rectal cancer subjects
Source: Cancer Causes Control. 2017 Mar 16;28(6):545–55. doi: 10.1007/s10552-017-0882-2 (PMC5400787; doi:10.1007/s10552-017-0882-2)
Supplement: Supplementary file 2 — Supplementary material 2 (DOCX 1248 KB) [file 10552_2017_882_MOESM2_ESM.docx]

|  |  |  |  | **P-values** | |  |
| --- | --- | --- | --- | --- | --- | --- |
| **Gene** | **mRNA Ensemble Gene (ENSG) ID** | **Normal Mean miRNA Expression** | **Beta^1^** | **Raw** | **FDR** | **Fold Change (T/N)** |
| A1CF | ENSG00000148584 | 75.63 | 0.22 | 0.0025 | 0.0342 | 0.67 |
| AAAS | ENSG00000094914 | 50.02 | 0.21 | 0.0045 | 0.0352 | 1.60 |
| AACS | ENSG00000081760 | 154.15 | 0.19 | 0.0085 | 0.0399 | 1.43 |
| AAGAB | ENSG00000103591 | 105.02 | 0.18 | 0.0146 | 0.0481 | 1.41 |
| AARS | ENSG00000090861 | 114.45 | 0.18 | 0.0135 | 0.0464 | 2.51 |
| AARS2 | ENSG00000124608 | 65.98 | 0.23 | 0.0016 | 0.0342 | 1.43 |
| AASDH | ENSG00000157426 | 36.65 | 0.20 | 0.0058 | 0.0364 | 1.28 |
| AATF | ENSG00000108270 | 50.62 | 0.18 | 0.0143 | 0.0476 | 2.44 |
| ABAT | ENSG00000183044 | 54.36 | 0.18 | 0.0161 | 0.0500 | 1.89 |
| ABCA10 | ENSG00000154263 | 54.49 | 0.19 | 0.0131 | 0.0460 | 0.42 |
| ABCA2 | ENSG00000107331 | 210.39 | 0.22 | 0.0041 | 0.0349 | 1.79 |
| ABCA5 | ENSG00000154265 | 319.82 | 0.21 | 0.0106 | 0.0427 | 0.52 |
| ABCB10 | ENSG00000135776 | 46.48 | 0.18 | 0.0154 | 0.0491 | 1.75 |
| ABCB6 | ENSG00000115657 | 20.98 | 0.23 | 0.0042 | 0.0350 | 2.50 |
| ABCB7 | ENSG00000131269 | 36.45 | 0.23 | 0.0025 | 0.0342 | 2.10 |
| ABCC10 | ENSG00000124574 | 120.01 | 0.18 | 0.0146 | 0.0481 | 1.75 |
| ABCC3 | ENSG00000108846 | 893.89 | 0.23 | 0.0028 | 0.0342 | 0.98 |
| ABCC6 | ENSG00000091262 | 68.26 | 0.24 | 0.0019 | 0.0342 | 1.01 |
| ABCD1 | ENSG00000101986 | 35.03 | 0.22 | 0.0043 | 0.0351 | 1.69 |
| ABCD3 | ENSG00000117528 | 199.15 | 0.21 | 0.0055 | 0.0363 | 1.08 |
| ABCD4 | ENSG00000119688 | 106.63 | 0.22 | 0.0029 | 0.0342 | 1.33 |
| ABCF1 | ENSG00000204574 | 111.89 | 0.18 | 0.0130 | 0.0458 | 1.80 |
| ABCF2 | ENSG00000033050 | 56.73 | 0.17 | 0.0137 | 0.0467 | 2.23 |
| ABCG1 | ENSG00000160179 | 116.31 | 0.20 | 0.0095 | 0.0413 | 1.10 |
| ABHD10 | ENSG00000144827 | 31.70 | 0.21 | 0.0045 | 0.0352 | 1.99 |
| ABHD11 | ENSG00000106077 | 83.58 | 0.18 | 0.0116 | 0.0439 | 1.70 |
| ABHD12 | ENSG00000100997 | 80.90 | 0.20 | 0.0055 | 0.0363 | 2.21 |
| ABHD14B | ENSG00000114779 | 80.99 | 0.23 | 0.0016 | 0.0342 | 1.51 |
| ABHD15 | ENSG00000168792 | 52.03 | 0.19 | 0.0076 | 0.0382 | 1.23 |
| ABHD16A | ENSG00000204427 | 80.32 | 0.19 | 0.0104 | 0.0424 | 1.83 |
| ABHD16B | ENSG00000183260 | 17.46 | 0.19 | 0.0131 | 0.0460 | 2.10 |
| ABHD2 | ENSG00000140526 | 432.89 | 0.23 | 0.0042 | 0.0350 | 2.67 |
| ABHD3 | ENSG00000158201 | 61.11 | 0.18 | 0.0151 | 0.0487 | 0.70 |
| ABHD4 | ENSG00000100439 | 35.63 | 0.21 | 0.0039 | 0.0345 | 1.21 |
| ABHD6 | ENSG00000163686 | 27.53 | 0.19 | 0.0092 | 0.0408 | 1.12 |
| ABI1 | ENSG00000136754 | 133.36 | 0.19 | 0.0106 | 0.0427 | 1.54 |
| ABLIM1 | ENSG00000099204 | 602.83 | 0.18 | 0.0114 | 0.0437 | 1.39 |
| ABLIM2 | ENSG00000163995 | 39.03 | 0.24 | 0.0020 | 0.0342 | 3.35 |
| ABO | ENSG00000175164 | 79.57 | 0.27 | 0.0002 | 0.0342 | 1.04 |
| ABP1 | ENSG00000002726 | 364.37 | 0.25 | 0.0008 | 0.0342 | 0.53 |
| ABR | ENSG00000159842 | 866.93 | 0.18 | 0.0144 | 0.0477 | 1.04 |
| ABT1 | ENSG00000146109 | 27.47 | 0.19 | 0.0125 | 0.0451 | 1.83 |
| AC004410.1 | ENSG00000005206 | 204.69 | 0.23 | 0.0030 | 0.0342 | 1.26 |
| AC005013.1 | ENSG00000255690 | 19.81 | 0.20 | 0.0097 | 0.0415 | 1.52 |
| AC005280.1 | ENSG00000170468 | 23.54 | 0.25 | 0.0012 | 0.0342 | 1.71 |
| AC007405.7 | ENSG00000204334 | 1.15 | 0.24 | 0.0085 | 0.0399 | 3.96 |
| AC008073.5 | ENSG00000115128 | 27.10 | 0.20 | 0.0070 | 0.0376 | 2.22 |
| AC009403.2 | ENSG00000216895 | 12.49 | 0.19 | 0.0138 | 0.0468 | 1.59 |
| AC012313.1 | ENSG00000232098 | 20.63 | 0.19 | 0.0111 | 0.0433 | 1.72 |
| AC012652.1 | ENSG00000187446 | 407.39 | 0.21 | 0.0063 | 0.0370 | 0.85 |
| AC022098.1 | ENSG00000141854 | 28.55 | 0.20 | 0.0052 | 0.0359 | 1.49 |
| AC062017.1 | ENSG00000222020 | 2.41 | 0.20 | 0.0154 | 0.0491 | 1.08 |
| AC073346.2 | ENSG00000214194 | 11.21 | 0.24 | 0.0044 | 0.0351 | 2.26 |
| AC091153.1 | ENSG00000141456 | 65.13 | 0.20 | 0.0081 | 0.0391 | 1.78 |
| ACAA1 | ENSG00000060971 | 118.29 | 0.20 | 0.0083 | 0.0395 | 1.21 |
| ACACA | ENSG00000132142 | 238.11 | 0.20 | 0.0062 | 0.0368 | 2.58 |
| ACAD10 | ENSG00000111271 | 116.35 | 0.25 | 0.0007 | 0.0342 | 1.22 |
| ACADS | ENSG00000122971 | 92.51 | 0.21 | 0.0078 | 0.0386 | 0.55 |
| ACADVL | ENSG00000072778 | 702.24 | 0.20 | 0.0086 | 0.0399 | 0.84 |
| ACAP3 | ENSG00000131584 | 185.07 | 0.20 | 0.0072 | 0.0377 | 1.38 |
| ACBD3 | ENSG00000182827 | 120.36 | 0.23 | 0.0020 | 0.0342 | 1.60 |
| ACBD4 | ENSG00000181513 | 39.46 | 0.20 | 0.0114 | 0.0437 | 1.11 |
| ACBD5 | ENSG00000107897 | 70.83 | 0.28 | 0.0002 | 0.0342 | 1.73 |
| ACCS | ENSG00000110455 | 87.77 | 0.20 | 0.0107 | 0.0428 | 1.20 |
| ACER2 | ENSG00000177076 | 17.12 | 0.19 | 0.0110 | 0.0432 | 1.14 |
| ACER3 | ENSG00000078124 | 189.94 | 0.23 | 0.0022 | 0.0342 | 0.85 |
| ACIN1 | ENSG00000100813 | 498.82 | 0.18 | 0.0097 | 0.0415 | 1.17 |
| ACLY | ENSG00000131473 | 191.40 | 0.19 | 0.0081 | 0.0391 | 2.51 |
| ACO1 | ENSG00000122729 | 102.74 | 0.22 | 0.0025 | 0.0342 | 1.31 |
| ACO2 | ENSG00000100412 | 310.23 | 0.20 | 0.0071 | 0.0376 | 0.93 |
| ACOT1 | ENSG00000184227 | 9.34 | 0.19 | 0.0128 | 0.0456 | 1.19 |
| ACOT11 | ENSG00000162390 | 267.33 | 0.20 | 0.0061 | 0.0368 | 0.88 |
| ACOT13 | ENSG00000112304 | 92.82 | 0.22 | 0.0027 | 0.0342 | 1.22 |
| ACOT2 | ENSG00000119673 | 17.90 | 0.22 | 0.0032 | 0.0342 | 1.23 |
| ACOT8 | ENSG00000101473 | 80.07 | 0.20 | 0.0053 | 0.0361 | 1.67 |
| ACOX1 | ENSG00000161533 | 356.02 | 0.19 | 0.0133 | 0.0462 | 1.26 |
| ACOX2 | ENSG00000168306 | 29.90 | 0.24 | 0.0020 | 0.0342 | 1.72 |
| ACOX3 | ENSG00000087008 | 38.90 | 0.22 | 0.0027 | 0.0342 | 1.23 |
| ACP1 | ENSG00000143727 | 94.70 | 0.24 | 0.0020 | 0.0342 | 2.12 |
| ACP2 | ENSG00000134575 | 53.46 | 0.19 | 0.0100 | 0.0419 | 1.16 |
| ACP6 | ENSG00000162836 | 27.29 | 0.25 | 0.0006 | 0.0342 | 2.13 |
| ACPL2 | ENSG00000155893 | 32.24 | 0.19 | 0.0125 | 0.0451 | 2.60 |
| ACPP | ENSG00000014257 | 17.23 | 0.25 | 0.0016 | 0.0342 | 1.00 |
| ACSF2 | ENSG00000167107 | 122.65 | 0.23 | 0.0034 | 0.0342 | 0.76 |
| ACSF3 | ENSG00000176715 | 106.90 | 0.19 | 0.0133 | 0.0462 | 1.38 |
| ACSL3 | ENSG00000123983 | 154.90 | 0.24 | 0.0010 | 0.0342 | 1.79 |
| ACSL5 | ENSG00000197142 | 392.81 | 0.23 | 0.0014 | 0.0342 | 1.86 |
| ACSS1 | ENSG00000154930 | 150.67 | 0.21 | 0.0053 | 0.0361 | 1.98 |
| ACSS2 | ENSG00000131069 | 459.31 | 0.26 | 0.0006 | 0.0342 | 0.90 |
| ACTR1B | ENSG00000115073 | 93.46 | 0.22 | 0.0036 | 0.0342 | 1.68 |
| ACTR2 | ENSG00000138071 | 371.05 | 0.19 | 0.0101 | 0.0421 | 1.71 |
| ACTR3B | ENSG00000133627 | 17.44 | 0.20 | 0.0090 | 0.0405 | 2.05 |
| ACTR3C | ENSG00000106526 | 96.82 | 0.20 | 0.0050 | 0.0356 | 1.51 |
| ACTR8 | ENSG00000113812 | 53.13 | 0.23 | 0.0009 | 0.0342 | 1.74 |
| ACVR1 | ENSG00000115170 | 48.37 | 0.20 | 0.0055 | 0.0363 | 1.77 |
| ACVR1B | ENSG00000135503 | 210.39 | 0.26 | 0.0004 | 0.0342 | 1.18 |
| ACVR1C | ENSG00000123612 | 24.92 | 0.24 | 0.0018 | 0.0342 | 0.81 |
| ACVR2A | ENSG00000121989 | 71.12 | 0.20 | 0.0078 | 0.0386 | 1.32 |
| ACVR2B | ENSG00000114739 | 22.32 | 0.22 | 0.0036 | 0.0342 | 1.94 |
| ACVRL1 | ENSG00000139567 | 269.17 | 0.22 | 0.0029 | 0.0342 | 0.48 |
| ACY1 | ENSG00000243989 | 55.45 | 0.22 | 0.0037 | 0.0342 | 1.84 |
| ACY3 | ENSG00000132744 | 9.48 | 0.23 | 0.0028 | 0.0342 | 1.12 |
| ADAM10 | ENSG00000137845 | 219.68 | 0.23 | 0.0022 | 0.0342 | 1.45 |
| ADAM12 | ENSG00000148848 | 36.42 | 0.20 | 0.0093 | 0.0409 | 3.86 |
| ADAM15 | ENSG00000143537 | 199.55 | 0.22 | 0.0028 | 0.0342 | 1.79 |
| ADAM9 | ENSG00000168615 | 223.63 | 0.26 | 0.0015 | 0.0342 | 1.86 |
| ADAMTS12 | ENSG00000151388 | 20.95 | 0.20 | 0.0161 | 0.0500 | 9.29 |
| ADAMTS13 | ENSG00000160323 | 28.29 | 0.23 | 0.0034 | 0.0342 | 1.21 |
| ADAMTSL5 | ENSG00000185761 | 14.96 | 0.24 | 0.0017 | 0.0342 | 1.85 |
| ADAR | ENSG00000160710 | 680.14 | 0.20 | 0.0071 | 0.0376 | 1.77 |
| ADCK3 | ENSG00000163050 | 107.12 | 0.19 | 0.0089 | 0.0403 | 1.71 |
| ADCK4 | ENSG00000123815 | 43.95 | 0.21 | 0.0066 | 0.0373 | 1.54 |
| ADCK5 | ENSG00000173137 | 37.08 | 0.22 | 0.0046 | 0.0352 | 1.84 |
| ADCY6 | ENSG00000174233 | 453.83 | 0.25 | 0.0008 | 0.0342 | 0.92 |
| ADCY9 | ENSG00000162104 | 159.78 | 0.18 | 0.0155 | 0.0492 | 0.84 |
| ADD1 | ENSG00000087274 | 560.90 | 0.20 | 0.0057 | 0.0363 | 1.08 |
| ADD3 | ENSG00000148700 | 325.45 | 0.27 | 0.0003 | 0.0342 | 1.62 |
| ADH1C | ENSG00000248144 | 216.51 | 0.19 | 0.0116 | 0.0439 | 0.13 |
| ADH5 | ENSG00000197894 | 105.89 | 0.19 | 0.0093 | 0.0409 | 1.31 |
| ADH6 | ENSG00000172955 | 31.17 | 0.25 | 0.0010 | 0.0342 | 0.70 |
| ADHFE1 | ENSG00000147576 | 25.14 | 0.19 | 0.0113 | 0.0435 | 0.97 |
| ADI1 | ENSG00000182551 | 97.79 | 0.27 | 0.0004 | 0.0342 | 1.59 |
| ADIPOR1 | ENSG00000159346 | 133.38 | 0.20 | 0.0053 | 0.0361 | 1.92 |
| ADIPOR2 | ENSG00000006831 | 218.39 | 0.18 | 0.0146 | 0.0481 | 1.66 |
| ADNP | ENSG00000101126 | 306.88 | 0.18 | 0.0128 | 0.0456 | 2.63 |
| ADNP2 | ENSG00000101544 | 110.34 | 0.20 | 0.0081 | 0.0391 | 1.21 |
| ADO | ENSG00000181915 | 36.19 | 0.18 | 0.0149 | 0.0485 | 2.20 |
| ADPRHL2 | ENSG00000116863 | 26.15 | 0.24 | 0.0023 | 0.0342 | 1.57 |
| ADSS | ENSG00000035687 | 93.26 | 0.19 | 0.0086 | 0.0399 | 1.81 |
| AEBP2 | ENSG00000139154 | 66.73 | 0.17 | 0.0149 | 0.0485 | 1.71 |
| AES | ENSG00000104964 | 299.19 | 0.19 | 0.0110 | 0.0432 | 1.25 |
| AF011889.5 | ENSG00000241489 | 107.81 | 0.19 | 0.0130 | 0.0458 | 1.19 |
| AFAP1L2 | ENSG00000169129 | 64.27 | 0.21 | 0.0032 | 0.0342 | 1.72 |
| AFF1 | ENSG00000172493 | 701.07 | 0.19 | 0.0085 | 0.0399 | 0.94 |
| AFF2 | ENSG00000155966 | 13.22 | -0.27 | 0.0025 | 0.0342 | 0.73 |
| AFG3L2 | ENSG00000141385 | 166.65 | 0.19 | 0.0092 | 0.0408 | 0.98 |
| AFMID | ENSG00000183077 | 61.01 | 0.23 | 0.0014 | 0.0342 | 1.79 |
| AFTPH | ENSG00000119844 | 193.42 | 0.20 | 0.0059 | 0.0365 | 1.39 |
| AGAP1 | ENSG00000157985 | 218.10 | 0.21 | 0.0053 | 0.0361 | 1.27 |
| AGAP3 | ENSG00000133612 | 115.15 | 0.21 | 0.0060 | 0.0366 | 2.37 |
| AGAP5 | ENSG00000172650 | 22.65 | 0.20 | 0.0083 | 0.0395 | 1.11 |
| AGBL5 | ENSG00000084693 | 108.59 | 0.20 | 0.0066 | 0.0373 | 1.62 |
| AGFG2 | ENSG00000106351 | 189.05 | 0.22 | 0.0040 | 0.0346 | 0.88 |
| AGGF1 | ENSG00000164252 | 79.81 | 0.19 | 0.0109 | 0.0431 | 1.61 |
| AGL | ENSG00000162688 | 110.37 | 0.20 | 0.0060 | 0.0366 | 1.21 |
| AGPAT1 | ENSG00000204310 | 91.61 | 0.21 | 0.0037 | 0.0342 | 1.61 |
| AGPAT3 | ENSG00000160216 | 283.14 | 0.23 | 0.0015 | 0.0342 | 1.36 |
| AGPAT5 | ENSG00000155189 | 58.03 | 0.20 | 0.0104 | 0.0424 | 1.92 |
| AGPS | ENSG00000018510 | 152.72 | 0.19 | 0.0100 | 0.0419 | 1.72 |
| AGRN | ENSG00000188157 | 247.42 | 0.25 | 0.0005 | 0.0342 | 2.49 |
| AGT | ENSG00000135744 | 9.44 | 0.20 | 0.0099 | 0.0417 | 3.33 |
| AGXT2L2 | ENSG00000175309 | 621.50 | 0.23 | 0.0015 | 0.0342 | 0.89 |
| AHCY | ENSG00000101444 | 93.42 | 0.25 | 0.0010 | 0.0342 | 4.52 |
| AHCYL1 | ENSG00000168710 | 219.21 | 0.18 | 0.0149 | 0.0485 | 1.44 |
| AHCYL2 | ENSG00000158467 | 737.88 | 0.21 | 0.0034 | 0.0342 | 0.35 |
| AHDC1 | ENSG00000126705 | 98.06 | 0.19 | 0.0126 | 0.0452 | 1.22 |
| AHRR | ENSG00000063438 | 25.74 | 0.20 | 0.0088 | 0.0402 | 0.79 |
| AHSA2 | ENSG00000173209 | 198.17 | 0.21 | 0.0042 | 0.0350 | 1.35 |
| AIDA | ENSG00000186063 | 42.22 | 0.19 | 0.0118 | 0.0441 | 1.80 |
| AIFM1 | ENSG00000156709 | 50.45 | 0.18 | 0.0113 | 0.0435 | 2.10 |
| AIG1 | ENSG00000146416 | 27.18 | 0.23 | 0.0022 | 0.0342 | 2.22 |
| AIM1 | ENSG00000112297 | 414.07 | 0.19 | 0.0101 | 0.0421 | 1.45 |
| AIMP1 | ENSG00000164022 | 52.69 | 0.19 | 0.0093 | 0.0409 | 1.68 |
| AK2 | ENSG00000004455 | 200.40 | 0.21 | 0.0046 | 0.0352 | 1.39 |
| AK3 | ENSG00000147853 | 147.50 | 0.27 | 0.0006 | 0.0342 | 1.66 |
| AK4 | ENSG00000162433 | 64.47 | 0.21 | 0.0031 | 0.0342 | 2.56 |
| AKAP1 | ENSG00000121057 | 359.84 | 0.24 | 0.0011 | 0.0342 | 1.66 |
| AKAP10 | ENSG00000108599 | 92.53 | 0.18 | 0.0102 | 0.0422 | 1.26 |
| AKAP11 | ENSG00000023516 | 305.29 | 0.19 | 0.0093 | 0.0409 | 2.34 |
| AKAP5 | ENSG00000179841 | 65.71 | 0.23 | 0.0019 | 0.0342 | 0.67 |
| AKAP8 | ENSG00000105127 | 101.45 | 0.18 | 0.0151 | 0.0487 | 1.42 |
| AKAP8L | ENSG00000011243 | 56.13 | 0.20 | 0.0076 | 0.0382 | 1.56 |
| AKIP1 | ENSG00000166452 | 12.98 | 0.19 | 0.0108 | 0.0429 | 2.05 |
| AKR1A1 | ENSG00000117448 | 96.74 | 0.24 | 0.0017 | 0.0342 | 1.63 |
| AKR1C3 | ENSG00000196139 | 38.14 | 0.22 | 0.0030 | 0.0342 | 1.19 |
| AKR7A2 | ENSG00000053371 | 56.10 | 0.26 | 0.0003 | 0.0342 | 1.13 |
| AKR7A3 | ENSG00000162482 | 32.47 | 0.22 | 0.0025 | 0.0342 | 1.02 |
| AKT1 | ENSG00000142208 | 284.31 | 0.18 | 0.0130 | 0.0458 | 1.38 |
| AKT1S1 | ENSG00000204673 | 88.91 | 0.19 | 0.0112 | 0.0434 | 1.49 |
| AKTIP | ENSG00000166971 | 36.82 | 0.21 | 0.0064 | 0.0370 | 1.49 |
| AL034548.2 | ENSG00000247315 | 37.23 | 0.20 | 0.0084 | 0.0397 | 1.77 |
| ALAD | ENSG00000148218 | 107.89 | 0.27 | 0.0002 | 0.0342 | 1.14 |
| ALAS1 | ENSG00000023330 | 69.50 | 0.21 | 0.0040 | 0.0346 | 1.35 |
| ALCAM | ENSG00000170017 | 65.18 | 0.23 | 0.0029 | 0.0342 | 2.71 |
| ALDH18A1 | ENSG00000059573 | 215.28 | 0.24 | 0.0013 | 0.0342 | 1.70 |
| ALDH2 | ENSG00000111275 | 181.47 | 0.22 | 0.0022 | 0.0342 | 1.77 |
| ALDH3A2 | ENSG00000072210 | 147.69 | 0.20 | 0.0035 | 0.0342 | 1.30 |
| ALDH3B1 | ENSG00000006534 | 58.69 | 0.27 | 0.0004 | 0.0342 | 1.45 |
| ALDH4A1 | ENSG00000159423 | 12.44 | 0.23 | 0.0039 | 0.0345 | 5.12 |
| ALDH5A1 | ENSG00000112294 | 60.85 | 0.19 | 0.0073 | 0.0379 | 1.66 |
| ALDH6A1 | ENSG00000119711 | 41.92 | 0.23 | 0.0024 | 0.0342 | 0.95 |
| ALDH7A1 | ENSG00000164904 | 64.38 | 0.20 | 0.0059 | 0.0365 | 1.67 |
| ALDH9A1 | ENSG00000143149 | 66.80 | 0.21 | 0.0045 | 0.0352 | 1.58 |
| ALDOA | ENSG00000149925 | 582.02 | 0.23 | 0.0013 | 0.0342 | 2.22 |
| ALDOC | ENSG00000109107 | 14.00 | 0.19 | 0.0144 | 0.0477 | 3.05 |
| ALG1 | ENSG00000033011 | 26.81 | 0.23 | 0.0022 | 0.0342 | 2.06 |
| ALG11 | ENSG00000253710 | 73.33 | 0.23 | 0.0024 | 0.0342 | 1.76 |
| ALG12 | ENSG00000182858 | 50.47 | 0.19 | 0.0111 | 0.0433 | 1.09 |
| ALG2 | ENSG00000119523 | 56.22 | 0.23 | 0.0030 | 0.0342 | 1.63 |
| ALG9 | ENSG00000086848 | 41.25 | 0.18 | 0.0160 | 0.0499 | 1.56 |
| ALKBH1 | ENSG00000100601 | 17.26 | 0.20 | 0.0050 | 0.0356 | 1.33 |
| ALKBH4 | ENSG00000160993 | 18.05 | 0.22 | 0.0050 | 0.0356 | 1.45 |
| ALKBH5 | ENSG00000091542 | 165.61 | 0.23 | 0.0024 | 0.0342 | 1.27 |
| ALOX12B | ENSG00000179477 | 20.66 | 0.21 | 0.0072 | 0.0377 | 0.25 |
| ALPK1 | ENSG00000073331 | 155.96 | 0.19 | 0.0123 | 0.0448 | 1.13 |
| ALS2 | ENSG00000003393 | 80.93 | 0.25 | 0.0009 | 0.0342 | 1.82 |
| ALS2CL | ENSG00000178038 | 329.61 | 0.22 | 0.0022 | 0.0342 | 1.29 |
| AMACR | ENSG00000242110 | 72.16 | 0.25 | 0.0010 | 0.0342 | 2.80 |
| AMFR | ENSG00000159461 | 139.54 | 0.21 | 0.0033 | 0.0342 | 1.57 |
| AMIGO1 | ENSG00000181754 | 19.64 | 0.18 | 0.0161 | 0.0500 | 1.27 |
| AMIGO3 | ENSG00000176020 | 165.38 | 0.19 | 0.0108 | 0.0429 | 0.93 |
| AMMECR1 | ENSG00000101935 | 66.10 | 0.21 | 0.0074 | 0.0380 | 1.95 |
| AMN | ENSG00000166126 | 226.39 | 0.20 | 0.0064 | 0.0370 | 0.39 |
| AMOT | ENSG00000126016 | 57.31 | 0.32 | 0.0010 | 0.0342 | 3.21 |
| AMPD3 | ENSG00000133805 | 66.33 | 0.18 | 0.0127 | 0.0454 | 1.44 |
| AMT | ENSG00000145020 | 93.69 | 0.19 | 0.0114 | 0.0437 | 1.01 |
| ANAPC1 | ENSG00000153107 | 89.11 | 0.19 | 0.0113 | 0.0435 | 2.48 |
| ANAPC10 | ENSG00000164162 | 14.98 | 0.18 | 0.0151 | 0.0487 | 1.65 |
| ANAPC16 | ENSG00000166295 | 100.14 | 0.19 | 0.0117 | 0.0439 | 1.39 |
| ANAPC4 | ENSG00000053900 | 83.17 | 0.21 | 0.0033 | 0.0342 | 1.37 |
| ANAPC5 | ENSG00000089053 | 387.84 | 0.19 | 0.0115 | 0.0438 | 1.57 |
| ANG | ENSG00000214274 | 32.27 | 0.18 | 0.0151 | 0.0487 | 0.76 |
| ANGEL1 | ENSG00000013523 | 71.48 | 0.20 | 0.0089 | 0.0403 | 1.86 |
| ANGEL2 | ENSG00000174606 | 79.73 | 0.20 | 0.0095 | 0.0413 | 1.64 |
| ANGPTL3 | ENSG00000132855 | 21.51 | 0.19 | 0.0125 | 0.0451 | 1.10 |
| ANGPTL7 | ENSG00000171819 | 16.30 | 0.18 | 0.0148 | 0.0484 | 1.16 |
| ANKAR | ENSG00000151687 | 49.03 | 0.19 | 0.0119 | 0.0442 | 0.84 |
| ANKFY1 | ENSG00000185722 | 239.85 | 0.19 | 0.0069 | 0.0375 | 1.17 |
| ANKHD1 | ENSG00000131503 | 730.14 | 0.18 | 0.0135 | 0.0464 | 1.25 |
| ANKHD1-EIF4EBP3 | ENSG00000254996 | 711.28 | 0.19 | 0.0101 | 0.0421 | 1.24 |
| ANKIB1 | ENSG00000001629 | 157.38 | 0.18 | 0.0132 | 0.0460 | 1.96 |
| ANKMY1 | ENSG00000144504 | 129.78 | 0.20 | 0.0057 | 0.0363 | 1.66 |
| ANKMY2 | ENSG00000106524 | 42.22 | 0.21 | 0.0054 | 0.0362 | 1.31 |
| ANKRA2 | ENSG00000164331 | 26.02 | 0.24 | 0.0003 | 0.0342 | 1.44 |
| ANKRD11 | ENSG00000167522 | 370.72 | 0.19 | 0.0126 | 0.0452 | 1.47 |
| ANKRD13A | ENSG00000076513 | 161.27 | 0.20 | 0.0052 | 0.0359 | 0.96 |
| ANKRD13C | ENSG00000118454 | 82.62 | 0.20 | 0.0042 | 0.0350 | 1.37 |
| ANKRD17 | ENSG00000132466 | 652.48 | 0.20 | 0.0063 | 0.0370 | 1.30 |
| ANKRD18A | ENSG00000180071 | 54.77 | 0.19 | 0.0133 | 0.0462 | 1.21 |
| ANKRD23 | ENSG00000163126 | 48.95 | 0.21 | 0.0058 | 0.0364 | 1.63 |
| ANKRD26 | ENSG00000107890 | 74.75 | 0.18 | 0.0141 | 0.0472 | 1.39 |
| ANKRD27 | ENSG00000105186 | 96.71 | 0.21 | 0.0027 | 0.0342 | 2.11 |
| ANKRD37 | ENSG00000186352 | 20.07 | 0.18 | 0.0140 | 0.0471 | 1.35 |
| ANKRD39 | ENSG00000213337 | 45.72 | 0.22 | 0.0037 | 0.0342 | 1.64 |
| ANKRD43 | ENSG00000198944 | 32.33 | 0.22 | 0.0046 | 0.0352 | 0.94 |
| ANKRD5 | ENSG00000132623 | 20.05 | 0.20 | 0.0097 | 0.0415 | 2.62 |
| ANKRD50 | ENSG00000151458 | 146.09 | 0.23 | 0.0017 | 0.0342 | 1.43 |
| ANKRD52 | ENSG00000139645 | 287.04 | 0.19 | 0.0100 | 0.0419 | 1.56 |
| ANKRD54 | ENSG00000100124 | 33.11 | 0.18 | 0.0117 | 0.0439 | 1.22 |
| ANKRD56 | ENSG00000186212 | 35.87 | 0.25 | 0.0010 | 0.0342 | 1.12 |
| ANKRD61 | ENSG00000157999 | 18.27 | 0.24 | 0.0021 | 0.0342 | 1.67 |
| ANKS1A | ENSG00000064999 | 143.89 | 0.22 | 0.0027 | 0.0342 | 1.65 |
| ANKS3 | ENSG00000168096 | 36.11 | 0.24 | 0.0015 | 0.0342 | 1.65 |
| ANKS4B | ENSG00000175311 | 85.75 | 0.25 | 0.0014 | 0.0342 | 1.23 |
| ANKS6 | ENSG00000165138 | 94.56 | 0.28 | 0.0002 | 0.0342 | 2.40 |
| ANKUB1 | ENSG00000206199 | 13.83 | 0.25 | 0.0014 | 0.0342 | 1.68 |
| ANKZF1 | ENSG00000163516 | 92.63 | 0.24 | 0.0013 | 0.0342 | 1.76 |
| ANO10 | ENSG00000160746 | 108.18 | 0.19 | 0.0078 | 0.0386 | 0.86 |
| ANO5 | ENSG00000171714 | 43.07 | 0.24 | 0.0011 | 0.0342 | 0.31 |
| ANO6 | ENSG00000177119 | 104.57 | 0.22 | 0.0052 | 0.0359 | 2.85 |
| ANO7 | ENSG00000146205 | 307.41 | 0.21 | 0.0075 | 0.0382 | 0.36 |
| ANO9 | ENSG00000185101 | 305.66 | 0.25 | 0.0031 | 0.0342 | 2.65 |
| ANP32B | ENSG00000136938 | 85.68 | 0.21 | 0.0051 | 0.0358 | 2.26 |
| ANTXR2 | ENSG00000163297 | 201.09 | 0.23 | 0.0015 | 0.0342 | 0.96 |
| ANXA3 | ENSG00000138772 | 35.52 | 0.21 | 0.0060 | 0.0366 | 3.67 |
| ANXA4 | ENSG00000196975 | 248.77 | 0.26 | 0.0007 | 0.0342 | 2.00 |
| ANXA9 | ENSG00000143412 | 20.74 | 0.22 | 0.0035 | 0.0342 | 1.29 |
| AP000295.9 | ENSG00000249624 | 48.48 | 0.18 | 0.0146 | 0.0481 | 1.08 |
| AP000304.12 | ENSG00000249209 | 44.32 | 0.26 | 0.0002 | 0.0342 | 1.52 |
| AP000679.2 | ENSG00000176984 | 5.60 | 0.23 | 0.0049 | 0.0355 | 0.69 |
| AP003068.23 | ENSG00000254614 | 10.49 | 0.26 | 0.0008 | 0.0342 | 1.74 |
| AP003068.6 | ENSG00000187066 | 51.16 | 0.21 | 0.0056 | 0.0363 | 1.11 |
| AP003419.11 | ENSG00000256514 | 89.19 | 0.20 | 0.0089 | 0.0403 | 0.81 |
| AP006621.5 | ENSG00000255284 | 8.06 | 0.21 | 0.0084 | 0.0397 | 1.93 |
| AP1G1 | ENSG00000166747 | 374.52 | 0.21 | 0.0045 | 0.0352 | 1.28 |
| AP1G2 | ENSG00000213983 | 242.97 | 0.21 | 0.0040 | 0.0346 | 1.17 |
| AP1M2 | ENSG00000129354 | 67.66 | 0.22 | 0.0024 | 0.0342 | 1.70 |
| AP1S1 | ENSG00000106367 | 38.37 | 0.25 | 0.0017 | 0.0342 | 3.05 |
| AP2A1 | ENSG00000196961 | 122.59 | 0.19 | 0.0118 | 0.0441 | 1.75 |
| AP2B1 | ENSG00000006125 | 481.86 | 0.22 | 0.0027 | 0.0342 | 1.61 |
| AP2M1 | ENSG00000161203 | 169.47 | 0.20 | 0.0057 | 0.0363 | 1.59 |
| AP3B1 | ENSG00000132842 | 163.63 | 0.19 | 0.0081 | 0.0391 | 1.27 |
| AP3D1 | ENSG00000065000 | 305.95 | 0.21 | 0.0038 | 0.0342 | 1.80 |
| AP3M1 | ENSG00000185009 | 90.19 | 0.19 | 0.0091 | 0.0407 | 1.80 |
| AP3S1 | ENSG00000177879 | 52.07 | 0.19 | 0.0103 | 0.0423 | 2.09 |
| AP4B1 | ENSG00000134262 | 82.38 | 0.18 | 0.0117 | 0.0439 | 1.08 |
| AP4M1 | ENSG00000221838 | 57.80 | 0.20 | 0.0079 | 0.0387 | 1.96 |
| APAF1 | ENSG00000120868 | 159.77 | 0.23 | 0.0023 | 0.0342 | 1.29 |
| APBA1 | ENSG00000107282 | 31.55 | 0.20 | 0.0074 | 0.0380 | 0.66 |
| APBA3 | ENSG00000011132 | 29.05 | 0.21 | 0.0077 | 0.0384 | 1.39 |
| APEH | ENSG00000164062 | 113.06 | 0.19 | 0.0096 | 0.0414 | 1.85 |
| APEX1 | ENSG00000100823 | 80.06 | 0.23 | 0.0021 | 0.0342 | 2.18 |
| APEX2 | ENSG00000169188 | 39.67 | 0.21 | 0.0042 | 0.0350 | 1.90 |
| APH1A | ENSG00000117362 | 196.23 | 0.19 | 0.0101 | 0.0421 | 1.45 |
| API5 | ENSG00000166181 | 115.49 | 0.23 | 0.0020 | 0.0342 | 2.05 |
| APLP2 | ENSG00000084234 | 518.94 | 0.20 | 0.0071 | 0.0376 | 1.27 |
| APOBEC3C | ENSG00000244509 | 23.69 | 0.20 | 0.0111 | 0.0433 | 1.13 |
| APOBEC3F | ENSG00000128394 | 17.67 | 0.24 | 0.0026 | 0.0342 | 0.98 |
| APOBR | ENSG00000184730 | 253.00 | 0.21 | 0.0054 | 0.0362 | 0.51 |
| APOC1 | ENSG00000130208 | 5.75 | 0.28 | 0.0018 | 0.0342 | 3.66 |
| APOL2 | ENSG00000128335 | 76.06 | 0.26 | 0.0015 | 0.0342 | 1.39 |
| APOL6 | ENSG00000221963 | 125.26 | 0.21 | 0.0064 | 0.0370 | 1.37 |
| APOO | ENSG00000184831 | 9.27 | 0.19 | 0.0143 | 0.0476 | 1.88 |
| APOOL | ENSG00000155008 | 32.18 | 0.20 | 0.0078 | 0.0386 | 1.73 |
| APP | ENSG00000142192 | 782.81 | 0.23 | 0.0011 | 0.0342 | 1.53 |
| APPBP2 | ENSG00000062725 | 97.37 | 0.20 | 0.0077 | 0.0384 | 1.44 |
| APPL2 | ENSG00000136044 | 201.01 | 0.22 | 0.0026 | 0.0342 | 0.83 |
| APRT | ENSG00000198931 | 47.75 | 0.21 | 0.0042 | 0.0350 | 2.08 |
| AQR | ENSG00000021776 | 120.38 | 0.19 | 0.0083 | 0.0395 | 1.50 |
| ARCN1 | ENSG00000095139 | 293.24 | 0.21 | 0.0045 | 0.0352 | 1.56 |
| ARF1 | ENSG00000143761 | 399.73 | 0.21 | 0.0038 | 0.0342 | 1.53 |
| ARF4 | ENSG00000168374 | 226.97 | 0.22 | 0.0038 | 0.0342 | 1.24 |
| ARFGAP1 | ENSG00000101199 | 240.23 | 0.21 | 0.0059 | 0.0365 | 2.00 |
| ARFGAP2 | ENSG00000149182 | 161.50 | 0.21 | 0.0040 | 0.0346 | 1.46 |
| ARFGEF1 | ENSG00000066777 | 534.97 | 0.19 | 0.0088 | 0.0402 | 1.56 |
| ARFGEF2 | ENSG00000124198 | 450.78 | 0.22 | 0.0021 | 0.0342 | 2.04 |
| ARFIP1 | ENSG00000164144 | 87.53 | 0.20 | 0.0069 | 0.0375 | 1.39 |
| ARFRP1 | ENSG00000101246 | 121.07 | 0.19 | 0.0130 | 0.0458 | 2.08 |
| ARG2 | ENSG00000081181 | 47.78 | 0.19 | 0.0083 | 0.0395 | 1.30 |
| ARHGAP1 | ENSG00000175220 | 231.08 | 0.20 | 0.0091 | 0.0407 | 1.50 |
| ARHGAP12 | ENSG00000165322 | 192.15 | 0.24 | 0.0006 | 0.0342 | 1.54 |
| ARHGAP18 | ENSG00000146376 | 91.80 | 0.20 | 0.0056 | 0.0363 | 2.33 |
| ARHGAP21 | ENSG00000107863 | 311.06 | 0.18 | 0.0141 | 0.0472 | 1.29 |
| ARHGAP26 | ENSG00000145819 | 449.74 | 0.19 | 0.0076 | 0.0382 | 1.17 |
| ARHGAP27 | ENSG00000159314 | 381.63 | 0.20 | 0.0088 | 0.0402 | 1.17 |
| ARHGAP32 | ENSG00000134909 | 526.56 | 0.29 | 0.0026 | 0.0342 | 1.49 |
| ARHGAP35 | ENSG00000160007 | 369.24 | 0.21 | 0.0046 | 0.0352 | 1.29 |
| ARHGAP44 | ENSG00000006740 | 162.55 | 0.18 | 0.0126 | 0.0452 | 0.35 |
| ARHGAP5 | ENSG00000100852 | 369.56 | 0.22 | 0.0032 | 0.0342 | 1.75 |
| ARHGAP8 | ENSG00000241484 | 143.66 | 0.21 | 0.0075 | 0.0382 | 1.53 |
| ARHGEF10L | ENSG00000074964 | 432.43 | 0.25 | 0.0009 | 0.0342 | 1.13 |
| ARHGEF11 | ENSG00000132694 | 308.94 | 0.23 | 0.0020 | 0.0342 | 1.27 |
| ARHGEF12 | ENSG00000196914 | 760.99 | 0.22 | 0.0013 | 0.0342 | 1.48 |
| ARHGEF16 | ENSG00000130762 | 175.89 | 0.23 | 0.0023 | 0.0342 | 0.92 |
| ARHGEF17 | ENSG00000110237 | 151.07 | 0.19 | 0.0150 | 0.0486 | 1.70 |
| ARHGEF2 | ENSG00000116584 | 437.88 | 0.21 | 0.0047 | 0.0353 | 0.98 |
| ARHGEF37 | ENSG00000183111 | 58.20 | 0.24 | 0.0014 | 0.0342 | 1.02 |
| ARHGEF38 | ENSG00000236699 | 134.77 | 0.23 | 0.0024 | 0.0342 | 1.15 |
| ARHGEF40 | ENSG00000165801 | 195.85 | 0.19 | 0.0111 | 0.0433 | 1.23 |
| ARHGEF7 | ENSG00000102606 | 279.61 | 0.18 | 0.0130 | 0.0458 | 1.55 |
| ARID1A | ENSG00000117713 | 445.25 | 0.21 | 0.0048 | 0.0354 | 1.28 |
| ARID4A | ENSG00000032219 | 111.30 | 0.18 | 0.0108 | 0.0429 | 1.21 |
| ARID4B | ENSG00000054267 | 212.07 | 0.18 | 0.0128 | 0.0456 | 1.58 |
| ARL1 | ENSG00000120805 | 97.97 | 0.24 | 0.0010 | 0.0342 | 1.63 |
| ARL15 | ENSG00000185305 | 42.88 | 0.18 | 0.0145 | 0.0479 | 1.45 |
| ARL2 | ENSG00000213465 | 30.18 | 0.20 | 0.0053 | 0.0361 | 1.72 |
| ARL2BP | ENSG00000102931 | 55.73 | 0.19 | 0.0117 | 0.0439 | 1.51 |
| ARL6IP1 | ENSG00000170540 | 163.74 | 0.20 | 0.0091 | 0.0407 | 2.48 |
| ARL6IP5 | ENSG00000144746 | 85.11 | 0.20 | 0.0095 | 0.0413 | 1.61 |
| ARL6IP6 | ENSG00000177917 | 22.91 | 0.21 | 0.0086 | 0.0399 | 1.84 |
| ARL8B | ENSG00000134108 | 97.69 | 0.19 | 0.0104 | 0.0424 | 1.59 |
| ARMC10 | ENSG00000170632 | 54.63 | 0.22 | 0.0032 | 0.0342 | 2.14 |
| ARMCX5 | ENSG00000125962 | 52.97 | 0.22 | 0.0038 | 0.0342 | 1.71 |
| ARNT | ENSG00000143437 | 186.49 | 0.19 | 0.0058 | 0.0364 | 1.15 |
| ARPC3 | ENSG00000111229 | 76.58 | 0.19 | 0.0115 | 0.0438 | 1.74 |
| ARPC4 | ENSG00000241553 | 211.90 | 0.18 | 0.0153 | 0.0489 | 1.41 |
| ARPC4-TTLL3 | ENSG00000250151 | 139.45 | 0.19 | 0.0123 | 0.0448 | 1.34 |
| ARPC5 | ENSG00000162704 | 94.21 | 0.20 | 0.0058 | 0.0364 | 1.92 |
| ARRB1 | ENSG00000137486 | 157.55 | 0.24 | 0.0008 | 0.0342 | 0.85 |
| ARRDC1 | ENSG00000197070 | 153.14 | 0.20 | 0.0092 | 0.0408 | 1.61 |
| ARSA | ENSG00000100299 | 74.73 | 0.23 | 0.0038 | 0.0342 | 0.88 |
| ARSD | ENSG00000006756 | 240.31 | 0.26 | 0.0003 | 0.0342 | 1.33 |
| ARSE | ENSG00000157399 | 28.27 | 0.23 | 0.0029 | 0.0342 | 2.78 |
| ARV1 | ENSG00000173409 | 26.57 | 0.23 | 0.0015 | 0.0342 | 1.25 |
| ASAH1 | ENSG00000104763 | 157.21 | 0.24 | 0.0008 | 0.0342 | 1.22 |
| ASAP2 | ENSG00000151693 | 263.74 | 0.22 | 0.0025 | 0.0342 | 1.19 |
| ASAP3 | ENSG00000088280 | 91.14 | 0.18 | 0.0138 | 0.0468 | 0.64 |
| ASB13 | ENSG00000196372 | 44.57 | 0.21 | 0.0072 | 0.0377 | 1.63 |
| ASB16 | ENSG00000161664 | 30.46 | 0.22 | 0.0048 | 0.0354 | 1.18 |
| ASB6 | ENSG00000148331 | 33.94 | 0.20 | 0.0055 | 0.0363 | 1.68 |
| ASCC1 | ENSG00000138303 | 46.78 | 0.18 | 0.0141 | 0.0472 | 1.52 |
| ASCC2 | ENSG00000100325 | 147.69 | 0.19 | 0.0114 | 0.0437 | 1.04 |
| ASCC3 | ENSG00000112249 | 228.66 | 0.20 | 0.0071 | 0.0376 | 1.81 |
| ASCL2 | ENSG00000183734 | 16.01 | 0.24 | 0.0040 | 0.0346 | 13.99 |
| ASF1A | ENSG00000111875 | 36.82 | 0.19 | 0.0142 | 0.0474 | 1.45 |
| ASH2L | ENSG00000129691 | 78.70 | 0.17 | 0.0149 | 0.0485 | 1.27 |
| ASNA1 | ENSG00000198356 | 23.51 | 0.18 | 0.0148 | 0.0484 | 1.88 |
| ASPG | ENSG00000166183 | 45.14 | 0.21 | 0.0080 | 0.0389 | 0.25 |
| ASPH | ENSG00000198363 | 321.11 | 0.26 | 0.0012 | 0.0342 | 2.44 |
| ASPHD2 | ENSG00000128203 | 24.49 | 0.20 | 0.0088 | 0.0402 | 1.09 |
| ASPSCR1 | ENSG00000169696 | 34.78 | 0.20 | 0.0087 | 0.0401 | 1.99 |
| ASXL2 | ENSG00000143970 | 256.53 | 0.18 | 0.0115 | 0.0438 | 1.75 |
| ATAD2 | ENSG00000156802 | 61.03 | 0.20 | 0.0109 | 0.0431 | 3.71 |
| ATAT1 | ENSG00000137343 | 60.70 | 0.21 | 0.0050 | 0.0356 | 1.58 |
| ATE1 | ENSG00000107669 | 112.52 | 0.22 | 0.0021 | 0.0342 | 1.53 |
| ATF1 | ENSG00000123268 | 33.61 | 0.25 | 0.0009 | 0.0342 | 1.86 |
| ATF6B | ENSG00000213676 | 178.36 | 0.21 | 0.0044 | 0.0351 | 1.38 |
| ATF7 | ENSG00000170653 | 250.07 | 0.17 | 0.0147 | 0.0482 | 1.31 |
| ATG10 | ENSG00000152348 | 65.93 | 0.18 | 0.0118 | 0.0441 | 1.85 |
| ATG13 | ENSG00000175224 | 142.98 | 0.21 | 0.0049 | 0.0355 | 1.38 |
| ATG14 | ENSG00000126775 | 118.70 | 0.19 | 0.0089 | 0.0403 | 1.01 |
| ATG3 | ENSG00000144848 | 61.24 | 0.19 | 0.0150 | 0.0486 | 1.87 |
| ATG4A | ENSG00000101844 | 28.98 | 0.18 | 0.0127 | 0.0454 | 0.95 |
| ATG4B | ENSG00000168397 | 151.92 | 0.21 | 0.0057 | 0.0363 | 1.13 |
| ATG4D | ENSG00000130734 | 67.90 | 0.19 | 0.0139 | 0.0469 | 0.94 |
| ATG5 | ENSG00000057663 | 60.89 | 0.20 | 0.0062 | 0.0368 | 1.54 |
| ATG7 | ENSG00000197548 | 141.93 | 0.17 | 0.0131 | 0.0460 | 1.47 |
| ATG9A | ENSG00000198925 | 127.23 | 0.22 | 0.0035 | 0.0342 | 1.59 |
| ATIC | ENSG00000138363 | 65.18 | 0.24 | 0.0008 | 0.0342 | 3.23 |
| ATL1 | ENSG00000198513 | 14.33 | 0.19 | 0.0107 | 0.0428 | 1.16 |
| ATL2 | ENSG00000119787 | 92.60 | 0.21 | 0.0048 | 0.0354 | 1.68 |
| ATL3 | ENSG00000184743 | 198.39 | 0.24 | 0.0014 | 0.0342 | 1.55 |
| ATMIN | ENSG00000166454 | 102.10 | 0.23 | 0.0029 | 0.0342 | 1.51 |
| ATOH1 | ENSG00000172238 | 28.09 | 0.21 | 0.0081 | 0.0391 | 0.45 |
| ATP10B | ENSG00000118322 | 940.49 | 0.21 | 0.0038 | 0.0342 | 1.14 |
| ATP11A | ENSG00000068650 | 110.01 | 0.19 | 0.0134 | 0.0463 | 4.87 |
| ATP11B | ENSG00000058063 | 386.29 | 0.24 | 0.0012 | 0.0342 | 1.25 |
| ATP13A2 | ENSG00000159363 | 101.74 | 0.25 | 0.0008 | 0.0342 | 1.69 |
| ATP13A4 | ENSG00000127249 | 63.74 | 0.20 | 0.0151 | 0.0487 | 0.25 |
| ATP1A1 | ENSG00000163399 | 1503.78 | 0.25 | 0.0007 | 0.0342 | 1.32 |
| ATP1B1 | ENSG00000143153 | 489.52 | 0.22 | 0.0039 | 0.0345 | 2.01 |
| ATP2A3 | ENSG00000074370 | 723.35 | 0.19 | 0.0104 | 0.0424 | 0.37 |
| ATP2C1 | ENSG00000017260 | 210.54 | 0.22 | 0.0022 | 0.0342 | 1.91 |
| ATP2C2 | ENSG00000064270 | 483.25 | 0.21 | 0.0046 | 0.0352 | 0.67 |
| ATP5A1 | ENSG00000152234 | 327.11 | 0.17 | 0.0160 | 0.0499 | 1.06 |
| ATP5B | ENSG00000110955 | 592.61 | 0.17 | 0.0145 | 0.0479 | 1.35 |
| ATP5C1 | ENSG00000165629 | 112.35 | 0.20 | 0.0063 | 0.0370 | 1.50 |
| ATP5D | ENSG00000099624 | 66.54 | 0.19 | 0.0090 | 0.0405 | 1.24 |
| ATP5F1 | ENSG00000116459 | 105.50 | 0.19 | 0.0083 | 0.0395 | 1.35 |
| ATP5G1 | ENSG00000159199 | 50.45 | 0.20 | 0.0081 | 0.0391 | 1.31 |
| ATP5G2 | ENSG00000135390 | 112.35 | 0.19 | 0.0110 | 0.0432 | 1.72 |
| ATP5H | ENSG00000167863 | 51.34 | 0.19 | 0.0095 | 0.0413 | 1.77 |
| ATP5I | ENSG00000169020 | 37.99 | 0.21 | 0.0036 | 0.0342 | 1.51 |
| ATP5J | ENSG00000154723 | 37.76 | 0.18 | 0.0111 | 0.0433 | 1.49 |
| ATP5J2-PTCD1 | ENSG00000248919 | 30.02 | 0.19 | 0.0098 | 0.0416 | 2.39 |
| ATP5L | ENSG00000167283 | 70.84 | 0.18 | 0.0099 | 0.0417 | 1.47 |
| ATP5O | ENSG00000241837 | 92.66 | 0.24 | 0.0015 | 0.0342 | 1.39 |
| ATP5SL | ENSG00000105341 | 34.62 | 0.22 | 0.0018 | 0.0342 | 1.54 |
| ATP6AP1 | ENSG00000071553 | 115.82 | 0.22 | 0.0030 | 0.0342 | 1.71 |
| ATP6V0A1 | ENSG00000033627 | 180.05 | 0.22 | 0.0029 | 0.0342 | 1.17 |
| ATP6V0B | ENSG00000117410 | 64.17 | 0.18 | 0.0101 | 0.0421 | 1.45 |
| ATP6V0C | ENSG00000185883 | 87.15 | 0.18 | 0.0126 | 0.0452 | 1.55 |
| ATP6V0E1 | ENSG00000113732 | 64.75 | 0.21 | 0.0035 | 0.0342 | 1.86 |
| ATP6V1A | ENSG00000114573 | 107.80 | 0.18 | 0.0111 | 0.0433 | 1.71 |
| ATP6V1C2 | ENSG00000143882 | 96.33 | 0.21 | 0.0059 | 0.0365 | 2.52 |
| ATP6V1E1 | ENSG00000131100 | 54.18 | 0.23 | 0.0015 | 0.0342 | 1.53 |
| ATP6V1G1 | ENSG00000136888 | 41.90 | 0.23 | 0.0024 | 0.0342 | 2.13 |
| ATP7A | ENSG00000165240 | 76.85 | 0.20 | 0.0079 | 0.0387 | 1.44 |
| ATP7B | ENSG00000123191 | 92.61 | 0.22 | 0.0031 | 0.0342 | 2.28 |
| ATP8A1 | ENSG00000124406 | 419.83 | 0.21 | 0.0055 | 0.0363 | 0.65 |
| ATP8B1 | ENSG00000081923 | 909.73 | 0.24 | 0.0010 | 0.0342 | 0.58 |
| ATP9A | ENSG00000054793 | 417.82 | 0.26 | 0.0003 | 0.0342 | 3.38 |
| ATPIF1 | ENSG00000130770 | 99.42 | 0.21 | 0.0060 | 0.0366 | 1.00 |
| ATRIP | ENSG00000164053 | 21.70 | 0.22 | 0.0037 | 0.0342 | 1.76 |
| ATXN1 | ENSG00000124788 | 354.97 | 0.20 | 0.0062 | 0.0368 | 1.27 |
| ATXN7L3 | ENSG00000087152 | 137.01 | 0.21 | 0.0056 | 0.0363 | 1.61 |
| ATXN7L3B | ENSG00000253719 | 184.85 | 0.21 | 0.0053 | 0.0361 | 1.75 |
| AUH | ENSG00000148090 | 38.40 | 0.22 | 0.0024 | 0.0342 | 1.41 |
| AUP1 | ENSG00000115307 | 167.20 | 0.21 | 0.0042 | 0.0350 | 1.61 |
| AURKA | ENSG00000087586 | 20.83 | 0.19 | 0.0124 | 0.0450 | 4.45 |
| AUTS2 | ENSG00000158321 | 221.01 | 0.21 | 0.0039 | 0.0345 | 1.99 |
| AVEN | ENSG00000169857 | 17.81 | 0.21 | 0.0050 | 0.0356 | 1.87 |
| AVL9 | ENSG00000105778 | 392.47 | 0.20 | 0.0053 | 0.0361 | 1.98 |
| AXIN2 | ENSG00000168646 | 128.90 | 0.26 | 0.0005 | 0.0342 | 7.44 |
| AZIN1 | ENSG00000155096 | 201.73 | 0.23 | 0.0030 | 0.0342 | 2.63 |
| B2M | ENSG00000166710 | 1332.39 | 0.23 | 0.0016 | 0.0342 | 1.30 |
| B3GALT4 | ENSG00000235863 | 28.30 | 0.21 | 0.0068 | 0.0374 | 0.67 |
| B3GALT5 | ENSG00000183778 | 346.83 | 0.29 | 0.0003 | 0.0342 | 0.14 |
| B3GALT6 | ENSG00000176022 | 23.93 | 0.24 | 0.0018 | 0.0342 | 2.42 |
| B3GAT3 | ENSG00000149541 | 25.93 | 0.19 | 0.0133 | 0.0462 | 1.56 |
| B3GNT1 | ENSG00000174684 | 13.98 | 0.18 | 0.0149 | 0.0485 | 1.26 |
| B3GNT2 | ENSG00000170340 | 48.39 | 0.22 | 0.0041 | 0.0349 | 1.72 |
| B3GNT3 | ENSG00000179913 | 272.55 | 0.17 | 0.0157 | 0.0496 | 1.16 |
| B3GNT4 | ENSG00000176383 | 38.04 | 0.19 | 0.0133 | 0.0462 | 1.65 |
| B3GNT7 | ENSG00000156966 | 1264.30 | 0.18 | 0.0160 | 0.0499 | 0.04 |
| B3GNT8 | ENSG00000177191 | 36.55 | 0.21 | 0.0135 | 0.0464 | 0.88 |
| B4GALT4 | ENSG00000121578 | 129.18 | 0.22 | 0.0027 | 0.0342 | 0.85 |
| B4GALT5 | ENSG00000158470 | 184.55 | 0.18 | 0.0145 | 0.0479 | 2.07 |
| BABAM1 | ENSG00000105393 | 61.44 | 0.18 | 0.0155 | 0.0492 | 1.58 |
| BACE2 | ENSG00000182240 | 131.55 | 0.20 | 0.0085 | 0.0399 | 2.39 |
| BACH2 | ENSG00000112182 | 31.74 | -0.22 | 0.0062 | 0.0368 | 0.56 |
| BAD | ENSG00000002330 | 36.33 | 0.21 | 0.0057 | 0.0363 | 0.98 |
| BAG6 | ENSG00000204463 | 316.41 | 0.21 | 0.0046 | 0.0352 | 1.56 |
| BAHCC1 | ENSG00000171282 | 125.84 | 0.23 | 0.0028 | 0.0342 | 1.04 |
| BAIAP2L2 | ENSG00000128298 | 153.73 | 0.20 | 0.0099 | 0.0417 | 0.85 |
| BANF1 | ENSG00000175334 | 28.16 | 0.19 | 0.0102 | 0.0422 | 1.95 |
| BAP1 | ENSG00000163930 | 147.83 | 0.18 | 0.0123 | 0.0448 | 1.37 |
| BATF2 | ENSG00000168062 | 32.09 | 0.24 | 0.0069 | 0.0375 | 2.08 |
| BAZ1B | ENSG00000009954 | 260.51 | 0.20 | 0.0054 | 0.0362 | 2.29 |
| BAZ2A | ENSG00000076108 | 564.93 | 0.20 | 0.0054 | 0.0362 | 1.33 |
| BBC3 | ENSG00000105327 | 23.20 | 0.19 | 0.0160 | 0.0499 | 2.41 |
| BBIP1 | ENSG00000214413 | 166.84 | 0.19 | 0.0134 | 0.0463 | 0.51 |
| BBS1 | ENSG00000174483 | 81.03 | 0.25 | 0.0007 | 0.0342 | 1.29 |
| BBS10 | ENSG00000179941 | 13.65 | 0.19 | 0.0094 | 0.0411 | 2.62 |
| BBS12 | ENSG00000181004 | 11.86 | 0.20 | 0.0067 | 0.0373 | 1.06 |
| BBS2 | ENSG00000125124 | 44.80 | 0.23 | 0.0017 | 0.0342 | 1.63 |
| BBS4 | ENSG00000140463 | 29.46 | 0.19 | 0.0098 | 0.0416 | 1.18 |
| BBX | ENSG00000114439 | 182.61 | 0.18 | 0.0138 | 0.0468 | 1.69 |
| BCAP29 | ENSG00000075790 | 95.58 | 0.20 | 0.0073 | 0.0379 | 1.11 |
| BCAP31 | ENSG00000185825 | 74.38 | 0.22 | 0.0022 | 0.0342 | 2.74 |
| BCAR3 | ENSG00000137936 | 57.81 | 0.23 | 0.0025 | 0.0342 | 0.95 |
| BCAS1 | ENSG00000064787 | 502.38 | 0.25 | 0.0016 | 0.0342 | 0.24 |
| BCCIP | ENSG00000107949 | 59.29 | 0.19 | 0.0113 | 0.0435 | 1.77 |
| BCKDHA | ENSG00000248098 | 39.73 | 0.19 | 0.0104 | 0.0424 | 1.37 |
| BCKDHB | ENSG00000083123 | 67.26 | 0.21 | 0.0054 | 0.0362 | 1.31 |
| BCKDK | ENSG00000103507 | 39.72 | 0.21 | 0.0047 | 0.0353 | 1.73 |
| BCL10 | ENSG00000142867 | 111.40 | 0.17 | 0.0151 | 0.0487 | 0.93 |
| BCL2L1 | ENSG00000171552 | 112.67 | 0.24 | 0.0022 | 0.0342 | 3.40 |
| BCL2L13 | ENSG00000099968 | 123.46 | 0.19 | 0.0083 | 0.0395 | 1.28 |
| BCL2L14 | ENSG00000121380 | 100.10 | 0.28 | 0.0003 | 0.0342 | 0.99 |
| BCL2L15 | ENSG00000188761 | 177.23 | 0.25 | 0.0008 | 0.0342 | 0.73 |
| BCL2L2 | ENSG00000129473 | 147.61 | 0.24 | 0.0010 | 0.0342 | 1.16 |
| BCL9 | ENSG00000116128 | 102.55 | 0.22 | 0.0032 | 0.0342 | 1.79 |
| BCMO1 | ENSG00000135697 | 10.36 | 0.19 | 0.0126 | 0.0452 | 0.93 |
| BCOR | ENSG00000183337 | 134.99 | 0.22 | 0.0041 | 0.0349 | 1.76 |
| BCORL1 | ENSG00000085185 | 64.75 | 0.20 | 0.0085 | 0.0399 | 1.73 |
| BCR | ENSG00000186716 | 184.15 | 0.18 | 0.0125 | 0.0451 | 1.47 |
| BDH1 | ENSG00000161267 | 231.81 | 0.24 | 0.0012 | 0.0342 | 1.00 |
| BECN1 | ENSG00000126581 | 134.99 | 0.22 | 0.0027 | 0.0342 | 1.65 |
| BEND7 | ENSG00000165626 | 81.58 | 0.19 | 0.0109 | 0.0431 | 1.55 |
| BEST1 | ENSG00000167995 | 587.34 | 0.19 | 0.0089 | 0.0403 | 1.33 |
| BET1 | ENSG00000105829 | 27.96 | 0.20 | 0.0061 | 0.0368 | 1.39 |
| BET1L | ENSG00000177951 | 128.21 | 0.21 | 0.0061 | 0.0368 | 1.37 |
| BFAR | ENSG00000103429 | 86.65 | 0.25 | 0.0021 | 0.0342 | 1.61 |
| BHLHB9 | ENSG00000198908 | 15.92 | 0.19 | 0.0101 | 0.0421 | 2.01 |
| BHLHE41 | ENSG00000123095 | 99.00 | 0.21 | 0.0058 | 0.0364 | 0.74 |
| BIRC5 | ENSG00000089685 | 19.39 | 0.20 | 0.0119 | 0.0442 | 4.48 |
| BIRC6 | ENSG00000115760 | 1220.31 | 0.21 | 0.0037 | 0.0342 | 1.39 |
| BIVM | ENSG00000134897 | 67.22 | 0.18 | 0.0129 | 0.0457 | 2.12 |
| BLCAP | ENSG00000166619 | 221.61 | 0.26 | 0.0004 | 0.0342 | 1.68 |
| BLOC1S1 | ENSG00000135441 | 42.10 | 0.20 | 0.0067 | 0.0373 | 1.12 |
| BMI1 | ENSG00000168283 | 59.58 | 0.18 | 0.0157 | 0.0496 | 2.01 |
| BMP1 | ENSG00000168487 | 98.98 | 0.20 | 0.0141 | 0.0472 | 1.46 |
| BMP4 | ENSG00000125378 | 42.25 | 0.26 | 0.0011 | 0.0342 | 3.13 |
| BMP8B | ENSG00000116985 | 42.18 | 0.20 | 0.0062 | 0.0368 | 1.43 |
| BMPR1A | ENSG00000107779 | 39.94 | 0.18 | 0.0141 | 0.0472 | 1.47 |
| BMPR2 | ENSG00000204217 | 290.76 | 0.21 | 0.0043 | 0.0351 | 1.82 |
| BNIP3L | ENSG00000104765 | 81.75 | 0.22 | 0.0035 | 0.0342 | 1.80 |
| BOD1 | ENSG00000145919 | 42.97 | 0.23 | 0.0028 | 0.0342 | 2.42 |
| BOK | ENSG00000176720 | 34.58 | 0.21 | 0.0067 | 0.0373 | 2.25 |
| BORA | ENSG00000136122 | 27.25 | 0.22 | 0.0058 | 0.0364 | 2.71 |
| BPHL | ENSG00000137274 | 32.32 | 0.19 | 0.0117 | 0.0439 | 2.07 |
| BPNT1 | ENSG00000162813 | 58.27 | 0.20 | 0.0053 | 0.0361 | 1.13 |
| BRAP | ENSG00000089234 | 54.73 | 0.23 | 0.0021 | 0.0342 | 1.66 |
| BRCC3 | ENSG00000185515 | 53.79 | 0.22 | 0.0041 | 0.0349 | 2.25 |
| BRD3 | ENSG00000169925 | 61.85 | 0.23 | 0.0026 | 0.0342 | 1.93 |
| BRD8 | ENSG00000112983 | 104.65 | 0.19 | 0.0085 | 0.0399 | 1.60 |
| BRF2 | ENSG00000104221 | 35.57 | 0.21 | 0.0052 | 0.0359 | 1.83 |
| BRI3BP | ENSG00000184992 | 211.42 | 0.22 | 0.0023 | 0.0342 | 1.68 |
| BRMS1 | ENSG00000174744 | 38.86 | 0.19 | 0.0136 | 0.0466 | 1.60 |
| BRMS1L | ENSG00000100916 | 11.62 | 0.19 | 0.0091 | 0.0407 | 1.44 |
| BROX | ENSG00000162819 | 126.76 | 0.26 | 0.0005 | 0.0342 | 1.98 |
| BRPF3 | ENSG00000096070 | 227.15 | 0.23 | 0.0007 | 0.0342 | 1.04 |
| BRWD1 | ENSG00000185658 | 562.35 | 0.18 | 0.0124 | 0.0450 | 1.30 |
| BRWD3 | ENSG00000165288 | 216.11 | 0.18 | 0.0106 | 0.0427 | 1.79 |
| BSCL2 | ENSG00000168000 | 64.99 | 0.21 | 0.0050 | 0.0356 | 1.56 |
| BSDC1 | ENSG00000160058 | 230.14 | 0.22 | 0.0023 | 0.0342 | 1.11 |
| BSPRY | ENSG00000119411 | 45.83 | 0.31 | 0.0005 | 0.0342 | 1.93 |
| BTBD1 | ENSG00000064726 | 97.54 | 0.20 | 0.0078 | 0.0386 | 1.80 |
| BTBD2 | ENSG00000133243 | 126.63 | 0.21 | 0.0057 | 0.0363 | 1.71 |
| BTBD6 | ENSG00000184887 | 84.62 | 0.19 | 0.0102 | 0.0422 | 1.16 |
| BTBD7 | ENSG00000011114 | 192.99 | 0.20 | 0.0041 | 0.0349 | 1.14 |
| BTBD8 | ENSG00000189195 | 5.59 | 0.20 | 0.0098 | 0.0416 | 1.65 |
| BTC | ENSG00000174808 | 15.73 | 0.24 | 0.0025 | 0.0342 | 1.22 |
| BTD | ENSG00000169814 | 25.78 | 0.24 | 0.0027 | 0.0342 | 0.89 |
| BTF3 | ENSG00000145741 | 151.30 | 0.22 | 0.0019 | 0.0342 | 2.27 |
| BTG3 | ENSG00000154640 | 39.82 | 0.23 | 0.0025 | 0.0342 | 1.63 |
| BTN2A1 | ENSG00000112763 | 82.79 | 0.23 | 0.0014 | 0.0342 | 1.36 |
| BTN3A1 | ENSG00000026950 | 184.37 | 0.24 | 0.0026 | 0.0342 | 1.21 |
| BTN3A2 | ENSG00000186470 | 109.70 | 0.28 | 0.0008 | 0.0342 | 1.36 |
| BTN3A3 | ENSG00000111801 | 79.91 | 0.26 | 0.0009 | 0.0342 | 1.39 |
| BTNL3 | ENSG00000168903 | 108.21 | 0.24 | 0.0027 | 0.0342 | 0.23 |
| BTNL8 | ENSG00000113303 | 84.78 | 0.22 | 0.0046 | 0.0352 | 0.17 |
| BTNL9 | ENSG00000165810 | 41.07 | 0.27 | 0.0022 | 0.0342 | 1.32 |
| BTRC | ENSG00000166167 | 67.51 | 0.22 | 0.0027 | 0.0342 | 1.51 |
| BUB3 | ENSG00000154473 | 97.56 | 0.18 | 0.0139 | 0.0469 | 2.18 |
| BZW2 | ENSG00000136261 | 80.84 | 0.21 | 0.0060 | 0.0366 | 2.90 |
| C10orf113 | ENSG00000204683 | 5.71 | 0.23 | 0.0038 | 0.0342 | 3.20 |
| C10orf118 | ENSG00000165813 | 204.96 | 0.19 | 0.0110 | 0.0432 | 1.46 |
| C10orf12 | ENSG00000155640 | 109.36 | 0.22 | 0.0027 | 0.0342 | 1.32 |
| C10orf125 | ENSG00000148803 | 10.55 | 0.25 | 0.0051 | 0.0358 | 1.81 |
| C10orf2 | ENSG00000107815 | 24.23 | 0.18 | 0.0159 | 0.0498 | 2.62 |
| C10orf26 | ENSG00000166272 | 126.15 | 0.20 | 0.0062 | 0.0368 | 1.25 |
| C10orf46 | ENSG00000151893 | 168.78 | 0.20 | 0.0050 | 0.0356 | 1.64 |
| C10orf47 | ENSG00000148426 | 14.66 | 0.20 | 0.0110 | 0.0432 | 1.77 |
| C10orf57 | ENSG00000133678 | 41.36 | 0.20 | 0.0068 | 0.0374 | 1.20 |
| C10orf76 | ENSG00000120029 | 101.91 | 0.18 | 0.0112 | 0.0434 | 1.21 |
| C10orf95 | ENSG00000120055 | 21.80 | 0.26 | 0.0007 | 0.0342 | 1.73 |
| C10orf99 | ENSG00000188373 | 131.68 | 0.18 | 0.0149 | 0.0485 | 0.69 |
| C11orf1 | ENSG00000137720 | 14.96 | 0.21 | 0.0050 | 0.0356 | 2.17 |
| C11orf2 | ENSG00000149823 | 159.82 | 0.20 | 0.0076 | 0.0382 | 1.40 |
| C11orf24 | ENSG00000171067 | 77.51 | 0.21 | 0.0027 | 0.0342 | 1.10 |
| C11orf31 | ENSG00000211450 | 37.94 | 0.19 | 0.0159 | 0.0498 | 1.92 |
| C11orf35 | ENSG00000185522 | 25.67 | 0.25 | 0.0036 | 0.0342 | 1.41 |
| C11orf48 | ENSG00000162194 | 45.43 | 0.24 | 0.0017 | 0.0342 | 1.62 |
| C11orf52 | ENSG00000149300 | 7.49 | 0.23 | 0.0032 | 0.0342 | 1.29 |
| C11orf54 | ENSG00000182919 | 125.56 | 0.26 | 0.0010 | 0.0342 | 1.02 |
| C11orf61 | ENSG00000120458 | 52.23 | 0.20 | 0.0079 | 0.0387 | 1.24 |
| C11orf68 | ENSG00000175573 | 28.71 | 0.21 | 0.0053 | 0.0361 | 1.59 |
| C11orf71 | ENSG00000180425 | 18.46 | 0.24 | 0.0029 | 0.0342 | 1.33 |
| C11orf75 | ENSG00000166002 | 21.20 | 0.21 | 0.0040 | 0.0346 | 1.10 |
| C11orf80 | ENSG00000173715 | 64.39 | 0.19 | 0.0080 | 0.0389 | 1.34 |
| C11orf9 | ENSG00000124920 | 144.35 | 0.28 | 0.0007 | 0.0342 | 1.29 |
| C11orf95 | ENSG00000188070 | 39.53 | 0.20 | 0.0078 | 0.0386 | 2.93 |
| C12orf10 | ENSG00000139637 | 21.20 | 0.20 | 0.0073 | 0.0379 | 1.97 |
| C12orf11 | ENSG00000064102 | 31.66 | 0.20 | 0.0080 | 0.0389 | 2.87 |
| C12orf23 | ENSG00000151135 | 79.37 | 0.20 | 0.0082 | 0.0393 | 1.31 |
| C12orf24 | ENSG00000204856 | 15.58 | 0.22 | 0.0038 | 0.0342 | 1.36 |
| C12orf45 | ENSG00000151131 | 9.28 | 0.25 | 0.0017 | 0.0342 | 3.06 |
| C12orf48 | ENSG00000185480 | 17.01 | 0.19 | 0.0141 | 0.0472 | 3.37 |
| C12orf49 | ENSG00000111412 | 78.01 | 0.18 | 0.0140 | 0.0471 | 1.66 |
| C12orf51 | ENSG00000173064 | 717.14 | 0.20 | 0.0077 | 0.0384 | 1.34 |
| C12orf57 | ENSG00000111678 | 32.51 | 0.18 | 0.0125 | 0.0451 | 0.97 |
| C12orf75 | ENSG00000235162 | 31.99 | 0.20 | 0.0072 | 0.0377 | 2.99 |
| C14orf119 | ENSG00000179933 | 40.81 | 0.18 | 0.0157 | 0.0496 | 1.60 |
| C14orf129 | ENSG00000100744 | 59.29 | 0.22 | 0.0060 | 0.0366 | 1.13 |
| C14orf133 | ENSG00000151445 | 38.41 | 0.26 | 0.0007 | 0.0342 | 1.33 |
| C14orf142 | ENSG00000170270 | 13.28 | 0.20 | 0.0098 | 0.0416 | 1.16 |
| C14orf149 | ENSG00000126790 | 28.50 | 0.22 | 0.0028 | 0.0342 | 1.45 |
| C14orf159 | ENSG00000133943 | 277.94 | 0.21 | 0.0039 | 0.0345 | 0.80 |
| C14orf166 | ENSG00000087302 | 54.52 | 0.20 | 0.0087 | 0.0401 | 2.22 |
| C14orf169 | ENSG00000255242 | 23.28 | 0.24 | 0.0009 | 0.0342 | 1.72 |
| C14orf176 | ENSG00000232070 | 17.36 | 0.20 | 0.0067 | 0.0373 | 0.50 |
| C14orf2 | ENSG00000156411 | 31.51 | 0.20 | 0.0059 | 0.0365 | 1.52 |
| C14orf28 | ENSG00000179476 | 10.97 | 0.22 | 0.0033 | 0.0342 | 1.05 |
| C14orf45 | ENSG00000119636 | 27.88 | 0.21 | 0.0066 | 0.0373 | 0.78 |
| C14orf93 | ENSG00000100802 | 25.33 | 0.27 | 0.0005 | 0.0342 | 1.27 |
| C15orf17 | ENSG00000178761 | 134.47 | 0.27 | 0.0004 | 0.0342 | 1.09 |
| C15orf33 | ENSG00000166262 | 6.76 | 0.25 | 0.0057 | 0.0363 | 1.57 |
| C15orf38 | ENSG00000242498 | 110.81 | 0.23 | 0.0013 | 0.0342 | 0.93 |
| C15orf38-AP3S2 | ENSG00000250021 | 104.22 | 0.19 | 0.0110 | 0.0432 | 1.06 |
| C15orf39 | ENSG00000167173 | 120.81 | 0.21 | 0.0057 | 0.0363 | 1.45 |
| C15orf40 | ENSG00000169609 | 44.06 | 0.19 | 0.0119 | 0.0442 | 1.18 |
| C15orf44 | ENSG00000138614 | 49.98 | 0.22 | 0.0042 | 0.0350 | 1.84 |
| C15orf48 | ENSG00000166920 | 146.34 | 0.25 | 0.0005 | 0.0342 | 0.47 |
| C15orf57 | ENSG00000128891 | 26.53 | 0.21 | 0.0035 | 0.0342 | 1.14 |
| C16orf13 | ENSG00000249124 | 18.10 | 0.20 | 0.0129 | 0.0457 | 3.13 |
| C16orf3 | ENSG00000221819 | 7.46 | 0.19 | 0.0119 | 0.0442 | 1.95 |
| C16orf53 | ENSG00000185928 | 102.04 | 0.22 | 0.0030 | 0.0342 | 1.02 |
| C16orf57 | ENSG00000103005 | 41.29 | 0.21 | 0.0050 | 0.0356 | 1.74 |
| C16orf58 | ENSG00000140688 | 136.55 | 0.21 | 0.0055 | 0.0363 | 1.50 |
| C16orf61 | ENSG00000103121 | 13.94 | 0.20 | 0.0116 | 0.0439 | 2.13 |
| C16orf70 | ENSG00000125149 | 42.72 | 0.22 | 0.0029 | 0.0342 | 1.65 |
| C16orf72 | ENSG00000182831 | 150.78 | 0.19 | 0.0107 | 0.0428 | 1.36 |
| C16orf88 | ENSG00000103550 | 23.84 | 0.27 | 0.0006 | 0.0342 | 2.54 |
| C16orf89 | ENSG00000153446 | 8.42 | 0.18 | 0.0155 | 0.0492 | 0.33 |
| C17orf101 | ENSG00000181396 | 43.98 | 0.19 | 0.0091 | 0.0407 | 1.26 |
| C17orf28 | ENSG00000167861 | 148.22 | 0.20 | 0.0069 | 0.0375 | 1.02 |
| C17orf49 | ENSG00000161939 | 25.58 | 0.19 | 0.0082 | 0.0393 | 1.53 |
| C17orf53 | ENSG00000125319 | 8.41 | 0.21 | 0.0075 | 0.0382 | 3.01 |
| C17orf63 | ENSG00000173065 | 131.42 | 0.19 | 0.0107 | 0.0428 | 1.25 |
| C17orf67 | ENSG00000214226 | 7.37 | 0.24 | 0.0030 | 0.0342 | 1.76 |
| C17orf70 | ENSG00000185504 | 97.30 | 0.19 | 0.0123 | 0.0448 | 1.76 |
| C17orf75 | ENSG00000108666 | 10.68 | 0.20 | 0.0076 | 0.0382 | 2.17 |
| C17orf76 | ENSG00000181350 | 317.59 | 0.22 | 0.0022 | 0.0342 | 1.51 |
| C18orf10 | ENSG00000134779 | 45.35 | 0.23 | 0.0030 | 0.0342 | 1.89 |
| C18orf45 | ENSG00000134490 | 22.43 | 0.20 | 0.0096 | 0.0414 | 1.70 |
| C19orf10 | ENSG00000074842 | 45.04 | 0.21 | 0.0044 | 0.0351 | 1.76 |
| C19orf12 | ENSG00000131943 | 41.31 | 0.20 | 0.0057 | 0.0363 | 1.54 |
| C19orf2 | ENSG00000105176 | 100.27 | 0.21 | 0.0030 | 0.0342 | 2.20 |
| C19orf25 | ENSG00000119559 | 42.57 | 0.23 | 0.0033 | 0.0342 | 1.39 |
| C19orf28 | ENSG00000161091 | 81.96 | 0.19 | 0.0153 | 0.0489 | 2.13 |
| C19orf33 | ENSG00000167644 | 45.73 | 0.23 | 0.0013 | 0.0342 | 0.98 |
| C19orf42 | ENSG00000214046 | 114.97 | 0.19 | 0.0082 | 0.0393 | 1.28 |
| C19orf43 | ENSG00000123144 | 83.11 | 0.22 | 0.0029 | 0.0342 | 1.68 |
| C19orf44 | ENSG00000105072 | 22.46 | 0.22 | 0.0032 | 0.0342 | 1.75 |
| C19orf46 | ENSG00000181392 | 14.90 | 0.22 | 0.0048 | 0.0354 | 1.73 |
| C19orf48 | ENSG00000167747 | 38.11 | 0.19 | 0.0149 | 0.0485 | 3.40 |
| C19orf50 | ENSG00000105700 | 64.17 | 0.21 | 0.0042 | 0.0350 | 1.85 |
| C19orf52 | ENSG00000142444 | 16.86 | 0.25 | 0.0019 | 0.0342 | 1.75 |
| C19orf53 | ENSG00000104979 | 20.98 | 0.19 | 0.0111 | 0.0433 | 2.31 |
| C19orf54 | ENSG00000188493 | 36.86 | 0.19 | 0.0127 | 0.0454 | 1.33 |
| C19orf79 | ENSG00000229833 | 9.30 | 0.26 | 0.0009 | 0.0342 | 1.80 |
| C19orf80 | ENSG00000130173 | 11.81 | 0.23 | 0.0025 | 0.0342 | 1.92 |
| C1GALT1 | ENSG00000106392 | 106.85 | 0.22 | 0.0031 | 0.0342 | 1.86 |
| C1orf106 | ENSG00000163362 | 282.50 | 0.22 | 0.0037 | 0.0342 | 0.90 |
| C1orf109 | ENSG00000116922 | 18.72 | 0.20 | 0.0066 | 0.0373 | 2.10 |
| C1orf115 | ENSG00000162817 | 99.78 | 0.22 | 0.0060 | 0.0366 | 0.48 |
| C1orf123 | ENSG00000162384 | 47.82 | 0.19 | 0.0095 | 0.0413 | 1.26 |
| C1orf131 | ENSG00000143633 | 31.98 | 0.26 | 0.0004 | 0.0342 | 1.61 |
| C1orf144 | ENSG00000055070 | 189.93 | 0.18 | 0.0152 | 0.0489 | 1.45 |
| C1orf159 | ENSG00000131591 | 65.86 | 0.22 | 0.0030 | 0.0342 | 1.42 |
| C1orf172 | ENSG00000175707 | 23.08 | 0.21 | 0.0057 | 0.0363 | 1.31 |
| C1orf174 | ENSG00000198912 | 40.44 | 0.20 | 0.0079 | 0.0387 | 1.46 |
| C1orf182 | ENSG00000163467 | 6.14 | 0.23 | 0.0034 | 0.0342 | 2.21 |
| C1orf192 | ENSG00000188931 | 20.06 | 0.25 | 0.0011 | 0.0342 | 1.48 |
| C1orf195 | ENSG00000204464 | 16.50 | 0.22 | 0.0048 | 0.0354 | 1.35 |
| C1orf198 | ENSG00000119280 | 65.86 | 0.18 | 0.0154 | 0.0491 | 2.83 |
| C1orf21 | ENSG00000116667 | 284.59 | 0.20 | 0.0044 | 0.0351 | 0.60 |
| C1orf210 | ENSG00000253313 | 36.61 | 0.25 | 0.0005 | 0.0342 | 0.70 |
| C1orf212 | ENSG00000163866 | 61.12 | 0.20 | 0.0067 | 0.0373 | 1.16 |
| C1orf226 | ENSG00000239887 | 74.15 | 0.17 | 0.0153 | 0.0489 | 1.11 |
| C1orf229 | ENSG00000221953 | 2.19 | 0.22 | 0.0084 | 0.0397 | 1.44 |
| C1orf27 | ENSG00000157181 | 79.71 | 0.19 | 0.0091 | 0.0407 | 1.54 |
| C1orf43 | ENSG00000143612 | 154.19 | 0.24 | 0.0009 | 0.0342 | 2.13 |
| C1orf50 | ENSG00000164008 | 17.75 | 0.24 | 0.0020 | 0.0342 | 1.22 |
| C1orf63 | ENSG00000117616 | 336.35 | 0.24 | 0.0018 | 0.0342 | 0.85 |
| C1orf85 | ENSG00000198715 | 32.17 | 0.25 | 0.0010 | 0.0342 | 1.76 |
| C1orf86 | ENSG00000162585 | 61.40 | 0.19 | 0.0106 | 0.0427 | 1.36 |
| C1orf93 | ENSG00000157870 | 91.79 | 0.23 | 0.0019 | 0.0342 | 1.70 |
| C1QTNF3 | ENSG00000082196 | 93.09 | 0.20 | 0.0064 | 0.0370 | 2.31 |
| C1RL | ENSG00000139178 | 152.71 | 0.19 | 0.0103 | 0.0423 | 1.35 |
| C20orf108 | ENSG00000124098 | 70.99 | 0.26 | 0.0006 | 0.0342 | 2.74 |
| C20orf11 | ENSG00000101193 | 73.86 | 0.20 | 0.0067 | 0.0373 | 2.55 |
| C20orf134 | ENSG00000182584 | 19.06 | 0.21 | 0.0065 | 0.0371 | 1.80 |
| C20orf151 | ENSG00000130701 | 12.81 | 0.26 | 0.0013 | 0.0342 | 3.44 |
| C20orf29 | ENSG00000125843 | 22.94 | 0.20 | 0.0087 | 0.0401 | 1.24 |
| C20orf3 | ENSG00000101474 | 70.66 | 0.18 | 0.0119 | 0.0442 | 2.80 |
| C20orf30 | ENSG00000089063 | 76.21 | 0.21 | 0.0042 | 0.0350 | 2.04 |
| C20orf72 | ENSG00000125871 | 37.79 | 0.20 | 0.0101 | 0.0421 | 2.03 |
| C20orf94 | ENSG00000149346 | 14.77 | 0.19 | 0.0082 | 0.0393 | 1.85 |
| C21orf2 | ENSG00000160226 | 120.91 | 0.20 | 0.0080 | 0.0389 | 0.91 |
| C21orf33 | ENSG00000160221 | 122.91 | 0.21 | 0.0047 | 0.0353 | 1.15 |
| C21orf58 | ENSG00000160298 | 35.68 | 0.24 | 0.0024 | 0.0342 | 1.62 |
| C21orf59 | ENSG00000159079 | 36.98 | 0.19 | 0.0090 | 0.0405 | 1.89 |
| C21orf88 | ENSG00000184809 | 65.23 | 0.25 | 0.0013 | 0.0342 | 0.04 |
| C22orf13 | ENSG00000138867 | 220.48 | 0.22 | 0.0025 | 0.0342 | 1.15 |
| C22orf26 | ENSG00000182257 | 24.46 | 0.22 | 0.0052 | 0.0359 | 1.24 |
| C22orf28 | ENSG00000100220 | 84.54 | 0.19 | 0.0087 | 0.0401 | 1.42 |
| C22orf29 | ENSG00000215012 | 93.05 | 0.18 | 0.0160 | 0.0499 | 2.59 |
| C22orf36 | ENSG00000178026 | 22.60 | 0.23 | 0.0015 | 0.0342 | 1.40 |
| C22orf40 | ENSG00000205643 | 9.65 | 0.28 | 0.0004 | 0.0342 | 1.27 |
| C22orf46 | ENSG00000184208 | 43.07 | 0.24 | 0.0017 | 0.0342 | 1.14 |
| C2CD2 | ENSG00000157617 | 105.33 | 0.22 | 0.0045 | 0.0352 | 1.92 |
| C2CD2L | ENSG00000172375 | 87.94 | 0.19 | 0.0133 | 0.0462 | 0.97 |
| C2CD3 | ENSG00000168014 | 176.26 | 0.17 | 0.0153 | 0.0489 | 1.33 |
| C2CD4A | ENSG00000198535 | 9.46 | 0.21 | 0.0118 | 0.0441 | 6.03 |
| C2orf15 | ENSG00000241962 | 64.95 | 0.19 | 0.0119 | 0.0442 | 2.06 |
| C2orf18 | ENSG00000213699 | 90.22 | 0.18 | 0.0132 | 0.0460 | 1.90 |
| C2orf28 | ENSG00000138085 | 51.94 | 0.19 | 0.0103 | 0.0423 | 1.78 |
| C2orf29 | ENSG00000158435 | 99.87 | 0.23 | 0.0017 | 0.0342 | 1.88 |
| C2orf3 | ENSG00000005436 | 46.62 | 0.18 | 0.0158 | 0.0497 | 1.68 |
| C2orf43 | ENSG00000118961 | 53.55 | 0.24 | 0.0018 | 0.0342 | 1.51 |
| C2orf44 | ENSG00000163026 | 15.22 | 0.20 | 0.0082 | 0.0393 | 2.04 |
| C2orf54 | ENSG00000172478 | 9.23 | 0.20 | 0.0106 | 0.0427 | 2.89 |
| C2orf55 | ENSG00000196872 | 39.45 | 0.26 | 0.0004 | 0.0342 | 1.51 |
| C2orf68 | ENSG00000168887 | 97.22 | 0.23 | 0.0018 | 0.0342 | 1.44 |
| C2orf69 | ENSG00000178074 | 37.66 | 0.20 | 0.0119 | 0.0442 | 1.90 |
| C2orf72 | ENSG00000204128 | 146.20 | 0.28 | 0.0002 | 0.0342 | 0.42 |
| C2orf82 | ENSG00000182600 | 76.03 | 0.19 | 0.0138 | 0.0468 | 1.25 |
| C2orf88 | ENSG00000187699 | 158.57 | 0.22 | 0.0041 | 0.0349 | 0.48 |
| C3orf19 | ENSG00000154781 | 53.99 | 0.18 | 0.0148 | 0.0484 | 1.34 |
| C3orf23 | ENSG00000179152 | 72.55 | 0.19 | 0.0107 | 0.0428 | 1.49 |
| C3orf65 | ENSG00000163915 | 33.51 | 0.18 | 0.0147 | 0.0482 | 1.49 |
| C3orf70 | ENSG00000187068 | 75.47 | 0.19 | 0.0086 | 0.0399 | 0.82 |
| C4orf14 | ENSG00000084092 | 38.57 | 0.23 | 0.0025 | 0.0342 | 1.80 |
| C4orf3 | ENSG00000164096 | 72.83 | 0.19 | 0.0089 | 0.0403 | 1.66 |
| C4orf33 | ENSG00000151470 | 29.52 | 0.20 | 0.0048 | 0.0354 | 0.98 |
| C4orf34 | ENSG00000163683 | 85.57 | 0.19 | 0.0074 | 0.0380 | 0.63 |
| C4orf36 | ENSG00000163633 | 30.47 | 0.26 | 0.0009 | 0.0342 | 1.00 |
| C4orf41 | ENSG00000168538 | 101.90 | 0.18 | 0.0116 | 0.0439 | 1.37 |
| C4orf52 | ENSG00000250317 | 20.39 | 0.21 | 0.0049 | 0.0355 | 1.56 |
| C4orf7 | ENSG00000181617 | 12.14 | -0.27 | 0.0024 | 0.0342 | 0.17 |
| C5orf15 | ENSG00000113583 | 43.86 | 0.21 | 0.0082 | 0.0393 | 1.75 |
| C5orf22 | ENSG00000082213 | 44.03 | 0.21 | 0.0074 | 0.0380 | 1.62 |
| C5orf30 | ENSG00000181751 | 49.68 | 0.23 | 0.0038 | 0.0342 | 1.04 |
| C5orf32 | ENSG00000120306 | 105.49 | 0.19 | 0.0134 | 0.0463 | 0.97 |
| C5orf4 | ENSG00000170271 | 80.67 | 0.19 | 0.0128 | 0.0456 | 1.33 |
| C5orf43 | ENSG00000188725 | 81.41 | 0.21 | 0.0062 | 0.0368 | 1.65 |
| C5orf51 | ENSG00000205765 | 83.59 | 0.19 | 0.0158 | 0.0497 | 1.72 |
| C5orf55 | ENSG00000221990 | 5.58 | 0.23 | 0.0030 | 0.0342 | 1.29 |
| C5orf63 | ENSG00000164241 | 55.23 | 0.21 | 0.0068 | 0.0374 | 0.85 |
| C5orf65 | ENSG00000228672 | 77.58 | 0.26 | 0.0005 | 0.0342 | 0.57 |
| C6orf106 | ENSG00000196821 | 193.02 | 0.20 | 0.0064 | 0.0370 | 1.82 |
| C6orf108 | ENSG00000112667 | 24.66 | 0.22 | 0.0046 | 0.0352 | 2.00 |
| C6orf115 | ENSG00000146386 | 21.32 | 0.24 | 0.0029 | 0.0342 | 2.14 |
| C6orf120 | ENSG00000185127 | 75.70 | 0.18 | 0.0121 | 0.0445 | 1.48 |
| C6orf123 | ENSG00000146521 | 5.37 | 0.21 | 0.0104 | 0.0424 | 4.22 |
| C6orf130 | ENSG00000124596 | 67.42 | 0.20 | 0.0054 | 0.0362 | 1.43 |
| C6orf132 | ENSG00000188112 | 172.25 | 0.19 | 0.0110 | 0.0432 | 1.68 |
| C6orf136 | ENSG00000204564 | 53.90 | 0.22 | 0.0045 | 0.0352 | 0.88 |
| C6orf165 | ENSG00000213204 | 61.27 | 0.21 | 0.0037 | 0.0342 | 0.87 |
| C6orf192 | ENSG00000146409 | 33.11 | 0.19 | 0.0118 | 0.0441 | 1.43 |
| C6orf203 | ENSG00000130349 | 20.21 | 0.20 | 0.0083 | 0.0395 | 1.48 |
| C6orf222 | ENSG00000189325 | 35.95 | 0.29 | 0.0003 | 0.0342 | 2.80 |
| C6orf223 | ENSG00000181577 | 6.79 | 0.24 | 0.0078 | 0.0386 | 16.08 |
| C6orf228 | ENSG00000224531 | 22.54 | 0.19 | 0.0139 | 0.0469 | 2.26 |
| C6orf35 | ENSG00000215712 | 22.04 | 0.23 | 0.0020 | 0.0342 | 1.17 |
| C6orf62 | ENSG00000112308 | 325.58 | 0.20 | 0.0066 | 0.0373 | 1.70 |
| C6orf64 | ENSG00000112167 | 22.41 | 0.19 | 0.0112 | 0.0434 | 1.84 |
| C6orf89 | ENSG00000198663 | 80.93 | 0.19 | 0.0101 | 0.0421 | 1.46 |
| C7orf26 | ENSG00000146576 | 46.95 | 0.20 | 0.0078 | 0.0386 | 1.51 |
| C7orf29 | ENSG00000188707 | 27.48 | 0.22 | 0.0047 | 0.0353 | 2.33 |
| C7orf36 | ENSG00000241127 | 17.86 | 0.18 | 0.0155 | 0.0492 | 3.35 |
| C7orf42 | ENSG00000106609 | 286.26 | 0.19 | 0.0079 | 0.0387 | 1.69 |
| C7orf43 | ENSG00000146826 | 85.16 | 0.24 | 0.0022 | 0.0342 | 0.99 |
| C7orf50 | ENSG00000146540 | 52.45 | 0.19 | 0.0119 | 0.0442 | 2.17 |
| C7orf59 | ENSG00000188186 | 68.50 | 0.21 | 0.0034 | 0.0342 | 1.25 |
| C7orf68 | ENSG00000135245 | 11.65 | 0.21 | 0.0062 | 0.0368 | 6.24 |
| C7orf70 | ENSG00000178397 | 15.97 | 0.23 | 0.0028 | 0.0342 | 2.33 |
| C7orf73 | ENSG00000243317 | 54.12 | 0.20 | 0.0060 | 0.0366 | 1.77 |
| C7orf74 | ENSG00000253276 | 46.59 | 0.18 | 0.0147 | 0.0482 | 2.60 |
| C8orf33 | ENSG00000182307 | 73.67 | 0.29 | <.0001 | 0.0342 | 2.15 |
| C8orf44 | ENSG00000213865 | 25.38 | 0.19 | 0.0128 | 0.0456 | 1.59 |
| C8orf47 | ENSG00000177459 | 5.57 | 0.22 | 0.0071 | 0.0376 | 0.76 |
| C8orf55 | ENSG00000130193 | 33.95 | 0.22 | 0.0026 | 0.0342 | 2.96 |
| C8orf59 | ENSG00000176731 | 36.83 | 0.21 | 0.0075 | 0.0382 | 2.00 |
| C8orf73 | ENSG00000204839 | 36.20 | 0.26 | 0.0022 | 0.0342 | 2.99 |
| C8orf83 | ENSG00000205133 | 54.93 | 0.26 | 0.0005 | 0.0342 | 1.21 |
| C9orf100 | ENSG00000137135 | 41.07 | 0.22 | 0.0044 | 0.0351 | 1.95 |
| C9orf102 | ENSG00000182150 | 137.63 | 0.21 | 0.0044 | 0.0351 | 1.32 |
| C9orf114 | ENSG00000198917 | 67.45 | 0.24 | 0.0017 | 0.0342 | 1.80 |
| C9orf123 | ENSG00000137038 | 33.09 | 0.27 | 0.0003 | 0.0342 | 1.82 |
| C9orf125 | ENSG00000165152 | 74.42 | 0.25 | 0.0010 | 0.0342 | 0.90 |
| C9orf129 | ENSG00000204352 | 42.64 | 0.23 | 0.0015 | 0.0342 | 1.15 |
| C9orf140 | ENSG00000186193 | 39.94 | 0.28 | 0.0015 | 0.0342 | 4.99 |
| C9orf152 | ENSG00000188959 | 116.12 | 0.32 | <.0001 | 0.0342 | 1.14 |
| C9orf16 | ENSG00000171159 | 37.03 | 0.21 | 0.0073 | 0.0379 | 2.21 |
| C9orf172 | ENSG00000232434 | 29.33 | 0.19 | 0.0159 | 0.0498 | 1.65 |
| C9orf23 | ENSG00000164967 | 10.57 | 0.20 | 0.0107 | 0.0428 | 1.72 |
| C9orf40 | ENSG00000135045 | 15.90 | 0.23 | 0.0032 | 0.0342 | 1.60 |
| C9orf41 | ENSG00000156017 | 62.16 | 0.20 | 0.0097 | 0.0415 | 2.12 |
| C9orf46 | ENSG00000107020 | 19.24 | 0.26 | 0.0004 | 0.0342 | 1.48 |
| C9orf5 | ENSG00000106771 | 189.91 | 0.23 | 0.0013 | 0.0342 | 1.80 |
| C9orf50 | ENSG00000179058 | 9.40 | 0.23 | 0.0054 | 0.0362 | 4.43 |
| C9orf68 | ENSG00000106686 | 66.25 | 0.29 | <.0001 | 0.0342 | 1.55 |
| C9orf69 | ENSG00000238227 | 76.51 | 0.22 | 0.0046 | 0.0352 | 1.66 |
| C9orf7 | ENSG00000160325 | 88.12 | 0.24 | 0.0026 | 0.0342 | 0.86 |
| C9orf78 | ENSG00000136819 | 63.54 | 0.18 | 0.0121 | 0.0445 | 1.61 |
| C9orf82 | ENSG00000120159 | 46.08 | 0.19 | 0.0120 | 0.0444 | 1.45 |
| C9orf85 | ENSG00000155621 | 24.75 | 0.22 | 0.0058 | 0.0364 | 1.36 |
| C9orf86 | ENSG00000196642 | 353.02 | 0.22 | 0.0033 | 0.0342 | 1.87 |
| C9orf91 | ENSG00000157693 | 47.94 | 0.19 | 0.0098 | 0.0416 | 1.37 |
| C9orf93 | ENSG00000164989 | 63.15 | 0.18 | 0.0150 | 0.0486 | 0.75 |
| CA12 | ENSG00000074410 | 739.78 | 0.18 | 0.0148 | 0.0484 | 0.33 |
| CA13 | ENSG00000185015 | 57.33 | 0.19 | 0.0128 | 0.0456 | 1.17 |
| CA8 | ENSG00000178538 | 23.71 | 0.20 | 0.0120 | 0.0444 | 1.37 |
| CABIN1 | ENSG00000099991 | 337.58 | 0.19 | 0.0114 | 0.0437 | 1.33 |
| CABLES1 | ENSG00000134508 | 41.71 | 0.21 | 0.0074 | 0.0380 | 1.38 |
| CACNA1F | ENSG00000102001 | 12.97 | 0.21 | 0.0079 | 0.0387 | 0.42 |
| CACNB3 | ENSG00000167535 | 59.97 | 0.19 | 0.0096 | 0.0414 | 1.46 |
| CAD | ENSG00000084774 | 132.72 | 0.21 | 0.0057 | 0.0363 | 2.93 |
| CADPS | ENSG00000163618 | 56.99 | 0.20 | 0.0109 | 0.0431 | 3.55 |
| CADPS2 | ENSG00000081803 | 82.51 | 0.19 | 0.0094 | 0.0411 | 1.37 |
| CALCOCO1 | ENSG00000012822 | 156.13 | 0.25 | 0.0014 | 0.0342 | 1.38 |
| CALCOCO2 | ENSG00000136436 | 352.67 | 0.22 | 0.0023 | 0.0342 | 0.92 |
| CALM2 | ENSG00000143933 | 462.86 | 0.21 | 0.0037 | 0.0342 | 1.42 |
| CALM3 | ENSG00000160014 | 272.27 | 0.21 | 0.0037 | 0.0342 | 1.32 |
| CALML4 | ENSG00000129007 | 198.73 | 0.21 | 0.0033 | 0.0342 | 1.02 |
| CAMK1D | ENSG00000183049 | 223.10 | 0.20 | 0.0059 | 0.0365 | 0.86 |
| CAMK2D | ENSG00000145349 | 231.62 | 0.20 | 0.0061 | 0.0368 | 0.91 |
| CAMK2G | ENSG00000148660 | 152.16 | 0.25 | 0.0007 | 0.0342 | 1.24 |
| CAMK2N1 | ENSG00000162545 | 211.41 | 0.20 | 0.0057 | 0.0363 | 0.99 |
| CAMKK2 | ENSG00000110931 | 175.56 | 0.23 | 0.0019 | 0.0342 | 1.35 |
| CAMLG | ENSG00000164615 | 21.03 | 0.19 | 0.0085 | 0.0399 | 2.20 |
| CAMSAP1 | ENSG00000130559 | 86.37 | 0.20 | 0.0074 | 0.0380 | 2.26 |
| CAMSAP3 | ENSG00000076826 | 127.13 | 0.19 | 0.0129 | 0.0457 | 1.52 |
| CANT1 | ENSG00000171302 | 453.51 | 0.25 | <.0001 | 0.0342 | 0.97 |
| CANX | ENSG00000127022 | 821.84 | 0.22 | 0.0030 | 0.0342 | 2.29 |
| CAP1 | ENSG00000131236 | 306.67 | 0.19 | 0.0115 | 0.0438 | 1.55 |
| CAPG | ENSG00000042493 | 52.12 | 0.20 | 0.0058 | 0.0364 | 2.20 |
| CAPN1 | ENSG00000014216 | 312.27 | 0.21 | 0.0039 | 0.0345 | 1.60 |
| CAPN10 | ENSG00000142330 | 104.73 | 0.22 | 0.0050 | 0.0356 | 2.09 |
| CAPN13 | ENSG00000162949 | 118.33 | 0.22 | 0.0034 | 0.0342 | 0.32 |
| CAPN2 | ENSG00000162909 | 658.35 | 0.21 | 0.0028 | 0.0342 | 0.97 |
| CAPN3 | ENSG00000092529 | 98.26 | 0.19 | 0.0149 | 0.0485 | 0.89 |
| CAPN5 | ENSG00000149260 | 569.87 | 0.25 | 0.0006 | 0.0342 | 0.61 |
| CAPN9 | ENSG00000135773 | 83.51 | 0.26 | 0.0009 | 0.0342 | 0.35 |
| CAPNS1 | ENSG00000126247 | 247.22 | 0.20 | 0.0078 | 0.0386 | 1.52 |
| CAPRIN1 | ENSG00000135387 | 434.43 | 0.21 | 0.0047 | 0.0353 | 2.39 |
| CAPRIN2 | ENSG00000110888 | 85.22 | 0.18 | 0.0139 | 0.0469 | 1.67 |
| CAPS | ENSG00000105519 | 66.33 | 0.18 | 0.0144 | 0.0477 | 1.30 |
| CAPZA2 | ENSG00000198898 | 109.36 | 0.18 | 0.0132 | 0.0460 | 2.22 |
| CARD10 | ENSG00000100065 | 148.82 | 0.22 | 0.0063 | 0.0370 | 1.36 |
| CARD14 | ENSG00000141527 | 18.94 | 0.23 | 0.0068 | 0.0374 | 1.86 |
| CARD16 | ENSG00000204397 | 13.25 | 0.19 | 0.0108 | 0.0429 | 1.02 |
| CARHSP1 | ENSG00000153048 | 110.39 | 0.21 | 0.0043 | 0.0351 | 1.73 |
| CARM1 | ENSG00000142453 | 109.52 | 0.21 | 0.0035 | 0.0342 | 2.03 |
| CARS | ENSG00000110619 | 122.06 | 0.19 | 0.0081 | 0.0391 | 1.57 |
| CASC4 | ENSG00000166734 | 283.38 | 0.22 | 0.0015 | 0.0342 | 1.28 |
| CASK | ENSG00000147044 | 289.71 | 0.19 | 0.0076 | 0.0382 | 1.85 |
| CASP1 | ENSG00000137752 | 54.33 | 0.23 | 0.0046 | 0.0352 | 1.05 |
| CASP10 | ENSG00000003400 | 207.78 | 0.18 | 0.0122 | 0.0446 | 0.78 |
| CASP3 | ENSG00000164305 | 78.25 | 0.23 | 0.0027 | 0.0342 | 1.22 |
| CASP5 | ENSG00000137757 | 22.03 | 0.23 | 0.0029 | 0.0342 | 0.51 |
| CASP6 | ENSG00000138794 | 37.12 | 0.22 | 0.0032 | 0.0342 | 1.53 |
| CASP8 | ENSG00000064012 | 97.42 | 0.20 | 0.0086 | 0.0399 | 1.70 |
| CAST | ENSG00000153113 | 1028.47 | 0.18 | 0.0149 | 0.0485 | 1.10 |
| CASZ1 | ENSG00000130940 | 254.63 | 0.18 | 0.0132 | 0.0460 | 0.86 |
| CAT | ENSG00000121691 | 99.39 | 0.20 | 0.0057 | 0.0363 | 1.44 |
| CATSPER2 | ENSG00000166762 | 67.03 | 0.18 | 0.0133 | 0.0462 | 1.01 |
| CBFA2T2 | ENSG00000078699 | 245.48 | 0.20 | 0.0071 | 0.0376 | 1.71 |
| CBLC | ENSG00000142273 | 79.66 | 0.23 | 0.0027 | 0.0342 | 1.24 |
| CBR3 | ENSG00000159231 | 16.52 | 0.26 | 0.0010 | 0.0342 | 1.12 |
| CBR4 | ENSG00000145439 | 97.09 | 0.23 | 0.0011 | 0.0342 | 1.46 |
| CBX1 | ENSG00000108468 | 43.21 | 0.20 | 0.0080 | 0.0389 | 2.37 |
| CBX3 | ENSG00000122565 | 106.11 | 0.19 | 0.0102 | 0.0422 | 3.42 |
| CBY1 | ENSG00000100211 | 29.69 | 0.19 | 0.0098 | 0.0416 | 1.56 |
| CC2D1A | ENSG00000132024 | 145.09 | 0.19 | 0.0127 | 0.0454 | 1.46 |
| CCDC101 | ENSG00000176476 | 15.96 | 0.18 | 0.0153 | 0.0489 | 1.42 |
| CCDC107 | ENSG00000159884 | 18.69 | 0.19 | 0.0116 | 0.0439 | 1.38 |
| CCDC111 | ENSG00000164306 | 21.87 | 0.23 | 0.0029 | 0.0342 | 1.33 |
| CCDC115 | ENSG00000136710 | 34.80 | 0.20 | 0.0064 | 0.0370 | 1.52 |
| CCDC117 | ENSG00000159873 | 75.96 | 0.19 | 0.0112 | 0.0434 | 1.61 |
| CCDC125 | ENSG00000183323 | 96.40 | 0.28 | 0.0004 | 0.0342 | 1.10 |
| CCDC127 | ENSG00000164366 | 13.12 | 0.20 | 0.0088 | 0.0402 | 1.81 |
| CCDC132 | ENSG00000004766 | 67.14 | 0.20 | 0.0066 | 0.0373 | 1.58 |
| CCDC14 | ENSG00000175455 | 587.42 | 0.19 | 0.0095 | 0.0413 | 1.10 |
| CCDC142 | ENSG00000135637 | 59.19 | 0.23 | 0.0024 | 0.0342 | 1.44 |
| CCDC152 | ENSG00000198865 | 637.01 | 0.24 | 0.0024 | 0.0342 | 0.24 |
| CCDC153 | ENSG00000248712 | 41.40 | 0.27 | 0.0016 | 0.0342 | 0.43 |
| CCDC165 | ENSG00000168502 | 61.94 | 0.20 | 0.0130 | 0.0458 | 1.89 |
| CCDC24 | ENSG00000159214 | 40.23 | 0.22 | 0.0040 | 0.0346 | 1.33 |
| CCDC25 | ENSG00000147419 | 54.75 | 0.19 | 0.0085 | 0.0399 | 1.25 |
| CCDC28A | ENSG00000024862 | 14.48 | 0.20 | 0.0082 | 0.0393 | 1.52 |
| CCDC34 | ENSG00000109881 | 14.78 | 0.19 | 0.0149 | 0.0485 | 2.54 |
| CCDC43 | ENSG00000180329 | 30.64 | 0.20 | 0.0097 | 0.0415 | 2.21 |
| CCDC47 | ENSG00000108588 | 185.15 | 0.20 | 0.0046 | 0.0352 | 1.55 |
| CCDC51 | ENSG00000164051 | 13.36 | 0.19 | 0.0131 | 0.0460 | 2.20 |
| CCDC53 | ENSG00000120860 | 33.40 | 0.19 | 0.0138 | 0.0468 | 1.82 |
| CCDC56 | ENSG00000183978 | 42.68 | 0.18 | 0.0136 | 0.0466 | 1.59 |
| CCDC57 | ENSG00000176155 | 203.05 | 0.20 | 0.0067 | 0.0373 | 1.47 |
| CCDC6 | ENSG00000108091 | 200.75 | 0.23 | 0.0022 | 0.0342 | 1.95 |
| CCDC64B | ENSG00000162069 | 100.15 | 0.18 | 0.0151 | 0.0487 | 1.02 |
| CCDC68 | ENSG00000166510 | 96.63 | 0.18 | 0.0160 | 0.0499 | 0.44 |
| CCDC71 | ENSG00000177352 | 24.66 | 0.19 | 0.0122 | 0.0446 | 1.28 |
| CCDC74A | ENSG00000163040 | 3.94 | 0.23 | 0.0047 | 0.0353 | 3.33 |
| CCDC78 | ENSG00000162004 | 14.42 | 0.23 | 0.0031 | 0.0342 | 2.61 |
| CCDC93 | ENSG00000125633 | 219.85 | 0.22 | 0.0016 | 0.0342 | 1.54 |
| CCHCR1 | ENSG00000204536 | 65.11 | 0.23 | 0.0030 | 0.0342 | 1.37 |
| CCL14 | ENSG00000213494 | 38.53 | 0.18 | 0.0141 | 0.0472 | 0.55 |
| CCL15 | ENSG00000161574 | 32.48 | 0.19 | 0.0134 | 0.0463 | 0.58 |
| CCL20 | ENSG00000115009 | 17.41 | 0.29 | 0.0025 | 0.0342 | 4.41 |
| CCL28 | ENSG00000151882 | 235.74 | 0.31 | <.0001 | 0.0342 | 0.28 |
| CCNC | ENSG00000112237 | 110.06 | 0.19 | 0.0104 | 0.0424 | 1.83 |
| CCND1 | ENSG00000110092 | 211.42 | 0.27 | 0.0022 | 0.0342 | 3.52 |
| CCND2 | ENSG00000118971 | 887.65 | 0.18 | 0.0160 | 0.0499 | 2.19 |
| CCNDBP1 | ENSG00000166946 | 53.95 | 0.20 | 0.0053 | 0.0361 | 1.07 |
| CCNE2 | ENSG00000175305 | 34.29 | 0.23 | 0.0031 | 0.0342 | 1.71 |
| CCNF | ENSG00000162063 | 27.32 | 0.22 | 0.0082 | 0.0393 | 4.27 |
| CCNG1 | ENSG00000113328 | 157.19 | 0.25 | 0.0012 | 0.0342 | 1.44 |
| CCNG2 | ENSG00000138764 | 123.75 | 0.22 | 0.0035 | 0.0342 | 0.98 |
| CCNI | ENSG00000118816 | 480.53 | 0.19 | 0.0068 | 0.0374 | 1.48 |
| CCNI2 | ENSG00000205089 | 25.13 | 0.20 | 0.0106 | 0.0427 | 2.28 |
| CCNJ | ENSG00000107443 | 42.63 | 0.18 | 0.0141 | 0.0472 | 1.73 |
| CCNL2 | ENSG00000221978 | 432.03 | 0.20 | 0.0071 | 0.0376 | 1.37 |
| CCNO | ENSG00000152669 | 3.89 | 0.25 | 0.0013 | 0.0342 | 3.99 |
| CCNY | ENSG00000108100 | 133.23 | 0.18 | 0.0112 | 0.0434 | 1.77 |
| CCP110 | ENSG00000103540 | 69.44 | 0.19 | 0.0112 | 0.0434 | 1.67 |
| CCR2 | ENSG00000121807 | 18.24 | 0.20 | 0.0098 | 0.0416 | 0.52 |
| CCRL2 | ENSG00000121797 | 15.74 | 0.21 | 0.0038 | 0.0342 | 1.31 |
| CCS | ENSG00000173992 | 40.81 | 0.21 | 0.0059 | 0.0365 | 1.18 |
| CCT3 | ENSG00000163468 | 111.01 | 0.19 | 0.0072 | 0.0377 | 3.38 |
| CCT4 | ENSG00000115484 | 71.93 | 0.24 | 0.0010 | 0.0342 | 3.78 |
| CCT5 | ENSG00000150753 | 123.03 | 0.19 | 0.0086 | 0.0399 | 2.91 |
| CCT6A | ENSG00000146731 | 126.66 | 0.19 | 0.0101 | 0.0421 | 3.35 |
| CCT6B | ENSG00000132141 | 5.48 | 0.20 | 0.0078 | 0.0386 | 1.25 |
| CCT8 | ENSG00000156261 | 76.22 | 0.19 | 0.0128 | 0.0456 | 2.57 |
| CD14 | ENSG00000170458 | 31.02 | 0.18 | 0.0105 | 0.0426 | 0.94 |
| CD151 | ENSG00000177697 | 258.21 | 0.23 | 0.0019 | 0.0342 | 1.45 |
| CD164 | ENSG00000135535 | 734.29 | 0.24 | 0.0017 | 0.0342 | 1.34 |
| CD22 | ENSG00000012124 | 45.54 | -0.20 | 0.0136 | 0.0466 | 0.21 |
| CD276 | ENSG00000103855 | 73.67 | 0.20 | 0.0062 | 0.0368 | 2.63 |
| CD2AP | ENSG00000198087 | 313.45 | 0.22 | 0.0031 | 0.0342 | 1.44 |
| CD2BP2 | ENSG00000169217 | 84.02 | 0.20 | 0.0094 | 0.0411 | 1.71 |
| CD300E | ENSG00000186407 | 11.70 | -0.22 | 0.0111 | 0.0433 | 1.09 |
| CD46 | ENSG00000117335 | 463.77 | 0.23 | 0.0029 | 0.0342 | 3.31 |
| CD63 | ENSG00000135404 | 220.34 | 0.19 | 0.0105 | 0.0426 | 1.31 |
| CD82 | ENSG00000085117 | 53.34 | 0.20 | 0.0074 | 0.0380 | 1.67 |
| CDAN1 | ENSG00000140326 | 62.60 | 0.18 | 0.0134 | 0.0463 | 1.17 |
| CDC123 | ENSG00000151465 | 42.95 | 0.19 | 0.0123 | 0.0448 | 2.63 |
| CDC14B | ENSG00000081377 | 76.34 | 0.19 | 0.0086 | 0.0399 | 1.87 |
| CDC16 | ENSG00000130177 | 100.92 | 0.18 | 0.0142 | 0.0474 | 2.27 |
| CDC25B | ENSG00000101224 | 95.58 | 0.19 | 0.0151 | 0.0487 | 4.39 |
| CDC25C | ENSG00000158402 | 6.63 | 0.20 | 0.0113 | 0.0435 | 3.45 |
| CDC42 | ENSG00000070831 | 269.98 | 0.18 | 0.0142 | 0.0474 | 1.29 |
| CDC42BPB | ENSG00000198752 | 443.79 | 0.22 | 0.0022 | 0.0342 | 1.34 |
| CDC42BPG | ENSG00000171219 | 149.21 | 0.24 | 0.0025 | 0.0342 | 0.88 |
| CDC42EP1 | ENSG00000128283 | 127.20 | 0.24 | 0.0034 | 0.0342 | 1.45 |
| CDC42EP3 | ENSG00000163171 | 95.09 | 0.18 | 0.0128 | 0.0456 | 1.26 |
| CDC42EP5 | ENSG00000167617 | 87.06 | 0.24 | 0.0019 | 0.0342 | 0.61 |
| CDC5L | ENSG00000096401 | 94.07 | 0.21 | 0.0053 | 0.0361 | 2.08 |
| CDCA5 | ENSG00000146670 | 15.63 | 0.20 | 0.0094 | 0.0411 | 4.97 |
| CDCA7L | ENSG00000164649 | 67.89 | 0.22 | 0.0040 | 0.0346 | 2.28 |
| CDCA8 | ENSG00000134690 | 36.79 | 0.20 | 0.0084 | 0.0397 | 2.02 |
| CDCP1 | ENSG00000163814 | 246.09 | 0.26 | 0.0007 | 0.0342 | 1.41 |
| CDH1 | ENSG00000039068 | 856.45 | 0.25 | 0.0003 | 0.0342 | 1.68 |
| CDH11 | ENSG00000140937 | 75.30 | 0.20 | 0.0067 | 0.0373 | 3.72 |
| CDH17 | ENSG00000079112 | 767.43 | 0.21 | 0.0035 | 0.0342 | 1.21 |
| CDH3 | ENSG00000062038 | 8.38 | 0.25 | 0.0066 | 0.0373 | 15.60 |
| CDHR1 | ENSG00000148600 | 510.37 | 0.20 | 0.0082 | 0.0393 | 1.36 |
| CDHR3 | ENSG00000128536 | 53.93 | 0.23 | 0.0014 | 0.0342 | 1.33 |
| CDHR5 | ENSG00000099834 | 668.38 | 0.22 | 0.0056 | 0.0363 | 0.39 |
| CDIPT | ENSG00000103502 | 64.32 | 0.20 | 0.0074 | 0.0380 | 1.90 |
| CDK12 | ENSG00000167258 | 328.46 | 0.19 | 0.0107 | 0.0428 | 1.71 |
| CDK16 | ENSG00000102225 | 153.79 | 0.18 | 0.0141 | 0.0472 | 1.83 |
| CDK18 | ENSG00000117266 | 100.28 | 0.27 | 0.0006 | 0.0342 | 1.92 |
| CDK19 | ENSG00000155111 | 102.02 | 0.25 | 0.0009 | 0.0342 | 1.64 |
| CDK2AP1 | ENSG00000111328 | 59.36 | 0.19 | 0.0102 | 0.0422 | 2.08 |
| CDK5RAP2 | ENSG00000136861 | 154.46 | 0.17 | 0.0155 | 0.0492 | 1.64 |
| CDK5RAP3 | ENSG00000108465 | 159.26 | 0.22 | 0.0039 | 0.0345 | 1.28 |
| CDK6 | ENSG00000105810 | 275.01 | 0.19 | 0.0078 | 0.0386 | 2.40 |
| CDK7 | ENSG00000134058 | 22.34 | 0.22 | 0.0034 | 0.0342 | 2.56 |
| CDK8 | ENSG00000132964 | 49.79 | 0.28 | 0.0006 | 0.0342 | 2.52 |
| CDKN1B | ENSG00000111276 | 83.05 | 0.17 | 0.0161 | 0.0500 | 1.87 |
| CDKN2AIPNL | ENSG00000237190 | 15.15 | 0.19 | 0.0076 | 0.0382 | 1.88 |
| CDKN2B | ENSG00000147883 | 133.53 | 0.23 | 0.0029 | 0.0342 | 0.42 |
| CDS1 | ENSG00000163624 | 130.15 | 0.28 | 0.0003 | 0.0342 | 0.89 |
| CDS2 | ENSG00000101290 | 211.03 | 0.19 | 0.0102 | 0.0422 | 1.53 |
| CDSN | ENSG00000204539 | 24.26 | 0.21 | 0.0121 | 0.0445 | 1.60 |
| CDX1 | ENSG00000113722 | 248.75 | 0.24 | 0.0009 | 0.0342 | 1.00 |
| CDX2 | ENSG00000165556 | 205.80 | 0.20 | 0.0072 | 0.0377 | 2.12 |
| CDYL | ENSG00000153046 | 96.06 | 0.21 | 0.0039 | 0.0345 | 1.41 |
| CEACAM16 | ENSG00000213892 | 16.24 | 0.18 | 0.0151 | 0.0487 | 0.51 |
| CEACAM19 | ENSG00000186567 | 60.67 | 0.18 | 0.0142 | 0.0474 | 0.82 |
| CEACAM5 | ENSG00000105388 | 2754.94 | 0.18 | 0.0153 | 0.0489 | 2.06 |
| CEACAM6 | ENSG00000086548 | 536.99 | 0.21 | 0.0054 | 0.0362 | 5.27 |
| CEBPG | ENSG00000153879 | 147.34 | 0.23 | 0.0019 | 0.0342 | 1.77 |
| CECR5 | ENSG00000069998 | 54.09 | 0.21 | 0.0040 | 0.0346 | 1.61 |
| CELSR2 | ENSG00000143126 | 50.61 | 0.23 | 0.0013 | 0.0342 | 2.65 |
| CELSR3 | ENSG00000008300 | 48.22 | 0.26 | 0.0018 | 0.0342 | 5.20 |
| CENPB | ENSG00000125817 | 84.33 | 0.19 | 0.0117 | 0.0439 | 2.00 |
| CENPF | ENSG00000117724 | 138.38 | 0.19 | 0.0145 | 0.0479 | 4.57 |
| CENPV | ENSG00000166582 | 45.65 | 0.19 | 0.0111 | 0.0433 | 1.22 |
| CEP104 | ENSG00000116198 | 99.07 | 0.23 | 0.0021 | 0.0342 | 1.38 |
| CEP19 | ENSG00000174007 | 4.77 | 0.23 | 0.0070 | 0.0376 | 1.27 |
| CEP192 | ENSG00000101639 | 282.79 | 0.21 | 0.0025 | 0.0342 | 1.16 |
| CEP250 | ENSG00000126001 | 139.27 | 0.21 | 0.0057 | 0.0363 | 2.69 |
| CEP350 | ENSG00000135837 | 412.03 | 0.19 | 0.0073 | 0.0379 | 1.44 |
| CEP44 | ENSG00000164118 | 47.34 | 0.20 | 0.0099 | 0.0417 | 1.33 |
| CEP55 | ENSG00000138180 | 18.26 | 0.23 | 0.0100 | 0.0419 | 4.42 |
| CEP72 | ENSG00000112877 | 38.04 | 0.19 | 0.0101 | 0.0421 | 3.09 |
| CEP89 | ENSG00000121289 | 21.41 | 0.20 | 0.0064 | 0.0370 | 2.00 |
| CERK | ENSG00000100422 | 169.75 | 0.19 | 0.0102 | 0.0422 | 0.97 |
| CERS2 | ENSG00000143418 | 171.09 | 0.24 | 0.0011 | 0.0342 | 1.89 |
| CERS6 | ENSG00000172292 | 409.65 | 0.25 | 0.0021 | 0.0342 | 2.06 |
| CES2 | ENSG00000172831 | 622.56 | 0.23 | 0.0010 | 0.0342 | 0.67 |
| CES3 | ENSG00000172828 | 253.16 | 0.25 | 0.0015 | 0.0342 | 0.44 |
| CETN2 | ENSG00000147400 | 14.70 | 0.21 | 0.0049 | 0.0355 | 3.36 |
| CFDP1 | ENSG00000153774 | 44.42 | 0.18 | 0.0129 | 0.0457 | 1.91 |
| CFTR | ENSG00000001626 | 592.39 | 0.19 | 0.0098 | 0.0416 | 1.57 |
| CGGBP1 | ENSG00000163320 | 167.39 | 0.18 | 0.0156 | 0.0494 | 1.50 |
| CGN | ENSG00000143375 | 382.55 | 0.19 | 0.0126 | 0.0452 | 0.95 |
| CHAD | ENSG00000136457 | 38.16 | 0.24 | 0.0020 | 0.0342 | 0.64 |
| CHAF1A | ENSG00000167670 | 46.99 | 0.18 | 0.0138 | 0.0468 | 2.15 |
| CHCHD2 | ENSG00000106153 | 57.62 | 0.22 | 0.0030 | 0.0342 | 2.62 |
| CHCHD4 | ENSG00000163528 | 13.43 | 0.19 | 0.0105 | 0.0426 | 1.97 |
| CHD1L | ENSG00000131778 | 81.77 | 0.23 | 0.0024 | 0.0342 | 1.77 |
| CHD4 | ENSG00000111642 | 472.67 | 0.18 | 0.0130 | 0.0458 | 1.89 |
| CHD8 | ENSG00000100888 | 263.86 | 0.18 | 0.0122 | 0.0446 | 1.45 |
| CHDH | ENSG00000016391 | 107.62 | 0.25 | 0.0014 | 0.0342 | 1.93 |
| CHEK1 | ENSG00000149554 | 26.26 | 0.19 | 0.0135 | 0.0464 | 3.15 |
| CHEK2 | ENSG00000183765 | 21.59 | 0.24 | 0.0019 | 0.0342 | 2.08 |
| CHERP | ENSG00000085872 | 88.80 | 0.19 | 0.0071 | 0.0376 | 1.99 |
| CHID1 | ENSG00000177830 | 66.77 | 0.23 | 0.0017 | 0.0342 | 2.03 |
| CHKA | ENSG00000110721 | 149.69 | 0.20 | 0.0062 | 0.0368 | 0.83 |
| CHMP1A | ENSG00000131165 | 115.14 | 0.22 | 0.0057 | 0.0363 | 1.69 |
| CHMP3 | ENSG00000115561 | 239.87 | 0.18 | 0.0149 | 0.0485 | 1.43 |
| CHMP4B | ENSG00000101421 | 161.22 | 0.18 | 0.0140 | 0.0471 | 2.00 |
| CHMP4C | ENSG00000164695 | 25.02 | 0.24 | 0.0018 | 0.0342 | 2.51 |
| CHMP5 | ENSG00000086065 | 52.78 | 0.21 | 0.0047 | 0.0353 | 1.70 |
| CHPF | ENSG00000123989 | 73.94 | 0.21 | 0.0068 | 0.0374 | 2.93 |
| CHPF2 | ENSG00000033100 | 165.81 | 0.21 | 0.0044 | 0.0351 | 1.39 |
| CHPT1 | ENSG00000111666 | 82.62 | 0.23 | 0.0022 | 0.0342 | 1.52 |
| CHRNB1 | ENSG00000170175 | 11.69 | 0.25 | 0.0013 | 0.0342 | 1.63 |
| CHST5 | ENSG00000135702 | 220.47 | 0.24 | 0.0015 | 0.0342 | 0.17 |
| CHST6 | ENSG00000183196 | 36.39 | 0.21 | 0.0062 | 0.0368 | 0.52 |
| CHTF8 | ENSG00000168802 | 115.30 | 0.20 | 0.0054 | 0.0362 | 1.71 |
| CHTOP | ENSG00000160679 | 186.80 | 0.19 | 0.0081 | 0.0391 | 1.42 |
| CHUK | ENSG00000213341 | 65.50 | 0.19 | 0.0128 | 0.0456 | 1.52 |
| CHURC1-FNTB | ENSG00000125954 | 146.44 | 0.24 | 0.0009 | 0.0342 | 1.35 |
| CIAO1 | ENSG00000144021 | 125.42 | 0.20 | 0.0079 | 0.0387 | 1.70 |
| CIAPIN1 | ENSG00000005194 | 35.99 | 0.19 | 0.0093 | 0.0409 | 1.74 |
| CIB1 | ENSG00000185043 | 96.25 | 0.20 | 0.0068 | 0.0374 | 1.01 |
| CIC | ENSG00000079432 | 172.81 | 0.19 | 0.0128 | 0.0456 | 1.36 |
| CIR1 | ENSG00000138433 | 45.26 | 0.22 | 0.0021 | 0.0342 | 1.82 |
| CIRBP | ENSG00000099622 | 455.55 | 0.22 | 0.0035 | 0.0342 | 0.99 |
| CISD1 | ENSG00000122873 | 18.24 | 0.21 | 0.0034 | 0.0342 | 2.16 |
| CISD2 | ENSG00000145354 | 21.83 | 0.23 | 0.0061 | 0.0368 | 1.57 |
| CISD3 | ENSG00000230055 | 96.22 | 0.21 | 0.0054 | 0.0362 | 1.29 |
| CISH | ENSG00000114737 | 66.30 | 0.20 | 0.0090 | 0.0405 | 1.24 |
| CIZ1 | ENSG00000148337 | 136.42 | 0.19 | 0.0125 | 0.0451 | 1.93 |
| CKAP4 | ENSG00000136026 | 176.31 | 0.21 | 0.0048 | 0.0354 | 1.36 |
| CKAP5 | ENSG00000175216 | 206.06 | 0.20 | 0.0079 | 0.0387 | 2.76 |
| CKB | ENSG00000166165 | 539.33 | 0.21 | 0.0065 | 0.0371 | 0.69 |
| CKMT1A | ENSG00000223572 | 85.34 | 0.20 | 0.0083 | 0.0395 | 0.69 |
| CKMT1B | ENSG00000237289 | 124.21 | 0.19 | 0.0097 | 0.0415 | 0.65 |
| CKS1B | ENSG00000173207 | 9.54 | 0.19 | 0.0147 | 0.0482 | 2.30 |
| CLCC1 | ENSG00000121940 | 117.74 | 0.19 | 0.0086 | 0.0399 | 2.17 |
| CLCN3 | ENSG00000109572 | 360.64 | 0.25 | 0.0011 | 0.0342 | 1.30 |
| CLCN5 | ENSG00000171365 | 111.84 | 0.22 | 0.0021 | 0.0342 | 1.85 |
| CLCN7 | ENSG00000103249 | 121.99 | 0.19 | 0.0147 | 0.0482 | 2.10 |
| CLDN12 | ENSG00000157224 | 115.53 | 0.32 | 0.0003 | 0.0342 | 2.30 |
| CLDN7 | ENSG00000181885 | 451.36 | 0.21 | 0.0063 | 0.0370 | 0.67 |
| CLDN8 | ENSG00000156284 | 71.74 | 0.26 | 0.0009 | 0.0342 | 0.01 |
| CLEC16A | ENSG00000038532 | 181.54 | 0.17 | 0.0157 | 0.0496 | 1.29 |
| CLEC18B | ENSG00000140839 | 9.10 | 0.21 | 0.0132 | 0.0460 | 0.86 |
| CLEC4F | ENSG00000152672 | 7.12 | 0.24 | 0.0039 | 0.0345 | 0.41 |
| CLIC1 | ENSG00000213719 | 221.77 | 0.23 | 0.0022 | 0.0342 | 2.56 |
| CLIC3 | ENSG00000169583 | 2.95 | 0.21 | 0.0139 | 0.0469 | 2.97 |
| CLIC5 | ENSG00000112782 | 176.89 | 0.25 | 0.0015 | 0.0342 | 0.66 |
| CLINT1 | ENSG00000113282 | 327.87 | 0.19 | 0.0077 | 0.0384 | 1.04 |
| CLIP2 | ENSG00000106665 | 238.08 | 0.20 | 0.0111 | 0.0433 | 1.74 |
| CLK2 | ENSG00000176444 | 88.09 | 0.22 | 0.0021 | 0.0342 | 1.37 |
| CLLU1 | ENSG00000205056 | 23.62 | -0.25 | 0.0047 | 0.0353 | 0.31 |
| CLMN | ENSG00000165959 | 783.18 | 0.21 | 0.0042 | 0.0350 | 0.58 |
| CLN3 | ENSG00000188603 | 103.41 | 0.25 | 0.0003 | 0.0342 | 1.29 |
| CLN5 | ENSG00000102805 | 43.98 | 0.21 | 0.0037 | 0.0342 | 1.58 |
| CLN6 | ENSG00000128973 | 68.57 | 0.19 | 0.0125 | 0.0451 | 1.99 |
| CLNS1A | ENSG00000074201 | 115.86 | 0.25 | 0.0013 | 0.0342 | 2.25 |
| CLOCK | ENSG00000134852 | 135.86 | 0.26 | 0.0011 | 0.0342 | 1.62 |
| CLPTM1 | ENSG00000104853 | 101.15 | 0.23 | 0.0015 | 0.0342 | 1.43 |
| CLPTM1L | ENSG00000049656 | 187.20 | 0.21 | 0.0044 | 0.0351 | 1.50 |
| CLPX | ENSG00000166855 | 64.35 | 0.22 | 0.0021 | 0.0342 | 1.82 |
| CLRN3 | ENSG00000180745 | 35.02 | 0.23 | 0.0021 | 0.0342 | 1.31 |
| CLSTN1 | ENSG00000171603 | 506.31 | 0.23 | 0.0019 | 0.0342 | 1.06 |
| CLTA | ENSG00000122705 | 104.45 | 0.22 | 0.0028 | 0.0342 | 2.24 |
| CLTC | ENSG00000141367 | 793.53 | 0.21 | 0.0029 | 0.0342 | 1.65 |
| CLVS1 | ENSG00000177182 | 12.52 | 0.23 | 0.0031 | 0.0342 | 2.59 |
| CMAS | ENSG00000111726 | 69.03 | 0.21 | 0.0063 | 0.0370 | 1.61 |
| CMBL | ENSG00000164237 | 112.43 | 0.20 | 0.0083 | 0.0395 | 0.75 |
| CMIP | ENSG00000153815 | 299.77 | 0.19 | 0.0104 | 0.0424 | 1.25 |
| CMPK1 | ENSG00000162368 | 318.73 | 0.26 | 0.0004 | 0.0342 | 1.53 |
| CMTM2 | ENSG00000140932 | 5.71 | 0.20 | 0.0122 | 0.0446 | 1.20 |
| CMTM4 | ENSG00000183723 | 504.55 | 0.24 | 0.0011 | 0.0342 | 1.12 |
| CMTM6 | ENSG00000091317 | 156.30 | 0.22 | 0.0063 | 0.0370 | 2.14 |
| CNDP2 | ENSG00000133313 | 180.58 | 0.23 | 0.0016 | 0.0342 | 1.26 |
| CNIH | ENSG00000100528 | 42.99 | 0.23 | 0.0021 | 0.0342 | 2.00 |
| CNIH4 | ENSG00000143771 | 62.72 | 0.20 | 0.0067 | 0.0373 | 1.52 |
| CNKSR1 | ENSG00000142675 | 87.90 | 0.29 | <.0001 | 0.0342 | 1.28 |
| CNKSR3 | ENSG00000153721 | 72.82 | 0.21 | 0.0058 | 0.0364 | 0.99 |
| CNN2 | ENSG00000064666 | 143.71 | 0.20 | 0.0112 | 0.0434 | 3.01 |
| CNNM2 | ENSG00000148842 | 87.33 | 0.19 | 0.0107 | 0.0428 | 0.68 |
| CNNM3 | ENSG00000168763 | 85.86 | 0.18 | 0.0135 | 0.0464 | 1.65 |
| CNNM4 | ENSG00000158158 | 347.20 | 0.21 | 0.0042 | 0.0350 | 0.70 |
| CNOT1 | ENSG00000125107 | 833.44 | 0.21 | 0.0034 | 0.0342 | 1.86 |
| CNOT10 | ENSG00000182973 | 74.02 | 0.20 | 0.0034 | 0.0342 | 1.51 |
| CNOT3 | ENSG00000088038 | 133.53 | 0.20 | 0.0071 | 0.0376 | 1.20 |
| CNOT6 | ENSG00000113300 | 123.14 | 0.23 | 0.0017 | 0.0342 | 1.90 |
| CNOT8 | ENSG00000155508 | 111.30 | 0.23 | 0.0006 | 0.0342 | 1.46 |
| CNP | ENSG00000173786 | 86.98 | 0.19 | 0.0113 | 0.0435 | 1.76 |
| CNPPD1 | ENSG00000115649 | 97.75 | 0.23 | 0.0030 | 0.0342 | 1.41 |
| CNPY4 | ENSG00000166997 | 19.02 | 0.21 | 0.0068 | 0.0374 | 1.26 |
| CNTD1 | ENSG00000176563 | 64.26 | 0.19 | 0.0097 | 0.0415 | 1.44 |
| CNTROB | ENSG00000170037 | 67.57 | 0.19 | 0.0086 | 0.0399 | 1.37 |
| COASY | ENSG00000068120 | 70.71 | 0.21 | 0.0036 | 0.0342 | 1.90 |
| COBL | ENSG00000106078 | 201.57 | 0.25 | 0.0010 | 0.0342 | 1.35 |
| COBRA1 | ENSG00000188986 | 84.62 | 0.21 | 0.0062 | 0.0368 | 1.92 |
| COG1 | ENSG00000166685 | 105.61 | 0.21 | 0.0044 | 0.0351 | 1.36 |
| COG2 | ENSG00000135775 | 126.45 | 0.23 | 0.0016 | 0.0342 | 1.18 |
| COG3 | ENSG00000136152 | 160.38 | 0.25 | 0.0005 | 0.0342 | 1.70 |
| COG4 | ENSG00000103051 | 102.33 | 0.20 | 0.0049 | 0.0355 | 1.53 |
| COG5 | ENSG00000164597 | 224.31 | 0.19 | 0.0101 | 0.0421 | 1.53 |
| COG7 | ENSG00000168434 | 65.63 | 0.25 | 0.0008 | 0.0342 | 1.24 |
| COG8 | ENSG00000213380 | 84.26 | 0.22 | 0.0021 | 0.0342 | 1.77 |
| COIL | ENSG00000121058 | 28.53 | 0.21 | 0.0045 | 0.0352 | 2.06 |
| COL17A1 | ENSG00000065618 | 353.03 | 0.25 | 0.0009 | 0.0342 | 1.15 |
| COL4A3BP | ENSG00000113163 | 98.13 | 0.17 | 0.0144 | 0.0477 | 1.29 |
| COL9A2 | ENSG00000049089 | 89.34 | 0.27 | 0.0013 | 0.0342 | 0.81 |
| COMMD1 | ENSG00000173163 | 26.41 | 0.20 | 0.0082 | 0.0393 | 1.63 |
| COMMD2 | ENSG00000114744 | 34.84 | 0.21 | 0.0050 | 0.0356 | 1.85 |
| COMMD3 | ENSG00000148444 | 30.51 | 0.20 | 0.0096 | 0.0414 | 1.39 |
| COMMD4 | ENSG00000140365 | 26.04 | 0.22 | 0.0032 | 0.0342 | 1.50 |
| COMMD5 | ENSG00000170619 | 34.38 | 0.18 | 0.0160 | 0.0499 | 1.68 |
| COMMD6 | ENSG00000188243 | 22.47 | 0.21 | 0.0046 | 0.0352 | 3.10 |
| COMMD7 | ENSG00000149600 | 28.84 | 0.19 | 0.0115 | 0.0438 | 2.64 |
| COMT | ENSG00000093010 | 62.59 | 0.22 | 0.0015 | 0.0342 | 2.36 |
| COMTD1 | ENSG00000165644 | 22.18 | 0.21 | 0.0077 | 0.0384 | 1.72 |
| COPA | ENSG00000122218 | 387.71 | 0.20 | 0.0057 | 0.0363 | 1.62 |
| COPB1 | ENSG00000129083 | 161.26 | 0.20 | 0.0041 | 0.0349 | 1.67 |
| COPB2 | ENSG00000184432 | 244.15 | 0.20 | 0.0072 | 0.0377 | 1.59 |
| COPE | ENSG00000105669 | 70.08 | 0.22 | 0.0033 | 0.0342 | 1.57 |
| COPG | ENSG00000181789 | 327.53 | 0.20 | 0.0066 | 0.0373 | 1.26 |
| COPS2 | ENSG00000166200 | 66.19 | 0.18 | 0.0136 | 0.0466 | 2.09 |
| COPS5 | ENSG00000121022 | 111.48 | 0.19 | 0.0099 | 0.0417 | 1.60 |
| COPS7B | ENSG00000144524 | 69.52 | 0.23 | 0.0019 | 0.0342 | 1.67 |
| COPZ1 | ENSG00000111481 | 137.58 | 0.20 | 0.0068 | 0.0374 | 1.90 |
| COQ4 | ENSG00000167113 | 129.30 | 0.18 | 0.0161 | 0.0500 | 1.18 |
| COQ5 | ENSG00000110871 | 22.18 | 0.23 | 0.0018 | 0.0342 | 1.48 |
| COQ6 | ENSG00000119723 | 39.90 | 0.28 | 0.0003 | 0.0342 | 0.89 |
| COQ9 | ENSG00000088682 | 74.90 | 0.23 | 0.0009 | 0.0342 | 1.17 |
| CORO1B | ENSG00000172725 | 154.41 | 0.21 | 0.0075 | 0.0382 | 1.09 |
| CORO2A | ENSG00000106789 | 155.38 | 0.23 | 0.0025 | 0.0342 | 1.11 |
| COX11 | ENSG00000166260 | 51.47 | 0.23 | 0.0019 | 0.0342 | 1.61 |
| COX15 | ENSG00000014919 | 111.97 | 0.18 | 0.0132 | 0.0460 | 1.38 |
| COX17 | ENSG00000138495 | 23.19 | 0.23 | 0.0017 | 0.0342 | 1.45 |
| COX18 | ENSG00000163626 | 40.43 | 0.20 | 0.0077 | 0.0384 | 1.47 |
| COX19 | ENSG00000240230 | 145.71 | 0.18 | 0.0144 | 0.0477 | 1.97 |
| COX4I1 | ENSG00000131143 | 177.95 | 0.21 | 0.0035 | 0.0342 | 1.41 |
| COX5A | ENSG00000178741 | 107.53 | 0.20 | 0.0067 | 0.0373 | 1.36 |
| COX5B | ENSG00000135940 | 74.58 | 0.20 | 0.0059 | 0.0365 | 1.43 |
| COX6A1 | ENSG00000111775 | 77.17 | 0.19 | 0.0066 | 0.0373 | 1.47 |
| COX6B1 | ENSG00000126267 | 93.91 | 0.20 | 0.0062 | 0.0368 | 1.63 |
| COX6B2 | ENSG00000160471 | 39.32 | 0.23 | 0.0033 | 0.0342 | 0.26 |
| COX6C | ENSG00000164919 | 51.15 | 0.20 | 0.0067 | 0.0373 | 1.75 |
| COX7A2L | ENSG00000115944 | 94.63 | 0.20 | 0.0069 | 0.0375 | 1.53 |
| COX7C | ENSG00000127184 | 86.83 | 0.21 | 0.0039 | 0.0345 | 1.46 |
| COX8A | ENSG00000176340 | 110.11 | 0.22 | 0.0024 | 0.0342 | 1.16 |
| CPD | ENSG00000108582 | 259.75 | 0.26 | 0.0004 | 0.0342 | 2.12 |
| CPEB2 | ENSG00000137449 | 95.03 | 0.18 | 0.0161 | 0.0500 | 0.88 |
| CPNE2 | ENSG00000140848 | 95.56 | 0.24 | 0.0007 | 0.0342 | 1.11 |
| CPNE3 | ENSG00000085719 | 164.64 | 0.18 | 0.0133 | 0.0462 | 2.02 |
| CPOX | ENSG00000080819 | 37.78 | 0.22 | 0.0031 | 0.0342 | 1.83 |
| CPSF2 | ENSG00000165934 | 106.94 | 0.23 | 0.0032 | 0.0342 | 2.12 |
| CPT1A | ENSG00000110090 | 485.69 | 0.19 | 0.0107 | 0.0428 | 0.95 |
| CPT2 | ENSG00000157184 | 66.25 | 0.22 | 0.0035 | 0.0342 | 0.89 |
| CR2 | ENSG00000117322 | 35.21 | -0.20 | 0.0160 | 0.0499 | 0.19 |
| CRADD | ENSG00000169372 | 11.71 | 0.20 | 0.0056 | 0.0363 | 1.03 |
| CRAMP1L | ENSG00000007545 | 171.86 | 0.18 | 0.0127 | 0.0454 | 1.74 |
| CRAT | ENSG00000095321 | 76.84 | 0.24 | 0.0011 | 0.0342 | 1.20 |
| CRB3 | ENSG00000130545 | 32.62 | 0.18 | 0.0147 | 0.0482 | 1.08 |
| CRCP | ENSG00000241258 | 120.10 | 0.20 | 0.0068 | 0.0374 | 1.65 |
| CREB3 | ENSG00000107175 | 68.39 | 0.20 | 0.0063 | 0.0370 | 1.19 |
| CREB3L1 | ENSG00000157613 | 351.47 | 0.23 | 0.0019 | 0.0342 | 0.64 |
| CREB3L4 | ENSG00000143578 | 29.52 | 0.18 | 0.0131 | 0.0460 | 1.41 |
| CREBBP | ENSG00000005339 | 483.95 | 0.19 | 0.0108 | 0.0429 | 1.40 |
| CREBL2 | ENSG00000111269 | 81.90 | 0.20 | 0.0063 | 0.0370 | 1.50 |
| CREG1 | ENSG00000143162 | 77.25 | 0.20 | 0.0056 | 0.0363 | 2.25 |
| CRIP3 | ENSG00000146215 | 4.24 | -0.19 | 0.0138 | 0.0468 | 1.69 |
| CRIPAK | ENSG00000179979 | 53.99 | 0.26 | 0.0011 | 0.0342 | 0.99 |
| CRKL | ENSG00000099942 | 190.84 | 0.23 | 0.0011 | 0.0342 | 1.77 |
| CRYL1 | ENSG00000165475 | 48.12 | 0.21 | 0.0042 | 0.0350 | 1.65 |
| CRYZL1 | ENSG00000205758 | 55.98 | 0.18 | 0.0142 | 0.0474 | 1.09 |
| CS | ENSG00000062485 | 253.31 | 0.17 | 0.0154 | 0.0491 | 1.55 |
| CSDE1 | ENSG00000009307 | 737.70 | 0.23 | 0.0018 | 0.0342 | 1.76 |
| CSNK1G1 | ENSG00000169118 | 114.83 | 0.19 | 0.0076 | 0.0382 | 1.48 |
| CSNK1G3 | ENSG00000151292 | 119.03 | 0.19 | 0.0114 | 0.0437 | 1.43 |
| CSNK2A1 | ENSG00000101266 | 163.77 | 0.25 | 0.0011 | 0.0342 | 2.15 |
| CSNK2A2 | ENSG00000070770 | 41.20 | 0.20 | 0.0059 | 0.0365 | 2.88 |
| CSNK2B | ENSG00000204435 | 122.58 | 0.19 | 0.0096 | 0.0414 | 1.64 |
| CSRNP2 | ENSG00000110925 | 84.11 | 0.19 | 0.0137 | 0.0467 | 1.50 |
| CSRP2BP | ENSG00000149474 | 50.78 | 0.20 | 0.0072 | 0.0377 | 1.97 |
| CST3 | ENSG00000101439 | 260.58 | 0.19 | 0.0117 | 0.0439 | 1.13 |
| CSTF2 | ENSG00000101811 | 27.38 | 0.26 | 0.0037 | 0.0342 | 2.51 |
| CSTF3 | ENSG00000176102 | 54.02 | 0.20 | 0.0046 | 0.0352 | 1.80 |
| CTAGE5 | ENSG00000150527 | 148.29 | 0.26 | 0.0006 | 0.0342 | 0.87 |
| CTBP1 | ENSG00000159692 | 334.73 | 0.22 | 0.0035 | 0.0342 | 1.28 |
| CTBP2 | ENSG00000175029 | 277.48 | 0.19 | 0.0070 | 0.0376 | 1.41 |
| CTBS | ENSG00000117151 | 28.00 | 0.20 | 0.0066 | 0.0373 | 1.19 |
| CTC-203F4.1 | ENSG00000224186 | 68.92 | 0.19 | 0.0089 | 0.0403 | 1.68 |
| CTC-435M10.3 | ENSG00000255730 | 34.52 | 0.21 | 0.0039 | 0.0345 | 1.39 |
| CTCF | ENSG00000102974 | 113.05 | 0.19 | 0.0119 | 0.0442 | 1.70 |
| CTD-2330K9.3 | ENSG00000228008 | 5.81 | 0.19 | 0.0116 | 0.0439 | 1.02 |
| CTD-3074O7.11 | ENSG00000256349 | 66.47 | 0.25 | 0.0004 | 0.0342 | 1.38 |
| CTDNEP1 | ENSG00000175826 | 114.49 | 0.18 | 0.0141 | 0.0472 | 1.31 |
| CTDSP1 | ENSG00000144579 | 307.75 | 0.25 | 0.0013 | 0.0342 | 1.14 |
| CTDSP2 | ENSG00000175215 | 441.99 | 0.22 | 0.0018 | 0.0342 | 1.58 |
| CTDSPL | ENSG00000144677 | 162.39 | 0.22 | 0.0029 | 0.0342 | 1.56 |
| CTH | ENSG00000116761 | 9.99 | 0.18 | 0.0124 | 0.0450 | 1.19 |
| CTNNA1 | ENSG00000044115 | 604.42 | 0.18 | 0.0097 | 0.0415 | 1.44 |
| CTNNB1 | ENSG00000168036 | 653.64 | 0.18 | 0.0112 | 0.0434 | 2.29 |
| CTNNBIP1 | ENSG00000178585 | 41.28 | 0.18 | 0.0161 | 0.0500 | 1.76 |
| CTNND1 | ENSG00000198561 | 1641.15 | 0.23 | 0.0020 | 0.0342 | 1.06 |
| CTPS2 | ENSG00000047230 | 83.24 | 0.19 | 0.0099 | 0.0417 | 2.20 |
| CTR9 | ENSG00000198730 | 81.11 | 0.19 | 0.0088 | 0.0402 | 1.52 |
| CTSA | ENSG00000064601 | 260.94 | 0.20 | 0.0059 | 0.0365 | 1.68 |
| CTSB | ENSG00000164733 | 642.02 | 0.20 | 0.0085 | 0.0399 | 2.00 |
| CTSD | ENSG00000117984 | 695.52 | 0.23 | 0.0025 | 0.0342 | 1.11 |
| CTSE | ENSG00000196188 | 52.36 | 0.22 | 0.0071 | 0.0376 | 1.21 |
| CTSS | ENSG00000163131 | 341.86 | 0.26 | 0.0003 | 0.0342 | 0.83 |
| CTSZ | ENSG00000101160 | 233.48 | 0.18 | 0.0108 | 0.0429 | 1.49 |
| CTTN | ENSG00000085733 | 474.03 | 0.22 | 0.0015 | 0.0342 | 1.52 |
| CTTNBP2NL | ENSG00000143079 | 221.45 | 0.20 | 0.0064 | 0.0370 | 1.11 |
| CUL2 | ENSG00000108094 | 49.74 | 0.17 | 0.0155 | 0.0492 | 1.94 |
| CUL4A | ENSG00000139842 | 168.46 | 0.19 | 0.0085 | 0.0399 | 2.79 |
| CUL4B | ENSG00000158290 | 112.42 | 0.21 | 0.0047 | 0.0353 | 2.40 |
| CUL5 | ENSG00000166266 | 108.45 | 0.18 | 0.0125 | 0.0451 | 1.51 |
| CUL7 | ENSG00000044090 | 101.25 | 0.25 | 0.0009 | 0.0342 | 1.79 |
| CUL9 | ENSG00000112659 | 183.67 | 0.21 | 0.0076 | 0.0382 | 1.38 |
| CUTA | ENSG00000112514 | 38.95 | 0.19 | 0.0091 | 0.0407 | 2.12 |
| CWC27 | ENSG00000153015 | 37.64 | 0.19 | 0.0126 | 0.0452 | 1.77 |
| CXADR | ENSG00000154639 | 150.49 | 0.31 | <.0001 | 0.0342 | 1.99 |
| CXCL16 | ENSG00000161921 | 61.58 | 0.23 | 0.0029 | 0.0342 | 2.04 |
| CXorf23 | ENSG00000173681 | 114.45 | 0.23 | 0.0015 | 0.0342 | 1.19 |
| CXorf40A | ENSG00000197620 | 24.20 | 0.18 | 0.0148 | 0.0484 | 1.24 |
| CXXC1 | ENSG00000154832 | 62.89 | 0.19 | 0.0121 | 0.0445 | 0.90 |
| CXXC5 | ENSG00000171604 | 68.24 | 0.26 | 0.0003 | 0.0342 | 2.66 |
| CYB561 | ENSG00000008283 | 109.39 | 0.25 | 0.0008 | 0.0342 | 1.67 |
| CYB561D1 | ENSG00000174151 | 46.09 | 0.22 | 0.0027 | 0.0342 | 1.25 |
| CYB561D2 | ENSG00000114395 | 33.85 | 0.21 | 0.0032 | 0.0342 | 1.26 |
| CYB5B | ENSG00000103018 | 170.42 | 0.25 | 0.0010 | 0.0342 | 2.78 |
| CYB5R3 | ENSG00000100243 | 193.44 | 0.21 | 0.0042 | 0.0350 | 1.29 |
| CYB5RL | ENSG00000215883 | 33.78 | 0.21 | 0.0066 | 0.0373 | 1.65 |
| CYBA | ENSG00000051523 | 89.98 | 0.20 | 0.0060 | 0.0366 | 1.34 |
| CYC1 | ENSG00000179091 | 118.72 | 0.20 | 0.0059 | 0.0365 | 1.46 |
| CYFIP1 | ENSG00000068793 | 260.01 | 0.22 | 0.0013 | 0.0342 | 1.53 |
| CYHR1 | ENSG00000187954 | 132.28 | 0.26 | 0.0009 | 0.0342 | 1.74 |
| CYP2J2 | ENSG00000134716 | 49.66 | 0.29 | 0.0003 | 0.0342 | 1.24 |
| CYP2R1 | ENSG00000186104 | 54.18 | 0.22 | 0.0043 | 0.0351 | 1.12 |
| CYP2S1 | ENSG00000167600 | 77.42 | 0.29 | 0.0021 | 0.0342 | 3.62 |
| CYP4F3 | ENSG00000186529 | 29.31 | 0.20 | 0.0090 | 0.0405 | 3.30 |
| CYP4V2 | ENSG00000145476 | 144.25 | 0.22 | 0.0032 | 0.0342 | 0.89 |
| CYP51A1 | ENSG00000001630 | 113.10 | 0.20 | 0.0105 | 0.0426 | 2.50 |
| CYTH3 | ENSG00000008256 | 73.63 | 0.24 | 0.0009 | 0.0342 | 1.51 |
| CYYR1 | ENSG00000166265 | 17.24 | 0.21 | 0.0071 | 0.0376 | 0.88 |
| D2HGDH | ENSG00000180902 | 241.85 | 0.19 | 0.0132 | 0.0460 | 1.10 |
| DAB2IP | ENSG00000136848 | 364.09 | 0.25 | 0.0008 | 0.0342 | 1.14 |
| DACT1 | ENSG00000165617 | 34.41 | 0.19 | 0.0128 | 0.0456 | 1.95 |
| DAD1 | ENSG00000129562 | 40.49 | 0.23 | 0.0014 | 0.0342 | 1.83 |
| DAG1 | ENSG00000173402 | 252.23 | 0.21 | 0.0028 | 0.0342 | 1.66 |
| DAK | ENSG00000149476 | 82.46 | 0.23 | 0.0025 | 0.0342 | 1.47 |
| DALRD3 | ENSG00000178149 | 82.75 | 0.19 | 0.0066 | 0.0373 | 1.36 |
| DAP | ENSG00000112977 | 197.96 | 0.20 | 0.0080 | 0.0389 | 1.59 |
| DAPK2 | ENSG00000035664 | 54.37 | 0.21 | 0.0072 | 0.0377 | 1.23 |
| DARS | ENSG00000115866 | 65.63 | 0.19 | 0.0079 | 0.0387 | 3.02 |
| DARS2 | ENSG00000117593 | 66.49 | 0.20 | 0.0057 | 0.0363 | 1.83 |
| DAZAP2 | ENSG00000183283 | 314.66 | 0.21 | 0.0034 | 0.0342 | 1.25 |
| DBNDD1 | ENSG00000003249 | 11.58 | 0.22 | 0.0058 | 0.0364 | 3.94 |
| DBNDD2 | ENSG00000244274 | 93.58 | 0.19 | 0.0089 | 0.0403 | 1.57 |
| DCAF10 | ENSG00000122741 | 126.34 | 0.20 | 0.0096 | 0.0414 | 1.73 |
| DCAF11 | ENSG00000100897 | 163.40 | 0.24 | 0.0015 | 0.0342 | 1.09 |
| DCAF12 | ENSG00000198876 | 105.92 | 0.19 | 0.0103 | 0.0423 | 1.78 |
| DCAF17 | ENSG00000115827 | 64.34 | 0.19 | 0.0098 | 0.0416 | 1.71 |
| DCAF6 | ENSG00000143164 | 194.55 | 0.22 | 0.0024 | 0.0342 | 1.42 |
| DCAF7 | ENSG00000136485 | 275.61 | 0.20 | 0.0054 | 0.0362 | 1.88 |
| DCAKD | ENSG00000172992 | 52.33 | 0.23 | 0.0018 | 0.0342 | 1.64 |
| DCBLD1 | ENSG00000164465 | 70.31 | 0.20 | 0.0066 | 0.0373 | 1.65 |
| DCLRE1A | ENSG00000198924 | 25.63 | 0.23 | 0.0030 | 0.0342 | 2.52 |
| DCLRE1C | ENSG00000152457 | 69.53 | 0.19 | 0.0099 | 0.0417 | 1.80 |
| DCP1A | ENSG00000162290 | 109.47 | 0.18 | 0.0158 | 0.0497 | 1.72 |
| DCP1B | ENSG00000151065 | 27.60 | 0.20 | 0.0091 | 0.0407 | 1.74 |
| DCPS | ENSG00000110063 | 24.80 | 0.18 | 0.0123 | 0.0448 | 1.46 |
| DCST1 | ENSG00000163357 | 7.58 | 0.19 | 0.0120 | 0.0444 | 1.05 |
| DCTD | ENSG00000129187 | 64.62 | 0.22 | 0.0025 | 0.0342 | 2.04 |
| DCTN2 | ENSG00000175203 | 138.01 | 0.20 | 0.0046 | 0.0352 | 1.34 |
| DCTN3 | ENSG00000137100 | 31.65 | 0.21 | 0.0067 | 0.0373 | 1.55 |
| DCTN4 | ENSG00000132912 | 142.32 | 0.18 | 0.0160 | 0.0499 | 1.64 |
| DCTN5 | ENSG00000166847 | 119.06 | 0.23 | 0.0018 | 0.0342 | 1.85 |
| DCTPP1 | ENSG00000179958 | 32.02 | 0.19 | 0.0081 | 0.0391 | 2.24 |
| DCUN1D1 | ENSG00000043093 | 75.61 | 0.18 | 0.0153 | 0.0489 | 1.77 |
| DCUN1D2 | ENSG00000150401 | 52.13 | 0.19 | 0.0130 | 0.0458 | 1.83 |
| DCUN1D4 | ENSG00000109184 | 88.54 | 0.18 | 0.0142 | 0.0474 | 1.47 |
| DDA1 | ENSG00000130311 | 72.05 | 0.20 | 0.0082 | 0.0393 | 1.17 |
| DDAH1 | ENSG00000153904 | 274.89 | 0.22 | 0.0028 | 0.0342 | 1.62 |
| DDB1 | ENSG00000167986 | 295.20 | 0.19 | 0.0078 | 0.0386 | 1.83 |
| DDB2 | ENSG00000134574 | 34.64 | 0.20 | 0.0082 | 0.0393 | 1.47 |
| DDC | ENSG00000132437 | 39.75 | 0.20 | 0.0064 | 0.0370 | 1.96 |
| DDI2 | ENSG00000197312 | 220.79 | 0.21 | 0.0045 | 0.0352 | 1.72 |
| DDOST | ENSG00000244038 | 162.51 | 0.20 | 0.0060 | 0.0366 | 1.71 |
| DDR1 | ENSG00000204580 | 532.67 | 0.25 | 0.0007 | 0.0342 | 1.50 |
| DDRGK1 | ENSG00000198171 | 47.42 | 0.21 | 0.0039 | 0.0345 | 1.92 |
| DDT | ENSG00000099977 | 35.28 | 0.20 | 0.0047 | 0.0353 | 1.27 |
| DDTL | ENSG00000099974 | 49.21 | 0.21 | 0.0059 | 0.0365 | 1.13 |
| DDX1 | ENSG00000079785 | 98.22 | 0.22 | 0.0026 | 0.0342 | 2.21 |
| DDX11 | ENSG00000013573 | 67.15 | 0.20 | 0.0099 | 0.0417 | 2.22 |
| DDX17 | ENSG00000100201 | 2675.91 | 0.19 | 0.0092 | 0.0408 | 1.02 |
| DDX23 | ENSG00000174243 | 134.66 | 0.21 | 0.0047 | 0.0353 | 1.75 |
| DDX31 | ENSG00000125485 | 49.21 | 0.19 | 0.0089 | 0.0403 | 2.20 |
| DDX42 | ENSG00000198231 | 235.89 | 0.20 | 0.0063 | 0.0370 | 1.61 |
| DDX46 | ENSG00000145833 | 146.23 | 0.19 | 0.0103 | 0.0423 | 1.85 |
| DDX55 | ENSG00000111364 | 68.83 | 0.20 | 0.0065 | 0.0371 | 1.84 |
| DDX58 | ENSG00000107201 | 85.16 | 0.21 | 0.0124 | 0.0450 | 1.20 |
| DDX60 | ENSG00000137628 | 179.93 | 0.19 | 0.0155 | 0.0492 | 0.88 |
| DEAF1 | ENSG00000177030 | 54.65 | 0.20 | 0.0080 | 0.0389 | 1.55 |
| DECR1 | ENSG00000104325 | 84.95 | 0.20 | 0.0092 | 0.0408 | 1.32 |
| DECR2 | ENSG00000242612 | 56.09 | 0.24 | 0.0032 | 0.0342 | 1.38 |
| DEDD | ENSG00000158796 | 78.37 | 0.21 | 0.0049 | 0.0355 | 1.31 |
| DEGS2 | ENSG00000168350 | 18.98 | 0.23 | 0.0025 | 0.0342 | 1.31 |
| DEK | ENSG00000124795 | 190.63 | 0.20 | 0.0104 | 0.0424 | 3.30 |
| DENND1A | ENSG00000119522 | 182.25 | 0.21 | 0.0040 | 0.0346 | 1.39 |
| DENND1B | ENSG00000213047 | 160.30 | 0.20 | 0.0074 | 0.0380 | 0.94 |
| DENND2D | ENSG00000162777 | 172.68 | 0.20 | 0.0088 | 0.0402 | 0.93 |
| DENND4C | ENSG00000137145 | 299.39 | 0.25 | 0.0020 | 0.0342 | 1.81 |
| DERA | ENSG00000023697 | 49.17 | 0.23 | 0.0012 | 0.0342 | 1.27 |
| DERL1 | ENSG00000136986 | 153.29 | 0.19 | 0.0091 | 0.0407 | 1.50 |
| DFFB | ENSG00000169598 | 22.86 | 0.19 | 0.0103 | 0.0423 | 1.76 |
| DGAT1 | ENSG00000185000 | 150.38 | 0.22 | 0.0038 | 0.0342 | 1.21 |
| DGCR2 | ENSG00000070413 | 242.02 | 0.22 | 0.0034 | 0.0342 | 1.13 |
| DGCR8 | ENSG00000128191 | 163.18 | 0.19 | 0.0110 | 0.0432 | 1.39 |
| DGKE | ENSG00000153933 | 58.82 | 0.19 | 0.0102 | 0.0422 | 1.63 |
| DGKH | ENSG00000102780 | 129.04 | 0.22 | 0.0026 | 0.0342 | 2.21 |
| DGKQ | ENSG00000145214 | 177.78 | 0.23 | 0.0032 | 0.0342 | 1.13 |
| DHCR24 | ENSG00000116133 | 223.87 | 0.22 | 0.0030 | 0.0342 | 1.73 |
| DHFR | ENSG00000228716 | 47.57 | 0.19 | 0.0128 | 0.0456 | 1.94 |
| DHFRL1 | ENSG00000178700 | 25.85 | 0.25 | 0.0008 | 0.0342 | 1.40 |
| DHODH | ENSG00000102967 | 17.07 | 0.23 | 0.0028 | 0.0342 | 2.01 |
| DHPS | ENSG00000095059 | 39.58 | 0.18 | 0.0134 | 0.0463 | 1.66 |
| DHRS11 | ENSG00000108272 | 152.31 | 0.20 | 0.0064 | 0.0370 | 0.47 |
| DHRS13 | ENSG00000167536 | 11.89 | 0.21 | 0.0062 | 0.0368 | 1.63 |
| DHRS3 | ENSG00000162496 | 91.50 | 0.23 | 0.0014 | 0.0342 | 1.44 |
| DHRS7 | ENSG00000100612 | 39.10 | 0.21 | 0.0028 | 0.0342 | 1.30 |
| DHRSX | ENSG00000169084 | 26.39 | 0.20 | 0.0091 | 0.0407 | 1.69 |
| DHTKD1 | ENSG00000181192 | 100.16 | 0.21 | 0.0038 | 0.0342 | 1.37 |
| DHX16 | ENSG00000204560 | 123.45 | 0.19 | 0.0100 | 0.0419 | 1.18 |
| DHX29 | ENSG00000067248 | 68.97 | 0.20 | 0.0057 | 0.0363 | 1.67 |
| DHX30 | ENSG00000132153 | 126.03 | 0.18 | 0.0097 | 0.0415 | 1.74 |
| DHX32 | ENSG00000089876 | 109.55 | 0.23 | 0.0018 | 0.0342 | 1.25 |
| DHX34 | ENSG00000134815 | 59.91 | 0.19 | 0.0120 | 0.0444 | 2.37 |
| DHX35 | ENSG00000101452 | 50.25 | 0.18 | 0.0118 | 0.0441 | 1.85 |
| DHX38 | ENSG00000140829 | 117.93 | 0.19 | 0.0096 | 0.0414 | 1.60 |
| DHX9 | ENSG00000135829 | 195.18 | 0.19 | 0.0096 | 0.0414 | 2.19 |
| DIABLO | ENSG00000184047 | 61.70 | 0.22 | 0.0025 | 0.0342 | 1.58 |
| DICER1 | ENSG00000100697 | 406.76 | 0.18 | 0.0134 | 0.0463 | 1.19 |
| DIDO1 | ENSG00000101191 | 388.18 | 0.23 | 0.0018 | 0.0342 | 1.72 |
| DIP2A | ENSG00000160305 | 304.89 | 0.21 | 0.0045 | 0.0352 | 1.11 |
| DIP2B | ENSG00000066084 | 245.20 | 0.22 | 0.0026 | 0.0342 | 1.72 |
| DIRC2 | ENSG00000138463 | 48.78 | 0.24 | 0.0017 | 0.0342 | 1.01 |
| DIS3 | ENSG00000083520 | 104.13 | 0.19 | 0.0088 | 0.0402 | 2.67 |
| DKC1 | ENSG00000130826 | 73.54 | 0.19 | 0.0114 | 0.0437 | 3.88 |
| DLAT | ENSG00000150768 | 78.62 | 0.21 | 0.0054 | 0.0362 | 1.65 |
| DLEC1 | ENSG00000008226 | 70.41 | 0.20 | 0.0095 | 0.0413 | 0.96 |
| DLEU1 | ENSG00000176124 | 58.93 | 0.21 | 0.0065 | 0.0371 | 3.49 |
| DLG1 | ENSG00000075711 | 374.39 | 0.21 | 0.0035 | 0.0342 | 1.32 |
| DLG3 | ENSG00000082458 | 212.82 | 0.22 | 0.0032 | 0.0342 | 1.56 |
| DLG5 | ENSG00000151208 | 249.87 | 0.21 | 0.0040 | 0.0346 | 1.76 |
| DLL4 | ENSG00000128917 | 27.48 | 0.24 | 0.0013 | 0.0342 | 2.27 |
| DMXL2 | ENSG00000104093 | 489.31 | 0.19 | 0.0089 | 0.0403 | 0.72 |
| DNA2 | ENSG00000138346 | 21.28 | 0.23 | 0.0037 | 0.0342 | 2.80 |
| DNAAF2 | ENSG00000165506 | 24.62 | 0.21 | 0.0043 | 0.0351 | 1.38 |
| DNAJA1 | ENSG00000086061 | 101.62 | 0.20 | 0.0103 | 0.0423 | 2.86 |
| DNAJA3 | ENSG00000103423 | 71.82 | 0.21 | 0.0050 | 0.0356 | 2.37 |
| DNAJC11 | ENSG00000007923 | 170.87 | 0.21 | 0.0050 | 0.0356 | 1.18 |
| DNAJC13 | ENSG00000138246 | 279.06 | 0.22 | 0.0023 | 0.0342 | 1.40 |
| DNAJC16 | ENSG00000116138 | 127.61 | 0.22 | 0.0029 | 0.0342 | 1.29 |
| DNAJC19 | ENSG00000205981 | 27.34 | 0.23 | 0.0019 | 0.0342 | 1.72 |
| DNAJC21 | ENSG00000168724 | 107.54 | 0.18 | 0.0140 | 0.0471 | 1.95 |
| DNAJC22 | ENSG00000178401 | 34.83 | 0.30 | 0.0002 | 0.0342 | 1.13 |
| DNAJC25-GNG10 | ENSG00000244115 | 13.79 | 0.18 | 0.0142 | 0.0474 | 1.90 |
| DNAJC4 | ENSG00000110011 | 59.74 | 0.21 | 0.0062 | 0.0368 | 0.92 |
| DNAJC5 | ENSG00000101152 | 262.77 | 0.20 | 0.0076 | 0.0382 | 1.84 |
| DNAJC7 | ENSG00000168259 | 89.59 | 0.18 | 0.0097 | 0.0415 | 2.08 |
| DNAL1 | ENSG00000119661 | 36.35 | 0.18 | 0.0134 | 0.0463 | 1.49 |
| DNAL4 | ENSG00000100246 | 18.04 | 0.21 | 0.0082 | 0.0393 | 1.36 |
| DNASE1 | ENSG00000213918 | 47.24 | 0.22 | 0.0020 | 0.0342 | 2.09 |
| DNASE1L1 | ENSG00000013563 | 50.36 | 0.24 | 0.0013 | 0.0342 | 1.67 |
| DNASE2 | ENSG00000105612 | 23.21 | 0.22 | 0.0050 | 0.0356 | 2.19 |
| DNM2 | ENSG00000079805 | 934.15 | 0.23 | 0.0024 | 0.0342 | 0.96 |
| DNMBP | ENSG00000107554 | 323.33 | 0.20 | 0.0089 | 0.0403 | 1.24 |
| DNMT3A | ENSG00000119772 | 103.70 | 0.18 | 0.0160 | 0.0499 | 1.77 |
| DNMT3B | ENSG00000088305 | 10.57 | 0.19 | 0.0137 | 0.0467 | 3.59 |
| DNPEP | ENSG00000123992 | 101.00 | 0.20 | 0.0068 | 0.0374 | 1.84 |
| DOCK1 | ENSG00000150760 | 455.21 | 0.21 | 0.0040 | 0.0346 | 1.10 |
| DOCK5 | ENSG00000147459 | 577.86 | 0.22 | 0.0022 | 0.0342 | 0.85 |
| DOCK6 | ENSG00000130158 | 179.63 | 0.22 | 0.0036 | 0.0342 | 2.14 |
| DOCK7 | ENSG00000116641 | 163.71 | 0.19 | 0.0063 | 0.0370 | 1.38 |
| DOCK9 | ENSG00000088387 | 178.27 | 0.19 | 0.0097 | 0.0415 | 2.32 |
| DOK1 | ENSG00000115325 | 24.29 | 0.21 | 0.0084 | 0.0397 | 1.53 |
| DOK4 | ENSG00000125170 | 174.07 | 0.21 | 0.0053 | 0.0361 | 1.00 |
| DOLPP1 | ENSG00000167130 | 44.23 | 0.22 | 0.0045 | 0.0352 | 1.14 |
| DOPEY2 | ENSG00000142197 | 388.82 | 0.21 | 0.0036 | 0.0342 | 0.91 |
| DPAGT1 | ENSG00000172269 | 53.35 | 0.22 | 0.0032 | 0.0342 | 1.81 |
| DPF2 | ENSG00000133884 | 71.85 | 0.20 | 0.0061 | 0.0368 | 1.55 |
| DPH2 | ENSG00000132768 | 35.77 | 0.19 | 0.0112 | 0.0434 | 2.41 |
| DPM3 | ENSG00000179085 | 8.44 | 0.20 | 0.0077 | 0.0384 | 1.57 |
| DPP7 | ENSG00000176978 | 60.53 | 0.24 | 0.0026 | 0.0342 | 2.22 |
| DPP8 | ENSG00000074603 | 174.49 | 0.22 | 0.0030 | 0.0342 | 1.21 |
| DPP9 | ENSG00000142002 | 167.14 | 0.20 | 0.0078 | 0.0386 | 1.45 |
| DPY19L3 | ENSG00000178904 | 69.62 | 0.20 | 0.0092 | 0.0408 | 1.82 |
| DPY30 | ENSG00000162961 | 32.95 | 0.19 | 0.0074 | 0.0380 | 2.11 |
| DQX1 | ENSG00000144045 | 44.92 | 0.27 | 0.0002 | 0.0342 | 0.77 |
| DR1 | ENSG00000117505 | 94.39 | 0.22 | 0.0029 | 0.0342 | 1.62 |
| DRAM2 | ENSG00000156171 | 54.47 | 0.20 | 0.0047 | 0.0353 | 1.43 |
| DRAP1 | ENSG00000175550 | 23.57 | 0.18 | 0.0156 | 0.0494 | 1.90 |
| DRG1 | ENSG00000185721 | 33.93 | 0.20 | 0.0069 | 0.0375 | 1.71 |
| DROSHA | ENSG00000113360 | 144.97 | 0.23 | 0.0016 | 0.0342 | 1.92 |
| DSC2 | ENSG00000134755 | 381.59 | 0.18 | 0.0157 | 0.0496 | 0.86 |
| DSCR3 | ENSG00000157538 | 126.87 | 0.22 | 0.0023 | 0.0342 | 1.31 |
| DSG2 | ENSG00000046604 | 637.13 | 0.27 | 0.0004 | 0.0342 | 1.46 |
| DSN1 | ENSG00000149636 | 23.13 | 0.18 | 0.0138 | 0.0468 | 3.00 |
| DSP | ENSG00000096696 | 1252.51 | 0.22 | 0.0028 | 0.0342 | 1.59 |
| DST | ENSG00000151914 | 2333.81 | 0.20 | 0.0075 | 0.0382 | 0.93 |
| DSTN | ENSG00000125868 | 206.43 | 0.20 | 0.0080 | 0.0389 | 2.20 |
| DSTYK | ENSG00000133059 | 60.40 | 0.20 | 0.0068 | 0.0374 | 1.65 |
| DTNBP1 | ENSG00000047579 | 40.68 | 0.21 | 0.0050 | 0.0356 | 1.64 |
| DTWD1 | ENSG00000104047 | 26.64 | 0.20 | 0.0080 | 0.0389 | 1.39 |
| DTWD2 | ENSG00000169570 | 29.87 | 0.18 | 0.0157 | 0.0496 | 1.06 |
| DTX2 | ENSG00000091073 | 169.92 | 0.26 | 0.0008 | 0.0342 | 1.74 |
| DTX2 | ENSG00000250614 | 19.65 | 0.24 | 0.0026 | 0.0342 | 1.57 |
| DTX3L | ENSG00000163840 | 210.10 | 0.25 | 0.0036 | 0.0342 | 1.98 |
| DTX4 | ENSG00000110042 | 245.14 | 0.23 | 0.0026 | 0.0342 | 1.26 |
| DTYMK | ENSG00000168393 | 29.56 | 0.23 | 0.0025 | 0.0342 | 2.21 |
| DUOX1 | ENSG00000137857 | 58.22 | 0.22 | 0.0037 | 0.0342 | 1.16 |
| DUS1L | ENSG00000169718 | 121.78 | 0.21 | 0.0045 | 0.0352 | 2.00 |
| DUSP11 | ENSG00000144048 | 33.39 | 0.19 | 0.0101 | 0.0421 | 1.57 |
| DUSP16 | ENSG00000111266 | 175.26 | 0.18 | 0.0136 | 0.0466 | 1.62 |
| DUSP18 | ENSG00000167065 | 73.91 | 0.23 | 0.0029 | 0.0342 | 1.97 |
| DUSP3 | ENSG00000108861 | 152.01 | 0.21 | 0.0046 | 0.0352 | 1.55 |
| DVL1 | ENSG00000107404 | 117.28 | 0.21 | 0.0059 | 0.0365 | 1.77 |
| DVL2 | ENSG00000004975 | 50.97 | 0.20 | 0.0109 | 0.0431 | 1.56 |
| DYNC1H1 | ENSG00000197102 | 1359.96 | 0.23 | 0.0017 | 0.0342 | 1.59 |
| DYNC1I2 | ENSG00000077380 | 80.87 | 0.20 | 0.0071 | 0.0376 | 1.87 |
| DYNC1LI2 | ENSG00000135720 | 364.30 | 0.24 | 0.0014 | 0.0342 | 1.25 |
| DYNLL1 | ENSG00000088986 | 69.20 | 0.19 | 0.0093 | 0.0409 | 1.94 |
| DYNLL2 | ENSG00000121083 | 83.02 | 0.19 | 0.0095 | 0.0413 | 1.61 |
| DYNLRB1 | ENSG00000125971 | 81.44 | 0.22 | 0.0026 | 0.0342 | 2.33 |
| DYRK2 | ENSG00000127334 | 393.18 | 0.21 | 0.0056 | 0.0363 | 1.16 |
| DYX1C1-CCPG1 | ENSG00000256061 | 137.16 | 0.21 | 0.0034 | 0.0342 | 1.13 |
| E2F4 | ENSG00000205250 | 108.91 | 0.18 | 0.0152 | 0.0489 | 1.76 |
| EAF1 | ENSG00000144597 | 76.65 | 0.24 | 0.0019 | 0.0342 | 1.96 |
| EAPP | ENSG00000129518 | 33.79 | 0.18 | 0.0116 | 0.0439 | 1.18 |
| EBAG9 | ENSG00000147654 | 39.89 | 0.19 | 0.0119 | 0.0442 | 1.85 |
| EBNA1BP2 | ENSG00000117395 | 33.83 | 0.20 | 0.0088 | 0.0402 | 2.70 |
| EBP | ENSG00000147155 | 44.30 | 0.22 | 0.0033 | 0.0342 | 2.19 |
| EBPL | ENSG00000123179 | 14.44 | 0.25 | 0.0010 | 0.0342 | 3.61 |
| ECH1 | ENSG00000104823 | 114.18 | 0.18 | 0.0088 | 0.0402 | 1.03 |
| ECHDC2 | ENSG00000121310 | 417.57 | 0.21 | 0.0044 | 0.0351 | 0.84 |
| ECHS1 | ENSG00000127884 | 72.54 | 0.22 | 0.0019 | 0.0342 | 1.48 |
| ECI1 | ENSG00000167969 | 35.17 | 0.20 | 0.0060 | 0.0366 | 1.26 |
| ECI2 | ENSG00000198721 | 49.79 | 0.18 | 0.0133 | 0.0462 | 0.73 |
| ECSIT | ENSG00000130159 | 37.90 | 0.17 | 0.0150 | 0.0486 | 1.40 |
| ECT2 | ENSG00000114346 | 64.62 | 0.20 | 0.0131 | 0.0460 | 5.20 |
| EDC3 | ENSG00000179151 | 81.74 | 0.19 | 0.0095 | 0.0413 | 1.26 |
| EDEM2 | ENSG00000088298 | 54.17 | 0.20 | 0.0055 | 0.0363 | 1.82 |
| EDEM3 | ENSG00000116406 | 311.65 | 0.25 | 0.0013 | 0.0342 | 1.13 |
| EDF1 | ENSG00000107223 | 148.72 | 0.20 | 0.0059 | 0.0365 | 1.41 |
| EDN1 | ENSG00000078401 | 28.41 | 0.19 | 0.0106 | 0.0427 | 2.63 |
| EDN3 | ENSG00000124205 | 76.09 | 0.24 | 0.0010 | 0.0342 | 0.20 |
| EEA1 | ENSG00000102189 | 166.93 | 0.18 | 0.0108 | 0.0429 | 2.21 |
| EEF2 | ENSG00000167658 | 2251.95 | 0.23 | 0.0026 | 0.0342 | 2.13 |
| EEF2K | ENSG00000103319 | 157.08 | 0.24 | 0.0014 | 0.0342 | 1.25 |
| EFCAB4A | ENSG00000177685 | 156.61 | 0.23 | 0.0029 | 0.0342 | 1.12 |
| EFHA1 | ENSG00000165487 | 53.16 | 0.19 | 0.0099 | 0.0417 | 1.87 |
| EFHD2 | ENSG00000142634 | 225.65 | 0.19 | 0.0093 | 0.0409 | 1.23 |
| EFNA4 | ENSG00000243364 | 18.74 | 0.24 | 0.0022 | 0.0342 | 2.30 |
| EFNB1 | ENSG00000090776 | 132.01 | 0.20 | 0.0076 | 0.0382 | 1.80 |
| EFR3A | ENSG00000132294 | 131.51 | 0.24 | 0.0030 | 0.0342 | 2.12 |
| EFTUD1 | ENSG00000140598 | 65.10 | 0.23 | 0.0018 | 0.0342 | 1.38 |
| EGFR | ENSG00000146648 | 388.81 | 0.22 | 0.0023 | 0.0342 | 1.14 |
| EGLN1 | ENSG00000135766 | 121.90 | 0.25 | 0.0013 | 0.0342 | 1.26 |
| EGLN3 | ENSG00000129521 | 66.20 | 0.30 | 0.0007 | 0.0342 | 1.45 |
| EHF | ENSG00000135373 | 581.81 | 0.25 | 0.0009 | 0.0342 | 1.61 |
| EHHADH | ENSG00000113790 | 50.48 | 0.18 | 0.0133 | 0.0462 | 1.16 |
| EHMT2 | ENSG00000204371 | 128.11 | 0.25 | 0.0007 | 0.0342 | 1.87 |
| EI24 | ENSG00000149547 | 113.75 | 0.23 | 0.0015 | 0.0342 | 1.99 |
| EID1 | ENSG00000255302 | 65.83 | 0.17 | 0.0160 | 0.0499 | 1.49 |
| EIF1AX | ENSG00000173674 | 60.71 | 0.23 | 0.0060 | 0.0366 | 2.83 |
| EIF2AK1 | ENSG00000086232 | 244.98 | 0.23 | 0.0026 | 0.0342 | 2.13 |
| EIF2AK2 | ENSG00000055332 | 313.38 | 0.20 | 0.0113 | 0.0435 | 2.03 |
| EIF2B4 | ENSG00000115211 | 62.55 | 0.18 | 0.0122 | 0.0446 | 1.48 |
| EIF2B5 | ENSG00000145191 | 102.74 | 0.19 | 0.0070 | 0.0376 | 1.55 |
| EIF2C4 | ENSG00000134698 | 110.44 | 0.24 | 0.0005 | 0.0342 | 1.46 |
| EIF2S1 | ENSG00000134001 | 86.73 | 0.19 | 0.0154 | 0.0491 | 1.84 |
| EIF2S3 | ENSG00000130741 | 231.59 | 0.23 | 0.0034 | 0.0342 | 3.14 |
| EIF3A | ENSG00000107581 | 493.57 | 0.20 | 0.0072 | 0.0377 | 1.88 |
| EIF3D | ENSG00000100353 | 168.31 | 0.20 | 0.0062 | 0.0368 | 2.09 |
| EIF3E | ENSG00000104408 | 215.78 | 0.19 | 0.0107 | 0.0428 | 3.50 |
| EIF3G | ENSG00000130811 | 87.82 | 0.20 | 0.0070 | 0.0376 | 2.30 |
| EIF3H | ENSG00000147677 | 182.97 | 0.22 | 0.0031 | 0.0342 | 3.00 |
| EIF3K | ENSG00000178982 | 109.65 | 0.18 | 0.0161 | 0.0500 | 1.62 |
| EIF3M | ENSG00000149100 | 112.77 | 0.21 | 0.0052 | 0.0359 | 2.33 |
| EIF4A2 | ENSG00000156976 | 636.07 | 0.19 | 0.0103 | 0.0423 | 1.66 |
| EIF4B | ENSG00000063046 | 428.88 | 0.20 | 0.0087 | 0.0401 | 2.14 |
| EIF4E3 | ENSG00000163412 | 126.56 | 0.22 | 0.0037 | 0.0342 | 0.74 |
| EIF4EBP2 | ENSG00000148730 | 473.59 | 0.21 | 0.0036 | 0.0342 | 1.52 |
| EIF4ENIF1 | ENSG00000184708 | 90.79 | 0.20 | 0.0058 | 0.0364 | 1.17 |
| EIF4G2 | ENSG00000110321 | 1474.80 | 0.21 | 0.0042 | 0.0350 | 1.81 |
| EIF4H | ENSG00000106682 | 206.39 | 0.18 | 0.0130 | 0.0458 | 1.91 |
| EIF6 | ENSG00000242372 | 81.57 | 0.18 | 0.0133 | 0.0462 | 2.88 |
| ELAC1 | ENSG00000141642 | 12.54 | 0.20 | 0.0072 | 0.0377 | 0.92 |
| ELAC2 | ENSG00000006744 | 100.19 | 0.18 | 0.0111 | 0.0433 | 1.45 |
| ELF3 | ENSG00000163435 | 974.27 | 0.19 | 0.0116 | 0.0439 | 1.30 |
| ELK1 | ENSG00000126767 | 55.61 | 0.22 | 0.0033 | 0.0342 | 2.33 |
| ELMO2 | ENSG00000062598 | 72.57 | 0.18 | 0.0139 | 0.0469 | 2.11 |
| ELMO3 | ENSG00000102890 | 65.98 | 0.24 | 0.0010 | 0.0342 | 1.51 |
| ELMOD3 | ENSG00000115459 | 92.78 | 0.21 | 0.0037 | 0.0342 | 1.09 |
| ELOF1 | ENSG00000130165 | 27.24 | 0.18 | 0.0142 | 0.0474 | 1.74 |
| ELOVL1 | ENSG00000066322 | 80.05 | 0.21 | 0.0045 | 0.0352 | 1.58 |
| ELOVL7 | ENSG00000164181 | 35.36 | 0.18 | 0.0161 | 0.0500 | 1.75 |
| ELP3 | ENSG00000134014 | 55.98 | 0.25 | 0.0004 | 0.0342 | 1.45 |
| EME2 | ENSG00000197774 | 23.51 | 0.21 | 0.0088 | 0.0402 | 1.66 |
| EMILIN3 | ENSG00000183798 | 51.41 | 0.28 | 0.0003 | 0.0342 | 1.31 |
| EML2 | ENSG00000125746 | 74.91 | 0.21 | 0.0032 | 0.0342 | 1.25 |
| EMP2 | ENSG00000213853 | 328.81 | 0.21 | 0.0034 | 0.0342 | 1.38 |
| ENC1 | ENSG00000171617 | 222.23 | 0.23 | 0.0058 | 0.0364 | 3.17 |
| ENDOD1 | ENSG00000149218 | 219.77 | 0.21 | 0.0037 | 0.0342 | 0.65 |
| ENDOG | ENSG00000167136 | 21.80 | 0.20 | 0.0069 | 0.0375 | 1.66 |
| ENDOV | ENSG00000173818 | 60.72 | 0.19 | 0.0107 | 0.0428 | 0.88 |
| ENGASE | ENSG00000167280 | 254.09 | 0.22 | 0.0022 | 0.0342 | 2.34 |
| ENO1 | ENSG00000074800 | 356.14 | 0.22 | 0.0049 | 0.0355 | 2.96 |
| ENO4 | ENSG00000188316 | 11.29 | 0.21 | 0.0075 | 0.0382 | 1.21 |
| ENOSF1 | ENSG00000132199 | 175.81 | 0.18 | 0.0129 | 0.0457 | 0.90 |
| ENPP1 | ENSG00000197594 | 40.09 | 0.19 | 0.0144 | 0.0477 | 1.30 |
| ENPP3 | ENSG00000154269 | 32.12 | 0.22 | 0.0033 | 0.0342 | 1.20 |
| ENPP4 | ENSG00000001561 | 55.07 | 0.22 | 0.0034 | 0.0342 | 1.38 |
| ENPP5 | ENSG00000112796 | 7.66 | 0.18 | 0.0158 | 0.0497 | 2.18 |
| ENTPD2 | ENSG00000054179 | 22.83 | 0.22 | 0.0036 | 0.0342 | 2.86 |
| ENTPD5 | ENSG00000187097 | 140.66 | 0.23 | 0.0005 | 0.0342 | 0.51 |
| ENTPD6 | ENSG00000197586 | 348.49 | 0.22 | 0.0034 | 0.0342 | 1.79 |
| ENTPD8 | ENSG00000188833 | 111.21 | 0.23 | 0.0041 | 0.0349 | 0.47 |
| EP300 | ENSG00000100393 | 606.42 | 0.19 | 0.0086 | 0.0399 | 1.19 |
| EP400 | ENSG00000183495 | 374.62 | 0.19 | 0.0094 | 0.0411 | 1.67 |
| EPAS1 | ENSG00000116016 | 493.58 | 0.19 | 0.0117 | 0.0439 | 0.89 |
| EPB41L1 | ENSG00000088367 | 159.30 | 0.21 | 0.0036 | 0.0342 | 2.70 |
| EPB41L2 | ENSG00000079819 | 377.75 | 0.20 | 0.0111 | 0.0433 | 2.61 |
| EPB41L4A | ENSG00000129595 | 109.12 | 0.19 | 0.0079 | 0.0387 | 0.61 |
| EPB41L4B | ENSG00000095203 | 276.10 | 0.26 | 0.0005 | 0.0342 | 0.98 |
| EPB49 | ENSG00000158856 | 62.48 | 0.23 | 0.0020 | 0.0342 | 1.46 |
| EPCAM | ENSG00000119888 | 595.40 | 0.23 | 0.0018 | 0.0342 | 1.60 |
| EPG5 | ENSG00000152223 | 211.51 | 0.21 | 0.0034 | 0.0342 | 1.21 |
| EPHA10 | ENSG00000183317 | 201.24 | 0.22 | 0.0035 | 0.0342 | 0.51 |
| EPHB2 | ENSG00000133216 | 264.42 | 0.19 | 0.0123 | 0.0448 | 2.37 |
| EPHX1 | ENSG00000143819 | 103.86 | 0.22 | 0.0037 | 0.0342 | 1.44 |
| EPHX2 | ENSG00000120915 | 141.26 | 0.24 | 0.0008 | 0.0342 | 0.62 |
| EPN2 | ENSG00000072134 | 118.93 | 0.19 | 0.0107 | 0.0428 | 1.23 |
| EPN3 | ENSG00000049283 | 66.41 | 0.24 | 0.0023 | 0.0342 | 0.88 |
| EPOR | ENSG00000187266 | 20.32 | 0.20 | 0.0099 | 0.0417 | 0.98 |
| EPPK1 | ENSG00000227184 | 442.28 | 0.25 | 0.0019 | 0.0342 | 2.62 |
| EPS15L1 | ENSG00000127527 | 67.30 | 0.17 | 0.0158 | 0.0497 | 1.38 |
| EPS8 | ENSG00000151491 | 333.66 | 0.20 | 0.0050 | 0.0356 | 1.46 |
| EPS8L2 | ENSG00000177106 | 356.53 | 0.23 | 0.0028 | 0.0342 | 1.24 |
| EPS8L3 | ENSG00000198758 | 421.65 | 0.21 | 0.0052 | 0.0359 | 0.96 |
| ERAP1 | ENSG00000164307 | 390.93 | 0.20 | 0.0058 | 0.0364 | 1.07 |
| ERAP2 | ENSG00000164308 | 119.70 | 0.23 | 0.0058 | 0.0364 | 1.29 |
| ERBB2 | ENSG00000141736 | 469.77 | 0.26 | 0.0009 | 0.0342 | 1.32 |
| ERBB3 | ENSG00000065361 | 619.28 | 0.29 | <.0001 | 0.0342 | 2.00 |
| ERCC5 | ENSG00000134899 | 231.03 | 0.22 | 0.0024 | 0.0342 | 1.62 |
| ERGIC1 | ENSG00000113719 | 594.32 | 0.21 | 0.0037 | 0.0342 | 1.06 |
| ERGIC2 | ENSG00000087502 | 46.64 | 0.23 | 0.0029 | 0.0342 | 1.96 |
| ERGIC3 | ENSG00000125991 | 172.52 | 0.23 | 0.0016 | 0.0342 | 2.52 |
| ERI1 | ENSG00000104626 | 33.58 | 0.19 | 0.0144 | 0.0477 | 1.89 |
| ERI2 | ENSG00000196678 | 46.08 | 0.20 | 0.0054 | 0.0362 | 1.53 |
| ERLEC1 | ENSG00000068912 | 88.43 | 0.22 | 0.0035 | 0.0342 | 1.64 |
| ERLIN1 | ENSG00000107566 | 124.75 | 0.24 | 0.0005 | 0.0342 | 1.40 |
| ERLIN2 | ENSG00000147475 | 123.37 | 0.25 | 0.0008 | 0.0342 | 1.50 |
| ERMP1 | ENSG00000099219 | 200.86 | 0.25 | 0.0004 | 0.0342 | 1.32 |
| ERN2 | ENSG00000134398 | 403.07 | 0.25 | 0.0008 | 0.0342 | 0.67 |
| ERO1L | ENSG00000197930 | 165.73 | 0.25 | 0.0029 | 0.0342 | 2.82 |
| ERP29 | ENSG00000089248 | 101.06 | 0.21 | 0.0024 | 0.0342 | 1.61 |
| ERP44 | ENSG00000023318 | 114.78 | 0.19 | 0.0104 | 0.0424 | 1.42 |
| ESD | ENSG00000139684 | 43.91 | 0.21 | 0.0025 | 0.0342 | 2.42 |
| ESM1 | ENSG00000164283 | 2.03 | 0.23 | 0.0087 | 0.0401 | 9.01 |
| ESPL1 | ENSG00000135476 | 32.36 | 0.20 | 0.0130 | 0.0458 | 3.09 |
| ESRP1 | ENSG00000104413 | 292.47 | 0.24 | 0.0011 | 0.0342 | 1.64 |
| ESRP2 | ENSG00000103067 | 384.75 | 0.23 | 0.0025 | 0.0342 | 1.20 |
| ESRRA | ENSG00000173153 | 178.82 | 0.19 | 0.0113 | 0.0435 | 1.17 |
| ETAA1 | ENSG00000143971 | 31.78 | 0.20 | 0.0084 | 0.0397 | 1.82 |
| ETFA | ENSG00000140374 | 90.33 | 0.20 | 0.0056 | 0.0363 | 1.28 |
| ETFB | ENSG00000105379 | 42.41 | 0.18 | 0.0117 | 0.0439 | 1.50 |
| ETFDH | ENSG00000171503 | 79.16 | 0.18 | 0.0138 | 0.0468 | 0.84 |
| ETHE1 | ENSG00000105755 | 149.22 | 0.21 | 0.0050 | 0.0356 | 0.64 |
| ETV6 | ENSG00000139083 | 127.31 | 0.19 | 0.0111 | 0.0433 | 1.35 |
| EVI5 | ENSG00000067208 | 153.47 | 0.20 | 0.0060 | 0.0366 | 1.29 |
| EVPL | ENSG00000167880 | 107.97 | 0.27 | 0.0009 | 0.0342 | 4.31 |
| EXD2 | ENSG00000081177 | 52.38 | 0.20 | 0.0039 | 0.0345 | 1.26 |
| EXD3 | ENSG00000187609 | 98.50 | 0.22 | 0.0052 | 0.0359 | 1.00 |
| EXOC1 | ENSG00000090989 | 95.23 | 0.24 | 0.0002 | 0.0342 | 1.55 |
| EXOC2 | ENSG00000112685 | 82.11 | 0.18 | 0.0120 | 0.0444 | 1.55 |
| EXOC3L4 | ENSG00000205436 | 34.93 | 0.23 | 0.0035 | 0.0342 | 0.87 |
| EXOC5 | ENSG00000070367 | 89.07 | 0.19 | 0.0108 | 0.0429 | 1.89 |
| EXOC7 | ENSG00000182473 | 352.95 | 0.20 | 0.0073 | 0.0379 | 1.35 |
| EXOG | ENSG00000157036 | 49.73 | 0.19 | 0.0129 | 0.0457 | 1.20 |
| EXOSC10 | ENSG00000171824 | 120.32 | 0.17 | 0.0156 | 0.0494 | 1.48 |
| EXOSC4 | ENSG00000178896 | 10.05 | 0.21 | 0.0103 | 0.0423 | 2.62 |
| EXOSC6 | ENSG00000223496 | 33.04 | 0.21 | 0.0048 | 0.0354 | 1.79 |
| EXPH5 | ENSG00000110723 | 301.75 | 0.25 | 0.0010 | 0.0342 | 1.04 |
| EXT2 | ENSG00000151348 | 122.93 | 0.18 | 0.0152 | 0.0489 | 1.59 |
| EYA3 | ENSG00000158161 | 129.60 | 0.18 | 0.0150 | 0.0486 | 1.48 |
| EZH1 | ENSG00000108799 | 144.25 | 0.20 | 0.0059 | 0.0365 | 1.29 |
| F11R | ENSG00000158769 | 395.62 | 0.21 | 0.0035 | 0.0342 | 1.45 |
| F12 | ENSG00000131187 | 12.70 | 0.21 | 0.0062 | 0.0368 | 1.86 |
| F2R | ENSG00000181104 | 40.06 | 0.18 | 0.0149 | 0.0485 | 3.18 |
| F8A1 | ENSG00000197932 | 18.24 | 0.23 | 0.0037 | 0.0342 | 1.43 |
| FA2H | ENSG00000103089 | 63.42 | 0.19 | 0.0154 | 0.0491 | 1.18 |
| FAAH | ENSG00000117480 | 85.45 | 0.19 | 0.0093 | 0.0409 | 1.24 |
| FADD | ENSG00000168040 | 22.23 | 0.23 | 0.0015 | 0.0342 | 1.79 |
| FAF2 | ENSG00000113194 | 129.20 | 0.21 | 0.0046 | 0.0352 | 1.65 |
| FAHD1 | ENSG00000180185 | 30.06 | 0.19 | 0.0091 | 0.0407 | 1.44 |
| FAHD2A | ENSG00000115042 | 41.37 | 0.18 | 0.0153 | 0.0489 | 1.39 |
| FAM100B | ENSG00000185262 | 60.94 | 0.22 | 0.0049 | 0.0355 | 1.64 |
| FAM101A | ENSG00000178882 | 72.81 | 0.22 | 0.0025 | 0.0342 | 0.77 |
| FAM102B | ENSG00000162636 | 181.47 | 0.24 | 0.0014 | 0.0342 | 1.11 |
| FAM105A | ENSG00000145569 | 115.57 | 0.22 | 0.0026 | 0.0342 | 1.68 |
| FAM108C1 | ENSG00000136379 | 140.91 | 0.22 | 0.0040 | 0.0346 | 1.32 |
| FAM109A | ENSG00000198324 | 63.59 | 0.21 | 0.0043 | 0.0351 | 1.64 |
| FAM109B | ENSG00000177096 | 31.15 | 0.19 | 0.0097 | 0.0415 | 1.26 |
| FAM110C | ENSG00000184731 | 73.94 | 0.21 | 0.0088 | 0.0402 | 1.25 |
| FAM111A | ENSG00000166801 | 248.11 | 0.21 | 0.0069 | 0.0375 | 1.28 |
| FAM114A1 | ENSG00000197712 | 147.98 | 0.24 | 0.0008 | 0.0342 | 0.95 |
| FAM115A | ENSG00000198420 | 79.30 | 0.23 | 0.0024 | 0.0342 | 1.75 |
| FAM116A | ENSG00000174839 | 64.13 | 0.18 | 0.0130 | 0.0458 | 1.75 |
| FAM117B | ENSG00000138439 | 91.41 | 0.20 | 0.0067 | 0.0373 | 1.65 |
| FAM118B | ENSG00000197798 | 81.70 | 0.19 | 0.0098 | 0.0416 | 0.91 |
| FAM120A | ENSG00000048828 | 672.16 | 0.24 | 0.0007 | 0.0342 | 1.50 |
| FAM120AOS | ENSG00000188938 | 158.82 | 0.21 | 0.0043 | 0.0351 | 1.16 |
| FAM122B | ENSG00000156504 | 84.59 | 0.20 | 0.0077 | 0.0384 | 2.30 |
| FAM125A | ENSG00000141971 | 48.02 | 0.25 | 0.0012 | 0.0342 | 1.52 |
| FAM129B | ENSG00000136830 | 377.37 | 0.19 | 0.0091 | 0.0407 | 1.58 |
| FAM132A | ENSG00000184163 | 10.18 | 0.27 | 0.0034 | 0.0342 | 0.70 |
| FAM134A | ENSG00000144567 | 147.05 | 0.23 | 0.0026 | 0.0342 | 1.42 |
| FAM134C | ENSG00000141699 | 148.47 | 0.23 | 0.0009 | 0.0342 | 1.54 |
| FAM135A | ENSG00000082269 | 251.09 | 0.23 | 0.0013 | 0.0342 | 1.16 |
| FAM136A | ENSG00000035141 | 68.56 | 0.19 | 0.0080 | 0.0389 | 2.20 |
| FAM13A | ENSG00000138640 | 230.75 | 0.20 | 0.0079 | 0.0387 | 1.30 |
| FAM158A | ENSG00000100908 | 12.77 | 0.19 | 0.0126 | 0.0452 | 1.21 |
| FAM160A1 | ENSG00000164142 | 98.82 | 0.19 | 0.0101 | 0.0421 | 0.78 |
| FAM160A2 | ENSG00000051009 | 306.75 | 0.21 | 0.0044 | 0.0351 | 0.81 |
| FAM160B2 | ENSG00000158863 | 165.79 | 0.22 | 0.0024 | 0.0342 | 1.12 |
| FAM161B | ENSG00000156050 | 16.73 | 0.19 | 0.0132 | 0.0460 | 0.62 |
| FAM162A | ENSG00000114023 | 76.34 | 0.19 | 0.0067 | 0.0373 | 0.82 |
| FAM167B | ENSG00000183615 | 5.82 | 0.22 | 0.0070 | 0.0376 | 1.11 |
| FAM168B | ENSG00000152102 | 281.05 | 0.20 | 0.0057 | 0.0363 | 1.57 |
| FAM171A1 | ENSG00000148468 | 143.65 | 0.18 | 0.0095 | 0.0413 | 1.26 |
| FAM173A | ENSG00000103254 | 13.40 | 0.22 | 0.0065 | 0.0371 | 1.76 |
| FAM173B | ENSG00000150756 | 19.65 | 0.19 | 0.0094 | 0.0411 | 1.93 |
| FAM174B | ENSG00000185442 | 35.42 | 0.22 | 0.0048 | 0.0354 | 1.09 |
| FAM177A1 | ENSG00000151327 | 93.30 | 0.20 | 0.0064 | 0.0370 | 1.10 |
| FAM177B | ENSG00000197520 | 30.22 | 0.19 | 0.0149 | 0.0485 | 0.43 |
| FAM178A | ENSG00000119906 | 196.51 | 0.18 | 0.0120 | 0.0444 | 1.12 |
| FAM184B | ENSG00000047662 | 85.22 | 0.18 | 0.0112 | 0.0434 | 1.18 |
| FAM185A | ENSG00000222011 | 29.84 | 0.19 | 0.0084 | 0.0397 | 1.56 |
| FAM186A | ENSG00000185958 | 17.44 | 0.23 | 0.0022 | 0.0342 | 0.99 |
| FAM188A | ENSG00000148481 | 65.58 | 0.18 | 0.0110 | 0.0432 | 1.12 |
| FAM189B | ENSG00000160767 | 32.12 | 0.24 | 0.0031 | 0.0342 | 2.43 |
| FAM18B1 | ENSG00000171928 | 47.36 | 0.20 | 0.0076 | 0.0382 | 1.28 |
| FAM190A | ENSG00000184305 | 103.97 | 0.19 | 0.0088 | 0.0402 | 1.09 |
| FAM192A | ENSG00000172775 | 129.35 | 0.18 | 0.0121 | 0.0445 | 1.47 |
| FAM193A | ENSG00000125386 | 158.96 | 0.18 | 0.0127 | 0.0454 | 1.13 |
| FAM193B | ENSG00000146067 | 188.54 | 0.19 | 0.0133 | 0.0462 | 1.21 |
| FAM195A | ENSG00000172366 | 56.30 | 0.19 | 0.0125 | 0.0451 | 1.36 |
| FAM196A | ENSG00000188916 | 69.02 | 0.20 | 0.0048 | 0.0354 | 0.93 |
| FAM199X | ENSG00000123575 | 112.28 | 0.19 | 0.0132 | 0.0460 | 1.90 |
| FAM200B | ENSG00000237765 | 35.06 | 0.27 | 0.0003 | 0.0342 | 1.34 |
| FAM204A | ENSG00000165669 | 65.05 | 0.19 | 0.0117 | 0.0439 | 1.59 |
| FAM206A | ENSG00000119328 | 29.79 | 0.25 | 0.0026 | 0.0342 | 1.95 |
| FAM207A | ENSG00000160256 | 11.44 | 0.24 | 0.0050 | 0.0356 | 2.07 |
| FAM20B | ENSG00000116199 | 127.30 | 0.19 | 0.0101 | 0.0421 | 1.77 |
| FAM21A | ENSG00000099290 | 70.70 | 0.20 | 0.0055 | 0.0363 | 1.23 |
| FAM21C | ENSG00000172661 | 84.82 | 0.22 | 0.0022 | 0.0342 | 1.13 |
| FAM22G | ENSG00000188152 | 49.59 | 0.20 | 0.0088 | 0.0402 | 1.16 |
| FAM32A | ENSG00000105058 | 68.03 | 0.20 | 0.0054 | 0.0362 | 1.68 |
| FAM3A | ENSG00000071889 | 82.30 | 0.24 | 0.0009 | 0.0342 | 1.36 |
| FAM3D | ENSG00000198643 | 372.47 | 0.21 | 0.0030 | 0.0342 | 0.70 |
| FAM40A | ENSG00000143093 | 102.88 | 0.18 | 0.0153 | 0.0489 | 1.56 |
| FAM47E | ENSG00000189157 | 57.62 | 0.22 | 0.0023 | 0.0342 | 0.99 |
| FAM48A | ENSG00000102710 | 141.85 | 0.20 | 0.0073 | 0.0379 | 1.98 |
| FAM54B | ENSG00000117640 | 100.37 | 0.20 | 0.0052 | 0.0359 | 0.93 |
| FAM55A | ENSG00000095110 | 862.56 | 0.23 | 0.0024 | 0.0342 | 0.20 |
| FAM55D | ENSG00000137634 | 253.05 | 0.19 | 0.0082 | 0.0393 | 0.26 |
| FAM58A | ENSG00000147382 | 13.18 | 0.18 | 0.0150 | 0.0486 | 2.34 |
| FAM60A | ENSG00000139146 | 83.12 | 0.21 | 0.0054 | 0.0362 | 2.75 |
| FAM63A | ENSG00000143409 | 138.77 | 0.24 | 0.0006 | 0.0342 | 0.99 |
| FAM63B | ENSG00000128923 | 68.10 | 0.22 | 0.0024 | 0.0342 | 1.40 |
| FAM73B | ENSG00000148343 | 127.94 | 0.21 | 0.0073 | 0.0379 | 0.94 |
| FAM82A2 | ENSG00000137824 | 60.18 | 0.22 | 0.0025 | 0.0342 | 1.42 |
| FAM82B | ENSG00000176623 | 110.06 | 0.18 | 0.0148 | 0.0484 | 1.36 |
| FAM83B | ENSG00000168143 | 78.08 | 0.25 | 0.0007 | 0.0342 | 1.46 |
| FAM83E | ENSG00000105523 | 180.47 | 0.21 | 0.0055 | 0.0363 | 0.64 |
| FAM83H | ENSG00000180921 | 219.24 | 0.23 | 0.0044 | 0.0351 | 2.91 |
| FAM84A | ENSG00000162981 | 605.32 | 0.26 | 0.0005 | 0.0342 | 1.45 |
| FAM84B | ENSG00000168672 | 89.31 | 0.25 | 0.0019 | 0.0342 | 3.22 |
| FAM86A | ENSG00000118894 | 24.40 | 0.20 | 0.0069 | 0.0375 | 2.02 |
| FAM86C1 | ENSG00000158483 | 13.19 | 0.20 | 0.0077 | 0.0384 | 1.78 |
| FAM89B | ENSG00000176973 | 32.05 | 0.20 | 0.0107 | 0.0428 | 1.85 |
| FAM8A1 | ENSG00000137414 | 123.42 | 0.27 | 0.0006 | 0.0342 | 1.14 |
| FAM92A1 | ENSG00000188343 | 74.90 | 0.18 | 0.0146 | 0.0481 | 2.03 |
| FAM96A | ENSG00000166797 | 33.40 | 0.21 | 0.0067 | 0.0373 | 1.88 |
| FAN1 | ENSG00000198690 | 131.25 | 0.19 | 0.0092 | 0.0408 | 1.09 |
| FANCA | ENSG00000187741 | 132.63 | 0.19 | 0.0147 | 0.0482 | 2.08 |
| FANCF | ENSG00000183161 | 24.07 | 0.29 | <.0001 | 0.0342 | 2.53 |
| FANCL | ENSG00000115392 | 45.06 | 0.20 | 0.0102 | 0.0422 | 2.03 |
| FANK1 | ENSG00000203780 | 22.26 | 0.22 | 0.0039 | 0.0345 | 0.83 |
| FARP2 | ENSG00000006607 | 227.86 | 0.22 | 0.0029 | 0.0342 | 1.14 |
| FARS2 | ENSG00000145982 | 26.18 | 0.21 | 0.0035 | 0.0342 | 1.38 |
| FASN | ENSG00000169710 | 325.09 | 0.22 | 0.0048 | 0.0354 | 3.34 |
| FASTK | ENSG00000164896 | 111.64 | 0.24 | 0.0019 | 0.0342 | 1.40 |
| FASTKD1 | ENSG00000138399 | 85.36 | 0.20 | 0.0095 | 0.0413 | 1.18 |
| FASTKD3 | ENSG00000124279 | 14.70 | 0.18 | 0.0139 | 0.0469 | 1.55 |
| FAT1 | ENSG00000083857 | 1540.67 | 0.25 | 0.0021 | 0.0342 | 2.09 |
| FAT4 | ENSG00000196159 | 186.57 | 0.22 | 0.0034 | 0.0342 | 1.51 |
| FAU | ENSG00000149806 | 86.64 | 0.17 | 0.0137 | 0.0467 | 1.77 |
| FBL | ENSG00000105202 | 69.07 | 0.19 | 0.0117 | 0.0439 | 3.04 |
| FBLIM1 | ENSG00000162458 | 317.59 | 0.18 | 0.0156 | 0.0494 | 0.75 |
| FBP1 | ENSG00000165140 | 28.43 | 0.18 | 0.0122 | 0.0446 | 1.91 |
| FBXL12 | ENSG00000127452 | 34.39 | 0.19 | 0.0096 | 0.0414 | 1.17 |
| FBXL14 | ENSG00000171823 | 56.27 | 0.18 | 0.0136 | 0.0466 | 0.81 |
| FBXL15 | ENSG00000107872 | 21.69 | 0.25 | 0.0017 | 0.0342 | 1.01 |
| FBXL17 | ENSG00000145743 | 74.05 | 0.18 | 0.0122 | 0.0446 | 1.13 |
| FBXL20 | ENSG00000108306 | 190.14 | 0.20 | 0.0070 | 0.0376 | 1.30 |
| FBXL4 | ENSG00000112234 | 51.16 | 0.21 | 0.0049 | 0.0355 | 1.96 |
| FBXL6 | ENSG00000182325 | 55.03 | 0.24 | 0.0030 | 0.0342 | 2.77 |
| FBXL8 | ENSG00000135722 | 61.59 | 0.21 | 0.0051 | 0.0358 | 1.80 |
| FBXO16 | ENSG00000214050 | 68.08 | 0.18 | 0.0125 | 0.0451 | 1.41 |
| FBXO17 | ENSG00000104835 | 31.84 | 0.21 | 0.0059 | 0.0365 | 1.73 |
| FBXO18 | ENSG00000134452 | 215.25 | 0.18 | 0.0153 | 0.0489 | 1.28 |
| FBXO21 | ENSG00000135108 | 69.03 | 0.19 | 0.0101 | 0.0421 | 1.97 |
| FBXO25 | ENSG00000147364 | 58.55 | 0.21 | 0.0032 | 0.0342 | 0.93 |
| FBXO28 | ENSG00000143756 | 89.75 | 0.23 | 0.0024 | 0.0342 | 1.77 |
| FBXO31 | ENSG00000103264 | 110.86 | 0.24 | 0.0009 | 0.0342 | 1.55 |
| FBXO34 | ENSG00000178974 | 206.89 | 0.22 | 0.0023 | 0.0342 | 1.06 |
| FBXO38 | ENSG00000145868 | 125.22 | 0.18 | 0.0092 | 0.0408 | 1.27 |
| FBXO41 | ENSG00000163013 | 46.61 | 0.19 | 0.0153 | 0.0489 | 3.31 |
| FBXO42 | ENSG00000037637 | 111.74 | 0.18 | 0.0155 | 0.0492 | 1.24 |
| FBXO45 | ENSG00000174013 | 39.28 | 0.23 | 0.0037 | 0.0342 | 2.50 |
| FBXO46 | ENSG00000177051 | 82.78 | 0.19 | 0.0152 | 0.0489 | 1.56 |
| FBXO7 | ENSG00000100225 | 202.22 | 0.18 | 0.0144 | 0.0477 | 1.35 |
| FBXO8 | ENSG00000164117 | 28.65 | 0.18 | 0.0137 | 0.0467 | 1.03 |
| FBXO9 | ENSG00000112146 | 106.39 | 0.22 | 0.0033 | 0.0342 | 1.38 |
| FBXW11 | ENSG00000072803 | 110.52 | 0.18 | 0.0137 | 0.0467 | 1.56 |
| FBXW2 | ENSG00000119402 | 93.43 | 0.19 | 0.0081 | 0.0391 | 1.49 |
| FBXW5 | ENSG00000159069 | 169.06 | 0.21 | 0.0049 | 0.0355 | 1.18 |
| FCGRT | ENSG00000104870 | 300.16 | 0.20 | 0.0086 | 0.0399 | 1.15 |
| FCHO2 | ENSG00000157107 | 108.78 | 0.21 | 0.0031 | 0.0342 | 1.28 |
| FCRL1 | ENSG00000163534 | 29.77 | -0.22 | 0.0051 | 0.0358 | 0.15 |
| FCRL2 | ENSG00000132704 | 41.03 | -0.23 | 0.0037 | 0.0342 | 0.23 |
| FDFT1 | ENSG00000079459 | 157.42 | 0.22 | 0.0027 | 0.0342 | 2.11 |
| FDPS | ENSG00000160752 | 125.93 | 0.19 | 0.0104 | 0.0424 | 2.12 |
| FEM1A | ENSG00000141965 | 73.01 | 0.18 | 0.0143 | 0.0476 | 1.56 |
| FEM1B | ENSG00000169018 | 164.52 | 0.19 | 0.0094 | 0.0411 | 1.46 |
| FERMT1 | ENSG00000101311 | 234.35 | 0.24 | 0.0019 | 0.0342 | 4.76 |
| FGD6 | ENSG00000180263 | 145.66 | 0.18 | 0.0139 | 0.0469 | 1.73 |
| FGFR1OP | ENSG00000213066 | 75.90 | 0.20 | 0.0060 | 0.0366 | 1.76 |
| FGFR3 | ENSG00000068078 | 124.30 | 0.21 | 0.0046 | 0.0352 | 0.97 |
| FGFRL1 | ENSG00000127418 | 51.93 | 0.21 | 0.0072 | 0.0377 | 4.35 |
| FHDC1 | ENSG00000137460 | 100.83 | 0.21 | 0.0043 | 0.0351 | 1.78 |
| FHOD1 | ENSG00000135723 | 83.02 | 0.21 | 0.0065 | 0.0371 | 1.24 |
| FICD | ENSG00000198855 | 10.49 | 0.22 | 0.0026 | 0.0342 | 1.08 |
| FIG4 | ENSG00000112367 | 41.63 | 0.23 | 0.0016 | 0.0342 | 1.27 |
| FIP1L1 | ENSG00000145216 | 162.46 | 0.19 | 0.0093 | 0.0409 | 1.18 |
| FIS1 | ENSG00000214253 | 52.97 | 0.18 | 0.0148 | 0.0484 | 1.77 |
| FITM2 | ENSG00000197296 | 70.74 | 0.19 | 0.0095 | 0.0413 | 2.52 |
| FKBP15 | ENSG00000119321 | 184.22 | 0.20 | 0.0073 | 0.0379 | 1.30 |
| FKBP1A | ENSG00000088832 | 219.17 | 0.18 | 0.0160 | 0.0499 | 1.53 |
| FKBP2 | ENSG00000173486 | 73.25 | 0.20 | 0.0046 | 0.0352 | 1.02 |
| FKBP4 | ENSG00000004478 | 127.78 | 0.19 | 0.0102 | 0.0422 | 2.66 |
| FKBP9 | ENSG00000122642 | 144.89 | 0.21 | 0.0033 | 0.0342 | 2.36 |
| FKTN | ENSG00000106692 | 91.72 | 0.19 | 0.0110 | 0.0432 | 1.85 |
| FLCN | ENSG00000154803 | 152.53 | 0.22 | 0.0025 | 0.0342 | 1.07 |
| FLII | ENSG00000177731 | 258.40 | 0.19 | 0.0082 | 0.0393 | 1.25 |
| FLNB | ENSG00000136068 | 1857.85 | 0.20 | 0.0072 | 0.0377 | 1.13 |
| FLOT2 | ENSG00000132589 | 118.06 | 0.19 | 0.0118 | 0.0441 | 1.46 |
| FMN1 | ENSG00000248905 | 301.22 | 0.21 | 0.0036 | 0.0342 | 1.02 |
| FMO5 | ENSG00000131781 | 126.79 | 0.27 | 0.0003 | 0.0342 | 0.56 |
| FMOD | ENSG00000122176 | 111.49 | 0.19 | 0.0130 | 0.0458 | 1.05 |
| FMR1 | ENSG00000102081 | 120.54 | 0.18 | 0.0143 | 0.0476 | 2.39 |
| FN3K | ENSG00000167363 | 56.18 | 0.18 | 0.0153 | 0.0489 | 0.93 |
| FN3KRP | ENSG00000141560 | 47.76 | 0.24 | 0.0009 | 0.0342 | 1.58 |
| FNBP1L | ENSG00000137942 | 197.85 | 0.25 | 0.0009 | 0.0342 | 1.84 |
| FNDC3A | ENSG00000102531 | 323.54 | 0.19 | 0.0116 | 0.0439 | 1.56 |
| FNDC3B | ENSG00000075420 | 504.05 | 0.21 | 0.0041 | 0.0349 | 1.60 |
| FNIP2 | ENSG00000052795 | 284.57 | 0.19 | 0.0080 | 0.0389 | 0.97 |
| FNTA | ENSG00000168522 | 121.45 | 0.19 | 0.0103 | 0.0423 | 1.90 |
| FOXA1 | ENSG00000129514 | 79.14 | 0.21 | 0.0047 | 0.0353 | 0.80 |
| FOXA2 | ENSG00000125798 | 58.15 | 0.19 | 0.0135 | 0.0464 | 1.69 |
| FOXA3 | ENSG00000170608 | 48.32 | 0.18 | 0.0148 | 0.0484 | 0.98 |
| FOXD1 | ENSG00000183900 | 1.81 | 0.21 | 0.0122 | 0.0446 | 4.37 |
| FOXD2 | ENSG00000186564 | 74.09 | 0.27 | 0.0006 | 0.0342 | 0.88 |
| FOXH1 | ENSG00000160973 | 68.86 | 0.19 | 0.0124 | 0.0450 | 1.39 |
| FOXK1 | ENSG00000164916 | 312.40 | 0.21 | 0.0048 | 0.0354 | 2.68 |
| FOXM1 | ENSG00000111206 | 72.49 | 0.19 | 0.0144 | 0.0477 | 3.24 |
| FOXO4 | ENSG00000184481 | 57.74 | 0.21 | 0.0048 | 0.0354 | 1.31 |
| FOXP4 | ENSG00000137166 | 165.57 | 0.21 | 0.0049 | 0.0355 | 2.51 |
| FOXRED2 | ENSG00000100350 | 27.95 | 0.22 | 0.0053 | 0.0361 | 2.45 |
| FPGT | ENSG00000254685 | 36.80 | 0.20 | 0.0117 | 0.0439 | 1.18 |
| FRAT2 | ENSG00000181274 | 45.86 | 0.25 | 0.0013 | 0.0342 | 1.46 |
| FRG1 | ENSG00000109536 | 26.44 | 0.22 | 0.0026 | 0.0342 | 1.75 |
| FRG1B | ENSG00000149531 | 36.01 | 0.20 | 0.0072 | 0.0377 | 1.60 |
| FRK | ENSG00000111816 | 69.53 | 0.24 | 0.0011 | 0.0342 | 1.34 |
| FRMD3 | ENSG00000172159 | 72.07 | 0.27 | 0.0009 | 0.0342 | 0.44 |
| FRRS1 | ENSG00000156869 | 23.74 | 0.21 | 0.0032 | 0.0342 | 0.74 |
| FTH1 | ENSG00000167996 | 836.73 | 0.19 | 0.0103 | 0.0423 | 1.30 |
| FTL | ENSG00000087086 | 930.93 | 0.20 | 0.0081 | 0.0391 | 2.28 |
| FTSJ3 | ENSG00000108592 | 56.94 | 0.19 | 0.0107 | 0.0428 | 1.74 |
| FTSJD1 | ENSG00000180917 | 54.51 | 0.25 | 0.0015 | 0.0342 | 1.88 |
| FUBP1 | ENSG00000162613 | 277.49 | 0.18 | 0.0107 | 0.0428 | 1.54 |
| FUBP3 | ENSG00000107164 | 114.83 | 0.21 | 0.0040 | 0.0346 | 1.83 |
| FUCA2 | ENSG00000001036 | 69.85 | 0.20 | 0.0060 | 0.0366 | 1.90 |
| FUK | ENSG00000157353 | 97.55 | 0.23 | 0.0023 | 0.0342 | 1.59 |
| FUNDC1 | ENSG00000069509 | 8.84 | 0.18 | 0.0160 | 0.0499 | 2.14 |
| FUT10 | ENSG00000172728 | 49.60 | 0.20 | 0.0073 | 0.0379 | 1.53 |
| FUT2 | ENSG00000176920 | 120.14 | 0.26 | 0.0005 | 0.0342 | 1.17 |
| FUT3 | ENSG00000171124 | 262.16 | 0.18 | 0.0140 | 0.0471 | 1.01 |
| FUT4 | ENSG00000196371 | 175.42 | 0.18 | 0.0150 | 0.0486 | 1.88 |
| FUT5 | ENSG00000130383 | 34.11 | 0.22 | 0.0046 | 0.0352 | 1.47 |
| FUT6 | ENSG00000156413 | 150.58 | 0.19 | 0.0128 | 0.0456 | 0.92 |
| FUT8 | ENSG00000033170 | 83.87 | 0.22 | 0.0051 | 0.0358 | 1.76 |
| FXR1 | ENSG00000114416 | 115.33 | 0.20 | 0.0064 | 0.0370 | 2.10 |
| FXR2 | ENSG00000129245 | 58.35 | 0.19 | 0.0086 | 0.0399 | 1.32 |
| FXYD3 | ENSG00000089356 | 462.21 | 0.28 | 0.0002 | 0.0342 | 0.69 |
| FYCO1 | ENSG00000163820 | 254.08 | 0.20 | 0.0062 | 0.0368 | 1.29 |
| FZD1 | ENSG00000157240 | 55.55 | 0.22 | 0.0042 | 0.0350 | 1.21 |
| FZD3 | ENSG00000104290 | 32.38 | 0.19 | 0.0119 | 0.0442 | 3.25 |
| FZD4 | ENSG00000174804 | 73.30 | 0.18 | 0.0143 | 0.0476 | 1.52 |
| FZD5 | ENSG00000163251 | 525.41 | 0.27 | <.0001 | 0.0342 | 1.06 |
| FZD8 | ENSG00000177283 | 25.63 | 0.21 | 0.0040 | 0.0346 | 1.78 |
| FZR1 | ENSG00000105325 | 110.99 | 0.21 | 0.0074 | 0.0380 | 1.46 |
| G3BP2 | ENSG00000138757 | 225.11 | 0.19 | 0.0118 | 0.0441 | 1.62 |
| G6PD | ENSG00000160211 | 32.98 | 0.21 | 0.0071 | 0.0376 | 2.13 |
| GAB1 | ENSG00000109458 | 279.20 | 0.21 | 0.0044 | 0.0351 | 0.78 |
| GABARAP | ENSG00000170296 | 109.20 | 0.18 | 0.0155 | 0.0492 | 1.18 |
| GABARAPL2 | ENSG00000034713 | 35.20 | 0.18 | 0.0132 | 0.0460 | 1.72 |
| GABBR1 | ENSG00000204681 | 182.56 | 0.21 | 0.0061 | 0.0368 | 1.78 |
| GABPA | ENSG00000154727 | 45.46 | 0.20 | 0.0043 | 0.0351 | 1.58 |
| GABPB2 | ENSG00000143458 | 156.58 | 0.20 | 0.0063 | 0.0370 | 1.32 |
| GABRA2 | ENSG00000151834 | 21.45 | 0.23 | 0.0048 | 0.0354 | 2.10 |
| GABRE | ENSG00000102287 | 193.50 | 0.19 | 0.0147 | 0.0482 | 2.84 |
| GADD45GIP1 | ENSG00000179271 | 13.55 | 0.20 | 0.0070 | 0.0376 | 2.49 |
| GAK | ENSG00000178950 | 495.25 | 0.20 | 0.0066 | 0.0373 | 1.11 |
| GAL3ST2 | ENSG00000154252 | 77.88 | 0.20 | 0.0056 | 0.0363 | 0.69 |
| GALE | ENSG00000117308 | 99.05 | 0.22 | 0.0034 | 0.0342 | 1.02 |
| GALM | ENSG00000143891 | 71.90 | 0.20 | 0.0030 | 0.0342 | 1.10 |
| GALNS | ENSG00000141012 | 42.94 | 0.22 | 0.0033 | 0.0342 | 1.41 |
| GALNT1 | ENSG00000141429 | 158.68 | 0.22 | 0.0025 | 0.0342 | 1.38 |
| GALNT10 | ENSG00000164574 | 222.34 | 0.20 | 0.0069 | 0.0375 | 1.51 |
| GALNT11 | ENSG00000178234 | 100.38 | 0.19 | 0.0110 | 0.0432 | 1.59 |
| GALNT12 | ENSG00000119514 | 168.24 | 0.25 | 0.0006 | 0.0342 | 0.75 |
| GALNT2 | ENSG00000143641 | 159.39 | 0.18 | 0.0134 | 0.0463 | 1.65 |
| GALNT3 | ENSG00000115339 | 114.70 | 0.26 | 0.0016 | 0.0342 | 1.66 |
| GALNT5 | ENSG00000136542 | 265.08 | 0.29 | 0.0003 | 0.0342 | 0.95 |
| GALNT7 | ENSG00000109586 | 237.16 | 0.26 | 0.0005 | 0.0342 | 0.92 |
| GALT | ENSG00000213930 | 56.74 | 0.22 | 0.0034 | 0.0342 | 1.27 |
| GAN | ENSG00000127688 | 66.06 | 0.20 | 0.0051 | 0.0358 | 1.65 |
| GANAB | ENSG00000089597 | 386.87 | 0.18 | 0.0094 | 0.0411 | 1.63 |
| GANC | ENSG00000214013 | 131.94 | 0.23 | 0.0010 | 0.0342 | 0.96 |
| GAPDH | ENSG00000111640 | 1017.90 | 0.25 | 0.0016 | 0.0342 | 2.43 |
| GARS | ENSG00000106105 | 103.55 | 0.20 | 0.0090 | 0.0405 | 3.23 |
| GAS2L1 | ENSG00000185340 | 35.20 | 0.20 | 0.0074 | 0.0380 | 1.10 |
| GAS6 | ENSG00000183087 | 236.38 | 0.22 | 0.0046 | 0.0352 | 1.27 |
| GAS8 | ENSG00000141013 | 37.69 | 0.20 | 0.0084 | 0.0397 | 2.09 |
| GATA6 | ENSG00000141448 | 103.74 | 0.25 | 0.0009 | 0.0342 | 1.21 |
| GATAD1 | ENSG00000157259 | 140.93 | 0.23 | 0.0019 | 0.0342 | 1.48 |
| GATAD2A | ENSG00000167491 | 276.04 | 0.18 | 0.0122 | 0.0446 | 1.69 |
| GATC | ENSG00000111780 | 7.06 | 0.19 | 0.0144 | 0.0477 | 1.86 |
| GBA | ENSG00000177628 | 41.01 | 0.20 | 0.0080 | 0.0389 | 1.29 |
| GBA2 | ENSG00000070610 | 340.47 | 0.20 | 0.0068 | 0.0374 | 0.86 |
| GBF1 | ENSG00000107862 | 354.12 | 0.21 | 0.0040 | 0.0346 | 1.09 |
| GBP2 | ENSG00000162645 | 93.80 | 0.23 | 0.0026 | 0.0342 | 1.47 |
| GBP3 | ENSG00000117226 | 118.55 | 0.30 | 0.0003 | 0.0342 | 1.00 |
| GCAT | ENSG00000100116 | 14.44 | 0.22 | 0.0037 | 0.0342 | 1.55 |
| GCC2 | ENSG00000135968 | 356.90 | 0.22 | 0.0041 | 0.0349 | 1.44 |
| GCLC | ENSG00000001084 | 95.76 | 0.18 | 0.0161 | 0.0500 | 2.00 |
| GCN1L1 | ENSG00000089154 | 367.29 | 0.20 | 0.0076 | 0.0382 | 2.03 |
| GCNT1 | ENSG00000187210 | 114.53 | 0.25 | 0.0023 | 0.0342 | 1.54 |
| GCNT3 | ENSG00000140297 | 312.36 | 0.21 | 0.0085 | 0.0399 | 0.37 |
| GDA | ENSG00000119125 | 183.45 | 0.26 | 0.0059 | 0.0365 | 0.85 |
| GDE1 | ENSG00000006007 | 193.87 | 0.20 | 0.0065 | 0.0371 | 1.32 |
| GDI2 | ENSG00000057608 | 257.95 | 0.22 | 0.0026 | 0.0342 | 2.07 |
| GDPD3 | ENSG00000102886 | 32.40 | 0.20 | 0.0089 | 0.0403 | 0.83 |
| GEMIN4 | ENSG00000179409 | 52.66 | 0.20 | 0.0061 | 0.0368 | 1.79 |
| GEMIN5 | ENSG00000082516 | 41.25 | 0.19 | 0.0078 | 0.0386 | 2.76 |
| GEMIN8 | ENSG00000046647 | 24.96 | 0.25 | 0.0015 | 0.0342 | 1.78 |
| GET4 | ENSG00000239857 | 147.47 | 0.25 | 0.0012 | 0.0342 | 2.09 |
| GFER | ENSG00000127554 | 48.35 | 0.25 | 0.0015 | 0.0342 | 1.10 |
| GFI1 | ENSG00000162676 | 23.77 | 0.23 | 0.0017 | 0.0342 | 0.69 |
| GFOD2 | ENSG00000141098 | 30.75 | 0.18 | 0.0108 | 0.0429 | 1.19 |
| GFPT1 | ENSG00000198380 | 611.46 | 0.27 | 0.0007 | 0.0342 | 1.62 |
| GGA1 | ENSG00000100083 | 268.83 | 0.18 | 0.0160 | 0.0499 | 1.06 |
| GGA3 | ENSG00000125447 | 134.45 | 0.21 | 0.0055 | 0.0363 | 1.43 |
| GGPS1 | ENSG00000152904 | 49.99 | 0.24 | 0.0017 | 0.0342 | 1.84 |
| GGT1 | ENSG00000100031 | 30.58 | 0.21 | 0.0060 | 0.0366 | 1.05 |
| GGT5 | ENSG00000099998 | 24.05 | 0.20 | 0.0088 | 0.0402 | 1.88 |
| GGT6 | ENSG00000167741 | 164.85 | 0.26 | 0.0004 | 0.0342 | 0.53 |
| GHDC | ENSG00000167925 | 30.09 | 0.20 | 0.0088 | 0.0402 | 2.13 |
| GHITM | ENSG00000165678 | 168.45 | 0.19 | 0.0101 | 0.0421 | 1.51 |
| GHR | ENSG00000112964 | 43.44 | 0.22 | 0.0042 | 0.0350 | 0.36 |
| GIGYF1 | ENSG00000146830 | 305.43 | 0.20 | 0.0100 | 0.0419 | 1.78 |
| GIGYF2 | ENSG00000204120 | 278.55 | 0.18 | 0.0132 | 0.0460 | 1.56 |
| GIPC1 | ENSG00000123159 | 238.64 | 0.22 | 0.0043 | 0.0351 | 0.86 |
| GIPR | ENSG00000010310 | 22.28 | 0.21 | 0.0073 | 0.0379 | 0.46 |
| GIT1 | ENSG00000108262 | 97.69 | 0.20 | 0.0076 | 0.0382 | 2.49 |
| GIT2 | ENSG00000139436 | 148.81 | 0.21 | 0.0048 | 0.0354 | 1.19 |
| GJA9 | ENSG00000131233 | 25.26 | 0.27 | 0.0004 | 0.0342 | 1.14 |
| GJB1 | ENSG00000169562 | 38.35 | 0.21 | 0.0046 | 0.0352 | 2.09 |
| GJB2 | ENSG00000165474 | 46.03 | 0.25 | 0.0018 | 0.0342 | 2.06 |
| GK5 | ENSG00000175066 | 163.48 | 0.21 | 0.0040 | 0.0346 | 1.10 |
| GLB1 | ENSG00000170266 | 81.66 | 0.21 | 0.0024 | 0.0342 | 1.86 |
| GLB1L2 | ENSG00000149328 | 48.70 | 0.18 | 0.0131 | 0.0460 | 2.52 |
| GLCE | ENSG00000138604 | 94.28 | 0.28 | 0.0008 | 0.0342 | 2.20 |
| GLE1 | ENSG00000119392 | 79.34 | 0.22 | 0.0029 | 0.0342 | 1.81 |
| GLG1 | ENSG00000090863 | 388.05 | 0.22 | 0.0016 | 0.0342 | 1.78 |
| GLI1 | ENSG00000111087 | 28.26 | 0.18 | 0.0154 | 0.0491 | 1.37 |
| GLI4 | ENSG00000250571 | 73.30 | 0.19 | 0.0119 | 0.0442 | 1.19 |
| GLO1 | ENSG00000124767 | 60.45 | 0.22 | 0.0049 | 0.0355 | 3.55 |
| GLP2R | ENSG00000065325 | 35.25 | 0.19 | 0.0114 | 0.0437 | 0.17 |
| GLRX5 | ENSG00000182512 | 28.09 | 0.18 | 0.0119 | 0.0442 | 1.50 |
| GLT25D1 | ENSG00000130309 | 122.88 | 0.18 | 0.0141 | 0.0472 | 2.35 |
| GLTP | ENSG00000139433 | 97.19 | 0.20 | 0.0055 | 0.0363 | 0.65 |
| GLUD1 | ENSG00000148672 | 175.35 | 0.19 | 0.0086 | 0.0399 | 1.42 |
| GLYR1 | ENSG00000140632 | 189.45 | 0.20 | 0.0065 | 0.0371 | 1.46 |
| GMCL1 | ENSG00000087338 | 46.36 | 0.19 | 0.0086 | 0.0399 | 1.86 |
| GMDS | ENSG00000112699 | 171.81 | 0.20 | 0.0074 | 0.0380 | 1.74 |
| GMPPA | ENSG00000144591 | 72.91 | 0.23 | 0.0021 | 0.0342 | 1.28 |
| GMPPB | ENSG00000173540 | 168.02 | 0.18 | 0.0147 | 0.0482 | 0.93 |
| GMPS | ENSG00000163655 | 84.68 | 0.19 | 0.0105 | 0.0426 | 2.68 |
| GNA11 | ENSG00000088256 | 131.51 | 0.20 | 0.0108 | 0.0429 | 0.78 |
| GNAL | ENSG00000141404 | 70.63 | 0.18 | 0.0129 | 0.0457 | 0.85 |
| GNAQ | ENSG00000156052 | 310.18 | 0.26 | 0.0006 | 0.0342 | 0.98 |
| GNB1 | ENSG00000078369 | 483.58 | 0.20 | 0.0058 | 0.0364 | 1.53 |
| GNB2 | ENSG00000172354 | 166.13 | 0.19 | 0.0090 | 0.0405 | 1.80 |
| GNB2L1 | ENSG00000204628 | 677.98 | 0.22 | 0.0035 | 0.0342 | 2.36 |
| GNE | ENSG00000159921 | 320.86 | 0.21 | 0.0035 | 0.0342 | 0.83 |
| GNG12 | ENSG00000172380 | 287.37 | 0.25 | 0.0012 | 0.0342 | 1.20 |
| GNG5 | ENSG00000174021 | 82.76 | 0.19 | 0.0118 | 0.0441 | 1.49 |
| GNPAT | ENSG00000116906 | 59.05 | 0.26 | 0.0004 | 0.0342 | 2.02 |
| GNPDA1 | ENSG00000113552 | 40.08 | 0.23 | 0.0026 | 0.0342 | 2.50 |
| GNPNAT1 | ENSG00000100522 | 94.61 | 0.23 | 0.0033 | 0.0342 | 1.94 |
| GNPTAB | ENSG00000111670 | 307.06 | 0.23 | 0.0007 | 0.0342 | 0.90 |
| GNPTG | ENSG00000090581 | 63.79 | 0.19 | 0.0110 | 0.0432 | 1.41 |
| GNS | ENSG00000135677 | 170.58 | 0.19 | 0.0090 | 0.0405 | 1.98 |
| GOLGA1 | ENSG00000136935 | 100.90 | 0.23 | 0.0020 | 0.0342 | 1.22 |
| GOLGA2 | ENSG00000167110 | 245.49 | 0.19 | 0.0106 | 0.0427 | 1.22 |
| GOLGA3 | ENSG00000090615 | 719.57 | 0.21 | 0.0043 | 0.0351 | 1.21 |
| GOLGA4 | ENSG00000144674 | 708.63 | 0.20 | 0.0054 | 0.0362 | 1.40 |
| GOLGA5 | ENSG00000066455 | 83.33 | 0.23 | 0.0025 | 0.0342 | 1.47 |
| GOLGA7 | ENSG00000147533 | 53.42 | 0.20 | 0.0078 | 0.0386 | 1.90 |
| GOLGB1 | ENSG00000173230 | 927.10 | 0.22 | 0.0041 | 0.0349 | 1.29 |
| GOLIM4 | ENSG00000173905 | 319.85 | 0.21 | 0.0038 | 0.0342 | 1.72 |
| GOLM1 | ENSG00000135052 | 697.02 | 0.24 | 0.0013 | 0.0342 | 0.63 |
| GOLPH3 | ENSG00000113384 | 178.92 | 0.20 | 0.0064 | 0.0370 | 1.56 |
| GOLPH3L | ENSG00000143457 | 147.37 | 0.25 | 0.0008 | 0.0342 | 1.18 |
| GOLT1B | ENSG00000111711 | 36.77 | 0.20 | 0.0119 | 0.0442 | 2.09 |
| GOPC | ENSG00000047932 | 126.92 | 0.18 | 0.0129 | 0.0457 | 1.39 |
| GORASP1 | ENSG00000114745 | 197.64 | 0.20 | 0.0070 | 0.0376 | 1.11 |
| GORASP2 | ENSG00000115806 | 126.44 | 0.21 | 0.0050 | 0.0356 | 1.67 |
| GOSR2 | ENSG00000108433 | 165.03 | 0.18 | 0.0146 | 0.0481 | 1.27 |
| GOT1 | ENSG00000120053 | 68.34 | 0.19 | 0.0067 | 0.0373 | 1.03 |
| GOT2 | ENSG00000125166 | 104.26 | 0.21 | 0.0035 | 0.0342 | 2.00 |
| GP2 | ENSG00000169347 | 21.21 | 0.21 | 0.0060 | 0.0366 | 0.36 |
| GP6 | ENSG00000088053 | 11.81 | 0.20 | 0.0100 | 0.0419 | 1.41 |
| GPA33 | ENSG00000143167 | 564.62 | 0.20 | 0.0071 | 0.0376 | 0.50 |
| GPAA1 | ENSG00000197858 | 77.31 | 0.21 | 0.0056 | 0.0363 | 1.77 |
| GPANK1 | ENSG00000204438 | 28.40 | 0.21 | 0.0062 | 0.0368 | 1.48 |
| GPATCH8 | ENSG00000186566 | 200.13 | 0.18 | 0.0157 | 0.0496 | 1.57 |
| GPBP1L1 | ENSG00000159592 | 485.04 | 0.22 | 0.0013 | 0.0342 | 1.16 |
| GPC4 | ENSG00000076716 | 47.31 | 0.19 | 0.0087 | 0.0401 | 3.01 |
| GPD1L | ENSG00000152642 | 155.79 | 0.19 | 0.0116 | 0.0439 | 0.89 |
| GPD2 | ENSG00000115159 | 161.59 | 0.20 | 0.0076 | 0.0382 | 1.38 |
| GPI | ENSG00000105220 | 383.04 | 0.20 | 0.0067 | 0.0373 | 2.14 |
| GPKOW | ENSG00000068394 | 24.58 | 0.19 | 0.0089 | 0.0403 | 1.65 |
| GPM6B | ENSG00000046653 | 56.68 | 0.20 | 0.0104 | 0.0424 | 0.90 |
| GPN1 | ENSG00000198522 | 51.08 | 0.23 | 0.0029 | 0.0342 | 1.77 |
| GPN2 | ENSG00000142751 | 32.36 | 0.20 | 0.0072 | 0.0377 | 1.05 |
| GPR107 | ENSG00000148358 | 284.58 | 0.23 | 0.0016 | 0.0342 | 1.50 |
| GPR108 | ENSG00000125734 | 63.40 | 0.18 | 0.0129 | 0.0457 | 1.18 |
| GPR125 | ENSG00000152990 | 296.55 | 0.23 | 0.0020 | 0.0342 | 1.01 |
| GPR126 | ENSG00000112414 | 76.66 | 0.24 | 0.0028 | 0.0342 | 1.39 |
| GPR135 | ENSG00000181619 | 22.91 | 0.19 | 0.0133 | 0.0462 | 1.51 |
| GPR137 | ENSG00000173264 | 48.87 | 0.22 | 0.0033 | 0.0342 | 1.18 |
| GPR153 | ENSG00000158292 | 124.28 | 0.22 | 0.0030 | 0.0342 | 1.27 |
| GPR157 | ENSG00000180758 | 40.30 | 0.18 | 0.0151 | 0.0487 | 3.15 |
| GPR160 | ENSG00000173890 | 123.06 | 0.21 | 0.0038 | 0.0342 | 1.95 |
| GPR162 | ENSG00000250510 | 17.81 | 0.25 | 0.0022 | 0.0342 | 0.68 |
| GPR172A | ENSG00000185803 | 53.33 | 0.19 | 0.0120 | 0.0444 | 3.09 |
| GPR21 | ENSG00000188394 | 13.86 | 0.19 | 0.0102 | 0.0422 | 1.06 |
| GPR35 | ENSG00000178623 | 144.85 | 0.27 | 0.0003 | 0.0342 | 2.24 |
| GPR39 | ENSG00000183840 | 33.32 | 0.18 | 0.0130 | 0.0458 | 1.58 |
| GPR56 | ENSG00000205336 | 161.73 | 0.26 | 0.0005 | 0.0342 | 3.18 |
| GPR68 | ENSG00000119714 | 35.74 | 0.24 | 0.0018 | 0.0342 | 0.90 |
| GPRC5C | ENSG00000170412 | 65.10 | 0.21 | 0.0061 | 0.0368 | 1.13 |
| GPRIN2 | ENSG00000204175 | 203.30 | 0.26 | 0.0002 | 0.0342 | 0.69 |
| GPRIN3 | ENSG00000185477 | 168.19 | 0.19 | 0.0098 | 0.0416 | 1.29 |
| GPS2 | ENSG00000132522 | 88.19 | 0.19 | 0.0079 | 0.0387 | 1.27 |
| GPSM2 | ENSG00000121957 | 58.10 | 0.21 | 0.0051 | 0.0358 | 4.73 |
| GPT | ENSG00000167701 | 85.84 | 0.20 | 0.0103 | 0.0423 | 0.40 |
| GPX1 | ENSG00000233276 | 44.70 | 0.19 | 0.0106 | 0.0427 | 2.59 |
| GPX2 | ENSG00000176153 | 269.28 | 0.19 | 0.0120 | 0.0444 | 2.52 |
| GPX4 | ENSG00000167468 | 73.16 | 0.19 | 0.0112 | 0.0434 | 2.25 |
| GRAMD3 | ENSG00000155324 | 92.45 | 0.18 | 0.0153 | 0.0489 | 0.80 |
| GRAMD4 | ENSG00000075240 | 270.27 | 0.18 | 0.0161 | 0.0500 | 0.89 |
| GRB2 | ENSG00000177885 | 202.22 | 0.19 | 0.0113 | 0.0435 | 1.42 |
| GRB7 | ENSG00000141738 | 41.27 | 0.26 | 0.0016 | 0.0342 | 3.03 |
| GRHL2 | ENSG00000083307 | 150.57 | 0.23 | 0.0019 | 0.0342 | 1.84 |
| GRHPR | ENSG00000137106 | 85.20 | 0.18 | 0.0141 | 0.0472 | 1.63 |
| GRID2IP | ENSG00000215045 | 19.19 | 0.22 | 0.0068 | 0.0374 | 1.39 |
| GRIN1 | ENSG00000176884 | 85.62 | 0.20 | 0.0099 | 0.0417 | 0.46 |
| GRIN2B | ENSG00000150086 | 10.11 | 0.23 | 0.0066 | 0.0373 | 9.27 |
| GRIN2D | ENSG00000105464 | 8.11 | 0.21 | 0.0133 | 0.0462 | 12.06 |
| GRINL1A | ENSG00000255529 | 81.37 | 0.19 | 0.0049 | 0.0355 | 1.38 |
| GRIPAP1 | ENSG00000068400 | 113.55 | 0.19 | 0.0106 | 0.0427 | 1.53 |
| GRN | ENSG00000030582 | 296.06 | 0.22 | 0.0024 | 0.0342 | 1.20 |
| GRSF1 | ENSG00000132463 | 169.10 | 0.23 | 0.0029 | 0.0342 | 1.75 |
| GRTP1 | ENSG00000139835 | 61.35 | 0.28 | <.0001 | 0.0342 | 1.67 |
| GRWD1 | ENSG00000105447 | 41.77 | 0.18 | 0.0149 | 0.0485 | 2.13 |
| GSDMA | ENSG00000167914 | 3.22 | 0.22 | 0.0095 | 0.0413 | 2.15 |
| GSDMB | ENSG00000073605 | 263.21 | 0.27 | 0.0002 | 0.0342 | 1.05 |
| GSDMD | ENSG00000104518 | 108.26 | 0.19 | 0.0104 | 0.0424 | 1.34 |
| GSK3B | ENSG00000082701 | 231.19 | 0.20 | 0.0049 | 0.0355 | 1.74 |
| GSR | ENSG00000104687 | 212.90 | 0.22 | 0.0015 | 0.0342 | 1.01 |
| GSS | ENSG00000100983 | 85.12 | 0.25 | 0.0009 | 0.0342 | 1.85 |
| GSTK1 | ENSG00000197448 | 257.43 | 0.19 | 0.0109 | 0.0431 | 1.16 |
| GSTO2 | ENSG00000065621 | 50.11 | 0.23 | 0.0020 | 0.0342 | 1.66 |
| GSTP1 | ENSG00000084207 | 115.64 | 0.23 | 0.0024 | 0.0342 | 3.00 |
| GTF2A2 | ENSG00000140307 | 77.95 | 0.22 | 0.0042 | 0.0350 | 0.89 |
| GTF2E1 | ENSG00000153767 | 23.07 | 0.22 | 0.0034 | 0.0342 | 1.73 |
| GTF2F1 | ENSG00000125651 | 96.10 | 0.21 | 0.0043 | 0.0351 | 1.70 |
| GTF2F2 | ENSG00000188342 | 32.64 | 0.22 | 0.0043 | 0.0351 | 3.58 |
| GTF2H3 | ENSG00000111358 | 33.14 | 0.20 | 0.0081 | 0.0391 | 2.32 |
| GTF2I | ENSG00000077809 | 224.36 | 0.20 | 0.0050 | 0.0356 | 2.21 |
| GTF3A | ENSG00000122034 | 69.89 | 0.21 | 0.0051 | 0.0358 | 4.78 |
| GTF3C2 | ENSG00000115207 | 133.04 | 0.18 | 0.0126 | 0.0452 | 1.58 |
| GTF3C3 | ENSG00000119041 | 72.73 | 0.18 | 0.0155 | 0.0492 | 1.95 |
| GTF3C4 | ENSG00000125484 | 90.60 | 0.23 | 0.0019 | 0.0342 | 2.49 |
| GTF3C5 | ENSG00000148308 | 69.86 | 0.24 | 0.0008 | 0.0342 | 1.84 |
| GTPBP10 | ENSG00000105793 | 58.17 | 0.24 | 0.0023 | 0.0342 | 2.36 |
| GTPBP2 | ENSG00000172432 | 155.17 | 0.20 | 0.0069 | 0.0375 | 1.49 |
| GTPBP3 | ENSG00000130299 | 29.94 | 0.19 | 0.0122 | 0.0446 | 1.88 |
| GTPBP5 | ENSG00000101181 | 54.02 | 0.21 | 0.0056 | 0.0363 | 2.34 |
| GUCY2C | ENSG00000070019 | 173.87 | 0.22 | 0.0010 | 0.0342 | 1.44 |
| GUF1 | ENSG00000151806 | 89.07 | 0.19 | 0.0117 | 0.0439 | 1.88 |
| GUSB | ENSG00000169919 | 84.01 | 0.18 | 0.0134 | 0.0463 | 1.55 |
| GYS1 | ENSG00000104812 | 113.66 | 0.21 | 0.0043 | 0.0351 | 1.32 |
| H1F0 | ENSG00000189060 | 243.57 | 0.23 | 0.0015 | 0.0342 | 1.93 |
| H2AFJ | ENSG00000246705 | 43.41 | 0.21 | 0.0044 | 0.0351 | 2.20 |
| H2AFY | ENSG00000113648 | 867.49 | 0.21 | 0.0042 | 0.0350 | 1.93 |
| H2AFZ | ENSG00000164032 | 36.94 | 0.18 | 0.0153 | 0.0489 | 2.59 |
| H6PD | ENSG00000049239 | 350.05 | 0.21 | 0.0051 | 0.0358 | 1.35 |
| HADH | ENSG00000138796 | 81.70 | 0.18 | 0.0153 | 0.0489 | 1.28 |
| HADHA | ENSG00000084754 | 277.48 | 0.19 | 0.0099 | 0.0417 | 1.31 |
| HADHB | ENSG00000138029 | 128.38 | 0.21 | 0.0044 | 0.0351 | 1.22 |
| HAGH | ENSG00000063854 | 30.18 | 0.19 | 0.0139 | 0.0469 | 1.24 |
| HARS | ENSG00000170445 | 120.61 | 0.18 | 0.0130 | 0.0458 | 1.28 |
| HARS2 | ENSG00000112855 | 71.58 | 0.19 | 0.0088 | 0.0402 | 1.31 |
| HAUS3 | ENSG00000214367 | 42.83 | 0.19 | 0.0142 | 0.0474 | 1.61 |
| HAUS4 | ENSG00000092036 | 33.51 | 0.20 | 0.0071 | 0.0376 | 1.86 |
| HAUS6 | ENSG00000147874 | 41.59 | 0.19 | 0.0147 | 0.0482 | 2.60 |
| HAVCR1 | ENSG00000113249 | 15.58 | 0.21 | 0.0052 | 0.0359 | 2.37 |
| HAX1 | ENSG00000143575 | 48.14 | 0.19 | 0.0087 | 0.0401 | 1.74 |
| HBP1 | ENSG00000105856 | 173.02 | 0.20 | 0.0055 | 0.0363 | 1.40 |
| HBS1L | ENSG00000112339 | 100.30 | 0.19 | 0.0101 | 0.0421 | 1.88 |
| HCCS | ENSG00000004961 | 29.51 | 0.19 | 0.0092 | 0.0408 | 1.87 |
| HCFC1 | ENSG00000172534 | 358.89 | 0.19 | 0.0083 | 0.0395 | 2.05 |
| HCN3 | ENSG00000143630 | 36.52 | 0.25 | 0.0015 | 0.0342 | 1.53 |
| HDAC10 | ENSG00000100429 | 74.61 | 0.21 | 0.0087 | 0.0401 | 1.68 |
| HDAC11 | ENSG00000163517 | 40.86 | 0.25 | 0.0008 | 0.0342 | 1.82 |
| HDAC5 | ENSG00000108840 | 91.18 | 0.19 | 0.0121 | 0.0445 | 1.59 |
| HDAC6 | ENSG00000094631 | 171.18 | 0.22 | 0.0037 | 0.0342 | 1.34 |
| HDGF | ENSG00000143321 | 292.50 | 0.21 | 0.0054 | 0.0362 | 2.74 |
| HDHD1 | ENSG00000130021 | 37.78 | 0.23 | 0.0028 | 0.0342 | 1.49 |
| HDHD2 | ENSG00000167220 | 40.00 | 0.22 | 0.0031 | 0.0342 | 1.10 |
| HDHD3 | ENSG00000119431 | 129.57 | 0.28 | 0.0003 | 0.0342 | 0.97 |
| HDLBP | ENSG00000115677 | 1184.56 | 0.21 | 0.0041 | 0.0349 | 1.47 |
| HEATR2 | ENSG00000164818 | 78.74 | 0.21 | 0.0041 | 0.0349 | 2.32 |
| HEATR5A | ENSG00000129493 | 165.92 | 0.20 | 0.0052 | 0.0359 | 1.17 |
| HEATR5B | ENSG00000008869 | 163.10 | 0.19 | 0.0092 | 0.0408 | 1.19 |
| HEATR6 | ENSG00000068097 | 32.81 | 0.23 | 0.0032 | 0.0342 | 1.75 |
| HEATR7A | ENSG00000179832 | 191.12 | 0.22 | 0.0032 | 0.0342 | 1.92 |
| HEBP2 | ENSG00000051620 | 72.27 | 0.24 | 0.0020 | 0.0342 | 1.93 |
| HECTD3 | ENSG00000126107 | 171.13 | 0.20 | 0.0054 | 0.0362 | 0.85 |
| HEMK1 | ENSG00000114735 | 120.96 | 0.25 | 0.0011 | 0.0342 | 1.27 |
| HEPH | ENSG00000089472 | 550.90 | 0.26 | 0.0007 | 0.0342 | 1.14 |
| HERC2 | ENSG00000128731 | 459.49 | 0.20 | 0.0079 | 0.0387 | 1.54 |
| HERC3 | ENSG00000138641 | 130.42 | 0.20 | 0.0082 | 0.0393 | 0.84 |
| HES1 | ENSG00000114315 | 104.55 | 0.18 | 0.0144 | 0.0477 | 1.43 |
| HES2 | ENSG00000069812 | 54.67 | 0.21 | 0.0035 | 0.0342 | 1.21 |
| HEXA | ENSG00000213614 | 102.21 | 0.19 | 0.0095 | 0.0413 | 1.26 |
| HEXB | ENSG00000049860 | 112.70 | 0.22 | 0.0036 | 0.0342 | 2.19 |
| HEXIM1 | ENSG00000186834 | 173.79 | 0.18 | 0.0125 | 0.0451 | 0.94 |
| HFE | ENSG00000010704 | 20.75 | 0.23 | 0.0024 | 0.0342 | 1.63 |
| HGD | ENSG00000113924 | 37.90 | 0.22 | 0.0024 | 0.0342 | 0.41 |
| HGS | ENSG00000185359 | 139.62 | 0.19 | 0.0142 | 0.0474 | 1.70 |
| HHIP | ENSG00000164161 | 122.16 | 0.19 | 0.0095 | 0.0413 | 0.34 |
| HHLA2 | ENSG00000114455 | 211.10 | 0.24 | 0.0020 | 0.0342 | 0.28 |
| HIAT1 | ENSG00000156875 | 81.01 | 0.18 | 0.0142 | 0.0474 | 1.50 |
| HIATL1 | ENSG00000148110 | 128.57 | 0.26 | 0.0005 | 0.0342 | 1.98 |
| HIBADH | ENSG00000106049 | 51.76 | 0.18 | 0.0096 | 0.0414 | 2.60 |
| HIF1AN | ENSG00000166135 | 106.62 | 0.20 | 0.0048 | 0.0354 | 1.37 |
| HIGD2A | ENSG00000146066 | 65.30 | 0.19 | 0.0101 | 0.0421 | 1.40 |
| HINFP | ENSG00000172273 | 68.82 | 0.26 | 0.0006 | 0.0342 | 1.18 |
| HINT1 | ENSG00000169567 | 113.49 | 0.23 | 0.0011 | 0.0342 | 1.58 |
| HINT2 | ENSG00000137133 | 15.56 | 0.22 | 0.0028 | 0.0342 | 1.36 |
| HINT3 | ENSG00000111911 | 62.01 | 0.21 | 0.0072 | 0.0377 | 2.03 |
| HIP1R | ENSG00000130787 | 222.11 | 0.18 | 0.0142 | 0.0474 | 1.40 |
| HIRIP3 | ENSG00000149929 | 13.31 | 0.19 | 0.0108 | 0.0429 | 1.53 |
| HIST1H1E | ENSG00000168298 | 110.53 | 0.20 | 0.0096 | 0.0414 | 2.08 |
| HIST1H2AC | ENSG00000180573 | 57.29 | 0.28 | 0.0002 | 0.0342 | 2.02 |
| HIST1H2AD | ENSG00000196866 | 17.33 | 0.20 | 0.0064 | 0.0370 | 2.89 |
| HIST1H2AJ | ENSG00000182611 | 17.25 | 0.20 | 0.0113 | 0.0435 | 2.94 |
| HIST1H2BC | ENSG00000180596 | 37.47 | 0.20 | 0.0085 | 0.0399 | 2.16 |
| HIST1H2BD | ENSG00000158373 | 42.27 | 0.19 | 0.0116 | 0.0439 | 2.16 |
| HIST1H2BF | ENSG00000197846 | 18.30 | 0.18 | 0.0153 | 0.0489 | 2.96 |
| HIST1H2BG | ENSG00000187990 | 22.71 | 0.25 | 0.0015 | 0.0342 | 3.60 |
| HIST1H2BK | ENSG00000197903 | 36.61 | 0.22 | 0.0036 | 0.0342 | 2.21 |
| HIST1H2BN | ENSG00000233822 | 15.62 | 0.19 | 0.0109 | 0.0431 | 2.75 |
| HIST1H2BO | ENSG00000196331 | 10.86 | 0.24 | 0.0038 | 0.0342 | 3.35 |
| HIST1H3D | ENSG00000197409 | 35.04 | 0.19 | 0.0136 | 0.0466 | 2.70 |
| HIST1H3H | ENSG00000203813 | 14.69 | 0.24 | 0.0029 | 0.0342 | 3.40 |
| HIST1H4B | ENSG00000124529 | 14.39 | 0.19 | 0.0119 | 0.0442 | 2.83 |
| HIST1H4E | ENSG00000198518 | 50.04 | 0.19 | 0.0122 | 0.0446 | 2.32 |
| HIST1H4J | ENSG00000197238 | 18.53 | 0.20 | 0.0124 | 0.0450 | 3.18 |
| HIST2H2BE | ENSG00000184678 | 52.24 | 0.21 | 0.0056 | 0.0363 | 2.01 |
| HJURP | ENSG00000123485 | 19.03 | 0.19 | 0.0131 | 0.0460 | 4.03 |
| HK2 | ENSG00000159399 | 527.46 | 0.22 | 0.0038 | 0.0342 | 0.80 |
| HKDC1 | ENSG00000156510 | 29.45 | 0.22 | 0.0065 | 0.0371 | 5.95 |
| HLA-A | ENSG00000206503 | 825.71 | 0.24 | 0.0015 | 0.0342 | 1.53 |
| HLA-B | ENSG00000234745 | 1316.45 | 0.22 | 0.0051 | 0.0358 | 1.52 |
| HLA-C | ENSG00000204525 | 830.27 | 0.23 | 0.0034 | 0.0342 | 1.29 |
| HLA-DRB1 | ENSG00000196126 | 124.80 | 0.20 | 0.0101 | 0.0421 | 1.15 |
| HLA-DRB5 | ENSG00000198502 | 46.93 | 0.21 | 0.0059 | 0.0365 | 1.12 |
| HLA-E | ENSG00000204592 | 752.24 | 0.21 | 0.0036 | 0.0342 | 1.32 |
| HLA-F | ENSG00000204642 | 150.59 | 0.19 | 0.0133 | 0.0462 | 1.27 |
| HLCS | ENSG00000159267 | 80.15 | 0.20 | 0.0067 | 0.0373 | 1.86 |
| HM13 | ENSG00000101294 | 185.59 | 0.18 | 0.0120 | 0.0444 | 2.46 |
| HMG20A | ENSG00000140382 | 93.45 | 0.22 | 0.0032 | 0.0342 | 1.27 |
| HMG20B | ENSG00000064961 | 99.44 | 0.20 | 0.0084 | 0.0397 | 1.33 |
| HMGA1 | ENSG00000137309 | 145.47 | 0.20 | 0.0112 | 0.0434 | 3.79 |
| HMGA2 | ENSG00000149948 | 54.99 | 0.22 | 0.0030 | 0.0342 | 2.14 |
| HMGCL | ENSG00000117305 | 64.70 | 0.22 | 0.0037 | 0.0342 | 0.94 |
| HMGN4 | ENSG00000182952 | 70.84 | 0.20 | 0.0080 | 0.0389 | 1.87 |
| HMGXB4 | ENSG00000100281 | 65.07 | 0.25 | 0.0013 | 0.0342 | 1.63 |
| HN1L | ENSG00000206053 | 117.62 | 0.20 | 0.0074 | 0.0380 | 2.72 |
| HNF1A | ENSG00000135100 | 89.21 | 0.29 | 0.0004 | 0.0342 | 1.79 |
| HNF1B | ENSG00000108753 | 124.05 | 0.24 | 0.0015 | 0.0342 | 0.96 |
| HNF4A | ENSG00000101076 | 564.21 | 0.18 | 0.0120 | 0.0444 | 1.65 |
| HNF4G | ENSG00000164749 | 141.52 | 0.22 | 0.0038 | 0.0342 | 1.11 |
| HNMT | ENSG00000150540 | 63.72 | 0.25 | 0.0003 | 0.0342 | 1.89 |
| HNRNPAB | ENSG00000197451 | 285.18 | 0.20 | 0.0073 | 0.0379 | 1.43 |
| HNRNPH2 | ENSG00000126945 | 65.97 | 0.22 | 0.0040 | 0.0346 | 2.16 |
| HNRNPK | ENSG00000165119 | 461.41 | 0.18 | 0.0142 | 0.0474 | 1.84 |
| HNRNPR | ENSG00000125944 | 273.99 | 0.18 | 0.0146 | 0.0481 | 1.83 |
| HNRNPUL2 | ENSG00000214753 | 342.68 | 0.18 | 0.0117 | 0.0439 | 1.60 |
| HOMER3 | ENSG00000051128 | 14.16 | 0.21 | 0.0054 | 0.0362 | 1.96 |
| HOMEZ | ENSG00000215271 | 43.28 | 0.24 | 0.0010 | 0.0342 | 1.29 |
| HOOK2 | ENSG00000095066 | 73.43 | 0.21 | 0.0041 | 0.0349 | 1.53 |
| HOOK3 | ENSG00000168172 | 95.48 | 0.22 | 0.0018 | 0.0342 | 1.65 |
| HOXA10 | ENSG00000253293 | 37.46 | 0.24 | 0.0012 | 0.0342 | 2.74 |
| HOXA11 | ENSG00000005073 | 16.56 | 0.25 | 0.0017 | 0.0342 | 2.41 |
| HOXA13 | ENSG00000106031 | 77.72 | 0.34 | <.0001 | 0.0342 | 1.63 |
| HOXA6 | ENSG00000106006 | 22.53 | 0.21 | 0.0074 | 0.0380 | 1.16 |
| HOXB13 | ENSG00000159184 | 222.45 | 0.21 | 0.0039 | 0.0345 | 0.26 |
| HOXB13-AS1 | ENSG00000229637 | 55.64 | 0.22 | 0.0025 | 0.0342 | 0.29 |
| HOXB3 | ENSG00000120093 | 141.50 | 0.26 | 0.0026 | 0.0342 | 2.29 |
| HOXB4 | ENSG00000182742 | 29.11 | 0.26 | 0.0037 | 0.0342 | 2.50 |
| HOXB5 | ENSG00000120075 | 65.26 | 0.33 | 0.0004 | 0.0342 | 1.77 |
| HOXB6 | ENSG00000108511 | 108.27 | 0.28 | 0.0034 | 0.0342 | 1.97 |
| HOXB7 | ENSG00000120087 | 16.87 | 0.37 | <.0001 | 0.0342 | 2.12 |
| HOXB8 | ENSG00000120068 | 14.76 | 0.22 | 0.0116 | 0.0439 | 5.76 |
| HOXB9 | ENSG00000170689 | 210.39 | 0.23 | 0.0043 | 0.0351 | 2.36 |
| HP1BP3 | ENSG00000127483 | 502.43 | 0.20 | 0.0052 | 0.0359 | 1.47 |
| HPCA | ENSG00000121905 | 64.73 | 0.18 | 0.0135 | 0.0464 | 0.63 |
| HPCAL1 | ENSG00000115756 | 135.05 | 0.20 | 0.0070 | 0.0376 | 1.43 |
| HPS3 | ENSG00000163755 | 57.30 | 0.20 | 0.0057 | 0.0363 | 1.83 |
| HPS4 | ENSG00000100099 | 158.55 | 0.21 | 0.0040 | 0.0346 | 1.62 |
| HRNR | ENSG00000197915 | 26.66 | 0.21 | 0.0102 | 0.0422 | 1.12 |
| HS1BP3 | ENSG00000118960 | 68.88 | 0.20 | 0.0065 | 0.0371 | 1.62 |
| HSBP1 | ENSG00000230989 | 53.53 | 0.18 | 0.0154 | 0.0491 | 2.08 |
| HSBP1L1 | ENSG00000226742 | 26.01 | 0.21 | 0.0032 | 0.0342 | 0.73 |
| HSD11B2 | ENSG00000176387 | 382.70 | 0.19 | 0.0111 | 0.0433 | 0.41 |
| HSD17B1 | ENSG00000108786 | 22.92 | 0.24 | 0.0019 | 0.0342 | 1.39 |
| HSD17B11 | ENSG00000198189 | 126.35 | 0.27 | 0.0002 | 0.0342 | 1.00 |
| HSD17B12 | ENSG00000149084 | 137.29 | 0.19 | 0.0123 | 0.0448 | 2.22 |
| HSD17B2 | ENSG00000086696 | 37.68 | 0.21 | 0.0057 | 0.0363 | 0.27 |
| HSD17B4 | ENSG00000133835 | 152.06 | 0.17 | 0.0148 | 0.0484 | 1.67 |
| HSDL2 | ENSG00000119471 | 69.66 | 0.20 | 0.0074 | 0.0380 | 2.05 |
| HSF4 | ENSG00000102878 | 83.14 | 0.20 | 0.0107 | 0.0428 | 2.07 |
| HSP90AA1 | ENSG00000080824 | 499.73 | 0.18 | 0.0152 | 0.0489 | 3.38 |
| HSP90AB1 | ENSG00000096384 | 406.32 | 0.19 | 0.0079 | 0.0387 | 4.04 |
| HSPA4 | ENSG00000170606 | 147.21 | 0.19 | 0.0117 | 0.0439 | 2.39 |
| HSPA8 | ENSG00000109971 | 583.43 | 0.24 | 0.0033 | 0.0342 | 3.13 |
| HSPA9 | ENSG00000113013 | 256.43 | 0.18 | 0.0131 | 0.0460 | 2.36 |
| HSPB2-C11orf52 | ENSG00000254445 | 8.80 | 0.22 | 0.0044 | 0.0351 | 1.31 |
| HSPG2 | ENSG00000142798 | 1087.94 | 0.18 | 0.0161 | 0.0500 | 1.62 |
| HTATIP2 | ENSG00000109854 | 50.16 | 0.24 | 0.0012 | 0.0342 | 1.86 |
| HTR3A | ENSG00000166736 | 3.75 | -0.24 | 0.0055 | 0.0363 | 0.57 |
| HTT | ENSG00000197386 | 443.54 | 0.20 | 0.0060 | 0.0366 | 1.35 |
| HUNK | ENSG00000142149 | 65.63 | 0.17 | 0.0155 | 0.0492 | 2.59 |
| HUWE1 | ENSG00000086758 | 1076.34 | 0.20 | 0.0067 | 0.0373 | 2.12 |
| HYAL2 | ENSG00000068001 | 42.76 | 0.19 | 0.0105 | 0.0426 | 1.85 |
| HYI | ENSG00000178922 | 73.47 | 0.19 | 0.0131 | 0.0460 | 0.97 |
| HYOU1 | ENSG00000149428 | 292.49 | 0.18 | 0.0126 | 0.0452 | 1.58 |
| IARS | ENSG00000196305 | 229.66 | 0.18 | 0.0145 | 0.0479 | 2.67 |
| IARS2 | ENSG00000067704 | 148.46 | 0.24 | 0.0011 | 0.0342 | 2.17 |
| ICA1 | ENSG00000003147 | 94.22 | 0.26 | 0.0005 | 0.0342 | 2.50 |
| ICK | ENSG00000112144 | 114.58 | 0.21 | 0.0028 | 0.0342 | 1.74 |
| ICMT | ENSG00000116237 | 145.98 | 0.26 | 0.0006 | 0.0342 | 1.62 |
| ID2 | ENSG00000115738 | 50.20 | 0.23 | 0.0017 | 0.0342 | 1.36 |
| IDH1 | ENSG00000138413 | 129.01 | 0.24 | 0.0020 | 0.0342 | 1.70 |
| IDH2 | ENSG00000182054 | 134.63 | 0.22 | 0.0039 | 0.0345 | 1.82 |
| IDH3G | ENSG00000067829 | 55.28 | 0.25 | 0.0006 | 0.0342 | 1.71 |
| IDUA | ENSG00000127415 | 48.46 | 0.23 | 0.0039 | 0.0345 | 1.40 |
| IER3IP1 | ENSG00000134049 | 26.72 | 0.19 | 0.0098 | 0.0416 | 1.54 |
| IER5L | ENSG00000188483 | 11.59 | 0.21 | 0.0076 | 0.0382 | 4.43 |
| IFIH1 | ENSG00000115267 | 47.38 | 0.22 | 0.0104 | 0.0424 | 1.71 |
| IFIT5 | ENSG00000152778 | 31.64 | 0.24 | 0.0067 | 0.0373 | 1.68 |
| IFITM1 | ENSG00000185885 | 51.53 | 0.20 | 0.0159 | 0.0498 | 5.31 |
| IFNAR1 | ENSG00000142166 | 162.87 | 0.24 | 0.0018 | 0.0342 | 1.47 |
| IFNGR1 | ENSG00000027697 | 83.21 | 0.20 | 0.0052 | 0.0359 | 1.52 |
| IFNGR2 | ENSG00000159128 | 105.23 | 0.21 | 0.0037 | 0.0342 | 1.70 |
| IFNK | ENSG00000147896 | 19.93 | 0.20 | 0.0079 | 0.0387 | 0.71 |
| IFT140 | ENSG00000187535 | 64.55 | 0.21 | 0.0041 | 0.0349 | 1.83 |
| IFT52 | ENSG00000101052 | 22.42 | 0.21 | 0.0037 | 0.0342 | 2.64 |
| IFT80 | ENSG00000068885 | 68.65 | 0.19 | 0.0123 | 0.0448 | 1.49 |
| IFT88 | ENSG00000032742 | 44.72 | 0.18 | 0.0161 | 0.0500 | 2.04 |
| IGBP1 | ENSG00000089289 | 55.53 | 0.22 | 0.0018 | 0.0342 | 2.57 |
| IGF1R | ENSG00000140443 | 315.37 | 0.17 | 0.0154 | 0.0491 | 1.68 |
| IGF2BP2 | ENSG00000073792 | 145.50 | 0.21 | 0.0054 | 0.0362 | 1.73 |
| IGF2R | ENSG00000197081 | 523.99 | 0.23 | 0.0010 | 0.0342 | 1.95 |
| IGFBP3 | ENSG00000146674 | 108.29 | 0.21 | 0.0067 | 0.0373 | 2.61 |
| IGFBP5 | ENSG00000115461 | 807.61 | 0.20 | 0.0109 | 0.0431 | 1.54 |
| IGIP | ENSG00000182700 | 45.09 | 0.20 | 0.0090 | 0.0405 | 1.19 |
| IGSF3 | ENSG00000143061 | 95.18 | 0.24 | 0.0017 | 0.0342 | 1.06 |
| IGSF8 | ENSG00000162729 | 68.23 | 0.22 | 0.0027 | 0.0342 | 1.34 |
| IGSF9 | ENSG00000085552 | 126.51 | 0.20 | 0.0096 | 0.0414 | 0.61 |
| IHH | ENSG00000163501 | 93.19 | 0.24 | 0.0015 | 0.0342 | 1.55 |
| IKBKAP | ENSG00000070061 | 103.99 | 0.21 | 0.0044 | 0.0351 | 1.90 |
| IKBKB | ENSG00000104365 | 248.15 | 0.22 | 0.0031 | 0.0342 | 1.45 |
| IKBKE | ENSG00000143466 | 52.21 | 0.24 | 0.0019 | 0.0342 | 1.63 |
| IKBKG | ENSG00000073009 | 29.64 | 0.19 | 0.0138 | 0.0468 | 1.46 |
| IL10RB | ENSG00000243646 | 99.59 | 0.20 | 0.0055 | 0.0363 | 0.96 |
| IL11RA | ENSG00000137070 | 38.56 | 0.20 | 0.0093 | 0.0409 | 0.81 |
| IL13RA1 | ENSG00000131724 | 250.61 | 0.24 | 0.0017 | 0.0342 | 1.78 |
| IL15RA | ENSG00000134470 | 29.78 | 0.20 | 0.0089 | 0.0403 | 1.63 |
| IL17RA | ENSG00000177663 | 128.17 | 0.18 | 0.0137 | 0.0467 | 1.09 |
| IL17RC | ENSG00000163702 | 46.27 | 0.22 | 0.0033 | 0.0342 | 1.11 |
| IL17RE | ENSG00000163701 | 119.38 | 0.30 | <.0001 | 0.0342 | 1.20 |
| IL18BP | ENSG00000137496 | 151.60 | 0.19 | 0.0090 | 0.0405 | 1.21 |
| IL20RA | ENSG00000016402 | 21.36 | 0.23 | 0.0028 | 0.0342 | 3.84 |
| IL22RA1 | ENSG00000142677 | 62.92 | 0.22 | 0.0040 | 0.0346 | 1.73 |
| IL23R | ENSG00000162594 | 11.80 | 0.21 | 0.0084 | 0.0397 | 0.76 |
| IL32 | ENSG00000008517 | 59.63 | 0.19 | 0.0136 | 0.0466 | 2.13 |
| IL4I1 | ENSG00000104951 | 16.36 | 0.23 | 0.0048 | 0.0354 | 1.85 |
| IL7 | ENSG00000104432 | 24.04 | 0.20 | 0.0106 | 0.0427 | 1.47 |
| ILDR1 | ENSG00000145103 | 47.73 | 0.30 | <.0001 | 0.0342 | 0.90 |
| ILF2 | ENSG00000143621 | 93.66 | 0.19 | 0.0083 | 0.0395 | 2.40 |
| ILVBL | ENSG00000105135 | 88.23 | 0.21 | 0.0042 | 0.0350 | 1.49 |
| IMMP1L | ENSG00000148950 | 18.45 | 0.22 | 0.0045 | 0.0352 | 1.47 |
| IMMT | ENSG00000132305 | 149.31 | 0.18 | 0.0104 | 0.0424 | 1.62 |
| IMP3 | ENSG00000177971 | 55.80 | 0.20 | 0.0060 | 0.0366 | 1.55 |
| IMPA1 | ENSG00000133731 | 101.37 | 0.21 | 0.0065 | 0.0371 | 0.69 |
| IMPACT | ENSG00000154059 | 41.53 | 0.25 | 0.0010 | 0.0342 | 1.62 |
| IMPAD1 | ENSG00000104331 | 171.43 | 0.20 | 0.0068 | 0.0374 | 2.00 |
| IMPDH2 | ENSG00000178035 | 169.26 | 0.19 | 0.0097 | 0.0415 | 2.60 |
| INADL | ENSG00000132849 | 609.30 | 0.21 | 0.0037 | 0.0342 | 1.33 |
| INCA1 | ENSG00000196388 | 15.07 | 0.23 | 0.0039 | 0.0345 | 0.89 |
| INCENP | ENSG00000149503 | 47.83 | 0.20 | 0.0083 | 0.0395 | 2.48 |
| INF2 | ENSG00000203485 | 471.75 | 0.21 | 0.0063 | 0.0370 | 1.29 |
| ING1 | ENSG00000153487 | 26.25 | 0.21 | 0.0051 | 0.0358 | 2.29 |
| ING4 | ENSG00000111653 | 23.87 | 0.20 | 0.0072 | 0.0377 | 1.47 |
| ING5 | ENSG00000168395 | 110.21 | 0.22 | 0.0030 | 0.0342 | 1.31 |
| INHBA | ENSG00000122641 | 18.81 | 0.21 | 0.0129 | 0.0457 | 15.62 |
| INO80D | ENSG00000114933 | 371.18 | 0.18 | 0.0131 | 0.0460 | 1.47 |
| INO80E | ENSG00000169592 | 156.30 | 0.19 | 0.0095 | 0.0413 | 1.05 |
| INPP5E | ENSG00000148384 | 49.39 | 0.19 | 0.0125 | 0.0451 | 1.51 |
| INPP5F | ENSG00000198825 | 72.22 | 0.18 | 0.0153 | 0.0489 | 1.39 |
| INPPL1 | ENSG00000165458 | 271.39 | 0.19 | 0.0098 | 0.0416 | 1.58 |
| INSIG2 | ENSG00000125629 | 38.47 | 0.21 | 0.0057 | 0.0363 | 2.27 |
| INSR | ENSG00000171105 | 459.48 | 0.24 | 0.0011 | 0.0342 | 1.21 |
| INTS1 | ENSG00000164880 | 316.26 | 0.21 | 0.0055 | 0.0363 | 2.07 |
| INTS10 | ENSG00000104613 | 110.25 | 0.18 | 0.0161 | 0.0500 | 1.51 |
| INTS3 | ENSG00000143624 | 273.85 | 0.22 | 0.0029 | 0.0342 | 1.72 |
| INTS5 | ENSG00000185085 | 34.40 | 0.19 | 0.0092 | 0.0408 | 1.82 |
| INTS7 | ENSG00000143493 | 46.74 | 0.19 | 0.0080 | 0.0389 | 2.32 |
| INTS8 | ENSG00000164941 | 123.84 | 0.27 | 0.0002 | 0.0342 | 2.02 |
| INTS9 | ENSG00000104299 | 30.51 | 0.19 | 0.0128 | 0.0456 | 1.33 |
| IP6K1 | ENSG00000176095 | 91.22 | 0.21 | 0.0052 | 0.0359 | 1.50 |
| IP6K2 | ENSG00000068745 | 220.14 | 0.26 | 0.0010 | 0.0342 | 1.14 |
| IPMK | ENSG00000151151 | 56.05 | 0.21 | 0.0065 | 0.0371 | 1.42 |
| IPO5 | ENSG00000065150 | 187.51 | 0.19 | 0.0102 | 0.0422 | 3.78 |
| IPO7 | ENSG00000205339 | 202.37 | 0.19 | 0.0104 | 0.0424 | 3.06 |
| IPO8 | ENSG00000133704 | 151.66 | 0.21 | 0.0022 | 0.0342 | 1.58 |
| IPO9 | ENSG00000198700 | 182.63 | 0.18 | 0.0153 | 0.0489 | 1.97 |
| IPP | ENSG00000197429 | 45.64 | 0.27 | 0.0003 | 0.0342 | 1.25 |
| IQCE | ENSG00000106012 | 131.30 | 0.26 | 0.0005 | 0.0342 | 1.87 |
| IQGAP1 | ENSG00000140575 | 896.12 | 0.21 | 0.0032 | 0.0342 | 1.48 |
| IQGAP2 | ENSG00000145703 | 260.45 | 0.19 | 0.0104 | 0.0424 | 0.61 |
| IQGAP3 | ENSG00000183856 | 93.43 | 0.23 | 0.0040 | 0.0346 | 4.02 |
| IQSEC2 | ENSG00000124313 | 76.86 | 0.21 | 0.0053 | 0.0361 | 1.03 |
| IRAK1 | ENSG00000184216 | 150.93 | 0.19 | 0.0137 | 0.0467 | 3.16 |
| IREB2 | ENSG00000136381 | 145.14 | 0.20 | 0.0082 | 0.0393 | 1.62 |
| IRF2 | ENSG00000168310 | 68.36 | 0.21 | 0.0041 | 0.0349 | 1.25 |
| IRF2BP2 | ENSG00000168264 | 411.85 | 0.26 | 0.0004 | 0.0342 | 2.28 |
| IRF2BPL | ENSG00000119669 | 66.02 | 0.20 | 0.0115 | 0.0438 | 1.33 |
| IRF3 | ENSG00000126456 | 113.57 | 0.21 | 0.0063 | 0.0370 | 1.33 |
| IRS1 | ENSG00000169047 | 154.42 | 0.28 | 0.0002 | 0.0342 | 1.92 |
| ISCA2 | ENSG00000165898 | 15.35 | 0.18 | 0.0139 | 0.0469 | 1.17 |
| ISCU | ENSG00000136003 | 97.20 | 0.18 | 0.0122 | 0.0446 | 1.32 |
| IST1 | ENSG00000182149 | 265.00 | 0.19 | 0.0094 | 0.0411 | 1.36 |
| ISX | ENSG00000175329 | 154.17 | 0.23 | 0.0020 | 0.0342 | 0.42 |
| ISY1 | ENSG00000240682 | 90.03 | 0.18 | 0.0141 | 0.0472 | 1.60 |
| ITCH | ENSG00000078747 | 226.91 | 0.20 | 0.0047 | 0.0353 | 2.03 |
| ITFG1 | ENSG00000129636 | 85.67 | 0.18 | 0.0134 | 0.0463 | 1.53 |
| ITFG3 | ENSG00000167930 | 141.09 | 0.18 | 0.0130 | 0.0458 | 1.80 |
| ITGA1 | ENSG00000213949 | 305.11 | 0.21 | 0.0065 | 0.0371 | 1.62 |
| ITGA11 | ENSG00000137809 | 32.13 | 0.20 | 0.0109 | 0.0431 | 5.66 |
| ITGA3 | ENSG00000005884 | 326.53 | 0.25 | 0.0007 | 0.0342 | 1.04 |
| ITGAV | ENSG00000138448 | 183.58 | 0.20 | 0.0078 | 0.0386 | 2.42 |
| ITGB1 | ENSG00000150093 | 437.69 | 0.18 | 0.0151 | 0.0487 | 2.06 |
| ITGB1BP1 | ENSG00000119185 | 133.95 | 0.24 | 0.0014 | 0.0342 | 1.41 |
| ITGB4 | ENSG00000132470 | 500.75 | 0.28 | 0.0003 | 0.0342 | 1.91 |
| ITGB5 | ENSG00000082781 | 123.57 | 0.23 | 0.0009 | 0.0342 | 2.44 |
| ITGB6 | ENSG00000115221 | 62.14 | 0.30 | 0.0002 | 0.0342 | 1.54 |
| ITM2B | ENSG00000136156 | 205.15 | 0.19 | 0.0102 | 0.0422 | 1.39 |
| ITM2C | ENSG00000135916 | 1671.62 | 0.22 | 0.0024 | 0.0342 | 0.30 |
| ITPA | ENSG00000125877 | 30.34 | 0.19 | 0.0117 | 0.0439 | 2.38 |
| ITPK1 | ENSG00000100605 | 206.53 | 0.22 | 0.0026 | 0.0342 | 1.25 |
| ITPKA | ENSG00000137825 | 36.54 | 0.29 | 0.0004 | 0.0342 | 0.45 |
| ITPR3 | ENSG00000096433 | 708.33 | 0.20 | 0.0072 | 0.0377 | 1.67 |
| ITPRIPL2 | ENSG00000205730 | 222.86 | 0.21 | 0.0054 | 0.0362 | 2.03 |
| IVD | ENSG00000128928 | 133.64 | 0.22 | 0.0035 | 0.0342 | 1.18 |
| IVNS1ABP | ENSG00000116679 | 613.04 | 0.24 | 0.0018 | 0.0342 | 0.94 |
| IYD | ENSG00000009765 | 132.37 | 0.27 | 0.0002 | 0.0342 | 1.50 |
| JAG1 | ENSG00000101384 | 309.67 | 0.20 | 0.0059 | 0.0365 | 1.30 |
| JAG2 | ENSG00000184916 | 30.01 | 0.25 | 0.0031 | 0.0342 | 5.55 |
| JAGN1 | ENSG00000171135 | 26.80 | 0.24 | 0.0020 | 0.0342 | 1.62 |
| JAK1 | ENSG00000162434 | 358.82 | 0.19 | 0.0089 | 0.0403 | 1.48 |
| JAK2 | ENSG00000096968 | 102.16 | 0.20 | 0.0097 | 0.0415 | 1.10 |
| JHDM1D | ENSG00000006459 | 390.30 | 0.20 | 0.0061 | 0.0368 | 1.82 |
| JKAMP | ENSG00000050130 | 33.51 | 0.24 | 0.0013 | 0.0342 | 1.37 |
| JMJD4 | ENSG00000081692 | 46.28 | 0.22 | 0.0052 | 0.0359 | 1.64 |
| JMJD8 | ENSG00000161999 | 85.17 | 0.20 | 0.0080 | 0.0389 | 1.75 |
| JPH1 | ENSG00000104369 | 43.50 | 0.18 | 0.0120 | 0.0444 | 2.74 |
| JRK | ENSG00000234616 | 114.57 | 0.23 | 0.0019 | 0.0342 | 1.91 |
| JRKL | ENSG00000183340 | 36.53 | 0.21 | 0.0048 | 0.0354 | 1.81 |
| JTB | ENSG00000143543 | 93.31 | 0.23 | 0.0016 | 0.0342 | 1.70 |
| JUB | ENSG00000129474 | 14.03 | 0.24 | 0.0079 | 0.0387 | 6.85 |
| JUP | ENSG00000173801 | 487.91 | 0.20 | 0.0081 | 0.0391 | 1.96 |
| KAL1 | ENSG00000011201 | 6.61 | 0.23 | 0.0035 | 0.0342 | 7.47 |
| KALRN | ENSG00000160145 | 160.11 | 0.18 | 0.0119 | 0.0442 | 1.26 |
| KANK1 | ENSG00000107104 | 224.73 | 0.21 | 0.0036 | 0.0342 | 1.62 |
| KARS | ENSG00000065427 | 98.93 | 0.24 | 0.0026 | 0.0342 | 2.73 |
| KAT6B | ENSG00000156650 | 181.15 | 0.19 | 0.0089 | 0.0403 | 1.04 |
| KAT7 | ENSG00000136504 | 116.89 | 0.18 | 0.0126 | 0.0452 | 1.36 |
| KAZALD1 | ENSG00000107821 | 30.11 | 0.18 | 0.0159 | 0.0498 | 1.13 |
| KBTBD3 | ENSG00000182359 | 8.91 | 0.21 | 0.0058 | 0.0364 | 1.26 |
| KBTBD6 | ENSG00000165572 | 16.15 | 0.20 | 0.0056 | 0.0363 | 2.81 |
| KCNJ2 | ENSG00000123700 | 43.32 | 0.21 | 0.0057 | 0.0363 | 1.35 |
| KCNK6 | ENSG00000099337 | 72.87 | 0.21 | 0.0039 | 0.0345 | 0.95 |
| KCNQ1 | ENSG00000053918 | 109.66 | 0.19 | 0.0137 | 0.0467 | 2.32 |
| KCTD1 | ENSG00000134504 | 19.50 | 0.23 | 0.0016 | 0.0342 | 0.98 |
| KCTD12 | ENSG00000178695 | 235.15 | 0.21 | 0.0050 | 0.0356 | 0.78 |
| KCTD14 | ENSG00000151364 | 16.14 | 0.28 | 0.0004 | 0.0342 | 2.94 |
| KCTD15 | ENSG00000153885 | 49.30 | 0.21 | 0.0049 | 0.0355 | 1.21 |
| KCTD18 | ENSG00000155729 | 39.39 | 0.20 | 0.0075 | 0.0382 | 1.43 |
| KCTD2 | ENSG00000180901 | 53.20 | 0.21 | 0.0036 | 0.0342 | 1.46 |
| KCTD3 | ENSG00000136636 | 130.07 | 0.23 | 0.0018 | 0.0342 | 1.77 |
| KCTD7 | ENSG00000243335 | 126.92 | 0.19 | 0.0088 | 0.0402 | 1.48 |
| KDELR1 | ENSG00000105438 | 202.29 | 0.22 | 0.0022 | 0.0342 | 1.61 |
| KDELR2 | ENSG00000136240 | 356.69 | 0.22 | 0.0022 | 0.0342 | 1.63 |
| KDELR3 | ENSG00000100196 | 58.35 | 0.22 | 0.0036 | 0.0342 | 1.29 |
| KDM1A | ENSG00000004487 | 115.55 | 0.19 | 0.0092 | 0.0408 | 2.53 |
| KDM1B | ENSG00000165097 | 97.10 | 0.19 | 0.0118 | 0.0441 | 2.26 |
| KDM3A | ENSG00000115548 | 200.61 | 0.23 | 0.0008 | 0.0342 | 1.81 |
| KDM3B | ENSG00000120733 | 408.82 | 0.22 | 0.0044 | 0.0351 | 1.50 |
| KDM4A | ENSG00000066135 | 151.68 | 0.21 | 0.0033 | 0.0342 | 1.19 |
| KDM4B | ENSG00000127663 | 121.05 | 0.19 | 0.0114 | 0.0437 | 1.39 |
| KDM4C | ENSG00000107077 | 237.12 | 0.20 | 0.0061 | 0.0368 | 1.12 |
| KDM5A | ENSG00000073614 | 361.34 | 0.18 | 0.0160 | 0.0499 | 1.49 |
| KDM5B | ENSG00000117139 | 240.34 | 0.20 | 0.0053 | 0.0361 | 1.62 |
| KDM5C | ENSG00000126012 | 342.64 | 0.20 | 0.0058 | 0.0364 | 1.74 |
| KDM6A | ENSG00000147050 | 184.44 | 0.18 | 0.0124 | 0.0450 | 1.10 |
| KEAP1 | ENSG00000079999 | 54.36 | 0.23 | 0.0013 | 0.0342 | 2.04 |
| KEL | ENSG00000197993 | 4.25 | -0.21 | 0.0094 | 0.0411 | 1.15 |
| KHDRBS1 | ENSG00000121774 | 198.75 | 0.19 | 0.0108 | 0.0429 | 2.03 |
| KHNYN | ENSG00000100441 | 391.89 | 0.20 | 0.0079 | 0.0387 | 1.23 |
| KHSRP | ENSG00000088247 | 202.41 | 0.19 | 0.0095 | 0.0413 | 2.42 |
| KIAA0100 | ENSG00000007202 | 592.42 | 0.22 | 0.0024 | 0.0342 | 1.71 |
| KIAA0141 | ENSG00000081791 | 200.85 | 0.18 | 0.0127 | 0.0454 | 1.04 |
| KIAA0182 | ENSG00000131149 | 365.99 | 0.21 | 0.0044 | 0.0351 | 1.83 |
| KIAA0195 | ENSG00000177728 | 221.39 | 0.23 | 0.0017 | 0.0342 | 1.32 |
| KIAA0196 | ENSG00000164961 | 91.12 | 0.21 | 0.0046 | 0.0352 | 2.27 |
| KIAA0240 | ENSG00000112624 | 166.15 | 0.19 | 0.0102 | 0.0422 | 1.28 |
| KIAA0317 | ENSG00000119682 | 151.23 | 0.21 | 0.0027 | 0.0342 | 1.21 |
| KIAA0319L | ENSG00000142687 | 293.61 | 0.21 | 0.0051 | 0.0358 | 1.21 |
| KIAA0368 | ENSG00000136813 | 398.75 | 0.23 | 0.0016 | 0.0342 | 1.51 |
| KIAA0415 | ENSG00000242802 | 92.77 | 0.18 | 0.0159 | 0.0498 | 1.91 |
| KIAA0430 | ENSG00000166783 | 283.62 | 0.19 | 0.0058 | 0.0364 | 1.38 |
| KIAA0494 | ENSG00000159658 | 399.59 | 0.20 | 0.0053 | 0.0361 | 1.46 |
| KIAA0513 | ENSG00000135709 | 159.40 | 0.20 | 0.0076 | 0.0382 | 0.56 |
| KIAA0528 | ENSG00000111731 | 185.86 | 0.19 | 0.0117 | 0.0439 | 1.37 |
| KIAA0556 | ENSG00000047578 | 174.97 | 0.19 | 0.0110 | 0.0432 | 1.08 |
| KIAA0564 | ENSG00000102763 | 157.21 | 0.20 | 0.0064 | 0.0370 | 1.96 |
| KIAA0586 | ENSG00000100578 | 78.18 | 0.19 | 0.0098 | 0.0416 | 1.45 |
| KIAA0753 | ENSG00000198920 | 67.50 | 0.18 | 0.0116 | 0.0439 | 1.47 |
| KIAA0895 | ENSG00000164542 | 12.80 | 0.26 | 0.0021 | 0.0342 | 3.35 |
| KIAA0895L | ENSG00000196123 | 81.77 | 0.20 | 0.0076 | 0.0382 | 1.23 |
| KIAA0907 | ENSG00000132680 | 249.41 | 0.22 | 0.0031 | 0.0342 | 1.61 |
| KIAA0913 | ENSG00000214655 | 233.15 | 0.19 | 0.0106 | 0.0427 | 1.04 |
| KIAA1009 | ENSG00000135315 | 34.49 | 0.22 | 0.0037 | 0.0342 | 1.79 |
| KIAA1107 | ENSG00000069712 | 19.53 | 0.21 | 0.0067 | 0.0373 | 1.68 |
| KIAA1109 | ENSG00000138688 | 917.69 | 0.17 | 0.0160 | 0.0499 | 1.06 |
| KIAA1161 | ENSG00000164976 | 225.52 | 0.26 | 0.0005 | 0.0342 | 0.87 |
| KIAA1191 | ENSG00000122203 | 132.38 | 0.19 | 0.0107 | 0.0428 | 1.63 |
| KIAA1211 | ENSG00000109265 | 131.83 | 0.20 | 0.0062 | 0.0368 | 0.53 |
| KIAA1217 | ENSG00000120549 | 390.83 | 0.24 | 0.0019 | 0.0342 | 1.46 |
| KIAA1244 | ENSG00000112379 | 325.71 | 0.25 | 0.0010 | 0.0342 | 1.90 |
| KIAA1267 | ENSG00000120071 | 342.91 | 0.20 | 0.0057 | 0.0363 | 1.21 |
| KIAA1324 | ENSG00000116299 | 315.73 | 0.19 | 0.0119 | 0.0442 | 0.90 |
| KIAA1429 | ENSG00000164944 | 111.21 | 0.19 | 0.0087 | 0.0401 | 2.09 |
| KIAA1522 | ENSG00000162522 | 715.33 | 0.21 | 0.0042 | 0.0350 | 0.89 |
| KIAA1530 | ENSG00000163945 | 159.73 | 0.25 | 0.0013 | 0.0342 | 0.99 |
| KIAA1549 | ENSG00000122778 | 46.51 | 0.19 | 0.0149 | 0.0485 | 5.02 |
| KIAA1598 | ENSG00000187164 | 127.23 | 0.20 | 0.0055 | 0.0363 | 1.60 |
| KIAA1609 | ENSG00000140950 | 55.71 | 0.19 | 0.0118 | 0.0441 | 1.82 |
| KIAA1671 | ENSG00000197077 | 646.69 | 0.20 | 0.0080 | 0.0389 | 1.05 |
| KIAA1737 | ENSG00000198894 | 112.88 | 0.21 | 0.0064 | 0.0370 | 1.05 |
| KIAA1797 | ENSG00000188352 | 119.43 | 0.20 | 0.0074 | 0.0380 | 2.26 |
| KIAA1875 | ENSG00000179698 | 13.82 | 0.20 | 0.0078 | 0.0386 | 2.15 |
| KIAA1919 | ENSG00000173214 | 110.74 | 0.23 | 0.0010 | 0.0342 | 1.33 |
| KIAA1958 | ENSG00000165185 | 61.86 | 0.22 | 0.0059 | 0.0365 | 2.45 |
| KIAA1967 | ENSG00000158941 | 218.78 | 0.18 | 0.0116 | 0.0439 | 1.31 |
| KIAA1984 | ENSG00000213213 | 107.18 | 0.24 | 0.0032 | 0.0342 | 1.97 |
| KIAA2013 | ENSG00000116685 | 104.74 | 0.23 | 0.0013 | 0.0342 | 1.25 |
| KIAA2018 | ENSG00000176542 | 335.53 | 0.18 | 0.0122 | 0.0446 | 1.10 |
| KIDINS220 | ENSG00000134313 | 330.61 | 0.20 | 0.0049 | 0.0355 | 1.41 |
| KIF12 | ENSG00000136883 | 45.11 | 0.23 | 0.0028 | 0.0342 | 2.34 |
| KIF13A | ENSG00000137177 | 320.97 | 0.25 | 0.0008 | 0.0342 | 1.15 |
| KIF13B | ENSG00000197892 | 768.27 | 0.20 | 0.0058 | 0.0364 | 0.72 |
| KIF15 | ENSG00000163808 | 22.38 | 0.22 | 0.0038 | 0.0342 | 3.41 |
| KIF16B | ENSG00000089177 | 196.23 | 0.23 | 0.0022 | 0.0342 | 1.29 |
| KIF1C | ENSG00000129250 | 520.50 | 0.22 | 0.0036 | 0.0342 | 0.93 |
| KIF20B | ENSG00000138182 | 48.18 | 0.21 | 0.0090 | 0.0405 | 3.29 |
| KIF21A | ENSG00000139116 | 103.55 | 0.21 | 0.0049 | 0.0355 | 1.56 |
| KIF2A | ENSG00000068796 | 102.20 | 0.21 | 0.0044 | 0.0351 | 2.06 |
| KIF2C | ENSG00000142945 | 16.50 | 0.22 | 0.0072 | 0.0377 | 4.24 |
| KIF3B | ENSG00000101350 | 211.45 | 0.24 | 0.0012 | 0.0342 | 2.34 |
| KIF4A | ENSG00000090889 | 22.99 | 0.19 | 0.0119 | 0.0442 | 4.23 |
| KIF5B | ENSG00000170759 | 564.43 | 0.25 | 0.0013 | 0.0342 | 2.73 |
| KIFC1 | ENSG00000237649 | 31.60 | 0.22 | 0.0036 | 0.0342 | 3.08 |
| KIFC2 | ENSG00000167702 | 151.42 | 0.21 | 0.0063 | 0.0370 | 1.49 |
| KIN | ENSG00000151657 | 26.55 | 0.19 | 0.0108 | 0.0429 | 1.37 |
| KITLG | ENSG00000049130 | 102.21 | 0.22 | 0.0038 | 0.0342 | 1.14 |
| KLC2 | ENSG00000174996 | 51.79 | 0.19 | 0.0144 | 0.0477 | 1.60 |
| KLC4 | ENSG00000137171 | 136.47 | 0.24 | 0.0010 | 0.0342 | 1.07 |
| KLF5 | ENSG00000102554 | 908.92 | 0.23 | 0.0017 | 0.0342 | 1.39 |
| KLHDC2 | ENSG00000165516 | 72.13 | 0.22 | 0.0021 | 0.0342 | 1.13 |
| KLHDC3 | ENSG00000124702 | 59.13 | 0.20 | 0.0072 | 0.0377 | 1.91 |
| KLHDC4 | ENSG00000104731 | 43.86 | 0.18 | 0.0150 | 0.0486 | 1.40 |
| KLHDC7A | ENSG00000179023 | 7.64 | 0.19 | 0.0150 | 0.0486 | 0.82 |
| KLHL11 | ENSG00000178502 | 55.56 | 0.18 | 0.0154 | 0.0491 | 2.22 |
| KLHL12 | ENSG00000117153 | 74.50 | 0.21 | 0.0036 | 0.0342 | 1.58 |
| KLHL14 | ENSG00000197705 | 7.84 | -0.21 | 0.0096 | 0.0414 | 0.46 |
| KLHL17 | ENSG00000187961 | 19.01 | 0.23 | 0.0046 | 0.0352 | 1.84 |
| KLHL2 | ENSG00000109466 | 29.97 | 0.20 | 0.0084 | 0.0397 | 1.54 |
| KLHL20 | ENSG00000076321 | 46.49 | 0.20 | 0.0041 | 0.0349 | 1.21 |
| KLHL23 | ENSG00000213160 | 45.79 | 0.22 | 0.0038 | 0.0342 | 3.02 |
| KLHL24 | ENSG00000114796 | 118.78 | 0.23 | 0.0013 | 0.0342 | 1.63 |
| KLHL25 | ENSG00000183655 | 34.97 | 0.21 | 0.0064 | 0.0370 | 1.52 |
| KLHL33 | ENSG00000185271 | 3.70 | 0.19 | 0.0135 | 0.0464 | 1.26 |
| KLHL35 | ENSG00000149243 | 14.24 | 0.20 | 0.0132 | 0.0460 | 1.87 |
| KLHL36 | ENSG00000135686 | 161.19 | 0.20 | 0.0070 | 0.0376 | 1.52 |
| KLHL7 | ENSG00000122550 | 27.75 | 0.22 | 0.0030 | 0.0342 | 1.78 |
| KLHL8 | ENSG00000145332 | 57.98 | 0.30 | 0.0003 | 0.0342 | 1.44 |
| KLHL9 | ENSG00000198642 | 103.34 | 0.25 | 0.0004 | 0.0342 | 1.53 |
| KLK1 | ENSG00000167748 | 49.59 | 0.25 | 0.0018 | 0.0342 | 0.62 |
| KLKB1 | ENSG00000164344 | 15.60 | 0.24 | 0.0027 | 0.0342 | 0.83 |
| KPNA3 | ENSG00000102753 | 72.70 | 0.19 | 0.0092 | 0.0408 | 2.89 |
| KPNA6 | ENSG00000025800 | 248.82 | 0.19 | 0.0081 | 0.0391 | 1.53 |
| KPNB1 | ENSG00000108424 | 332.41 | 0.20 | 0.0089 | 0.0403 | 2.20 |
| KRAS | ENSG00000133703 | 211.37 | 0.19 | 0.0140 | 0.0471 | 1.09 |
| KRBA2 | ENSG00000184619 | 27.73 | 0.27 | 0.0006 | 0.0342 | 0.76 |
| KRCC1 | ENSG00000172086 | 72.75 | 0.25 | 0.0009 | 0.0342 | 1.13 |
| KRT18 | ENSG00000111057 | 283.41 | 0.19 | 0.0109 | 0.0431 | 2.85 |
| KRT19 | ENSG00000171345 | 726.65 | 0.19 | 0.0080 | 0.0389 | 0.90 |
| KRT80 | ENSG00000167767 | 7.72 | 0.20 | 0.0151 | 0.0487 | 13.39 |
| KRTAP5-1 | ENSG00000205869 | 5.40 | 0.20 | 0.0142 | 0.0474 | 2.29 |
| KRTAP5-2 | ENSG00000205867 | 7.52 | 0.26 | 0.0014 | 0.0342 | 1.37 |
| KRTAP5-4 | ENSG00000241598 | 4.44 | 0.25 | 0.0014 | 0.0342 | 1.67 |
| KRTCAP2 | ENSG00000163463 | 68.79 | 0.25 | 0.0006 | 0.0342 | 1.53 |
| KRTCAP3 | ENSG00000157992 | 31.67 | 0.22 | 0.0035 | 0.0342 | 1.84 |
| KSR1 | ENSG00000141068 | 117.74 | 0.19 | 0.0133 | 0.0462 | 1.40 |
| KTN1 | ENSG00000126777 | 759.85 | 0.20 | 0.0049 | 0.0355 | 1.44 |
| L3MBTL2 | ENSG00000100395 | 69.64 | 0.19 | 0.0098 | 0.0416 | 1.53 |
| LACTB | ENSG00000103642 | 20.96 | 0.18 | 0.0135 | 0.0464 | 1.43 |
| LACTB2 | ENSG00000147592 | 11.82 | 0.23 | 0.0041 | 0.0349 | 2.92 |
| LAD1 | ENSG00000159166 | 276.01 | 0.19 | 0.0076 | 0.0382 | 1.87 |
| LAMA5 | ENSG00000130702 | 291.88 | 0.19 | 0.0141 | 0.0472 | 2.41 |
| LAMB1 | ENSG00000091136 | 500.02 | 0.19 | 0.0077 | 0.0384 | 1.77 |
| LAMB2 | ENSG00000172037 | 166.41 | 0.19 | 0.0118 | 0.0441 | 1.94 |
| LAMP1 | ENSG00000185896 | 244.26 | 0.23 | 0.0009 | 0.0342 | 2.37 |
| LAMP2 | ENSG00000005893 | 85.07 | 0.21 | 0.0063 | 0.0370 | 2.57 |
| LAMTOR1 | ENSG00000149357 | 75.06 | 0.19 | 0.0104 | 0.0424 | 1.48 |
| LAMTOR2 | ENSG00000116586 | 14.51 | 0.22 | 0.0038 | 0.0342 | 2.03 |
| LAMTOR3 | ENSG00000109270 | 52.67 | 0.22 | 0.0027 | 0.0342 | 1.34 |
| LANCL1 | ENSG00000115365 | 68.02 | 0.19 | 0.0107 | 0.0428 | 1.92 |
| LANCL2 | ENSG00000132434 | 46.12 | 0.18 | 0.0138 | 0.0468 | 1.32 |
| LAP3 | ENSG00000002549 | 65.53 | 0.20 | 0.0142 | 0.0474 | 1.89 |
| LAPTM4A | ENSG00000068697 | 124.24 | 0.19 | 0.0079 | 0.0387 | 1.56 |
| LAPTM4B | ENSG00000104341 | 43.56 | 0.18 | 0.0157 | 0.0496 | 4.55 |
| LARGE | ENSG00000133424 | 145.54 | 0.19 | 0.0098 | 0.0416 | 0.86 |
| LARP1 | ENSG00000155506 | 618.90 | 0.21 | 0.0050 | 0.0356 | 2.16 |
| LARP4 | ENSG00000161813 | 175.03 | 0.20 | 0.0088 | 0.0402 | 1.95 |
| LARP4B | ENSG00000107929 | 310.88 | 0.19 | 0.0070 | 0.0376 | 1.61 |
| LARS | ENSG00000133706 | 215.78 | 0.23 | 0.0018 | 0.0342 | 1.89 |
| LARS2 | ENSG00000011376 | 77.45 | 0.18 | 0.0116 | 0.0439 | 2.16 |
| LASP1 | ENSG00000002834 | 567.25 | 0.22 | 0.0037 | 0.0342 | 1.45 |
| LATS1 | ENSG00000131023 | 167.57 | 0.18 | 0.0126 | 0.0452 | 1.47 |
| LBR | ENSG00000143815 | 171.11 | 0.19 | 0.0106 | 0.0427 | 2.66 |
| LBX2 | ENSG00000179528 | 20.79 | 0.22 | 0.0039 | 0.0345 | 1.92 |
| LCMT1 | ENSG00000205629 | 28.90 | 0.22 | 0.0031 | 0.0342 | 1.88 |
| LCN12 | ENSG00000184925 | 17.88 | 0.25 | 0.0020 | 0.0342 | 2.03 |
| LCN2 | ENSG00000148346 | 184.43 | 0.29 | 0.0035 | 0.0342 | 2.29 |
| LCOR | ENSG00000196233 | 342.92 | 0.25 | 0.0012 | 0.0342 | 1.36 |
| LDB1 | ENSG00000198728 | 101.88 | 0.20 | 0.0076 | 0.0382 | 1.44 |
| LDHA | ENSG00000134333 | 373.29 | 0.24 | 0.0035 | 0.0342 | 2.94 |
| LDHB | ENSG00000111716 | 85.03 | 0.19 | 0.0155 | 0.0492 | 4.07 |
| LDHD | ENSG00000166816 | 57.55 | 0.21 | 0.0058 | 0.0364 | 0.28 |
| LDLRAP1 | ENSG00000157978 | 148.13 | 0.22 | 0.0032 | 0.0342 | 1.07 |
| LDOC1L | ENSG00000188636 | 88.40 | 0.20 | 0.0075 | 0.0382 | 1.49 |
| LEFTY1 | ENSG00000243709 | 159.91 | 0.24 | 0.0042 | 0.0350 | 0.95 |
| LENG1 | ENSG00000105617 | 19.84 | 0.21 | 0.0087 | 0.0401 | 1.15 |
| LENG8 | ENSG00000167615 | 1276.62 | 0.19 | 0.0102 | 0.0422 | 1.16 |
| LEPREL4 | ENSG00000141696 | 22.31 | 0.27 | 0.0004 | 0.0342 | 2.23 |
| LEPROT | ENSG00000213625 | 164.75 | 0.22 | 0.0015 | 0.0342 | 1.41 |
| LEPROTL1 | ENSG00000104660 | 73.95 | 0.21 | 0.0066 | 0.0373 | 1.38 |
| LETMD1 | ENSG00000050426 | 100.99 | 0.24 | 0.0007 | 0.0342 | 1.56 |
| LGALS3BP | ENSG00000108679 | 392.11 | 0.22 | 0.0028 | 0.0342 | 1.40 |
| LGALS4 | ENSG00000171747 | 1291.38 | 0.19 | 0.0126 | 0.0452 | 0.59 |
| LGALS8 | ENSG00000116977 | 211.46 | 0.22 | 0.0033 | 0.0342 | 1.44 |
| LGALS9 | ENSG00000168961 | 185.51 | 0.21 | 0.0054 | 0.0362 | 1.06 |
| LGALS9B | ENSG00000170298 | 24.98 | 0.22 | 0.0051 | 0.0358 | 0.63 |
| LGALS9C | ENSG00000171916 | 31.04 | 0.21 | 0.0092 | 0.0408 | 0.61 |
| LGR4 | ENSG00000205213 | 307.42 | 0.24 | 0.0015 | 0.0342 | 0.80 |
| LHFPL2 | ENSG00000145685 | 220.87 | 0.18 | 0.0115 | 0.0438 | 1.08 |
| LHX4 | ENSG00000121454 | 13.92 | 0.19 | 0.0152 | 0.0489 | 1.26 |
| LIG4 | ENSG00000174405 | 35.83 | 0.19 | 0.0113 | 0.0435 | 1.47 |
| LIMA1 | ENSG00000050405 | 526.75 | 0.26 | 0.0004 | 0.0342 | 0.73 |
| LIMCH1 | ENSG00000064042 | 65.10 | 0.22 | 0.0024 | 0.0342 | 1.20 |
| LIMD1 | ENSG00000144791 | 326.13 | 0.22 | 0.0020 | 0.0342 | 1.28 |
| LIMK2 | ENSG00000182541 | 439.03 | 0.21 | 0.0038 | 0.0342 | 0.85 |
| LIPE | ENSG00000079435 | 20.22 | 0.18 | 0.0154 | 0.0491 | 1.67 |
| LIPH | ENSG00000163898 | 209.33 | 0.22 | 0.0025 | 0.0342 | 0.67 |
| LIPT1 | ENSG00000144182 | 10.30 | 0.22 | 0.0038 | 0.0342 | 1.48 |
| LL22NC03-75H12.2 | ENSG00000218357 | 5.42 | 0.23 | 0.0035 | 0.0342 | 0.30 |
| LLGL2 | ENSG00000073350 | 506.75 | 0.21 | 0.0051 | 0.0358 | 1.27 |
| LMAN1 | ENSG00000074695 | 193.57 | 0.20 | 0.0079 | 0.0387 | 1.47 |
| LMAN2 | ENSG00000169223 | 108.35 | 0.24 | 0.0009 | 0.0342 | 1.61 |
| LMAN2L | ENSG00000114988 | 30.51 | 0.24 | 0.0011 | 0.0342 | 1.66 |
| LMBR1 | ENSG00000105983 | 101.46 | 0.20 | 0.0092 | 0.0408 | 2.69 |
| LMBR1L | ENSG00000139636 | 59.30 | 0.21 | 0.0049 | 0.0355 | 1.39 |
| LMBRD1 | ENSG00000168216 | 42.84 | 0.21 | 0.0035 | 0.0342 | 1.37 |
| LMBRD2 | ENSG00000164187 | 93.09 | 0.21 | 0.0042 | 0.0350 | 1.36 |
| LMF1 | ENSG00000103227 | 41.02 | 0.18 | 0.0161 | 0.0500 | 1.11 |
| LMF2 | ENSG00000100258 | 88.19 | 0.21 | 0.0062 | 0.0368 | 1.26 |
| LMNB2 | ENSG00000176619 | 134.37 | 0.19 | 0.0124 | 0.0450 | 2.96 |
| LMO4 | ENSG00000143013 | 57.57 | 0.20 | 0.0055 | 0.0363 | 0.91 |
| LMTK2 | ENSG00000164715 | 337.39 | 0.23 | 0.0015 | 0.0342 | 1.33 |
| LNX1 | ENSG00000072201 | 58.30 | 0.22 | 0.0028 | 0.0342 | 1.24 |
| LNX2 | ENSG00000139517 | 83.12 | 0.21 | 0.0042 | 0.0350 | 2.47 |
| LONP2 | ENSG00000102910 | 345.19 | 0.19 | 0.0095 | 0.0413 | 1.44 |
| LPAR5 | ENSG00000184574 | 71.08 | 0.19 | 0.0099 | 0.0417 | 0.89 |
| LPCAT2 | ENSG00000087253 | 35.65 | 0.20 | 0.0078 | 0.0386 | 3.08 |
| LPCAT4 | ENSG00000176454 | 146.89 | 0.21 | 0.0033 | 0.0342 | 0.67 |
| LPGAT1 | ENSG00000123684 | 200.49 | 0.23 | 0.0031 | 0.0342 | 2.48 |
| LPHN1 | ENSG00000072071 | 116.45 | 0.21 | 0.0059 | 0.0365 | 1.84 |
| LPIN2 | ENSG00000101577 | 210.55 | 0.20 | 0.0064 | 0.0370 | 1.07 |
| LPIN3 | ENSG00000132793 | 182.87 | 0.27 | 0.0002 | 0.0342 | 1.56 |
| LRBA | ENSG00000198589 | 758.37 | 0.17 | 0.0150 | 0.0486 | 1.31 |
| LRCH1 | ENSG00000136141 | 87.93 | 0.19 | 0.0121 | 0.0445 | 2.49 |
| LRFN3 | ENSG00000126243 | 20.21 | 0.23 | 0.0018 | 0.0342 | 1.58 |
| LRG1 | ENSG00000171236 | 21.68 | 0.20 | 0.0069 | 0.0375 | 1.36 |
| LRIG2 | ENSG00000198799 | 131.08 | 0.19 | 0.0067 | 0.0373 | 1.20 |
| LRIG3 | ENSG00000139263 | 144.16 | 0.25 | 0.0009 | 0.0342 | 1.02 |
| LRMP | ENSG00000118308 | 34.27 | -0.22 | 0.0073 | 0.0379 | 0.40 |
| LRP1 | ENSG00000123384 | 1419.86 | 0.19 | 0.0099 | 0.0417 | 1.50 |
| LRP10 | ENSG00000197324 | 481.93 | 0.23 | 0.0026 | 0.0342 | 1.10 |
| LRP11 | ENSG00000120256 | 66.58 | 0.28 | 0.0002 | 0.0342 | 3.41 |
| LRP4 | ENSG00000134569 | 144.09 | 0.32 | 0.0003 | 0.0342 | 4.35 |
| LRP5 | ENSG00000162337 | 298.92 | 0.24 | 0.0015 | 0.0342 | 1.70 |
| LRP6 | ENSG00000070018 | 234.64 | 0.23 | 0.0017 | 0.0342 | 1.67 |
| LRPAP1 | ENSG00000163956 | 140.93 | 0.18 | 0.0114 | 0.0437 | 1.19 |
| LRRC1 | ENSG00000137269 | 125.63 | 0.22 | 0.0022 | 0.0342 | 1.05 |
| LRRC14 | ENSG00000160959 | 99.81 | 0.20 | 0.0096 | 0.0414 | 1.60 |
| LRRC16A | ENSG00000079691 | 222.59 | 0.20 | 0.0051 | 0.0358 | 1.28 |
| LRRC19 | ENSG00000184434 | 133.89 | 0.22 | 0.0029 | 0.0342 | 0.46 |
| LRRC20 | ENSG00000172731 | 9.86 | 0.23 | 0.0044 | 0.0351 | 4.04 |
| LRRC26 | ENSG00000184709 | 29.20 | 0.20 | 0.0102 | 0.0422 | 0.46 |
| LRRC28 | ENSG00000168904 | 40.60 | 0.19 | 0.0098 | 0.0416 | 1.22 |
| LRRC31 | ENSG00000114248 | 62.38 | 0.28 | 0.0004 | 0.0342 | 0.83 |
| LRRC37B | ENSG00000185158 | 30.74 | 0.21 | 0.0067 | 0.0373 | 1.16 |
| LRRC42 | ENSG00000116212 | 18.02 | 0.19 | 0.0129 | 0.0457 | 1.99 |
| LRRC45 | ENSG00000169683 | 32.27 | 0.19 | 0.0150 | 0.0486 | 2.09 |
| LRRC46 | ENSG00000141294 | 7.90 | 0.20 | 0.0111 | 0.0433 | 1.59 |
| LRRC47 | ENSG00000130764 | 66.46 | 0.21 | 0.0046 | 0.0352 | 1.60 |
| LRRC57 | ENSG00000180979 | 33.71 | 0.23 | 0.0023 | 0.0342 | 1.39 |
| LRRC66 | ENSG00000188993 | 28.06 | 0.33 | <.0001 | 0.0342 | 0.72 |
| LRRC8D | ENSG00000171492 | 69.26 | 0.27 | 0.0005 | 0.0342 | 1.61 |
| LRRCC1 | ENSG00000133739 | 56.21 | 0.19 | 0.0097 | 0.0415 | 1.92 |
| LRRD1 | ENSG00000240720 | 108.78 | 0.19 | 0.0121 | 0.0445 | 2.48 |
| LRRFIP2 | ENSG00000093167 | 303.78 | 0.19 | 0.0101 | 0.0421 | 0.90 |
| LRSAM1 | ENSG00000148356 | 64.06 | 0.26 | 0.0010 | 0.0342 | 1.81 |
| LSM1 | ENSG00000175324 | 22.79 | 0.21 | 0.0078 | 0.0386 | 1.87 |
| LSM11 | ENSG00000155858 | 40.96 | 0.21 | 0.0077 | 0.0384 | 1.51 |
| LSM14B | ENSG00000149657 | 79.18 | 0.20 | 0.0055 | 0.0363 | 2.39 |
| LSM4 | ENSG00000130520 | 52.25 | 0.24 | 0.0012 | 0.0342 | 2.32 |
| LSM5 | ENSG00000106355 | 30.71 | 0.21 | 0.0065 | 0.0371 | 2.56 |
| LSR | ENSG00000105699 | 243.59 | 0.21 | 0.0048 | 0.0354 | 1.44 |
| LSS | ENSG00000160285 | 155.77 | 0.20 | 0.0091 | 0.0407 | 1.80 |
| LTA4H | ENSG00000111144 | 82.87 | 0.22 | 0.0018 | 0.0342 | 2.20 |
| LTBR | ENSG00000111321 | 207.77 | 0.22 | 0.0027 | 0.0342 | 1.57 |
| LTK | ENSG00000062524 | 49.32 | 0.26 | 0.0013 | 0.0342 | 0.44 |
| LUC7L3 | ENSG00000108848 | 359.45 | 0.18 | 0.0144 | 0.0477 | 1.43 |
| LUZP1 | ENSG00000169641 | 255.35 | 0.18 | 0.0135 | 0.0464 | 1.30 |
| LXN | ENSG00000079257 | 33.79 | 0.22 | 0.0026 | 0.0342 | 1.10 |
| LY6G6E | ENSG00000204422 | 64.88 | 0.20 | 0.0074 | 0.0380 | 1.94 |
| LY75 | ENSG00000054219 | 205.68 | 0.19 | 0.0086 | 0.0399 | 1.93 |
| LY75-CD302 | ENSG00000248672 | 149.91 | 0.19 | 0.0075 | 0.0382 | 1.90 |
| LYPD1 | ENSG00000150551 | 13.74 | 0.20 | 0.0059 | 0.0365 | 1.60 |
| LYPD6 | ENSG00000187123 | 14.02 | 0.20 | 0.0108 | 0.0429 | 2.35 |
| LYPLA1 | ENSG00000120992 | 62.10 | 0.22 | 0.0043 | 0.0351 | 2.38 |
| LYRM1 | ENSG00000102897 | 27.53 | 0.21 | 0.0037 | 0.0342 | 2.07 |
| LYRM2 | ENSG00000083099 | 69.37 | 0.20 | 0.0067 | 0.0373 | 1.33 |
| LZIC | ENSG00000162441 | 55.44 | 0.24 | 0.0020 | 0.0342 | 1.56 |
| LZTFL1 | ENSG00000163818 | 35.13 | 0.20 | 0.0073 | 0.0379 | 1.92 |
| LZTR1 | ENSG00000099949 | 260.74 | 0.20 | 0.0096 | 0.0414 | 1.19 |
| LZTS2 | ENSG00000107816 | 117.45 | 0.20 | 0.0101 | 0.0421 | 1.66 |
| M6PR | ENSG00000003056 | 105.35 | 0.18 | 0.0123 | 0.0448 | 1.93 |
| MAB21L3 | ENSG00000173212 | 11.39 | 0.26 | 0.0009 | 0.0342 | 1.35 |
| MACC1 | ENSG00000183742 | 107.60 | 0.23 | 0.0052 | 0.0359 | 5.85 |
| MACF1 | ENSG00000127603 | 1956.73 | 0.18 | 0.0115 | 0.0438 | 1.34 |
| MAD2L1 | ENSG00000164109 | 8.36 | 0.19 | 0.0135 | 0.0464 | 4.32 |
| MAD2L2 | ENSG00000116670 | 10.98 | 0.22 | 0.0057 | 0.0363 | 2.41 |
| MADD | ENSG00000110514 | 245.41 | 0.20 | 0.0055 | 0.0363 | 1.37 |
| MAEA | ENSG00000090316 | 160.31 | 0.20 | 0.0056 | 0.0363 | 1.06 |
| MAF1 | ENSG00000179632 | 87.18 | 0.19 | 0.0112 | 0.0434 | 1.72 |
| MAGEA3 | ENSG00000221867 | 0.98 | 0.23 | 0.0107 | 0.0428 | 50.18 |
| MAGED2 | ENSG00000102316 | 64.69 | 0.20 | 0.0060 | 0.0366 | 2.37 |
| MAGEF1 | ENSG00000177383 | 31.25 | 0.22 | 0.0023 | 0.0342 | 1.92 |
| MAGEH1 | ENSG00000187601 | 10.73 | 0.20 | 0.0117 | 0.0439 | 1.07 |
| MAGI1 | ENSG00000151276 | 167.61 | 0.19 | 0.0127 | 0.0454 | 1.09 |
| MAGI3 | ENSG00000081026 | 262.40 | 0.24 | 0.0014 | 0.0342 | 0.80 |
| MAGT1 | ENSG00000102158 | 131.54 | 0.21 | 0.0057 | 0.0363 | 1.78 |
| MAL2 | ENSG00000147676 | 209.38 | 0.23 | 0.0016 | 0.0342 | 2.24 |
| MALL | ENSG00000144063 | 211.58 | 0.18 | 0.0125 | 0.0451 | 0.36 |
| MAMDC4 | ENSG00000177943 | 231.31 | 0.20 | 0.0079 | 0.0387 | 1.03 |
| MAML1 | ENSG00000161021 | 225.85 | 0.20 | 0.0085 | 0.0399 | 1.29 |
| MAML3 | ENSG00000196782 | 100.09 | 0.20 | 0.0074 | 0.0380 | 0.98 |
| MAN1A2 | ENSG00000198162 | 234.30 | 0.18 | 0.0124 | 0.0450 | 1.41 |
| MAN1B1 | ENSG00000177239 | 97.38 | 0.21 | 0.0052 | 0.0359 | 1.71 |
| MAN2A1 | ENSG00000112893 | 291.45 | 0.20 | 0.0066 | 0.0373 | 1.13 |
| MAN2A2 | ENSG00000196547 | 228.58 | 0.19 | 0.0133 | 0.0462 | 1.22 |
| MAN2B2 | ENSG00000013288 | 140.37 | 0.22 | 0.0028 | 0.0342 | 1.25 |
| MAN2C1 | ENSG00000140400 | 213.46 | 0.19 | 0.0098 | 0.0416 | 1.22 |
| MANBA | ENSG00000109323 | 66.81 | 0.18 | 0.0159 | 0.0498 | 1.52 |
| MANBAL | ENSG00000101363 | 41.01 | 0.23 | 0.0021 | 0.0342 | 2.11 |
| MANEAL | ENSG00000185090 | 21.86 | 0.19 | 0.0147 | 0.0482 | 2.35 |
| MANSC1 | ENSG00000111261 | 50.64 | 0.18 | 0.0152 | 0.0489 | 2.00 |
| MAOA | ENSG00000189221 | 390.85 | 0.24 | 0.0008 | 0.0342 | 0.58 |
| MAP1LC3A | ENSG00000101460 | 40.42 | 0.20 | 0.0096 | 0.0414 | 1.67 |
| MAP2K2 | ENSG00000126934 | 144.71 | 0.20 | 0.0082 | 0.0393 | 1.36 |
| MAP2K4 | ENSG00000065559 | 63.87 | 0.20 | 0.0086 | 0.0399 | 1.20 |
| MAP2K5 | ENSG00000137764 | 59.72 | 0.19 | 0.0117 | 0.0439 | 1.18 |
| MAP2K6 | ENSG00000108984 | 68.06 | 0.25 | 0.0009 | 0.0342 | 0.90 |
| MAP3K11 | ENSG00000173327 | 240.59 | 0.19 | 0.0108 | 0.0429 | 1.07 |
| MAP3K12 | ENSG00000139625 | 106.83 | 0.20 | 0.0085 | 0.0399 | 1.10 |
| MAP3K13 | ENSG00000073803 | 171.81 | 0.20 | 0.0054 | 0.0362 | 1.19 |
| MAP3K15 | ENSG00000180815 | 23.84 | 0.20 | 0.0123 | 0.0448 | 1.21 |
| MAP3K5 | ENSG00000197442 | 132.55 | 0.18 | 0.0131 | 0.0460 | 1.34 |
| MAP3K9 | ENSG00000006432 | 45.49 | 0.18 | 0.0127 | 0.0454 | 1.47 |
| MAP4 | ENSG00000047849 | 567.47 | 0.18 | 0.0140 | 0.0471 | 1.72 |
| MAP7 | ENSG00000135525 | 161.49 | 0.21 | 0.0041 | 0.0349 | 1.83 |
| MAPK1 | ENSG00000100030 | 330.31 | 0.18 | 0.0138 | 0.0468 | 1.34 |
| MAPK12 | ENSG00000188130 | 82.28 | 0.22 | 0.0048 | 0.0354 | 1.57 |
| MAPK13 | ENSG00000156711 | 117.78 | 0.24 | 0.0012 | 0.0342 | 1.53 |
| MAPK14 | ENSG00000112062 | 209.82 | 0.25 | 0.0007 | 0.0342 | 1.65 |
| MAPK3 | ENSG00000102882 | 174.58 | 0.22 | 0.0033 | 0.0342 | 1.03 |
| MAPK6 | ENSG00000069956 | 114.79 | 0.19 | 0.0113 | 0.0435 | 1.23 |
| MAPK8IP3 | ENSG00000138834 | 252.88 | 0.19 | 0.0138 | 0.0468 | 1.39 |
| MAPKAP1 | ENSG00000119487 | 156.08 | 0.18 | 0.0108 | 0.0429 | 1.66 |
| MAPKAPK5 | ENSG00000089022 | 86.29 | 0.23 | 0.0009 | 0.0342 | 1.74 |
| MAPKBP1 | ENSG00000137802 | 89.31 | 0.18 | 0.0159 | 0.0498 | 1.43 |
| MARCH2 | ENSG00000099785 | 35.76 | 0.21 | 0.0052 | 0.0359 | 1.18 |
| MARCH3 | ENSG00000173926 | 56.94 | 0.18 | 0.0135 | 0.0464 | 0.86 |
| MARCH5 | ENSG00000198060 | 38.27 | 0.20 | 0.0058 | 0.0364 | 1.89 |
| MARCKS | ENSG00000155130 | 506.60 | 0.20 | 0.0066 | 0.0373 | 0.99 |
| MARCKSL1 | ENSG00000175130 | 183.49 | 0.22 | 0.0048 | 0.0354 | 2.19 |
| MARK2 | ENSG00000072518 | 243.23 | 0.21 | 0.0043 | 0.0351 | 1.28 |
| MARK3 | ENSG00000075413 | 134.30 | 0.18 | 0.0158 | 0.0497 | 1.32 |
| MARK4 | ENSG00000007047 | 48.61 | 0.20 | 0.0087 | 0.0401 | 1.62 |
| MARS | ENSG00000166986 | 126.43 | 0.19 | 0.0084 | 0.0397 | 1.88 |
| MARVELD1 | ENSG00000155254 | 87.75 | 0.27 | 0.0008 | 0.0342 | 1.59 |
| MARVELD2 | ENSG00000152939 | 98.17 | 0.27 | 0.0002 | 0.0342 | 1.50 |
| MARVELD3 | ENSG00000140832 | 102.78 | 0.21 | 0.0041 | 0.0349 | 0.88 |
| MAST2 | ENSG00000086015 | 423.79 | 0.19 | 0.0101 | 0.0421 | 1.01 |
| MAST3 | ENSG00000099308 | 166.56 | 0.21 | 0.0064 | 0.0370 | 0.87 |
| MAT2B | ENSG00000038274 | 81.25 | 0.21 | 0.0045 | 0.0352 | 1.43 |
| MATR3 | ENSG00000015479 | 500.30 | 0.19 | 0.0117 | 0.0439 | 1.89 |
| MAU2 | ENSG00000129933 | 187.38 | 0.22 | 0.0028 | 0.0342 | 1.45 |
| MAVS | ENSG00000088888 | 400.78 | 0.19 | 0.0123 | 0.0448 | 1.15 |
| MAX | ENSG00000125952 | 127.93 | 0.27 | 0.0005 | 0.0342 | 1.28 |
| MAZ | ENSG00000103495 | 335.11 | 0.20 | 0.0078 | 0.0386 | 1.64 |
| MBD1 | ENSG00000141644 | 132.97 | 0.19 | 0.0093 | 0.0409 | 0.98 |
| MBD3 | ENSG00000071655 | 99.37 | 0.19 | 0.0117 | 0.0439 | 1.71 |
| MBD6 | ENSG00000166987 | 251.81 | 0.18 | 0.0159 | 0.0498 | 1.29 |
| MBNL2 | ENSG00000139793 | 175.78 | 0.18 | 0.0135 | 0.0464 | 1.79 |
| MBNL3 | ENSG00000076770 | 199.75 | 0.18 | 0.0122 | 0.0446 | 1.09 |
| MBOAT1 | ENSG00000172197 | 158.07 | 0.21 | 0.0041 | 0.0349 | 0.72 |
| MBOAT2 | ENSG00000143797 | 130.46 | 0.21 | 0.0071 | 0.0376 | 1.63 |
| MBOAT7 | ENSG00000125505 | 180.68 | 0.20 | 0.0056 | 0.0363 | 1.31 |
| MBP | ENSG00000197971 | 166.09 | 0.19 | 0.0096 | 0.0414 | 0.89 |
| MBTD1 | ENSG00000011258 | 101.65 | 0.19 | 0.0116 | 0.0439 | 1.73 |
| MBTPS1 | ENSG00000140943 | 257.13 | 0.19 | 0.0073 | 0.0379 | 1.37 |
| MBTPS2 | ENSG00000012174 | 40.97 | 0.20 | 0.0078 | 0.0386 | 2.31 |
| MCAT | ENSG00000100294 | 15.42 | 0.20 | 0.0109 | 0.0431 | 1.77 |
| MCCC2 | ENSG00000131844 | 144.71 | 0.22 | 0.0029 | 0.0342 | 1.88 |
| MCM3AP | ENSG00000160294 | 371.91 | 0.21 | 0.0052 | 0.0359 | 1.41 |
| MCM9 | ENSG00000111877 | 85.98 | 0.19 | 0.0063 | 0.0370 | 1.22 |
| MCOLN1 | ENSG00000090674 | 21.68 | 0.20 | 0.0098 | 0.0416 | 1.21 |
| MCRS1 | ENSG00000187778 | 55.13 | 0.20 | 0.0066 | 0.0373 | 1.69 |
| MCTP2 | ENSG00000140563 | 299.42 | 0.20 | 0.0069 | 0.0375 | 0.70 |
| MCTS1 | ENSG00000232119 | 18.97 | 0.18 | 0.0127 | 0.0454 | 2.51 |
| MCU | ENSG00000156026 | 131.57 | 0.19 | 0.0084 | 0.0397 | 1.11 |
| MDH1 | ENSG00000014641 | 88.24 | 0.20 | 0.0049 | 0.0355 | 1.45 |
| MDH2 | ENSG00000146701 | 137.13 | 0.20 | 0.0078 | 0.0386 | 2.47 |
| MDK | ENSG00000110492 | 46.97 | 0.26 | 0.0007 | 0.0342 | 2.89 |
| ME1 | ENSG00000065833 | 27.53 | 0.19 | 0.0113 | 0.0435 | 2.34 |
| MEA1 | ENSG00000124733 | 36.08 | 0.20 | 0.0068 | 0.0374 | 2.11 |
| MECOM | ENSG00000085276 | 335.49 | 0.19 | 0.0104 | 0.0424 | 1.16 |
| MED10 | ENSG00000133398 | 20.44 | 0.22 | 0.0034 | 0.0342 | 1.88 |
| MED12 | ENSG00000184634 | 197.41 | 0.18 | 0.0150 | 0.0486 | 1.67 |
| MED13 | ENSG00000108510 | 509.67 | 0.18 | 0.0145 | 0.0479 | 1.81 |
| MED13L | ENSG00000123066 | 492.47 | 0.19 | 0.0066 | 0.0373 | 1.39 |
| MED14 | ENSG00000180182 | 231.32 | 0.21 | 0.0042 | 0.0350 | 2.00 |
| MED16 | ENSG00000175221 | 91.86 | 0.19 | 0.0091 | 0.0407 | 1.26 |
| MED19 | ENSG00000156603 | 20.13 | 0.19 | 0.0108 | 0.0429 | 1.24 |
| MED20 | ENSG00000124641 | 33.54 | 0.18 | 0.0155 | 0.0492 | 1.69 |
| MED22 | ENSG00000148297 | 83.38 | 0.24 | 0.0011 | 0.0342 | 1.67 |
| MED23 | ENSG00000112282 | 139.85 | 0.22 | 0.0024 | 0.0342 | 1.36 |
| MED24 | ENSG00000008838 | 120.03 | 0.18 | 0.0125 | 0.0451 | 1.82 |
| MED29 | ENSG00000063322 | 70.46 | 0.19 | 0.0092 | 0.0408 | 1.56 |
| MED4 | ENSG00000136146 | 50.61 | 0.20 | 0.0086 | 0.0399 | 2.54 |
| MED8 | ENSG00000159479 | 41.83 | 0.22 | 0.0047 | 0.0353 | 1.38 |
| MEGF11 | ENSG00000157890 | 53.38 | 0.19 | 0.0135 | 0.0464 | 0.80 |
| MEGF6 | ENSG00000162591 | 49.84 | 0.21 | 0.0079 | 0.0387 | 3.74 |
| MEGF8 | ENSG00000105429 | 182.23 | 0.21 | 0.0062 | 0.0368 | 1.33 |
| MEN1 | ENSG00000133895 | 53.86 | 0.19 | 0.0124 | 0.0450 | 1.67 |
| MEP1A | ENSG00000112818 | 170.39 | 0.28 | 0.0003 | 0.0342 | 0.60 |
| MERTK | ENSG00000153208 | 50.17 | 0.19 | 0.0091 | 0.0407 | 1.30 |
| MESDC2 | ENSG00000117899 | 81.09 | 0.18 | 0.0140 | 0.0471 | 1.73 |
| MEST | ENSG00000106484 | 57.63 | 0.23 | 0.0015 | 0.0342 | 3.50 |
| MET | ENSG00000105976 | 140.01 | 0.20 | 0.0132 | 0.0460 | 5.55 |
| METRN | ENSG00000103260 | 11.41 | 0.21 | 0.0107 | 0.0428 | 1.74 |
| METTL10 | ENSG00000203791 | 46.98 | 0.22 | 0.0035 | 0.0342 | 1.29 |
| METTL13 | ENSG00000010165 | 58.63 | 0.22 | 0.0021 | 0.0342 | 2.14 |
| METTL14 | ENSG00000145388 | 31.04 | 0.26 | 0.0004 | 0.0342 | 1.49 |
| METTL15 | ENSG00000169519 | 29.19 | 0.19 | 0.0100 | 0.0419 | 1.21 |
| METTL17 | ENSG00000165792 | 77.07 | 0.18 | 0.0137 | 0.0467 | 1.18 |
| METTL21B | ENSG00000123427 | 18.81 | 0.23 | 0.0033 | 0.0342 | 2.07 |
| METTL23 | ENSG00000181038 | 17.40 | 0.19 | 0.0087 | 0.0401 | 1.71 |
| METTL2B | ENSG00000165055 | 72.42 | 0.19 | 0.0131 | 0.0460 | 1.83 |
| METTL4 | ENSG00000101574 | 25.55 | 0.21 | 0.0054 | 0.0362 | 1.17 |
| METTL5 | ENSG00000138382 | 29.66 | 0.18 | 0.0148 | 0.0484 | 2.83 |
| MEX3D | ENSG00000181588 | 36.45 | 0.19 | 0.0126 | 0.0452 | 2.93 |
| MFAP1 | ENSG00000140259 | 24.51 | 0.23 | 0.0022 | 0.0342 | 1.63 |
| MFAP3 | ENSG00000037749 | 86.54 | 0.20 | 0.0059 | 0.0365 | 1.54 |
| MFF | ENSG00000168958 | 106.48 | 0.21 | 0.0044 | 0.0351 | 1.63 |
| MFHAS1 | ENSG00000147324 | 187.03 | 0.24 | 0.0016 | 0.0342 | 1.45 |
| MFI2 | ENSG00000163975 | 53.19 | 0.27 | 0.0015 | 0.0342 | 3.76 |
| MFN1 | ENSG00000171109 | 137.26 | 0.18 | 0.0144 | 0.0477 | 1.42 |
| MFN2 | ENSG00000116688 | 246.10 | 0.21 | 0.0043 | 0.0351 | 1.10 |
| MFSD1 | ENSG00000118855 | 106.61 | 0.18 | 0.0119 | 0.0442 | 1.30 |
| MFSD11 | ENSG00000092931 | 45.46 | 0.18 | 0.0146 | 0.0481 | 1.15 |
| MFSD4 | ENSG00000174514 | 133.91 | 0.25 | 0.0019 | 0.0342 | 0.36 |
| MFSD5 | ENSG00000182544 | 23.64 | 0.18 | 0.0117 | 0.0439 | 1.58 |
| MFSD6 | ENSG00000151690 | 269.34 | 0.20 | 0.0067 | 0.0373 | 1.23 |
| MFSD6L | ENSG00000185156 | 15.28 | 0.20 | 0.0064 | 0.0370 | 0.38 |
| MFSD9 | ENSG00000135953 | 58.33 | 0.24 | 0.0017 | 0.0342 | 1.60 |
| MGAT1 | ENSG00000131446 | 208.06 | 0.18 | 0.0134 | 0.0463 | 1.20 |
| MGAT2 | ENSG00000168282 | 68.21 | 0.23 | 0.0019 | 0.0342 | 1.23 |
| MGAT3 | ENSG00000128268 | 42.96 | 0.22 | 0.0040 | 0.0346 | 0.94 |
| MGAT4A | ENSG00000071073 | 320.33 | 0.20 | 0.0070 | 0.0376 | 0.96 |
| MGAT4B | ENSG00000161013 | 457.26 | 0.20 | 0.0067 | 0.0373 | 1.38 |
| MGAT5 | ENSG00000152127 | 293.71 | 0.22 | 0.0031 | 0.0342 | 3.22 |
| MGEA5 | ENSG00000198408 | 674.92 | 0.18 | 0.0133 | 0.0462 | 1.28 |
| MGLL | ENSG00000074416 | 853.03 | 0.25 | 0.0011 | 0.0342 | 0.59 |
| MGST2 | ENSG00000085871 | 51.31 | 0.22 | 0.0022 | 0.0342 | 1.21 |
| MGST3 | ENSG00000143198 | 115.85 | 0.24 | 0.0012 | 0.0342 | 1.07 |
| MIA | ENSG00000167578 | 42.46 | 0.21 | 0.0053 | 0.0361 | 0.85 |
| MIA3 | ENSG00000154305 | 318.01 | 0.21 | 0.0038 | 0.0342 | 1.20 |
| MIB1 | ENSG00000101752 | 179.69 | 0.23 | 0.0019 | 0.0342 | 1.67 |
| MIB2 | ENSG00000197530 | 171.34 | 0.19 | 0.0140 | 0.0471 | 0.99 |
| MICA | ENSG00000204520 | 65.70 | 0.22 | 0.0023 | 0.0342 | 0.98 |
| MICAL3 | ENSG00000243156 | 367.10 | 0.20 | 0.0056 | 0.0363 | 1.03 |
| MICALL1 | ENSG00000100139 | 98.53 | 0.22 | 0.0042 | 0.0350 | 1.44 |
| MICALL2 | ENSG00000164877 | 286.38 | 0.24 | 0.0023 | 0.0342 | 1.26 |
| MICU1 | ENSG00000107745 | 74.85 | 0.21 | 0.0040 | 0.0346 | 1.30 |
| MID1 | ENSG00000101871 | 119.43 | 0.21 | 0.0056 | 0.0363 | 1.52 |
| MIIP | ENSG00000116691 | 26.59 | 0.19 | 0.0124 | 0.0450 | 2.18 |
| MINK1 | ENSG00000141503 | 466.68 | 0.19 | 0.0093 | 0.0409 | 1.09 |
| MIR4763 | ENSG00000197182 | 141.58 | 0.22 | 0.0046 | 0.0352 | 1.74 |
| MIS18A | ENSG00000159055 | 11.21 | 0.19 | 0.0098 | 0.0416 | 2.42 |
| MITD1 | ENSG00000158411 | 36.40 | 0.23 | 0.0017 | 0.0342 | 1.68 |
| MKKS | ENSG00000125863 | 45.67 | 0.21 | 0.0047 | 0.0353 | 1.69 |
| MKL2 | ENSG00000186260 | 212.60 | 0.18 | 0.0119 | 0.0442 | 1.33 |
| MKNK1 | ENSG00000079277 | 164.53 | 0.20 | 0.0081 | 0.0391 | 0.94 |
| MKNK2 | ENSG00000099875 | 447.63 | 0.21 | 0.0055 | 0.0363 | 1.08 |
| MKRN1 | ENSG00000133606 | 292.21 | 0.28 | <.0001 | 0.0342 | 1.37 |
| MKRN2 | ENSG00000075975 | 56.13 | 0.21 | 0.0050 | 0.0356 | 1.72 |
| MKS1 | ENSG00000011143 | 16.58 | 0.22 | 0.0054 | 0.0362 | 2.02 |
| MLEC | ENSG00000110917 | 1115.65 | 0.26 | 0.0011 | 0.0342 | 1.91 |
| MLH3 | ENSG00000119684 | 173.27 | 0.23 | 0.0029 | 0.0342 | 1.33 |
| MLL2 | ENSG00000167548 | 1023.81 | 0.20 | 0.0098 | 0.0416 | 1.40 |
| MLL3 | ENSG00000055609 | 1203.93 | 0.18 | 0.0125 | 0.0451 | 1.30 |
| MLLT1 | ENSG00000130382 | 126.44 | 0.18 | 0.0134 | 0.0463 | 1.96 |
| MLLT3 | ENSG00000171843 | 130.39 | 0.21 | 0.0048 | 0.0354 | 1.74 |
| MLLT4 | ENSG00000130396 | 757.24 | 0.23 | 0.0020 | 0.0342 | 1.53 |
| MLLT6 | ENSG00000108292 | 602.18 | 0.18 | 0.0148 | 0.0484 | 2.01 |
| MLPH | ENSG00000115648 | 332.20 | 0.25 | 0.0018 | 0.0342 | 0.76 |
| MLST8 | ENSG00000167965 | 29.13 | 0.23 | 0.0023 | 0.0342 | 2.09 |
| MLX | ENSG00000108788 | 89.79 | 0.20 | 0.0048 | 0.0354 | 1.23 |
| MLXIP | ENSG00000175727 | 1129.81 | 0.18 | 0.0136 | 0.0466 | 0.99 |
| MLXIPL | ENSG00000009950 | 38.62 | 0.26 | 0.0030 | 0.0342 | 5.74 |
| MLYCD | ENSG00000103150 | 28.79 | 0.19 | 0.0106 | 0.0427 | 1.33 |
| MMAA | ENSG00000151611 | 36.10 | 0.20 | 0.0073 | 0.0379 | 1.13 |
| MMACHC | ENSG00000132763 | 37.17 | 0.18 | 0.0150 | 0.0486 | 1.82 |
| MMADHC | ENSG00000168288 | 44.91 | 0.23 | 0.0016 | 0.0342 | 2.20 |
| MMEL1 | ENSG00000142606 | 16.14 | 0.20 | 0.0077 | 0.0384 | 1.85 |
| MMGT1 | ENSG00000169446 | 63.29 | 0.19 | 0.0097 | 0.0415 | 1.98 |
| MMP14 | ENSG00000157227 | 162.47 | 0.21 | 0.0066 | 0.0373 | 3.68 |
| MMP15 | ENSG00000102996 | 214.67 | 0.22 | 0.0066 | 0.0373 | 1.05 |
| MMP24 | ENSG00000125966 | 86.22 | 0.18 | 0.0114 | 0.0437 | 1.38 |
| MMP28 | ENSG00000129270 | 79.60 | 0.21 | 0.0027 | 0.0342 | 0.16 |
| MMS19 | ENSG00000155229 | 170.95 | 0.19 | 0.0089 | 0.0403 | 1.42 |
| MNX1 | ENSG00000130675 | 44.48 | 0.19 | 0.0113 | 0.0435 | 1.30 |
| MOB2 | ENSG00000182208 | 69.45 | 0.21 | 0.0040 | 0.0346 | 1.10 |
| MOB3B | ENSG00000120162 | 171.29 | 0.24 | 0.0007 | 0.0342 | 0.64 |
| MOGAT2 | ENSG00000166391 | 169.71 | 0.28 | 0.0003 | 0.0342 | 0.31 |
| MOGAT3 | ENSG00000106384 | 48.46 | 0.22 | 0.0037 | 0.0342 | 1.41 |
| MOGS | ENSG00000115275 | 132.98 | 0.19 | 0.0092 | 0.0408 | 1.38 |
| MON1B | ENSG00000103111 | 171.54 | 0.18 | 0.0115 | 0.0438 | 1.31 |
| MORC2 | ENSG00000133422 | 83.18 | 0.22 | 0.0031 | 0.0342 | 2.22 |
| MORC4 | ENSG00000133131 | 33.76 | 0.22 | 0.0088 | 0.0402 | 2.95 |
| MORF4L1 | ENSG00000185787 | 113.16 | 0.20 | 0.0044 | 0.0351 | 1.48 |
| MORN1 | ENSG00000116151 | 32.42 | 0.21 | 0.0081 | 0.0391 | 0.99 |
| MOSC1 | ENSG00000186205 | 32.57 | 0.25 | 0.0013 | 0.0342 | 1.82 |
| MOSC2 | ENSG00000117791 | 29.81 | 0.22 | 0.0022 | 0.0342 | 0.85 |
| MOV10 | ENSG00000155363 | 149.09 | 0.19 | 0.0113 | 0.0435 | 1.31 |
| MPDU1 | ENSG00000129255 | 80.18 | 0.22 | 0.0036 | 0.0342 | 0.93 |
| MPG | ENSG00000103152 | 26.46 | 0.22 | 0.0025 | 0.0342 | 2.10 |
| MPHOSPH10 | ENSG00000124383 | 44.20 | 0.18 | 0.0141 | 0.0472 | 2.11 |
| MPHOSPH8 | ENSG00000196199 | 165.34 | 0.18 | 0.0145 | 0.0479 | 1.68 |
| MPI | ENSG00000178802 | 56.84 | 0.26 | 0.0008 | 0.0342 | 1.00 |
| MPND | ENSG00000008382 | 51.20 | 0.21 | 0.0056 | 0.0363 | 0.93 |
| MPP1 | ENSG00000130830 | 33.99 | 0.18 | 0.0133 | 0.0462 | 1.90 |
| MPP5 | ENSG00000072415 | 136.61 | 0.19 | 0.0087 | 0.0401 | 1.08 |
| MPPE1 | ENSG00000154889 | 57.53 | 0.24 | 0.0013 | 0.0342 | 0.79 |
| MPRIP | ENSG00000133030 | 381.09 | 0.20 | 0.0076 | 0.0382 | 1.27 |
| MPST | ENSG00000128309 | 186.78 | 0.25 | 0.0015 | 0.0342 | 1.37 |
| MPV17 | ENSG00000115204 | 55.11 | 0.18 | 0.0155 | 0.0492 | 1.79 |
| MPZL1 | ENSG00000197965 | 288.20 | 0.22 | 0.0034 | 0.0342 | 1.79 |
| MPZL2 | ENSG00000149573 | 92.30 | 0.22 | 0.0032 | 0.0342 | 0.98 |
| MRAP2 | ENSG00000135324 | 16.58 | 0.29 | 0.0002 | 0.0342 | 0.98 |
| MRFAP1 | ENSG00000179010 | 121.88 | 0.18 | 0.0135 | 0.0464 | 1.81 |
| MRFAP1L1 | ENSG00000178988 | 61.83 | 0.19 | 0.0110 | 0.0432 | 1.57 |
| MRPL10 | ENSG00000159111 | 27.97 | 0.24 | 0.0019 | 0.0342 | 2.09 |
| MRPL13 | ENSG00000172172 | 24.73 | 0.23 | 0.0009 | 0.0342 | 2.39 |
| MRPL15 | ENSG00000137547 | 22.06 | 0.19 | 0.0108 | 0.0429 | 2.41 |
| MRPL16 | ENSG00000166902 | 31.95 | 0.21 | 0.0042 | 0.0350 | 1.84 |
| MRPL18 | ENSG00000112110 | 26.91 | 0.20 | 0.0060 | 0.0366 | 2.36 |
| MRPL19 | ENSG00000115364 | 65.71 | 0.18 | 0.0126 | 0.0452 | 2.11 |
| MRPL2 | ENSG00000112651 | 32.38 | 0.22 | 0.0035 | 0.0342 | 1.37 |
| MRPL20 | ENSG00000242485 | 77.16 | 0.20 | 0.0063 | 0.0370 | 1.54 |
| MRPL3 | ENSG00000114686 | 60.12 | 0.21 | 0.0065 | 0.0371 | 2.66 |
| MRPL30 | ENSG00000185414 | 53.65 | 0.22 | 0.0037 | 0.0342 | 2.01 |
| MRPL33 | ENSG00000243147 | 29.92 | 0.19 | 0.0096 | 0.0414 | 1.66 |
| MRPL34 | ENSG00000130312 | 19.78 | 0.21 | 0.0057 | 0.0363 | 1.59 |
| MRPL35 | ENSG00000132313 | 89.01 | 0.21 | 0.0062 | 0.0368 | 1.25 |
| MRPL37 | ENSG00000116221 | 62.05 | 0.22 | 0.0038 | 0.0342 | 2.25 |
| MRPL38 | ENSG00000204316 | 44.09 | 0.20 | 0.0091 | 0.0407 | 1.74 |
| MRPL42 | ENSG00000198015 | 36.45 | 0.20 | 0.0093 | 0.0409 | 2.43 |
| MRPL43 | ENSG00000055950 | 124.87 | 0.27 | 0.0003 | 0.0342 | 0.72 |
| MRPL44 | ENSG00000135900 | 23.05 | 0.20 | 0.0069 | 0.0375 | 1.83 |
| MRPL45 | ENSG00000174100 | 27.61 | 0.19 | 0.0094 | 0.0411 | 2.67 |
| MRPL47 | ENSG00000136522 | 19.72 | 0.21 | 0.0046 | 0.0352 | 2.45 |
| MRPL50 | ENSG00000136897 | 29.79 | 0.20 | 0.0117 | 0.0439 | 2.30 |
| MRPL53 | ENSG00000204822 | 24.05 | 0.27 | 0.0009 | 0.0342 | 1.51 |
| MRPS10 | ENSG00000048544 | 32.19 | 0.19 | 0.0119 | 0.0442 | 1.89 |
| MRPS11 | ENSG00000181991 | 21.88 | 0.18 | 0.0136 | 0.0466 | 1.20 |
| MRPS12 | ENSG00000128626 | 14.15 | 0.18 | 0.0135 | 0.0464 | 2.50 |
| MRPS16 | ENSG00000182180 | 115.01 | 0.19 | 0.0119 | 0.0442 | 1.83 |
| MRPS18B | ENSG00000204568 | 41.37 | 0.24 | 0.0008 | 0.0342 | 2.01 |
| MRPS2 | ENSG00000122140 | 34.57 | 0.23 | 0.0013 | 0.0342 | 2.31 |
| MRPS22 | ENSG00000175110 | 49.25 | 0.20 | 0.0065 | 0.0371 | 1.65 |
| MRPS24 | ENSG00000062582 | 27.06 | 0.22 | 0.0038 | 0.0342 | 2.39 |
| MRPS30 | ENSG00000112996 | 106.14 | 0.24 | 0.0018 | 0.0342 | 2.11 |
| MRPS31 | ENSG00000102738 | 25.11 | 0.21 | 0.0055 | 0.0363 | 2.20 |
| MRPS35 | ENSG00000061794 | 54.63 | 0.22 | 0.0029 | 0.0342 | 2.57 |
| MRPS5 | ENSG00000144029 | 96.26 | 0.22 | 0.0030 | 0.0342 | 1.64 |
| MRRF | ENSG00000148187 | 70.02 | 0.19 | 0.0116 | 0.0439 | 1.57 |
| MRS2 | ENSG00000124532 | 66.45 | 0.20 | 0.0050 | 0.0356 | 1.69 |
| MRTO4 | ENSG00000053372 | 38.95 | 0.26 | 0.0006 | 0.0342 | 2.10 |
| MS4A1 | ENSG00000156738 | 78.83 | -0.28 | 0.0018 | 0.0342 | 0.15 |
| MSH2 | ENSG00000095002 | 47.28 | 0.20 | 0.0085 | 0.0399 | 2.54 |
| MSH3 | ENSG00000113318 | 87.82 | 0.19 | 0.0069 | 0.0375 | 1.34 |
| MSH5 | ENSG00000204410 | 112.41 | 0.22 | 0.0023 | 0.0342 | 2.06 |
| MSH5-C6orf26 | ENSG00000255152 | 79.76 | 0.22 | 0.0044 | 0.0351 | 2.05 |
| MSI2 | ENSG00000153944 | 90.05 | 0.21 | 0.0052 | 0.0359 | 2.46 |
| MSL1 | ENSG00000188895 | 212.21 | 0.24 | 0.0011 | 0.0342 | 1.70 |
| MSL2 | ENSG00000174579 | 124.46 | 0.20 | 0.0056 | 0.0363 | 1.61 |
| MSLN | ENSG00000102854 | 11.61 | 0.25 | 0.0021 | 0.0342 | 6.57 |
| MSMP | ENSG00000215183 | 53.57 | 0.21 | 0.0036 | 0.0342 | 1.02 |
| MSRB2 | ENSG00000148450 | 24.11 | 0.20 | 0.0046 | 0.0352 | 1.68 |
| MST1R | ENSG00000164078 | 277.18 | 0.27 | <.0001 | 0.0342 | 1.64 |
| MST4 | ENSG00000134602 | 28.82 | 0.21 | 0.0078 | 0.0386 | 2.65 |
| MT-CO1 | ENSG00000198804 | 31272.10 | 0.18 | 0.0150 | 0.0486 | 0.99 |
| MT1E | ENSG00000169715 | 69.64 | 0.18 | 0.0149 | 0.0485 | 0.28 |
| MTA2 | ENSG00000149480 | 142.06 | 0.20 | 0.0051 | 0.0358 | 1.70 |
| MTA3 | ENSG00000057935 | 112.69 | 0.19 | 0.0075 | 0.0382 | 1.26 |
| MTAP | ENSG00000099810 | 74.05 | 0.19 | 0.0157 | 0.0496 | 2.40 |
| MTCH2 | ENSG00000109919 | 115.13 | 0.18 | 0.0119 | 0.0442 | 1.89 |
| MTCP1NB | ENSG00000182712 | 10.81 | 0.19 | 0.0146 | 0.0481 | 2.11 |
| MTDH | ENSG00000147649 | 237.06 | 0.18 | 0.0132 | 0.0460 | 2.52 |
| MTFMT | ENSG00000103707 | 21.72 | 0.21 | 0.0048 | 0.0354 | 1.69 |
| MTG1 | ENSG00000148824 | 83.76 | 0.18 | 0.0156 | 0.0494 | 1.35 |
| MTHFD2 | ENSG00000065911 | 70.69 | 0.19 | 0.0161 | 0.0500 | 2.75 |
| MTHFSD | ENSG00000103248 | 37.84 | 0.22 | 0.0030 | 0.0342 | 1.39 |
| MTIF2 | ENSG00000085760 | 79.42 | 0.22 | 0.0050 | 0.0356 | 1.94 |
| MTIF3 | ENSG00000122033 | 41.70 | 0.18 | 0.0130 | 0.0458 | 2.98 |
| MTL5 | ENSG00000132749 | 22.98 | 0.23 | 0.0025 | 0.0342 | 1.50 |
| MTM1 | ENSG00000171100 | 85.99 | 0.21 | 0.0049 | 0.0355 | 0.82 |
| MTMR10 | ENSG00000166912 | 156.17 | 0.21 | 0.0046 | 0.0352 | 0.96 |
| MTMR11 | ENSG00000014914 | 337.67 | 0.23 | 0.0037 | 0.0342 | 0.99 |
| MTMR12 | ENSG00000150712 | 82.17 | 0.19 | 0.0089 | 0.0403 | 1.85 |
| MTMR3 | ENSG00000100330 | 277.06 | 0.20 | 0.0047 | 0.0353 | 1.03 |
| MTMR4 | ENSG00000108389 | 199.10 | 0.25 | 0.0006 | 0.0342 | 1.25 |
| MTMR7 | ENSG00000003987 | 27.36 | 0.20 | 0.0107 | 0.0428 | 0.83 |
| MTOR | ENSG00000198793 | 341.30 | 0.21 | 0.0047 | 0.0353 | 1.27 |
| MTPAP | ENSG00000107951 | 65.39 | 0.20 | 0.0069 | 0.0375 | 1.91 |
| MTPN | ENSG00000105887 | 171.58 | 0.19 | 0.0086 | 0.0399 | 2.11 |
| MTRF1L | ENSG00000112031 | 31.88 | 0.18 | 0.0112 | 0.0434 | 1.43 |
| MTRNR2L3 | ENSG00000256222 | 50.48 | 0.22 | 0.0036 | 0.0342 | 1.43 |
| MTUS1 | ENSG00000129422 | 613.06 | 0.22 | 0.0028 | 0.0342 | 0.64 |
| MTX2 | ENSG00000128654 | 35.02 | 0.19 | 0.0098 | 0.0416 | 1.82 |
| MUC1 | ENSG00000185499 | 575.97 | 0.20 | 0.0092 | 0.0408 | 0.74 |
| MUC13 | ENSG00000173702 | 1208.14 | 0.18 | 0.0153 | 0.0489 | 0.76 |
| MUC2 | ENSG00000198788 | 15847.43 | 0.19 | 0.0128 | 0.0456 | 0.19 |
| MUC20 | ENSG00000176945 | 336.29 | 0.27 | 0.0004 | 0.0342 | 1.05 |
| MUDENG | ENSG00000053770 | 105.74 | 0.18 | 0.0110 | 0.0432 | 1.00 |
| MUM1 | ENSG00000160953 | 111.87 | 0.23 | 0.0021 | 0.0342 | 1.44 |
| MUT | ENSG00000146085 | 56.12 | 0.22 | 0.0018 | 0.0342 | 1.26 |
| MUTED | ENSG00000188428 | 782.79 | 0.20 | 0.0049 | 0.0355 | 0.94 |
| MVP | ENSG00000013364 | 518.39 | 0.23 | 0.0017 | 0.0342 | 1.19 |
| MXD3 | ENSG00000213347 | 104.75 | 0.20 | 0.0057 | 0.0363 | 1.49 |
| MXD4 | ENSG00000123933 | 195.93 | 0.24 | 0.0024 | 0.0342 | 0.96 |
| MXI1 | ENSG00000119950 | 116.56 | 0.21 | 0.0037 | 0.0342 | 0.94 |
| MXRA8 | ENSG00000162576 | 100.83 | 0.22 | 0.0059 | 0.0365 | 1.79 |
| MYADML2 | ENSG00000185105 | 1.16 | 0.20 | 0.0150 | 0.0486 | 7.47 |
| MYB | ENSG00000118513 | 173.79 | 0.26 | 0.0006 | 0.0342 | 1.87 |
| MYBL1 | ENSG00000185697 | 24.46 | -0.20 | 0.0110 | 0.0432 | 0.77 |
| MYBPC1 | ENSG00000196091 | 10.96 | 0.22 | 0.0090 | 0.0405 | 1.44 |
| MYCBP | ENSG00000214114 | 59.58 | 0.24 | 0.0017 | 0.0342 | 1.54 |
| MYCL1 | ENSG00000116990 | 26.49 | 0.22 | 0.0030 | 0.0342 | 2.28 |
| MYD88 | ENSG00000172936 | 122.92 | 0.18 | 0.0138 | 0.0468 | 1.30 |
| MYEOV2 | ENSG00000172428 | 13.98 | 0.18 | 0.0137 | 0.0467 | 2.02 |
| MYH14 | ENSG00000105357 | 1031.32 | 0.22 | 0.0035 | 0.0342 | 1.34 |
| MYH9 | ENSG00000100345 | 2410.56 | 0.21 | 0.0047 | 0.0353 | 1.54 |
| MYL12A | ENSG00000101608 | 133.52 | 0.22 | 0.0018 | 0.0342 | 1.43 |
| MYL12B | ENSG00000118680 | 215.87 | 0.21 | 0.0055 | 0.0363 | 1.30 |
| MYLIP | ENSG00000007944 | 57.17 | 0.22 | 0.0027 | 0.0342 | 1.63 |
| MYLK | ENSG00000065534 | 561.34 | 0.20 | 0.0060 | 0.0366 | 0.64 |
| MYO10 | ENSG00000145555 | 426.21 | 0.24 | 0.0015 | 0.0342 | 1.70 |
| MYO18A | ENSG00000196535 | 530.02 | 0.21 | 0.0033 | 0.0342 | 1.20 |
| MYO19 | ENSG00000141140 | 161.02 | 0.19 | 0.0106 | 0.0427 | 1.71 |
| MYO1A | ENSG00000166866 | 351.75 | 0.25 | 0.0003 | 0.0342 | 0.58 |
| MYO1B | ENSG00000128641 | 182.23 | 0.23 | 0.0023 | 0.0342 | 2.19 |
| MYO1C | ENSG00000197879 | 497.27 | 0.23 | 0.0015 | 0.0342 | 1.02 |
| MYO1D | ENSG00000176658 | 845.08 | 0.21 | 0.0033 | 0.0342 | 1.19 |
| MYO5B | ENSG00000167306 | 472.77 | 0.23 | 0.0018 | 0.0342 | 1.18 |
| MYO5C | ENSG00000128833 | 335.75 | 0.22 | 0.0031 | 0.0342 | 1.09 |
| MYO6 | ENSG00000196586 | 310.45 | 0.26 | 0.0007 | 0.0342 | 1.75 |
| MYO7B | ENSG00000169994 | 813.71 | 0.25 | 0.0005 | 0.0342 | 0.93 |
| MYOF | ENSG00000138119 | 459.18 | 0.22 | 0.0035 | 0.0342 | 1.29 |
| MYOM3 | ENSG00000142661 | 19.70 | 0.21 | 0.0068 | 0.0374 | 7.53 |
| MZF1 | ENSG00000099326 | 79.05 | 0.25 | 0.0015 | 0.0342 | 1.05 |
| MZT1 | ENSG00000204899 | 10.01 | 0.23 | 0.0062 | 0.0368 | 4.89 |
| MZT2A | ENSG00000173272 | 33.61 | 0.22 | 0.0038 | 0.0342 | 2.31 |
| MZT2B | ENSG00000152082 | 32.19 | 0.19 | 0.0128 | 0.0456 | 1.90 |
| N4BP1 | ENSG00000102921 | 161.11 | 0.23 | 0.0017 | 0.0342 | 1.29 |
| N4BP2L1 | ENSG00000139597 | 54.11 | 0.19 | 0.0111 | 0.0433 | 1.42 |
| N6AMT1 | ENSG00000156239 | 37.13 | 0.18 | 0.0111 | 0.0433 | 1.38 |
| NAA20 | ENSG00000173418 | 45.41 | 0.21 | 0.0053 | 0.0361 | 2.72 |
| NAA30 | ENSG00000139977 | 55.83 | 0.20 | 0.0076 | 0.0382 | 1.76 |
| NAA35 | ENSG00000135040 | 80.36 | 0.21 | 0.0037 | 0.0342 | 1.45 |
| NAA40 | ENSG00000110583 | 109.20 | 0.23 | 0.0017 | 0.0342 | 1.18 |
| NAAA | ENSG00000138744 | 81.99 | 0.22 | 0.0035 | 0.0342 | 0.67 |
| NACA | ENSG00000196531 | 354.96 | 0.19 | 0.0113 | 0.0435 | 2.53 |
| NACC2 | ENSG00000148411 | 114.32 | 0.20 | 0.0068 | 0.0374 | 1.32 |
| NADK | ENSG00000008130 | 186.05 | 0.22 | 0.0031 | 0.0342 | 1.15 |
| NADSYN1 | ENSG00000172890 | 402.63 | 0.20 | 0.0050 | 0.0356 | 1.12 |
| NAE1 | ENSG00000159593 | 37.32 | 0.21 | 0.0063 | 0.0370 | 2.88 |
| NAGA | ENSG00000198951 | 82.66 | 0.21 | 0.0038 | 0.0342 | 1.22 |
| NAGLU | ENSG00000108784 | 47.68 | 0.22 | 0.0034 | 0.0342 | 1.46 |
| NAGS | ENSG00000161653 | 9.00 | 0.20 | 0.0076 | 0.0382 | 1.32 |
| NANS | ENSG00000095380 | 225.53 | 0.24 | 0.0013 | 0.0342 | 0.71 |
| NAP1L4 | ENSG00000205531 | 187.89 | 0.18 | 0.0124 | 0.0450 | 1.82 |
| NAPEPLD | ENSG00000161048 | 169.58 | 0.25 | 0.0005 | 0.0342 | 1.18 |
| NAPG | ENSG00000134265 | 79.11 | 0.20 | 0.0062 | 0.0368 | 1.42 |
| NAPRT1 | ENSG00000147813 | 97.08 | 0.23 | 0.0038 | 0.0342 | 1.66 |
| NARF | ENSG00000141562 | 40.74 | 0.19 | 0.0105 | 0.0426 | 1.85 |
| NARFL | ENSG00000103245 | 43.33 | 0.23 | 0.0030 | 0.0342 | 1.59 |
| NARG2 | ENSG00000128915 | 76.24 | 0.20 | 0.0069 | 0.0375 | 1.68 |
| NARS | ENSG00000134440 | 194.23 | 0.18 | 0.0132 | 0.0460 | 1.39 |
| NARS2 | ENSG00000137513 | 28.30 | 0.19 | 0.0115 | 0.0438 | 2.42 |
| NBAS | ENSG00000151779 | 229.78 | 0.17 | 0.0158 | 0.0497 | 1.53 |
| NBEAL1 | ENSG00000144426 | 629.44 | 0.22 | 0.0031 | 0.0342 | 0.82 |
| NBL1 | ENSG00000158747 | 434.05 | 0.21 | 0.0060 | 0.0366 | 1.18 |
| NBPF1 | ENSG00000219481 | 244.75 | 0.24 | 0.0010 | 0.0342 | 0.81 |
| NBPF15 | ENSG00000243452 | 70.40 | 0.19 | 0.0099 | 0.0417 | 1.48 |
| NBPF8 | ENSG00000225241 | 66.07 | 0.19 | 0.0115 | 0.0438 | 1.59 |
| NBPF9 | ENSG00000168614 | 70.65 | 0.18 | 0.0127 | 0.0454 | 1.31 |
| NBR1 | ENSG00000188554 | 344.91 | 0.25 | 0.0007 | 0.0342 | 1.48 |
| NCAPD2 | ENSG00000010292 | 131.07 | 0.23 | 0.0015 | 0.0342 | 2.72 |
| NCBP2 | ENSG00000114503 | 120.03 | 0.18 | 0.0132 | 0.0460 | 2.19 |
| NCEH1 | ENSG00000144959 | 58.05 | 0.21 | 0.0069 | 0.0375 | 2.17 |
| NCKAP1 | ENSG00000061676 | 232.07 | 0.22 | 0.0027 | 0.0342 | 2.37 |
| NCKIPSD | ENSG00000213672 | 53.19 | 0.24 | 0.0016 | 0.0342 | 1.62 |
| NCL | ENSG00000115053 | 407.25 | 0.18 | 0.0132 | 0.0460 | 2.92 |
| NCLN | ENSG00000125912 | 170.75 | 0.22 | 0.0032 | 0.0342 | 1.90 |
| NCOA1 | ENSG00000084676 | 290.67 | 0.19 | 0.0076 | 0.0382 | 0.90 |
| NCOA2 | ENSG00000140396 | 296.86 | 0.19 | 0.0084 | 0.0397 | 1.30 |
| NCOA4 | ENSG00000138293 | 301.97 | 0.21 | 0.0042 | 0.0350 | 1.29 |
| NCOA5 | ENSG00000124160 | 75.80 | 0.19 | 0.0109 | 0.0431 | 1.81 |
| NCOA6 | ENSG00000198646 | 302.10 | 0.20 | 0.0046 | 0.0352 | 2.02 |
| NCOR1 | ENSG00000141027 | 926.47 | 0.21 | 0.0035 | 0.0342 | 1.02 |
| NCOR2 | ENSG00000196498 | 583.75 | 0.19 | 0.0121 | 0.0445 | 1.87 |
| NCSTN | ENSG00000162736 | 126.10 | 0.20 | 0.0051 | 0.0358 | 1.45 |
| NDFIP1 | ENSG00000131507 | 139.23 | 0.18 | 0.0116 | 0.0439 | 1.53 |
| NDFIP2 | ENSG00000102471 | 94.13 | 0.23 | 0.0021 | 0.0342 | 2.13 |
| NDOR1 | ENSG00000188566 | 89.95 | 0.23 | 0.0032 | 0.0342 | 1.52 |
| NDST1 | ENSG00000070614 | 225.61 | 0.19 | 0.0133 | 0.0462 | 1.33 |
| NDUFA1 | ENSG00000125356 | 54.20 | 0.18 | 0.0113 | 0.0435 | 1.54 |
| NDUFA12 | ENSG00000184752 | 23.09 | 0.25 | 0.0009 | 0.0342 | 1.81 |
| NDUFA13 | ENSG00000186010 | 76.66 | 0.20 | 0.0091 | 0.0407 | 1.24 |
| NDUFA3 | ENSG00000170906 | 39.96 | 0.23 | 0.0036 | 0.0342 | 1.46 |
| NDUFA4 | ENSG00000189043 | 41.80 | 0.20 | 0.0060 | 0.0366 | 2.12 |
| NDUFA6 | ENSG00000184983 | 54.87 | 0.20 | 0.0075 | 0.0382 | 1.10 |
| NDUFA7 | ENSG00000167774 | 14.44 | 0.18 | 0.0139 | 0.0469 | 1.86 |
| NDUFA8 | ENSG00000119421 | 17.14 | 0.19 | 0.0125 | 0.0451 | 2.02 |
| NDUFA9 | ENSG00000139180 | 102.57 | 0.20 | 0.0067 | 0.0373 | 1.65 |
| NDUFAF1 | ENSG00000137806 | 10.40 | 0.21 | 0.0068 | 0.0374 | 1.42 |
| NDUFAF3 | ENSG00000178057 | 34.31 | 0.19 | 0.0147 | 0.0482 | 1.60 |
| NDUFB11 | ENSG00000147123 | 32.00 | 0.22 | 0.0022 | 0.0342 | 2.01 |
| NDUFB3 | ENSG00000119013 | 24.66 | 0.22 | 0.0034 | 0.0342 | 1.54 |
| NDUFB5 | ENSG00000136521 | 68.02 | 0.20 | 0.0058 | 0.0364 | 1.49 |
| NDUFB7 | ENSG00000099795 | 31.53 | 0.18 | 0.0147 | 0.0482 | 1.47 |
| NDUFC2 | ENSG00000151366 | 35.36 | 0.20 | 0.0069 | 0.0375 | 1.82 |
| NDUFS1 | ENSG00000023228 | 226.85 | 0.22 | 0.0024 | 0.0342 | 1.45 |
| NDUFS2 | ENSG00000158864 | 105.31 | 0.19 | 0.0066 | 0.0373 | 1.36 |
| NDUFS3 | ENSG00000213619 | 57.47 | 0.19 | 0.0061 | 0.0368 | 1.45 |
| NDUFS5 | ENSG00000168653 | 32.13 | 0.19 | 0.0112 | 0.0434 | 2.13 |
| NDUFS7 | ENSG00000115286 | 60.30 | 0.18 | 0.0125 | 0.0451 | 1.16 |
| NDUFS8 | ENSG00000110717 | 60.05 | 0.20 | 0.0049 | 0.0355 | 1.28 |
| NDUFV2 | ENSG00000178127 | 77.35 | 0.18 | 0.0129 | 0.0457 | 1.18 |
| NDUFV3 | ENSG00000160194 | 58.34 | 0.19 | 0.0063 | 0.0370 | 1.36 |
| NEBL | ENSG00000078114 | 103.58 | 0.22 | 0.0068 | 0.0374 | 4.85 |
| NECAB3 | ENSG00000125967 | 57.02 | 0.22 | 0.0035 | 0.0342 | 1.87 |
| NECAP2 | ENSG00000157191 | 90.61 | 0.20 | 0.0073 | 0.0379 | 1.44 |
| NEDD4L | ENSG00000049759 | 520.66 | 0.20 | 0.0082 | 0.0393 | 0.46 |
| NEDD8 | ENSG00000129559 | 54.79 | 0.21 | 0.0037 | 0.0342 | 1.80 |
| NEDD8-MDP1 | ENSG00000255526 | 18.96 | 0.21 | 0.0058 | 0.0364 | 1.90 |
| NEIL1 | ENSG00000140398 | 51.13 | 0.19 | 0.0126 | 0.0452 | 0.88 |
| NEK3 | ENSG00000136098 | 81.21 | 0.25 | 0.0005 | 0.0342 | 2.05 |
| NEK4 | ENSG00000114904 | 63.10 | 0.20 | 0.0060 | 0.0366 | 1.83 |
| NEK6 | ENSG00000119408 | 188.50 | 0.26 | 0.0009 | 0.0342 | 1.55 |
| NEK7 | ENSG00000151414 | 93.09 | 0.19 | 0.0093 | 0.0409 | 1.66 |
| NEK8 | ENSG00000160602 | 19.64 | 0.22 | 0.0041 | 0.0349 | 1.64 |
| NEK9 | ENSG00000119638 | 196.37 | 0.19 | 0.0084 | 0.0397 | 1.36 |
| NELFE | ENSG00000204356 | 40.30 | 0.19 | 0.0108 | 0.0429 | 1.87 |
| NELL2 | ENSG00000184613 | 16.37 | 0.19 | 0.0139 | 0.0469 | 0.78 |
| NEMF | ENSG00000165525 | 156.61 | 0.19 | 0.0078 | 0.0386 | 1.36 |
| NEO1 | ENSG00000067141 | 592.47 | 0.23 | 0.0015 | 0.0342 | 0.67 |
| NET1 | ENSG00000173848 | 373.62 | 0.19 | 0.0085 | 0.0399 | 1.36 |
| NEU1 | ENSG00000204386 | 62.08 | 0.21 | 0.0034 | 0.0342 | 2.30 |
| NEU4 | ENSG00000204099 | 129.35 | 0.19 | 0.0143 | 0.0476 | 0.42 |
| NEURL | ENSG00000107954 | 114.13 | 0.19 | 0.0115 | 0.0438 | 0.51 |
| NEURL1B | ENSG00000214357 | 203.12 | 0.20 | 0.0107 | 0.0428 | 1.24 |
| NEURL4 | ENSG00000215041 | 95.68 | 0.20 | 0.0086 | 0.0399 | 1.10 |
| NF1 | ENSG00000196712 | 494.47 | 0.21 | 0.0046 | 0.0352 | 1.84 |
| NF2 | ENSG00000186575 | 137.51 | 0.18 | 0.0115 | 0.0438 | 1.54 |
| NFATC3 | ENSG00000072736 | 278.75 | 0.19 | 0.0131 | 0.0460 | 1.42 |
| NFE2L1 | ENSG00000082641 | 419.24 | 0.25 | 0.0008 | 0.0342 | 1.11 |
| NFIC | ENSG00000141905 | 108.60 | 0.18 | 0.0143 | 0.0476 | 1.07 |
| NFIX | ENSG00000008441 | 233.74 | 0.20 | 0.0110 | 0.0432 | 1.81 |
| NFKBIL1 | ENSG00000204498 | 20.92 | 0.22 | 0.0049 | 0.0355 | 1.39 |
| NFKBIZ | ENSG00000144802 | 310.18 | 0.22 | 0.0040 | 0.0346 | 0.97 |
| NFRKB | ENSG00000170322 | 108.38 | 0.24 | 0.0011 | 0.0342 | 1.50 |
| NFS1 | ENSG00000244005 | 68.27 | 0.20 | 0.0071 | 0.0376 | 2.43 |
| NFXL1 | ENSG00000170448 | 46.34 | 0.22 | 0.0054 | 0.0362 | 2.17 |
| NFYA | ENSG00000001167 | 93.82 | 0.20 | 0.0078 | 0.0386 | 1.69 |
| NFYC | ENSG00000066136 | 107.72 | 0.18 | 0.0146 | 0.0481 | 1.08 |
| NGEF | ENSG00000066248 | 91.56 | 0.21 | 0.0044 | 0.0351 | 1.01 |
| NGRN | ENSG00000182768 | 93.28 | 0.21 | 0.0035 | 0.0342 | 1.94 |
| NHEJ1 | ENSG00000187736 | 56.58 | 0.27 | 0.0002 | 0.0342 | 1.12 |
| NHLRC2 | ENSG00000196865 | 203.33 | 0.18 | 0.0138 | 0.0468 | 1.49 |
| NHLRC3 | ENSG00000188811 | 129.63 | 0.22 | 0.0033 | 0.0342 | 1.94 |
| NHP2 | ENSG00000145912 | 32.27 | 0.22 | 0.0029 | 0.0342 | 2.32 |
| NHSL1 | ENSG00000135540 | 458.21 | 0.24 | 0.0008 | 0.0342 | 0.75 |
| NICN1 | ENSG00000145029 | 50.47 | 0.19 | 0.0135 | 0.0464 | 1.44 |
| NIPA1 | ENSG00000170113 | 75.03 | 0.25 | 0.0009 | 0.0342 | 1.58 |
| NIPAL1 | ENSG00000163293 | 109.37 | 0.25 | 0.0007 | 0.0342 | 0.91 |
| NIPAL2 | ENSG00000104361 | 223.77 | 0.23 | 0.0013 | 0.0342 | 1.03 |
| NIPAL3 | ENSG00000001461 | 152.01 | 0.23 | 0.0010 | 0.0342 | 1.18 |
| NIPSNAP1 | ENSG00000184117 | 60.56 | 0.24 | 0.0012 | 0.0342 | 2.22 |
| NIT1 | ENSG00000158793 | 90.58 | 0.22 | 0.0022 | 0.0342 | 0.95 |
| NKIRAS2 | ENSG00000168256 | 60.69 | 0.19 | 0.0102 | 0.0422 | 1.78 |
| NME1-NME2 | ENSG00000243678 | 136.87 | 0.20 | 0.0050 | 0.0356 | 3.01 |
| NME2 | ENSG00000011052 | 138.76 | 0.20 | 0.0071 | 0.0376 | 3.00 |
| NME4 | ENSG00000103202 | 43.99 | 0.19 | 0.0148 | 0.0484 | 2.22 |
| NME6 | ENSG00000172113 | 37.20 | 0.21 | 0.0060 | 0.0366 | 1.42 |
| NMNAT1 | ENSG00000173614 | 43.34 | 0.20 | 0.0076 | 0.0382 | 0.95 |
| NMRAL1 | ENSG00000153406 | 26.55 | 0.20 | 0.0067 | 0.0373 | 2.04 |
| NMT1 | ENSG00000136448 | 149.26 | 0.20 | 0.0077 | 0.0384 | 1.54 |
| NMT2 | ENSG00000152465 | 56.50 | 0.21 | 0.0037 | 0.0342 | 1.18 |
| NNAT | ENSG00000053438 | 32.52 | 0.19 | 0.0128 | 0.0456 | 1.63 |
| NNT | ENSG00000112992 | 137.89 | 0.19 | 0.0097 | 0.0415 | 1.52 |
| NOB1 | ENSG00000141101 | 35.32 | 0.24 | 0.0017 | 0.0342 | 3.22 |
| NOD1 | ENSG00000106100 | 85.33 | 0.21 | 0.0048 | 0.0354 | 1.78 |
| NOL10 | ENSG00000115761 | 59.59 | 0.20 | 0.0069 | 0.0375 | 2.13 |
| NOL11 | ENSG00000130935 | 40.02 | 0.22 | 0.0034 | 0.0342 | 2.44 |
| NOL3 | ENSG00000140939 | 53.22 | 0.23 | 0.0032 | 0.0342 | 1.75 |
| NOL8 | ENSG00000198000 | 98.30 | 0.19 | 0.0139 | 0.0469 | 2.23 |
| NOP10 | ENSG00000182117 | 34.21 | 0.18 | 0.0130 | 0.0458 | 1.77 |
| NOS1AP | ENSG00000198929 | 72.37 | 0.22 | 0.0028 | 0.0342 | 1.10 |
| NOS2 | ENSG00000007171 | 51.76 | 0.24 | 0.0068 | 0.0374 | 1.82 |
| NOTCH1 | ENSG00000148400 | 378.93 | 0.20 | 0.0075 | 0.0382 | 2.17 |
| NOTCH2 | ENSG00000134250 | 485.97 | 0.18 | 0.0132 | 0.0460 | 1.43 |
| NOVA2 | ENSG00000104967 | 3.49 | 0.21 | 0.0107 | 0.0428 | 1.48 |
| NOX1 | ENSG00000007952 | 109.22 | 0.25 | 0.0007 | 0.0342 | 2.32 |
| NOXA1 | ENSG00000188747 | 81.67 | 0.27 | 0.0012 | 0.0342 | 1.27 |
| NOXO1 | ENSG00000196408 | 22.08 | 0.19 | 0.0083 | 0.0395 | 2.03 |
| NPAS2 | ENSG00000170485 | 148.45 | 0.23 | 0.0014 | 0.0342 | 2.17 |
| NPC2 | ENSG00000119655 | 69.76 | 0.22 | 0.0029 | 0.0342 | 1.52 |
| NPDC1 | ENSG00000107281 | 227.37 | 0.24 | 0.0015 | 0.0342 | 1.39 |
| NPEPPS | ENSG00000141279 | 303.89 | 0.20 | 0.0052 | 0.0359 | 1.49 |
| NPLOC4 | ENSG00000182446 | 312.97 | 0.19 | 0.0132 | 0.0460 | 1.59 |
| NPRL2 | ENSG00000114388 | 20.78 | 0.18 | 0.0129 | 0.0457 | 1.61 |
| NPRL3 | ENSG00000103148 | 86.69 | 0.25 | 0.0012 | 0.0342 | 2.18 |
| NPTN | ENSG00000156642 | 122.67 | 0.19 | 0.0120 | 0.0444 | 1.22 |
| NQO1 | ENSG00000181019 | 56.02 | 0.21 | 0.0063 | 0.0370 | 3.80 |
| NR1H3 | ENSG00000025434 | 43.58 | 0.22 | 0.0028 | 0.0342 | 1.61 |
| NR1I2 | ENSG00000144852 | 136.23 | 0.20 | 0.0081 | 0.0391 | 1.48 |
| NR2C2AP | ENSG00000184162 | 17.26 | 0.19 | 0.0127 | 0.0454 | 2.47 |
| NR2F2 | ENSG00000185551 | 112.43 | 0.22 | 0.0055 | 0.0363 | 1.39 |
| NR2F6 | ENSG00000160113 | 119.86 | 0.22 | 0.0046 | 0.0352 | 1.20 |
| NR3C2 | ENSG00000151623 | 512.82 | 0.21 | 0.0033 | 0.0342 | 0.26 |
| NR5A2 | ENSG00000116833 | 167.13 | 0.20 | 0.0051 | 0.0358 | 0.31 |
| NRBP1 | ENSG00000115216 | 128.45 | 0.18 | 0.0107 | 0.0428 | 1.48 |
| NRBP2 | ENSG00000185189 | 131.52 | 0.22 | 0.0038 | 0.0342 | 1.37 |
| NRD1 | ENSG00000078618 | 241.47 | 0.19 | 0.0061 | 0.0368 | 1.55 |
| NRGN | ENSG00000154146 | 62.76 | 0.20 | 0.0067 | 0.0373 | 0.25 |
| NRIP1 | ENSG00000180530 | 323.64 | 0.22 | 0.0020 | 0.0342 | 1.12 |
| NRIP2 | ENSG00000053702 | 69.90 | 0.18 | 0.0141 | 0.0472 | 1.14 |
| NRTN | ENSG00000171119 | 38.31 | 0.20 | 0.0060 | 0.0366 | 0.89 |
| NSD1 | ENSG00000165671 | 467.98 | 0.22 | 0.0028 | 0.0342 | 1.55 |
| NSDHL | ENSG00000147383 | 14.87 | 0.21 | 0.0045 | 0.0352 | 3.01 |
| NSF | ENSG00000073969 | 100.34 | 0.20 | 0.0042 | 0.0350 | 1.45 |
| NSMCE1 | ENSG00000169189 | 14.62 | 0.21 | 0.0054 | 0.0362 | 2.83 |
| NSMCE4A | ENSG00000107672 | 58.94 | 0.23 | 0.0010 | 0.0342 | 1.57 |
| NSUN5 | ENSG00000130305 | 33.35 | 0.20 | 0.0072 | 0.0377 | 1.99 |
| NSUN6 | ENSG00000241058 | 35.51 | 0.21 | 0.0058 | 0.0364 | 1.29 |
| NT5C1B-RDH14 | ENSG00000250741 | 11.56 | 0.21 | 0.0090 | 0.0405 | 1.13 |
| NT5C3L | ENSG00000141698 | 24.07 | 0.21 | 0.0070 | 0.0376 | 2.48 |
| NT5DC1 | ENSG00000178425 | 51.63 | 0.25 | 0.0007 | 0.0342 | 1.67 |
| NT5DC2 | ENSG00000168268 | 57.46 | 0.20 | 0.0103 | 0.0423 | 1.82 |
| NT5DC3 | ENSG00000111696 | 147.95 | 0.22 | 0.0046 | 0.0352 | 1.51 |
| NT5E | ENSG00000135318 | 97.49 | 0.20 | 0.0073 | 0.0379 | 1.64 |
| NTAN1 | ENSG00000157045 | 32.19 | 0.23 | 0.0027 | 0.0342 | 1.40 |
| NTN4 | ENSG00000074527 | 64.11 | 0.22 | 0.0025 | 0.0342 | 0.72 |
| NTPCR | ENSG00000135778 | 75.74 | 0.23 | 0.0018 | 0.0342 | 1.34 |
| NUAK1 | ENSG00000074590 | 31.13 | 0.19 | 0.0135 | 0.0464 | 2.45 |
| NUAK2 | ENSG00000163545 | 58.41 | 0.26 | 0.0008 | 0.0342 | 1.66 |
| NUB1 | ENSG00000013374 | 118.57 | 0.23 | 0.0021 | 0.0342 | 1.89 |
| NUDC | ENSG00000090273 | 55.05 | 0.20 | 0.0065 | 0.0371 | 2.04 |
| NUDCD2 | ENSG00000170584 | 15.92 | 0.18 | 0.0133 | 0.0462 | 2.30 |
| NUDCD3 | ENSG00000015676 | 154.46 | 0.19 | 0.0117 | 0.0439 | 2.09 |
| NUDT14 | ENSG00000183828 | 41.87 | 0.20 | 0.0084 | 0.0397 | 1.99 |
| NUDT15 | ENSG00000136159 | 36.11 | 0.22 | 0.0049 | 0.0355 | 2.46 |
| NUDT16 | ENSG00000198585 | 190.57 | 0.21 | 0.0047 | 0.0353 | 0.98 |
| NUDT16L1 | ENSG00000168101 | 29.92 | 0.25 | 0.0014 | 0.0342 | 1.35 |
| NUDT18 | ENSG00000173566 | 18.31 | 0.25 | <.0001 | 0.0342 | 0.98 |
| NUDT19 | ENSG00000213965 | 36.87 | 0.25 | 0.0012 | 0.0342 | 2.31 |
| NUDT2 | ENSG00000164978 | 15.71 | 0.24 | 0.0013 | 0.0342 | 1.59 |
| NUDT21 | ENSG00000167005 | 143.46 | 0.20 | 0.0081 | 0.0391 | 2.56 |
| NUDT22 | ENSG00000149761 | 77.00 | 0.22 | 0.0063 | 0.0370 | 0.91 |
| NUDT3 | ENSG00000112664 | 40.29 | 0.20 | 0.0064 | 0.0370 | 1.98 |
| NUDT4 | ENSG00000173598 | 160.15 | 0.21 | 0.0033 | 0.0342 | 2.40 |
| NUDT5 | ENSG00000165609 | 86.92 | 0.20 | 0.0072 | 0.0377 | 1.76 |
| NUMA1 | ENSG00000137497 | 1120.61 | 0.21 | 0.0041 | 0.0349 | 1.23 |
| NUMB | ENSG00000133961 | 229.25 | 0.20 | 0.0057 | 0.0363 | 1.04 |
| NUP133 | ENSG00000069248 | 69.05 | 0.20 | 0.0069 | 0.0375 | 2.26 |
| NUP214 | ENSG00000126883 | 307.08 | 0.19 | 0.0126 | 0.0452 | 1.45 |
| NUP35 | ENSG00000163002 | 22.63 | 0.24 | 0.0014 | 0.0342 | 2.03 |
| NUP62 | ENSG00000213024 | 112.93 | 0.21 | 0.0046 | 0.0352 | 1.97 |
| NUP85 | ENSG00000125450 | 67.98 | 0.22 | 0.0029 | 0.0342 | 1.90 |
| NUPL2 | ENSG00000136243 | 39.74 | 0.23 | 0.0032 | 0.0342 | 1.82 |
| NUPR1 | ENSG00000176046 | 59.47 | 0.21 | 0.0043 | 0.0351 | 0.97 |
| NWD1 | ENSG00000188039 | 20.11 | 0.23 | 0.0031 | 0.0342 | 0.81 |
| OAF | ENSG00000184232 | 180.11 | 0.18 | 0.0150 | 0.0486 | 0.86 |
| OAS1 | ENSG00000089127 | 78.59 | 0.22 | 0.0068 | 0.0374 | 1.90 |
| OCEL1 | ENSG00000099330 | 23.14 | 0.20 | 0.0070 | 0.0376 | 0.69 |
| OCIAD1 | ENSG00000109180 | 109.23 | 0.20 | 0.0056 | 0.0363 | 1.53 |
| OCIAD2 | ENSG00000145247 | 34.99 | 0.24 | 0.0017 | 0.0342 | 2.61 |
| OCLN | ENSG00000197822 | 84.02 | 0.21 | 0.0042 | 0.0350 | 1.54 |
| OCRL | ENSG00000122126 | 144.86 | 0.22 | 0.0016 | 0.0342 | 1.58 |
| OFD1 | ENSG00000046651 | 123.86 | 0.23 | 0.0024 | 0.0342 | 1.55 |
| OGFOD1 | ENSG00000087263 | 39.85 | 0.20 | 0.0070 | 0.0376 | 2.21 |
| OGFOD2 | ENSG00000111325 | 59.77 | 0.23 | 0.0024 | 0.0342 | 1.28 |
| OGT | ENSG00000147162 | 1270.77 | 0.18 | 0.0128 | 0.0456 | 1.58 |
| OLA1 | ENSG00000138430 | 71.77 | 0.20 | 0.0075 | 0.0382 | 3.22 |
| OMP | ENSG00000254550 | 12.07 | 0.28 | 0.0009 | 0.0342 | 0.77 |
| OPA1 | ENSG00000198836 | 222.78 | 0.19 | 0.0095 | 0.0413 | 1.73 |
| OPHN1 | ENSG00000079482 | 77.49 | 0.21 | 0.0048 | 0.0354 | 1.30 |
| OPTN | ENSG00000123240 | 183.37 | 0.26 | 0.0002 | 0.0342 | 1.38 |
| ORAI3 | ENSG00000175938 | 29.44 | 0.28 | 0.0005 | 0.0342 | 1.73 |
| ORAOV1 | ENSG00000149716 | 105.63 | 0.24 | 0.0014 | 0.0342 | 1.82 |
| ORC5 | ENSG00000164815 | 22.90 | 0.18 | 0.0157 | 0.0496 | 2.39 |
| ORMDL2 | ENSG00000123353 | 28.13 | 0.25 | 0.0007 | 0.0342 | 1.46 |
| ORMDL3 | ENSG00000172057 | 92.89 | 0.20 | 0.0062 | 0.0368 | 1.67 |
| OS9 | ENSG00000135506 | 317.51 | 0.21 | 0.0049 | 0.0355 | 1.18 |
| OSBP | ENSG00000110048 | 194.47 | 0.21 | 0.0049 | 0.0355 | 1.52 |
| OSBP2 | ENSG00000184792 | 34.82 | 0.21 | 0.0056 | 0.0363 | 1.83 |
| OSBPL2 | ENSG00000130703 | 157.87 | 0.23 | 0.0012 | 0.0342 | 1.99 |
| OSBPL7 | ENSG00000006025 | 139.33 | 0.22 | 0.0031 | 0.0342 | 0.94 |
| OSBPL8 | ENSG00000091039 | 124.42 | 0.19 | 0.0113 | 0.0435 | 2.38 |
| OSGIN1 | ENSG00000140961 | 12.40 | 0.19 | 0.0154 | 0.0491 | 0.87 |
| OST4 | ENSG00000228474 | 64.29 | 0.22 | 0.0020 | 0.0342 | 1.85 |
| OSTC | ENSG00000198856 | 33.40 | 0.20 | 0.0071 | 0.0376 | 2.15 |
| OTUB1 | ENSG00000167770 | 73.61 | 0.21 | 0.0045 | 0.0352 | 1.67 |
| OTUD4 | ENSG00000164164 | 180.69 | 0.23 | 0.0020 | 0.0342 | 1.77 |
| OTUD7B | ENSG00000163113 | 164.59 | 0.23 | 0.0012 | 0.0342 | 1.24 |
| OVOL1 | ENSG00000172818 | 64.32 | 0.21 | 0.0047 | 0.0353 | 1.47 |
| OXA1L | ENSG00000155463 | 115.14 | 0.18 | 0.0129 | 0.0457 | 1.36 |
| OXR1 | ENSG00000164830 | 145.11 | 0.20 | 0.0045 | 0.0352 | 1.54 |
| OXSM | ENSG00000151093 | 17.92 | 0.20 | 0.0077 | 0.0384 | 1.28 |
| P2RX4 | ENSG00000135124 | 156.95 | 0.21 | 0.0061 | 0.0368 | 0.78 |
| P2RY4 | ENSG00000186912 | 6.80 | 0.22 | 0.0072 | 0.0377 | 0.54 |
| P4HA1 | ENSG00000122884 | 54.07 | 0.23 | 0.0022 | 0.0342 | 2.82 |
| P4HA2 | ENSG00000072682 | 58.54 | 0.25 | 0.0007 | 0.0342 | 1.60 |
| P4HB | ENSG00000185624 | 613.22 | 0.20 | 0.0060 | 0.0366 | 1.64 |
| PABPC1 | ENSG00000070756 | 1422.28 | 0.22 | 0.0026 | 0.0342 | 2.94 |
| PABPN1 | ENSG00000100836 | 283.46 | 0.21 | 0.0045 | 0.0352 | 1.29 |
| PACS2 | ENSG00000179364 | 143.66 | 0.21 | 0.0056 | 0.0363 | 1.57 |
| PACSIN2 | ENSG00000100266 | 258.81 | 0.18 | 0.0124 | 0.0450 | 0.81 |
| PADI2 | ENSG00000117115 | 897.34 | 0.22 | 0.0027 | 0.0342 | 0.18 |
| PAFAH2 | ENSG00000158006 | 77.58 | 0.26 | 0.0006 | 0.0342 | 0.93 |
| PAG1 | ENSG00000076641 | 198.47 | 0.19 | 0.0094 | 0.0411 | 0.73 |
| PAIP1 | ENSG00000172239 | 40.31 | 0.18 | 0.0138 | 0.0468 | 2.69 |
| PAIP2 | ENSG00000120727 | 55.25 | 0.20 | 0.0076 | 0.0382 | 1.81 |
| PAK1 | ENSG00000149269 | 182.07 | 0.21 | 0.0031 | 0.0342 | 1.58 |
| PAK2 | ENSG00000180370 | 227.25 | 0.18 | 0.0125 | 0.0451 | 1.98 |
| PAK4 | ENSG00000130669 | 119.58 | 0.22 | 0.0031 | 0.0342 | 1.31 |
| PALLD | ENSG00000129116 | 320.01 | 0.21 | 0.0039 | 0.0345 | 1.46 |
| PAM | ENSG00000145730 | 157.30 | 0.18 | 0.0137 | 0.0467 | 2.10 |
| PAN2 | ENSG00000135473 | 147.97 | 0.20 | 0.0056 | 0.0363 | 1.44 |
| PANK1 | ENSG00000152782 | 42.22 | 0.19 | 0.0097 | 0.0415 | 1.88 |
| PANK3 | ENSG00000120137 | 650.95 | 0.19 | 0.0103 | 0.0423 | 1.08 |
| PANK4 | ENSG00000157881 | 43.86 | 0.23 | 0.0024 | 0.0342 | 1.09 |
| PAPOLA | ENSG00000090060 | 374.99 | 0.21 | 0.0055 | 0.0363 | 1.79 |
| PAPSS1 | ENSG00000138801 | 36.50 | 0.21 | 0.0039 | 0.0345 | 1.99 |
| PAPSS2 | ENSG00000198682 | 276.63 | 0.21 | 0.0052 | 0.0359 | 0.59 |
| PAQR8 | ENSG00000170915 | 186.89 | 0.22 | 0.0035 | 0.0342 | 1.00 |
| PARD3 | ENSG00000148498 | 168.67 | 0.22 | 0.0023 | 0.0342 | 1.44 |
| PARD3B | ENSG00000116117 | 150.39 | 0.21 | 0.0030 | 0.0342 | 1.36 |
| PARD6B | ENSG00000124171 | 54.99 | 0.19 | 0.0150 | 0.0486 | 2.68 |
| PARG | ENSG00000227345 | 25.44 | 0.21 | 0.0050 | 0.0356 | 1.95 |
| PARM1 | ENSG00000169116 | 797.07 | 0.27 | 0.0003 | 0.0342 | 0.51 |
| PARN | ENSG00000140694 | 84.77 | 0.23 | 0.0011 | 0.0342 | 1.79 |
| PARP10 | ENSG00000178685 | 122.94 | 0.23 | 0.0043 | 0.0351 | 1.78 |
| PARP12 | ENSG00000059378 | 142.65 | 0.24 | 0.0033 | 0.0342 | 1.37 |
| PARP16 | ENSG00000138617 | 22.04 | 0.19 | 0.0122 | 0.0446 | 1.49 |
| PARP2 | ENSG00000129484 | 35.59 | 0.19 | 0.0097 | 0.0415 | 1.36 |
| PARP3 | ENSG00000041880 | 36.58 | 0.21 | 0.0054 | 0.0362 | 1.34 |
| PARP4 | ENSG00000102699 | 402.65 | 0.25 | 0.0009 | 0.0342 | 2.27 |
| PARP6 | ENSG00000137817 | 88.35 | 0.22 | 0.0037 | 0.0342 | 1.44 |
| PARP8 | ENSG00000151883 | 148.82 | 0.19 | 0.0091 | 0.0407 | 1.08 |
| PARS2 | ENSG00000162396 | 14.78 | 0.21 | 0.0074 | 0.0380 | 1.67 |
| PATL1 | ENSG00000166889 | 124.15 | 0.19 | 0.0084 | 0.0397 | 2.12 |
| PAWR | ENSG00000177425 | 84.04 | 0.26 | 0.0004 | 0.0342 | 1.57 |
| PAXIP1 | ENSG00000157212 | 75.13 | 0.23 | 0.0018 | 0.0342 | 1.90 |
| PBRM1 | ENSG00000163939 | 250.46 | 0.18 | 0.0111 | 0.0433 | 1.51 |
| PC | ENSG00000173599 | 59.54 | 0.20 | 0.0065 | 0.0371 | 1.16 |
| PCBD1 | ENSG00000166228 | 47.42 | 0.24 | 0.0014 | 0.0342 | 2.52 |
| PCBP2 | ENSG00000197111 | 683.78 | 0.21 | 0.0035 | 0.0342 | 1.73 |
| PCBP4 | ENSG00000090097 | 46.34 | 0.22 | 0.0040 | 0.0346 | 1.77 |
| PCCA | ENSG00000175198 | 121.00 | 0.26 | 0.0006 | 0.0342 | 1.64 |
| PCCB | ENSG00000114054 | 86.14 | 0.22 | 0.0026 | 0.0342 | 1.29 |
| PCDH1 | ENSG00000156453 | 338.00 | 0.24 | 0.0013 | 0.0342 | 0.93 |
| PCDH17 | ENSG00000118946 | 22.26 | 0.23 | 0.0038 | 0.0342 | 2.15 |
| PCDH18 | ENSG00000189184 | 65.14 | 0.19 | 0.0119 | 0.0442 | 1.45 |
| PCGF1 | ENSG00000115289 | 16.78 | 0.19 | 0.0132 | 0.0460 | 1.68 |
| PCGF2 | ENSG00000056661 | 91.69 | 0.20 | 0.0075 | 0.0382 | 1.39 |
| PCGF3 | ENSG00000185619 | 346.77 | 0.21 | 0.0044 | 0.0351 | 1.15 |
| PCGF5 | ENSG00000180628 | 157.02 | 0.19 | 0.0110 | 0.0432 | 1.45 |
| PCID2 | ENSG00000126226 | 87.02 | 0.20 | 0.0103 | 0.0423 | 2.50 |
| PCK1 | ENSG00000124253 | 628.30 | 0.18 | 0.0137 | 0.0467 | 0.34 |
| PCK2 | ENSG00000100889 | 99.28 | 0.19 | 0.0088 | 0.0402 | 1.20 |
| PCLO | ENSG00000186472 | 191.34 | 0.18 | 0.0134 | 0.0463 | 1.06 |
| PCM1 | ENSG00000078674 | 449.20 | 0.19 | 0.0103 | 0.0423 | 1.30 |
| PCMT1 | ENSG00000120265 | 39.49 | 0.20 | 0.0074 | 0.0380 | 2.26 |
| PCMTD2 | ENSG00000203880 | 85.91 | 0.21 | 0.0032 | 0.0342 | 3.37 |
| PCNP | ENSG00000081154 | 94.62 | 0.20 | 0.0074 | 0.0380 | 2.12 |
| PCNXL3 | ENSG00000197136 | 265.32 | 0.20 | 0.0076 | 0.0382 | 1.43 |
| PCSK6 | ENSG00000140479 | 141.86 | 0.23 | 0.0013 | 0.0342 | 0.81 |
| PCSK9 | ENSG00000169174 | 21.03 | 0.21 | 0.0118 | 0.0441 | 6.37 |
| PCTP | ENSG00000141179 | 30.95 | 0.27 | 0.0012 | 0.0342 | 1.61 |
| PCYOX1 | ENSG00000116005 | 125.95 | 0.22 | 0.0035 | 0.0342 | 1.51 |
| PCYT1A | ENSG00000161217 | 185.86 | 0.21 | 0.0027 | 0.0342 | 1.17 |
| PCYT1B | ENSG00000102230 | 3.54 | -0.21 | 0.0090 | 0.0405 | 1.09 |
| PCYT2 | ENSG00000185813 | 59.17 | 0.26 | 0.0007 | 0.0342 | 1.56 |
| PDCD4 | ENSG00000150593 | 673.43 | 0.26 | 0.0006 | 0.0342 | 0.49 |
| PDCD6 | ENSG00000249915 | 204.54 | 0.20 | 0.0071 | 0.0376 | 1.43 |
| PDCD6IP | ENSG00000170248 | 369.71 | 0.20 | 0.0059 | 0.0365 | 1.37 |
| PDCL | ENSG00000136940 | 35.57 | 0.22 | 0.0021 | 0.0342 | 1.65 |
| PDDC1 | ENSG00000177225 | 148.83 | 0.21 | 0.0071 | 0.0376 | 2.17 |
| PDE10A | ENSG00000112541 | 12.71 | 0.21 | 0.0121 | 0.0445 | 3.16 |
| PDE12 | ENSG00000174840 | 65.16 | 0.19 | 0.0110 | 0.0432 | 1.61 |
| PDE3A | ENSG00000172572 | 243.54 | 0.19 | 0.0091 | 0.0407 | 0.50 |
| PDE4A | ENSG00000065989 | 67.08 | 0.20 | 0.0065 | 0.0371 | 1.58 |
| PDE4C | ENSG00000105650 | 61.21 | 0.22 | 0.0064 | 0.0370 | 0.46 |
| PDE4DIP | ENSG00000178104 | 479.29 | 0.18 | 0.0092 | 0.0408 | 0.91 |
| PDE8A | ENSG00000073417 | 242.81 | 0.21 | 0.0053 | 0.0361 | 0.81 |
| PDGFRL | ENSG00000104213 | 34.03 | 0.19 | 0.0089 | 0.0403 | 0.68 |
| PDHX | ENSG00000110435 | 49.24 | 0.20 | 0.0072 | 0.0377 | 1.77 |
| PDIA5 | ENSG00000065485 | 66.68 | 0.19 | 0.0073 | 0.0379 | 1.33 |
| PDIA6 | ENSG00000143870 | 201.66 | 0.21 | 0.0046 | 0.0352 | 2.48 |
| PDIK1L | ENSG00000175087 | 38.59 | 0.25 | 0.0013 | 0.0342 | 1.39 |
| PDK1 | ENSG00000152256 | 92.75 | 0.20 | 0.0073 | 0.0379 | 0.96 |
| PDK2 | ENSG00000005882 | 71.10 | 0.21 | 0.0045 | 0.0352 | 1.16 |
| PDLIM1 | ENSG00000107438 | 283.37 | 0.21 | 0.0040 | 0.0346 | 1.08 |
| PDLIM5 | ENSG00000163110 | 336.30 | 0.21 | 0.0038 | 0.0342 | 1.35 |
| PDP2 | ENSG00000172840 | 29.72 | 0.27 | 0.0003 | 0.0342 | 2.06 |
| PDPK1 | ENSG00000140992 | 213.06 | 0.23 | 0.0017 | 0.0342 | 1.58 |
| PDPR | ENSG00000090857 | 228.11 | 0.21 | 0.0041 | 0.0349 | 1.41 |
| PDS5A | ENSG00000121892 | 361.83 | 0.20 | 0.0070 | 0.0376 | 1.80 |
| PDS5B | ENSG00000083642 | 147.68 | 0.23 | 0.0020 | 0.0342 | 2.17 |
| PDSS2 | ENSG00000164494 | 45.53 | 0.21 | 0.0051 | 0.0358 | 1.51 |
| PDXDC1 | ENSG00000179889 | 492.06 | 0.22 | 0.0030 | 0.0342 | 1.14 |
| PDXK | ENSG00000160209 | 517.56 | 0.23 | 0.0021 | 0.0342 | 1.89 |
| PDZD3 | ENSG00000172367 | 121.11 | 0.28 | 0.0005 | 0.0342 | 0.46 |
| PDZD8 | ENSG00000165650 | 297.67 | 0.24 | 0.0051 | 0.0358 | 2.34 |
| PDZK1IP1 | ENSG00000162366 | 29.16 | 0.31 | 0.0007 | 0.0342 | 2.50 |
| PEAK1 | ENSG00000173517 | 460.56 | 0.21 | 0.0043 | 0.0351 | 0.97 |
| PELO | ENSG00000152684 | 48.86 | 0.26 | 0.0010 | 0.0342 | 1.63 |
| PERP | ENSG00000112378 | 130.73 | 0.30 | <.0001 | 0.0342 | 5.23 |
| PEX10 | ENSG00000157911 | 59.41 | 0.25 | 0.0009 | 0.0342 | 1.47 |
| PEX11B | ENSG00000131779 | 29.23 | 0.25 | 0.0005 | 0.0342 | 1.42 |
| PEX13 | ENSG00000162928 | 47.41 | 0.22 | 0.0018 | 0.0342 | 1.75 |
| PEX16 | ENSG00000121680 | 34.74 | 0.24 | 0.0013 | 0.0342 | 1.44 |
| PEX19 | ENSG00000162735 | 184.39 | 0.19 | 0.0103 | 0.0423 | 1.45 |
| PEX2 | ENSG00000164751 | 62.11 | 0.23 | 0.0020 | 0.0342 | 1.61 |
| PEX26 | ENSG00000215193 | 274.25 | 0.21 | 0.0062 | 0.0368 | 0.84 |
| PEX5 | ENSG00000139197 | 57.99 | 0.20 | 0.0054 | 0.0362 | 2.22 |
| PEX6 | ENSG00000124587 | 54.63 | 0.27 | 0.0002 | 0.0342 | 1.78 |
| PFKFB2 | ENSG00000123836 | 369.74 | 0.20 | 0.0074 | 0.0380 | 1.05 |
| PFKFB4 | ENSG00000114268 | 36.39 | 0.25 | 0.0022 | 0.0342 | 2.21 |
| PFKL | ENSG00000141959 | 436.53 | 0.23 | 0.0016 | 0.0342 | 1.35 |
| PFKM | ENSG00000152556 | 56.79 | 0.20 | 0.0056 | 0.0363 | 2.42 |
| PGAM4 | ENSG00000226784 | 14.87 | 0.18 | 0.0113 | 0.0435 | 1.48 |
| PGAP2 | ENSG00000148985 | 66.56 | 0.22 | 0.0032 | 0.0342 | 1.57 |
| PGAP3 | ENSG00000161395 | 73.02 | 0.18 | 0.0152 | 0.0489 | 1.13 |
| PGD | ENSG00000142657 | 77.03 | 0.20 | 0.0071 | 0.0376 | 2.18 |
| PGGT1B | ENSG00000164219 | 51.53 | 0.21 | 0.0038 | 0.0342 | 1.21 |
| PGK1 | ENSG00000102144 | 235.30 | 0.22 | 0.0049 | 0.0355 | 2.76 |
| PGLS | ENSG00000130313 | 39.40 | 0.22 | 0.0043 | 0.0351 | 2.03 |
| PGM1 | ENSG00000079739 | 105.26 | 0.19 | 0.0068 | 0.0374 | 0.94 |
| PGM2 | ENSG00000169299 | 35.24 | 0.20 | 0.0088 | 0.0402 | 2.18 |
| PGM3 | ENSG00000013375 | 167.32 | 0.19 | 0.0122 | 0.0446 | 1.37 |
| PGPEP1 | ENSG00000130517 | 276.98 | 0.25 | 0.0006 | 0.0342 | 1.00 |
| PGRMC1 | ENSG00000101856 | 106.69 | 0.21 | 0.0048 | 0.0354 | 2.42 |
| PGRMC2 | ENSG00000164040 | 100.10 | 0.25 | 0.0009 | 0.0342 | 1.27 |
| PGS1 | ENSG00000087157 | 64.34 | 0.19 | 0.0096 | 0.0414 | 1.42 |
| PHACTR2 | ENSG00000112419 | 258.92 | 0.20 | 0.0085 | 0.0399 | 1.54 |
| PHACTR4 | ENSG00000204138 | 198.47 | 0.20 | 0.0049 | 0.0355 | 1.03 |
| PHB | ENSG00000167085 | 112.28 | 0.20 | 0.0073 | 0.0379 | 1.87 |
| PHB2 | ENSG00000215021 | 202.18 | 0.21 | 0.0049 | 0.0355 | 1.86 |
| PHF12 | ENSG00000109118 | 126.09 | 0.18 | 0.0119 | 0.0442 | 1.16 |
| PHF13 | ENSG00000116273 | 32.31 | 0.19 | 0.0121 | 0.0445 | 2.03 |
| PHF14 | ENSG00000106443 | 132.15 | 0.18 | 0.0137 | 0.0467 | 2.28 |
| PHF19 | ENSG00000119403 | 57.56 | 0.18 | 0.0159 | 0.0498 | 2.64 |
| PHF2 | ENSG00000197724 | 131.45 | 0.19 | 0.0127 | 0.0454 | 1.42 |
| PHF21A | ENSG00000135365 | 127.45 | 0.19 | 0.0072 | 0.0377 | 1.20 |
| PHF3 | ENSG00000118482 | 433.10 | 0.18 | 0.0158 | 0.0497 | 1.34 |
| PHF6 | ENSG00000156531 | 67.55 | 0.21 | 0.0076 | 0.0382 | 2.34 |
| PHF7 | ENSG00000010318 | 29.80 | 0.18 | 0.0148 | 0.0484 | 0.90 |
| PHKA2 | ENSG00000044446 | 128.05 | 0.20 | 0.0087 | 0.0401 | 1.84 |
| PHKB | ENSG00000102893 | 198.23 | 0.20 | 0.0076 | 0.0382 | 1.52 |
| PHKG2 | ENSG00000156873 | 45.10 | 0.20 | 0.0054 | 0.0362 | 1.56 |
| PHLDB1 | ENSG00000019144 | 597.54 | 0.18 | 0.0121 | 0.0445 | 0.96 |
| PHLPP1 | ENSG00000081913 | 118.56 | 0.24 | 0.0008 | 0.0342 | 0.85 |
| PHPT1 | ENSG00000054148 | 97.48 | 0.19 | 0.0090 | 0.0405 | 0.99 |
| PHRF1 | ENSG00000070047 | 249.46 | 0.19 | 0.0125 | 0.0451 | 1.55 |
| PHYH | ENSG00000107537 | 36.53 | 0.18 | 0.0119 | 0.0442 | 1.30 |
| PI3 | ENSG00000124102 | 32.51 | 0.23 | 0.0033 | 0.0342 | 1.19 |
| PI4K2B | ENSG00000038210 | 88.52 | 0.20 | 0.0107 | 0.0428 | 1.76 |
| PI4KA | ENSG00000241973 | 349.98 | 0.18 | 0.0130 | 0.0458 | 1.15 |
| PIAS1 | ENSG00000033800 | 82.45 | 0.20 | 0.0064 | 0.0370 | 1.14 |
| PIAS3 | ENSG00000131788 | 63.00 | 0.19 | 0.0099 | 0.0417 | 1.48 |
| PIDD | ENSG00000177595 | 59.69 | 0.23 | 0.0042 | 0.0350 | 1.34 |
| PIGF | ENSG00000151665 | 42.09 | 0.18 | 0.0161 | 0.0500 | 1.62 |
| PIGG | ENSG00000174227 | 177.13 | 0.18 | 0.0159 | 0.0498 | 1.27 |
| PIGH | ENSG00000100564 | 26.13 | 0.21 | 0.0053 | 0.0361 | 1.13 |
| PIGM | ENSG00000143315 | 41.28 | 0.22 | 0.0036 | 0.0342 | 1.94 |
| PIGN | ENSG00000197563 | 91.18 | 0.23 | 0.0028 | 0.0342 | 1.03 |
| PIGO | ENSG00000165282 | 77.14 | 0.21 | 0.0039 | 0.0345 | 1.53 |
| PIGP | ENSG00000185808 | 21.64 | 0.21 | 0.0062 | 0.0368 | 1.72 |
| PIGQ | ENSG00000007541 | 91.64 | 0.22 | 0.0036 | 0.0342 | 1.71 |
| PIGR | ENSG00000162896 | 22412.24 | 0.25 | 0.0008 | 0.0342 | 0.29 |
| PIGS | ENSG00000087111 | 95.40 | 0.19 | 0.0076 | 0.0382 | 1.18 |
| PIGT | ENSG00000124155 | 174.62 | 0.18 | 0.0125 | 0.0451 | 1.75 |
| PIGU | ENSG00000101464 | 31.09 | 0.18 | 0.0120 | 0.0444 | 2.86 |
| PIGV | ENSG00000060642 | 26.10 | 0.21 | 0.0046 | 0.0352 | 1.43 |
| PIGY | ENSG00000145337 | 36.19 | 0.19 | 0.0121 | 0.0445 | 1.91 |
| PIGZ | ENSG00000119227 | 187.66 | 0.26 | 0.0016 | 0.0342 | 0.66 |
| PIK3C2B | ENSG00000133056 | 347.87 | 0.20 | 0.0061 | 0.0368 | 1.53 |
| PIK3C3 | ENSG00000078142 | 101.40 | 0.17 | 0.0144 | 0.0477 | 1.05 |
| PIK3CB | ENSG00000051382 | 138.00 | 0.21 | 0.0035 | 0.0342 | 1.58 |
| PIK3R2 | ENSG00000105647 | 99.66 | 0.24 | 0.0010 | 0.0342 | 1.97 |
| PIN1 | ENSG00000127445 | 38.98 | 0.20 | 0.0053 | 0.0361 | 1.46 |
| PIN4 | ENSG00000102309 | 17.51 | 0.21 | 0.0030 | 0.0342 | 2.07 |
| PINK1 | ENSG00000158828 | 148.94 | 0.23 | 0.0020 | 0.0342 | 0.85 |
| PIP4K2B | ENSG00000141720 | 118.85 | 0.19 | 0.0121 | 0.0445 | 1.90 |
| PIP4K2C | ENSG00000166908 | 84.46 | 0.18 | 0.0121 | 0.0445 | 1.47 |
| PIP5K1B | ENSG00000107242 | 226.38 | 0.24 | 0.0015 | 0.0342 | 0.92 |
| PITHD1 | ENSG00000057757 | 57.70 | 0.19 | 0.0112 | 0.0434 | 1.54 |
| PITPNA | ENSG00000174238 | 193.12 | 0.18 | 0.0151 | 0.0487 | 1.24 |
| PITX1 | ENSG00000069011 | 34.12 | 0.22 | 0.0058 | 0.0364 | 1.90 |
| PIWIL4 | ENSG00000134627 | 74.53 | 0.19 | 0.0134 | 0.0463 | 1.56 |
| PJA2 | ENSG00000198961 | 305.09 | 0.21 | 0.0028 | 0.0342 | 0.99 |
| PKD1 | ENSG00000008710 | 463.22 | 0.20 | 0.0098 | 0.0416 | 1.45 |
| PKDCC | ENSG00000162878 | 94.17 | 0.26 | 0.0012 | 0.0342 | 1.80 |
| PKLR | ENSG00000143627 | 10.18 | 0.25 | 0.0011 | 0.0342 | 1.69 |
| PKM2 | ENSG00000067225 | 367.66 | 0.23 | 0.0026 | 0.0342 | 2.95 |
| PKN2 | ENSG00000065243 | 229.82 | 0.20 | 0.0052 | 0.0359 | 1.25 |
| PKP2 | ENSG00000057294 | 333.23 | 0.19 | 0.0104 | 0.0424 | 1.01 |
| PKP3 | ENSG00000184363 | 144.58 | 0.19 | 0.0122 | 0.0446 | 2.07 |
| PKP4 | ENSG00000144283 | 186.51 | 0.24 | 0.0017 | 0.0342 | 2.11 |
| PLA2G10 | ENSG00000069764 | 24.37 | 0.23 | 0.0021 | 0.0342 | 0.37 |
| PLA2G12A | ENSG00000123739 | 28.68 | 0.24 | 0.0027 | 0.0342 | 1.68 |
| PLA2G2A | ENSG00000188257 | 571.39 | 0.22 | 0.0076 | 0.0382 | 0.17 |
| PLA2G4A | ENSG00000116711 | 14.55 | 0.19 | 0.0095 | 0.0413 | 1.40 |
| PLA2G4F | ENSG00000168907 | 65.57 | 0.22 | 0.0058 | 0.0364 | 1.02 |
| PLA2R1 | ENSG00000153246 | 104.26 | 0.20 | 0.0066 | 0.0373 | 1.92 |
| PLAA | ENSG00000137055 | 53.81 | 0.18 | 0.0125 | 0.0451 | 2.08 |
| PLAGL2 | ENSG00000126003 | 126.60 | 0.18 | 0.0138 | 0.0468 | 3.55 |
| PLBD1 | ENSG00000121316 | 85.95 | 0.19 | 0.0096 | 0.0414 | 1.95 |
| PLBD2 | ENSG00000151176 | 50.13 | 0.18 | 0.0150 | 0.0486 | 1.98 |
| PLCB3 | ENSG00000149782 | 174.18 | 0.22 | 0.0037 | 0.0342 | 1.56 |
| PLCB4 | ENSG00000101333 | 243.82 | 0.23 | 0.0036 | 0.0342 | 3.37 |
| PLCG1 | ENSG00000124181 | 207.30 | 0.23 | 0.0029 | 0.0342 | 2.64 |
| PLCH1 | ENSG00000114805 | 77.50 | 0.22 | 0.0026 | 0.0342 | 1.25 |
| PLCXD2 | ENSG00000240891 | 13.69 | 0.20 | 0.0088 | 0.0402 | 1.21 |
| PLD1 | ENSG00000075651 | 237.01 | 0.21 | 0.0032 | 0.0342 | 0.80 |
| PLD2 | ENSG00000129219 | 54.52 | 0.19 | 0.0133 | 0.0462 | 1.31 |
| PLD3 | ENSG00000105223 | 123.02 | 0.19 | 0.0115 | 0.0438 | 1.45 |
| PLEKHA1 | ENSG00000107679 | 151.46 | 0.21 | 0.0052 | 0.0359 | 1.95 |
| PLEKHA3 | ENSG00000116095 | 34.19 | 0.20 | 0.0067 | 0.0373 | 1.73 |
| PLEKHA5 | ENSG00000052126 | 179.23 | 0.19 | 0.0076 | 0.0382 | 1.58 |
| PLEKHA6 | ENSG00000143850 | 468.27 | 0.22 | 0.0033 | 0.0342 | 1.09 |
| PLEKHA7 | ENSG00000166689 | 238.51 | 0.24 | 0.0014 | 0.0342 | 0.97 |
| PLEKHB2 | ENSG00000115762 | 256.83 | 0.22 | 0.0035 | 0.0342 | 1.31 |
| PLEKHG3 | ENSG00000126822 | 269.61 | 0.25 | 0.0006 | 0.0342 | 1.33 |
| PLEKHG4 | ENSG00000196155 | 58.55 | 0.22 | 0.0045 | 0.0352 | 2.96 |
| PLEKHG5 | ENSG00000171680 | 98.36 | 0.22 | 0.0025 | 0.0342 | 2.37 |
| PLEKHH1 | ENSG00000054690 | 362.09 | 0.23 | 0.0025 | 0.0342 | 0.93 |
| PLEKHH3 | ENSG00000068137 | 31.60 | 0.22 | 0.0066 | 0.0373 | 1.78 |
| PLEKHJ1 | ENSG00000104886 | 80.11 | 0.21 | 0.0046 | 0.0352 | 1.41 |
| PLIN3 | ENSG00000105355 | 66.41 | 0.19 | 0.0090 | 0.0405 | 1.40 |
| PLK1 | ENSG00000166851 | 50.58 | 0.21 | 0.0050 | 0.0356 | 1.78 |
| PLK1S1 | ENSG00000088970 | 49.38 | 0.20 | 0.0069 | 0.0375 | 2.02 |
| PLLP | ENSG00000102934 | 29.96 | 0.21 | 0.0067 | 0.0373 | 0.36 |
| PLOD1 | ENSG00000083444 | 83.91 | 0.23 | 0.0014 | 0.0342 | 2.31 |
| PLOD3 | ENSG00000106397 | 97.30 | 0.20 | 0.0066 | 0.0373 | 2.90 |
| PLP2 | ENSG00000102007 | 94.27 | 0.18 | 0.0156 | 0.0494 | 2.83 |
| PLS1 | ENSG00000120756 | 344.39 | 0.24 | 0.0016 | 0.0342 | 1.00 |
| PLS3 | ENSG00000102024 | 47.25 | 0.20 | 0.0079 | 0.0387 | 4.08 |
| PLSCR4 | ENSG00000114698 | 63.15 | 0.18 | 0.0117 | 0.0439 | 0.97 |
| PLXDC2 | ENSG00000120594 | 87.48 | 0.20 | 0.0093 | 0.0409 | 1.35 |
| PLXNA1 | ENSG00000114554 | 147.83 | 0.20 | 0.0082 | 0.0393 | 3.43 |
| PLXNA2 | ENSG00000076356 | 480.02 | 0.22 | 0.0034 | 0.0342 | 0.78 |
| PLXNA3 | ENSG00000130827 | 183.99 | 0.27 | 0.0003 | 0.0342 | 1.87 |
| PLXNB1 | ENSG00000164050 | 404.58 | 0.22 | 0.0039 | 0.0345 | 2.18 |
| PLXNB2 | ENSG00000196576 | 683.18 | 0.24 | 0.0015 | 0.0342 | 1.35 |
| PM20D2 | ENSG00000146281 | 56.80 | 0.21 | 0.0075 | 0.0382 | 2.49 |
| PMF1 | ENSG00000160783 | 45.76 | 0.19 | 0.0107 | 0.0428 | 1.23 |
| PMM1 | ENSG00000100417 | 48.96 | 0.23 | 0.0021 | 0.0342 | 0.96 |
| PMM2 | ENSG00000140650 | 121.70 | 0.23 | 0.0019 | 0.0342 | 1.55 |
| PMPCB | ENSG00000105819 | 82.22 | 0.20 | 0.0057 | 0.0363 | 1.97 |
| PMS1 | ENSG00000064933 | 39.73 | 0.19 | 0.0153 | 0.0489 | 1.84 |
| PMS2 | ENSG00000122512 | 24.13 | 0.20 | 0.0049 | 0.0355 | 1.90 |
| PNKD | ENSG00000127838 | 100.60 | 0.21 | 0.0035 | 0.0342 | 1.75 |
| PNLIPRP1 | ENSG00000187021 | 8.54 | 0.22 | 0.0079 | 0.0387 | 0.45 |
| PNPLA2 | ENSG00000177666 | 240.14 | 0.23 | 0.0047 | 0.0353 | 1.02 |
| PNPLA6 | ENSG00000032444 | 115.98 | 0.20 | 0.0108 | 0.0429 | 1.48 |
| PNPLA7 | ENSG00000130653 | 131.44 | 0.22 | 0.0036 | 0.0342 | 0.49 |
| PNPO | ENSG00000108439 | 113.31 | 0.26 | 0.0007 | 0.0342 | 1.51 |
| PNRC2 | ENSG00000189266 | 121.30 | 0.21 | 0.0059 | 0.0365 | 1.31 |
| POC1B | ENSG00000139323 | 268.89 | 0.24 | 0.0009 | 0.0342 | 2.22 |
| PODXL | ENSG00000128567 | 136.08 | 0.22 | 0.0033 | 0.0342 | 2.85 |
| POF1B | ENSG00000124429 | 351.28 | 0.34 | 0.0002 | 0.0342 | 1.83 |
| POFUT1 | ENSG00000101346 | 146.38 | 0.29 | 0.0002 | 0.0342 | 3.72 |
| POGZ | ENSG00000143442 | 359.38 | 0.21 | 0.0053 | 0.0361 | 1.22 |
| POLD4 | ENSG00000175482 | 146.55 | 0.19 | 0.0093 | 0.0409 | 0.83 |
| POLDIP2 | ENSG00000004142 | 104.46 | 0.19 | 0.0099 | 0.0417 | 2.10 |
| POLDIP3 | ENSG00000100227 | 125.15 | 0.19 | 0.0095 | 0.0413 | 1.38 |
| POLE | ENSG00000177084 | 236.33 | 0.21 | 0.0046 | 0.0352 | 1.66 |
| POLL | ENSG00000166169 | 83.51 | 0.27 | 0.0004 | 0.0342 | 1.36 |
| POLR1A | ENSG00000068654 | 118.79 | 0.22 | 0.0035 | 0.0342 | 2.62 |
| POLR2A | ENSG00000181222 | 347.96 | 0.20 | 0.0068 | 0.0374 | 1.30 |
| POLR2H | ENSG00000163882 | 98.43 | 0.22 | 0.0036 | 0.0342 | 1.33 |
| POLR2J | ENSG00000005075 | 22.31 | 0.19 | 0.0109 | 0.0431 | 1.73 |
| POLR2J2 | ENSG00000228049 | 96.92 | 0.19 | 0.0148 | 0.0484 | 1.44 |
| POLR2L | ENSG00000177700 | 42.76 | 0.22 | 0.0032 | 0.0342 | 1.60 |
| POLR3B | ENSG00000013503 | 34.56 | 0.20 | 0.0062 | 0.0368 | 1.41 |
| POLR3GL | ENSG00000121851 | 24.72 | 0.22 | 0.0048 | 0.0354 | 1.11 |
| POLR3H | ENSG00000100413 | 193.43 | 0.20 | 0.0074 | 0.0380 | 1.10 |
| POMP | ENSG00000132963 | 35.36 | 0.20 | 0.0056 | 0.0363 | 3.49 |
| POMT1 | ENSG00000130714 | 70.83 | 0.19 | 0.0110 | 0.0432 | 1.15 |
| POMT2 | ENSG00000009830 | 50.29 | 0.18 | 0.0148 | 0.0484 | 1.38 |
| PON2 | ENSG00000105854 | 139.11 | 0.22 | 0.0025 | 0.0342 | 1.64 |
| PON3 | ENSG00000105852 | 15.15 | 0.18 | 0.0108 | 0.0429 | 0.94 |
| POP4 | ENSG00000105171 | 19.31 | 0.18 | 0.0111 | 0.0433 | 1.93 |
| POP7 | ENSG00000172336 | 11.73 | 0.22 | 0.0051 | 0.0358 | 2.49 |
| PORCN | ENSG00000102312 | 12.60 | 0.26 | 0.0021 | 0.0342 | 1.91 |
| POSTN | ENSG00000133110 | 161.01 | 0.20 | 0.0064 | 0.0370 | 2.71 |
| PPA1 | ENSG00000180817 | 87.67 | 0.23 | 0.0017 | 0.0342 | 3.51 |
| PPA2 | ENSG00000138777 | 82.33 | 0.18 | 0.0106 | 0.0427 | 1.37 |
| PPAP2C | ENSG00000141934 | 54.45 | 0.28 | <.0001 | 0.0342 | 2.46 |
| PPAPDC1B | ENSG00000147535 | 133.94 | 0.22 | 0.0018 | 0.0342 | 1.05 |
| PPAPDC2 | ENSG00000205808 | 54.59 | 0.31 | <.0001 | 0.0342 | 1.54 |
| PPARA | ENSG00000186951 | 319.40 | 0.25 | 0.0009 | 0.0342 | 1.23 |
| PPARGC1A | ENSG00000109819 | 187.43 | 0.21 | 0.0052 | 0.0359 | 0.43 |
| PPARGC1B | ENSG00000155846 | 339.13 | 0.21 | 0.0051 | 0.0358 | 0.65 |
| PPCDC | ENSG00000138621 | 24.88 | 0.19 | 0.0109 | 0.0431 | 1.21 |
| PPCS | ENSG00000127125 | 66.71 | 0.23 | 0.0016 | 0.0342 | 1.33 |
| PPEF1 | ENSG00000086717 | 1.96 | 0.20 | 0.0138 | 0.0468 | 2.64 |
| PPFIA1 | ENSG00000131626 | 237.25 | 0.21 | 0.0051 | 0.0358 | 1.38 |
| PPFIA3 | ENSG00000177380 | 26.36 | 0.21 | 0.0075 | 0.0382 | 2.18 |
| PPFIBP2 | ENSG00000166387 | 146.26 | 0.24 | 0.0017 | 0.0342 | 1.27 |
| PPIB | ENSG00000166794 | 179.45 | 0.18 | 0.0124 | 0.0450 | 1.87 |
| PPIC | ENSG00000168938 | 51.74 | 0.20 | 0.0069 | 0.0375 | 1.12 |
| PPID | ENSG00000171497 | 44.26 | 0.20 | 0.0086 | 0.0399 | 1.88 |
| PPIF | ENSG00000108179 | 89.90 | 0.18 | 0.0134 | 0.0463 | 2.17 |
| PPIL2 | ENSG00000100023 | 164.82 | 0.21 | 0.0061 | 0.0368 | 1.19 |
| PPIP5K1 | ENSG00000168781 | 165.23 | 0.20 | 0.0078 | 0.0386 | 0.99 |
| PPM1A | ENSG00000100614 | 233.88 | 0.19 | 0.0108 | 0.0429 | 1.10 |
| PPM1B | ENSG00000138032 | 166.36 | 0.20 | 0.0060 | 0.0366 | 1.16 |
| PPM1G | ENSG00000115241 | 95.20 | 0.18 | 0.0157 | 0.0496 | 2.33 |
| PPM1H | ENSG00000111110 | 46.85 | 0.22 | 0.0036 | 0.0342 | 5.07 |
| PPM1J | ENSG00000155367 | 56.19 | 0.18 | 0.0122 | 0.0446 | 1.21 |
| PPM1L | ENSG00000163590 | 116.32 | 0.24 | 0.0007 | 0.0342 | 0.80 |
| PPME1 | ENSG00000214517 | 93.09 | 0.19 | 0.0122 | 0.0446 | 1.80 |
| PPP1CA | ENSG00000172531 | 122.91 | 0.19 | 0.0108 | 0.0429 | 1.48 |
| PPP1CB | ENSG00000213639 | 391.11 | 0.19 | 0.0119 | 0.0442 | 1.38 |
| PPP1R11 | ENSG00000204619 | 131.40 | 0.19 | 0.0103 | 0.0423 | 1.28 |
| PPP1R12B | ENSG00000077157 | 886.07 | 0.18 | 0.0138 | 0.0468 | 0.64 |
| PPP1R13B | ENSG00000088808 | 139.40 | 0.18 | 0.0130 | 0.0458 | 0.90 |
| PPP1R13L | ENSG00000104881 | 44.00 | 0.26 | 0.0011 | 0.0342 | 2.43 |
| PPP1R16A | ENSG00000160972 | 142.21 | 0.21 | 0.0091 | 0.0407 | 1.49 |
| PPP1R1B | ENSG00000131771 | 283.30 | 0.22 | 0.0023 | 0.0342 | 1.42 |
| PPP1R21 | ENSG00000162869 | 55.41 | 0.21 | 0.0040 | 0.0346 | 1.52 |
| PPP1R26 | ENSG00000196422 | 82.18 | 0.28 | <.0001 | 0.0342 | 1.78 |
| PPP1R36 | ENSG00000165807 | 11.03 | 0.20 | 0.0081 | 0.0391 | 0.80 |
| PPP1R39 | ENSG00000156463 | 322.82 | 0.23 | 0.0011 | 0.0342 | 0.76 |
| PPP1R3B | ENSG00000173281 | 72.77 | 0.27 | 0.0011 | 0.0342 | 1.79 |
| PPP1R3E | ENSG00000235194 | 60.95 | 0.26 | 0.0007 | 0.0342 | 1.07 |
| PPP1R7 | ENSG00000115685 | 56.74 | 0.19 | 0.0111 | 0.0433 | 1.67 |
| PPP1R8 | ENSG00000117751 | 48.71 | 0.19 | 0.0104 | 0.0424 | 1.73 |
| PPP1R9A | ENSG00000158528 | 167.84 | 0.22 | 0.0031 | 0.0342 | 1.12 |
| PPP2R1B | ENSG00000137713 | 132.40 | 0.18 | 0.0124 | 0.0450 | 1.49 |
| PPP2R3B | ENSG00000167393 | 15.45 | 0.19 | 0.0144 | 0.0477 | 2.48 |
| PPP2R3C | ENSG00000092020 | 27.09 | 0.22 | 0.0021 | 0.0342 | 1.34 |
| PPP2R4 | ENSG00000119383 | 130.89 | 0.25 | 0.0008 | 0.0342 | 2.06 |
| PPP2R5A | ENSG00000066027 | 130.17 | 0.25 | 0.0006 | 0.0342 | 1.52 |
| PPP2R5E | ENSG00000154001 | 138.62 | 0.24 | 0.0020 | 0.0342 | 1.23 |
| PPP3CA | ENSG00000138814 | 131.47 | 0.18 | 0.0116 | 0.0439 | 1.48 |
| PPP3CB | ENSG00000107758 | 96.06 | 0.18 | 0.0148 | 0.0484 | 1.34 |
| PPP4R1 | ENSG00000154845 | 120.01 | 0.19 | 0.0102 | 0.0422 | 1.22 |
| PPP4R2 | ENSG00000163605 | 112.40 | 0.19 | 0.0065 | 0.0371 | 1.81 |
| PPP5C | ENSG00000011485 | 101.23 | 0.20 | 0.0072 | 0.0377 | 1.50 |
| PPP6C | ENSG00000119414 | 102.59 | 0.20 | 0.0072 | 0.0377 | 1.47 |
| PPP6R2 | ENSG00000100239 | 225.85 | 0.20 | 0.0079 | 0.0387 | 1.12 |
| PPP6R3 | ENSG00000110075 | 359.21 | 0.20 | 0.0077 | 0.0384 | 1.39 |
| PPPDE2 | ENSG00000100418 | 119.98 | 0.19 | 0.0135 | 0.0464 | 1.68 |
| PPT1 | ENSG00000131238 | 74.22 | 0.19 | 0.0109 | 0.0431 | 2.57 |
| PPT2 | ENSG00000221988 | 120.30 | 0.21 | 0.0053 | 0.0361 | 1.36 |
| PPYR1 | ENSG00000204174 | 86.80 | 0.26 | 0.0022 | 0.0342 | 0.34 |
| PQLC1 | ENSG00000122490 | 105.86 | 0.19 | 0.0098 | 0.0416 | 0.65 |
| PRAC | ENSG00000159182 | 26.34 | 0.21 | 0.0071 | 0.0376 | 0.78 |
| PRDM4 | ENSG00000110851 | 117.41 | 0.18 | 0.0111 | 0.0433 | 1.69 |
| PRDX1 | ENSG00000117450 | 106.14 | 0.21 | 0.0058 | 0.0364 | 2.85 |
| PRDX2 | ENSG00000167815 | 76.77 | 0.22 | 0.0038 | 0.0342 | 2.17 |
| PRDX3 | ENSG00000165672 | 64.07 | 0.22 | 0.0031 | 0.0342 | 1.86 |
| PRDX4 | ENSG00000123131 | 25.67 | 0.22 | 0.0047 | 0.0353 | 3.15 |
| PRDX5 | ENSG00000126432 | 108.13 | 0.18 | 0.0151 | 0.0487 | 2.62 |
| PRDX6 | ENSG00000117592 | 234.53 | 0.22 | 0.0029 | 0.0342 | 0.85 |
| PREB | ENSG00000138073 | 83.04 | 0.19 | 0.0108 | 0.0429 | 1.80 |
| PRELID1 | ENSG00000169230 | 79.02 | 0.19 | 0.0104 | 0.0424 | 1.68 |
| PRELID2 | ENSG00000186314 | 124.11 | 0.26 | 0.0006 | 0.0342 | 1.29 |
| PREP | ENSG00000085377 | 157.10 | 0.18 | 0.0097 | 0.0415 | 1.76 |
| PREPL | ENSG00000138078 | 147.62 | 0.21 | 0.0026 | 0.0342 | 1.63 |
| PREX2 | ENSG00000046889 | 64.12 | 0.18 | 0.0153 | 0.0489 | 0.65 |
| PRICKLE3 | ENSG00000012211 | 51.54 | 0.19 | 0.0079 | 0.0387 | 2.35 |
| PRIM1 | ENSG00000198056 | 9.14 | 0.21 | 0.0064 | 0.0370 | 3.04 |
| PRKAA1 | ENSG00000132356 | 201.71 | 0.23 | 0.0026 | 0.0342 | 1.34 |
| PRKAA2 | ENSG00000162409 | 38.68 | 0.21 | 0.0060 | 0.0366 | 0.55 |
| PRKAB1 | ENSG00000111725 | 224.45 | 0.19 | 0.0114 | 0.0437 | 1.39 |
| PRKAB2 | ENSG00000131791 | 88.31 | 0.32 | <.0001 | 0.0342 | 1.69 |
| PRKACB | ENSG00000142875 | 268.35 | 0.22 | 0.0036 | 0.0342 | 0.57 |
| PRKAG1 | ENSG00000181929 | 57.82 | 0.20 | 0.0085 | 0.0399 | 1.79 |
| PRKAG2 | ENSG00000106617 | 111.88 | 0.19 | 0.0092 | 0.0408 | 0.77 |
| PRKAR1B | ENSG00000188191 | 36.39 | 0.18 | 0.0144 | 0.0477 | 2.56 |
| PRKAR2A | ENSG00000114302 | 227.10 | 0.19 | 0.0075 | 0.0382 | 1.60 |
| PRKCA | ENSG00000154229 | 144.54 | 0.18 | 0.0152 | 0.0489 | 1.21 |
| PRKCD | ENSG00000163932 | 115.39 | 0.20 | 0.0055 | 0.0363 | 1.07 |
| PRKCI | ENSG00000163558 | 112.17 | 0.21 | 0.0051 | 0.0358 | 1.71 |
| PRKCSH | ENSG00000130175 | 211.53 | 0.21 | 0.0051 | 0.0358 | 1.67 |
| PRKCZ | ENSG00000067606 | 107.52 | 0.20 | 0.0068 | 0.0374 | 1.20 |
| PRKDC | ENSG00000253729 | 673.30 | 0.18 | 0.0116 | 0.0439 | 3.43 |
| PRKRIR | ENSG00000137492 | 65.84 | 0.18 | 0.0147 | 0.0482 | 1.78 |
| PRKX | ENSG00000183943 | 78.11 | 0.18 | 0.0109 | 0.0431 | 1.77 |
| PRLR | ENSG00000113494 | 169.24 | 0.23 | 0.0017 | 0.0342 | 1.57 |
| PRMT1 | ENSG00000126457 | 64.18 | 0.18 | 0.0128 | 0.0456 | 2.42 |
| PRMT2 | ENSG00000160310 | 145.11 | 0.17 | 0.0157 | 0.0496 | 1.27 |
| PRMT3 | ENSG00000185238 | 38.60 | 0.19 | 0.0141 | 0.0472 | 2.53 |
| PRMT5 | ENSG00000100462 | 60.62 | 0.21 | 0.0056 | 0.0363 | 2.30 |
| PRMT7 | ENSG00000132600 | 40.92 | 0.21 | 0.0060 | 0.0366 | 1.79 |
| PROM2 | ENSG00000155066 | 129.11 | 0.20 | 0.0081 | 0.0391 | 0.62 |
| PROSC | ENSG00000147471 | 75.39 | 0.26 | 0.0011 | 0.0342 | 1.41 |
| PROSER1 | ENSG00000120685 | 162.35 | 0.18 | 0.0154 | 0.0491 | 2.97 |
| PROX1 | ENSG00000117707 | 56.02 | 0.25 | 0.0037 | 0.0342 | 6.70 |
| PRPF18 | ENSG00000165630 | 31.95 | 0.17 | 0.0159 | 0.0498 | 1.51 |
| PRPF19 | ENSG00000110107 | 89.63 | 0.19 | 0.0103 | 0.0423 | 2.35 |
| PRPF3 | ENSG00000117360 | 135.50 | 0.18 | 0.0155 | 0.0492 | 1.43 |
| PRPF4 | ENSG00000136875 | 42.40 | 0.20 | 0.0086 | 0.0399 | 2.38 |
| PRPF6 | ENSG00000101161 | 112.55 | 0.19 | 0.0089 | 0.0403 | 2.78 |
| PRPF8 | ENSG00000174231 | 643.24 | 0.20 | 0.0070 | 0.0376 | 1.54 |
| PRPS2 | ENSG00000101911 | 55.93 | 0.20 | 0.0065 | 0.0371 | 2.74 |
| PRPSAP1 | ENSG00000161542 | 46.25 | 0.23 | 0.0016 | 0.0342 | 1.79 |
| PRPSAP2 | ENSG00000141127 | 35.38 | 0.18 | 0.0110 | 0.0432 | 1.21 |
| PRR12 | ENSG00000126464 | 204.07 | 0.21 | 0.0061 | 0.0368 | 1.57 |
| PRR13 | ENSG00000205352 | 113.85 | 0.22 | 0.0026 | 0.0342 | 1.37 |
| PRR14 | ENSG00000156858 | 51.72 | 0.19 | 0.0116 | 0.0439 | 1.68 |
| PRR14L | ENSG00000183530 | 432.47 | 0.21 | 0.0043 | 0.0351 | 1.23 |
| PRR15 | ENSG00000176532 | 103.71 | 0.19 | 0.0134 | 0.0463 | 1.85 |
| PRR15L | ENSG00000167183 | 164.53 | 0.34 | 0.0002 | 0.0342 | 1.36 |
| PRR5 | ENSG00000186654 | 74.38 | 0.20 | 0.0098 | 0.0416 | 1.49 |
| PRR5-ARHGAP8 | ENSG00000248405 | 81.97 | 0.21 | 0.0043 | 0.0351 | 1.62 |
| PRRC1 | ENSG00000164244 | 159.27 | 0.25 | 0.0006 | 0.0342 | 1.54 |
| PRRC2A | ENSG00000204469 | 629.29 | 0.19 | 0.0096 | 0.0414 | 2.08 |
| PRRC2B | ENSG00000130723 | 737.76 | 0.20 | 0.0065 | 0.0371 | 1.95 |
| PRRG2 | ENSG00000126460 | 15.81 | 0.22 | 0.0048 | 0.0354 | 1.22 |
| PRRG4 | ENSG00000135378 | 50.16 | 0.29 | <.0001 | 0.0342 | 1.24 |
| PRSS12 | ENSG00000164099 | 137.10 | 0.21 | 0.0063 | 0.0370 | 0.56 |
| PRSS16 | ENSG00000112812 | 34.06 | 0.20 | 0.0063 | 0.0370 | 1.23 |
| PRSS23 | ENSG00000150687 | 105.11 | 0.19 | 0.0102 | 0.0422 | 2.89 |
| PRSS3 | ENSG00000010438 | 72.65 | 0.21 | 0.0055 | 0.0363 | 1.04 |
| PRSS48 | ENSG00000189099 | 69.23 | 0.21 | 0.0065 | 0.0371 | 0.98 |
| PRSS8 | ENSG00000052344 | 261.85 | 0.23 | 0.0027 | 0.0342 | 1.05 |
| PRUNE | ENSG00000143363 | 42.33 | 0.24 | 0.0010 | 0.0342 | 1.55 |
| PSAP | ENSG00000197746 | 716.67 | 0.18 | 0.0119 | 0.0442 | 1.61 |
| PSAT1 | ENSG00000135069 | 9.80 | 0.19 | 0.0155 | 0.0492 | 6.84 |
| PSD3 | ENSG00000156011 | 244.94 | 0.18 | 0.0119 | 0.0442 | 1.09 |
| PSEN1 | ENSG00000080815 | 204.53 | 0.20 | 0.0052 | 0.0359 | 1.01 |
| PSEN2 | ENSG00000143801 | 33.98 | 0.22 | 0.0030 | 0.0342 | 1.41 |
| PSKH1 | ENSG00000159792 | 89.42 | 0.20 | 0.0070 | 0.0376 | 1.15 |
| PSMA2 | ENSG00000106588 | 53.87 | 0.20 | 0.0058 | 0.0364 | 2.65 |
| PSMA3 | ENSG00000100567 | 49.98 | 0.19 | 0.0094 | 0.0411 | 1.84 |
| PSMA4 | ENSG00000041357 | 46.82 | 0.20 | 0.0074 | 0.0380 | 2.24 |
| PSMA6 | ENSG00000100902 | 33.90 | 0.19 | 0.0080 | 0.0389 | 2.26 |
| PSMA7 | ENSG00000101182 | 63.39 | 0.18 | 0.0107 | 0.0428 | 3.61 |
| PSMB1 | ENSG00000008018 | 74.69 | 0.23 | 0.0020 | 0.0342 | 2.13 |
| PSMB2 | ENSG00000126067 | 77.74 | 0.21 | 0.0056 | 0.0363 | 2.24 |
| PSMB3 | ENSG00000108294 | 33.16 | 0.21 | 0.0044 | 0.0351 | 2.32 |
| PSMB4 | ENSG00000159377 | 56.03 | 0.18 | 0.0159 | 0.0498 | 2.21 |
| PSMB5 | ENSG00000100804 | 35.31 | 0.20 | 0.0067 | 0.0373 | 2.50 |
| PSMB6 | ENSG00000142507 | 27.15 | 0.21 | 0.0046 | 0.0352 | 1.89 |
| PSMB7 | ENSG00000136930 | 92.20 | 0.25 | 0.0015 | 0.0342 | 1.87 |
| PSMB8 | ENSG00000204264 | 66.82 | 0.22 | 0.0057 | 0.0363 | 2.06 |
| PSMB9 | ENSG00000240065 | 29.86 | 0.21 | 0.0110 | 0.0432 | 2.36 |
| PSMC2 | ENSG00000161057 | 38.54 | 0.26 | 0.0008 | 0.0342 | 2.75 |
| PSMC3 | ENSG00000165916 | 53.94 | 0.18 | 0.0152 | 0.0489 | 1.94 |
| PSMC6 | ENSG00000100519 | 38.41 | 0.18 | 0.0155 | 0.0492 | 1.86 |
| PSMD1 | ENSG00000173692 | 140.67 | 0.21 | 0.0040 | 0.0346 | 2.08 |
| PSMD10 | ENSG00000101843 | 22.22 | 0.20 | 0.0071 | 0.0376 | 2.16 |
| PSMD11 | ENSG00000108671 | 107.81 | 0.18 | 0.0141 | 0.0472 | 2.01 |
| PSMD13 | ENSG00000185627 | 74.47 | 0.20 | 0.0055 | 0.0363 | 2.00 |
| PSMD3 | ENSG00000108344 | 128.04 | 0.19 | 0.0071 | 0.0376 | 1.90 |
| PSMD4 | ENSG00000159352 | 50.95 | 0.21 | 0.0047 | 0.0353 | 2.12 |
| PSMD9 | ENSG00000110801 | 55.40 | 0.18 | 0.0158 | 0.0497 | 1.46 |
| PSME1 | ENSG00000092010 | 75.42 | 0.24 | 0.0017 | 0.0342 | 1.73 |
| PSMF1 | ENSG00000125818 | 135.45 | 0.18 | 0.0130 | 0.0458 | 1.96 |
| PSMG1 | ENSG00000183527 | 25.31 | 0.20 | 0.0116 | 0.0439 | 3.07 |
| PSMG2 | ENSG00000128789 | 30.48 | 0.19 | 0.0129 | 0.0457 | 1.99 |
| PSMG3 | ENSG00000157778 | 24.87 | 0.18 | 0.0137 | 0.0467 | 2.14 |
| PSMG4 | ENSG00000180822 | 259.52 | 0.23 | 0.0017 | 0.0342 | 0.94 |
| PSORS1C1 | ENSG00000204540 | 39.15 | 0.24 | 0.0053 | 0.0361 | 1.68 |
| PSRC1 | ENSG00000134222 | 7.30 | 0.23 | 0.0029 | 0.0342 | 4.10 |
| PTAR1 | ENSG00000188647 | 355.93 | 0.20 | 0.0055 | 0.0363 | 1.16 |
| PTBP1 | ENSG00000011304 | 325.82 | 0.21 | 0.0051 | 0.0358 | 1.93 |
| PTCD1 | ENSG00000106246 | 72.54 | 0.18 | 0.0153 | 0.0489 | 2.12 |
| PTCH1 | ENSG00000185920 | 252.41 | 0.19 | 0.0113 | 0.0435 | 1.36 |
| PTDSS1 | ENSG00000156471 | 78.50 | 0.18 | 0.0138 | 0.0468 | 2.56 |
| PTDSS2 | ENSG00000174915 | 42.11 | 0.19 | 0.0116 | 0.0439 | 1.90 |
| PTEN | ENSG00000171862 | 256.99 | 0.20 | 0.0076 | 0.0382 | 1.16 |
| PTER | ENSG00000165983 | 42.33 | 0.19 | 0.0122 | 0.0446 | 1.49 |
| PTGES2 | ENSG00000148334 | 81.05 | 0.24 | 0.0022 | 0.0342 | 1.98 |
| PTGES3 | ENSG00000110958 | 206.47 | 0.21 | 0.0062 | 0.0368 | 2.65 |
| PTGFRN | ENSG00000134247 | 237.36 | 0.24 | 0.0007 | 0.0342 | 1.54 |
| PTGR1 | ENSG00000106853 | 41.23 | 0.23 | 0.0016 | 0.0342 | 1.14 |
| PTGR2 | ENSG00000140043 | 28.84 | 0.18 | 0.0149 | 0.0485 | 1.24 |
| PTK2 | ENSG00000169398 | 315.29 | 0.21 | 0.0032 | 0.0342 | 2.02 |
| PTK6 | ENSG00000101213 | 159.99 | 0.23 | 0.0027 | 0.0342 | 1.34 |
| PTK7 | ENSG00000112655 | 53.82 | 0.18 | 0.0138 | 0.0468 | 5.13 |
| PTOV1 | ENSG00000104960 | 108.66 | 0.20 | 0.0092 | 0.0408 | 1.42 |
| PTP4A2 | ENSG00000184007 | 416.05 | 0.20 | 0.0058 | 0.0364 | 1.39 |
| PTPDC1 | ENSG00000158079 | 50.14 | 0.19 | 0.0112 | 0.0434 | 1.87 |
| PTPLAD1 | ENSG00000074696 | 88.21 | 0.25 | 0.0017 | 0.0342 | 2.92 |
| PTPLB | ENSG00000206527 | 133.13 | 0.22 | 0.0027 | 0.0342 | 1.45 |
| PTPMT1 | ENSG00000110536 | 45.10 | 0.18 | 0.0141 | 0.0472 | 1.72 |
| PTPN11 | ENSG00000179295 | 216.99 | 0.22 | 0.0028 | 0.0342 | 2.52 |
| PTPN12 | ENSG00000127947 | 166.60 | 0.19 | 0.0112 | 0.0434 | 2.43 |
| PTPN13 | ENSG00000163629 | 58.31 | 0.22 | 0.0106 | 0.0427 | 2.92 |
| PTPN2 | ENSG00000175354 | 55.01 | 0.21 | 0.0039 | 0.0345 | 1.42 |
| PTPN21 | ENSG00000070778 | 140.01 | 0.21 | 0.0024 | 0.0342 | 0.68 |
| PTPN3 | ENSG00000070159 | 207.23 | 0.19 | 0.0102 | 0.0422 | 1.78 |
| PTPN9 | ENSG00000169410 | 99.61 | 0.20 | 0.0057 | 0.0363 | 1.36 |
| PTPRB | ENSG00000127329 | 187.84 | 0.18 | 0.0133 | 0.0462 | 1.32 |
| PTPRF | ENSG00000142949 | 1289.70 | 0.23 | 0.0019 | 0.0342 | 0.95 |
| PTPRG | ENSG00000144724 | 239.65 | 0.19 | 0.0113 | 0.0435 | 1.82 |
| PTPRH | ENSG00000080031 | 266.19 | 0.21 | 0.0052 | 0.0359 | 0.38 |
| PTPRJ | ENSG00000149177 | 283.83 | 0.19 | 0.0098 | 0.0416 | 1.35 |
| PTPRK | ENSG00000152894 | 434.63 | 0.22 | 0.0032 | 0.0342 | 1.38 |
| PTPRM | ENSG00000173482 | 132.64 | 0.18 | 0.0154 | 0.0491 | 0.89 |
| PTPRN2 | ENSG00000155093 | 89.29 | 0.19 | 0.0135 | 0.0464 | 1.14 |
| PTPRR | ENSG00000153233 | 24.40 | 0.19 | 0.0160 | 0.0499 | 1.13 |
| PTTG1IP | ENSG00000183255 | 361.79 | 0.25 | 0.0008 | 0.0342 | 1.22 |
| PUM1 | ENSG00000134644 | 291.47 | 0.19 | 0.0118 | 0.0441 | 1.56 |
| PUM2 | ENSG00000055917 | 400.06 | 0.20 | 0.0046 | 0.0352 | 1.84 |
| PURA | ENSG00000185129 | 66.10 | 0.20 | 0.0100 | 0.0419 | 1.28 |
| PUS3 | ENSG00000110060 | 15.01 | 0.20 | 0.0111 | 0.0433 | 2.02 |
| PVR | ENSG00000073008 | 200.95 | 0.17 | 0.0160 | 0.0499 | 1.50 |
| PVRL1 | ENSG00000110400 | 115.58 | 0.21 | 0.0054 | 0.0362 | 2.58 |
| PVRL2 | ENSG00000130202 | 135.58 | 0.18 | 0.0150 | 0.0486 | 1.73 |
| PVRL3 | ENSG00000177707 | 98.38 | 0.25 | 0.0004 | 0.0342 | 0.90 |
| PVRL4 | ENSG00000143217 | 23.05 | 0.24 | 0.0029 | 0.0342 | 2.51 |
| PWWP2A | ENSG00000170234 | 105.18 | 0.25 | 0.0006 | 0.0342 | 1.22 |
| PXMP2 | ENSG00000176894 | 37.03 | 0.21 | 0.0034 | 0.0342 | 0.84 |
| PXN | ENSG00000089159 | 421.18 | 0.18 | 0.0129 | 0.0457 | 1.27 |
| PYCR1 | ENSG00000183010 | 44.66 | 0.24 | 0.0017 | 0.0342 | 3.40 |
| PYCR2 | ENSG00000143811 | 135.07 | 0.21 | 0.0071 | 0.0376 | 1.41 |
| PYGB | ENSG00000100994 | 674.85 | 0.23 | 0.0010 | 0.0342 | 1.87 |
| PYGO2 | ENSG00000163348 | 94.96 | 0.22 | 0.0032 | 0.0342 | 1.68 |
| PYROXD1 | ENSG00000121350 | 40.71 | 0.19 | 0.0092 | 0.0408 | 1.40 |
| QARS | ENSG00000172053 | 264.48 | 0.20 | 0.0050 | 0.0356 | 1.63 |
| QPRT | ENSG00000103485 | 36.20 | 0.23 | 0.0016 | 0.0342 | 2.80 |
| QSOX1 | ENSG00000116260 | 545.60 | 0.23 | 0.0010 | 0.0342 | 0.90 |
| R3HCC1 | ENSG00000104679 | 29.30 | 0.18 | 0.0161 | 0.0500 | 1.31 |
| R3HDM2 | ENSG00000179912 | 212.41 | 0.20 | 0.0054 | 0.0362 | 1.75 |
| R3HDML | ENSG00000101074 | 19.45 | 0.19 | 0.0087 | 0.0401 | 3.41 |
| RAB10 | ENSG00000084733 | 212.90 | 0.19 | 0.0079 | 0.0387 | 1.75 |
| RAB11A | ENSG00000103769 | 218.09 | 0.20 | 0.0073 | 0.0379 | 1.26 |
| RAB11FIP1 | ENSG00000156675 | 569.68 | 0.20 | 0.0065 | 0.0371 | 1.61 |
| RAB11FIP4 | ENSG00000131242 | 146.44 | 0.18 | 0.0128 | 0.0456 | 1.60 |
| RAB13 | ENSG00000143545 | 38.61 | 0.19 | 0.0114 | 0.0437 | 1.52 |
| RAB14 | ENSG00000119396 | 161.17 | 0.20 | 0.0075 | 0.0382 | 1.55 |
| RAB15 | ENSG00000139998 | 107.31 | 0.26 | 0.0011 | 0.0342 | 2.56 |
| RAB17 | ENSG00000124839 | 57.50 | 0.25 | 0.0021 | 0.0342 | 1.61 |
| RAB18 | ENSG00000099246 | 82.42 | 0.19 | 0.0112 | 0.0434 | 1.53 |
| RAB19 | ENSG00000146955 | 8.66 | 0.27 | 0.0006 | 0.0342 | 1.35 |
| RAB1B | ENSG00000174903 | 150.62 | 0.20 | 0.0073 | 0.0379 | 1.12 |
| RAB22A | ENSG00000124209 | 172.33 | 0.23 | 0.0020 | 0.0342 | 2.63 |
| RAB24 | ENSG00000169228 | 37.99 | 0.23 | 0.0023 | 0.0342 | 1.32 |
| RAB25 | ENSG00000132698 | 80.73 | 0.23 | 0.0023 | 0.0342 | 1.70 |
| RAB27A | ENSG00000069974 | 96.35 | 0.22 | 0.0061 | 0.0368 | 0.64 |
| RAB27B | ENSG00000041353 | 55.93 | 0.22 | 0.0045 | 0.0352 | 0.86 |
| RAB2A | ENSG00000104388 | 137.88 | 0.19 | 0.0084 | 0.0397 | 1.77 |
| RAB3D | ENSG00000105514 | 104.35 | 0.23 | 0.0017 | 0.0342 | 1.94 |
| RAB3GAP1 | ENSG00000115839 | 144.61 | 0.23 | 0.0014 | 0.0342 | 1.45 |
| RAB3GAP2 | ENSG00000118873 | 160.78 | 0.17 | 0.0146 | 0.0481 | 1.63 |
| RAB3IP | ENSG00000127328 | 68.10 | 0.20 | 0.0067 | 0.0373 | 1.89 |
| RAB40C | ENSG00000197562 | 70.85 | 0.21 | 0.0071 | 0.0376 | 1.74 |
| RAB4A | ENSG00000168118 | 59.85 | 0.19 | 0.0112 | 0.0434 | 1.68 |
| RAB5A | ENSG00000144566 | 101.23 | 0.20 | 0.0089 | 0.0403 | 1.60 |
| RAB5B | ENSG00000111540 | 298.91 | 0.23 | 0.0015 | 0.0342 | 1.31 |
| RAB5C | ENSG00000108774 | 126.21 | 0.19 | 0.0087 | 0.0401 | 1.67 |
| RAB6A | ENSG00000175582 | 173.66 | 0.21 | 0.0028 | 0.0342 | 1.68 |
| RABAC1 | ENSG00000105404 | 36.11 | 0.20 | 0.0096 | 0.0414 | 1.13 |
| RABEP2 | ENSG00000177548 | 59.71 | 0.19 | 0.0116 | 0.0439 | 1.35 |
| RABGAP1 | ENSG00000011454 | 250.54 | 0.20 | 0.0069 | 0.0375 | 1.25 |
| RABGEF1 | ENSG00000154710 | 72.94 | 0.19 | 0.0099 | 0.0417 | 1.61 |
| RABGGTA | ENSG00000100949 | 29.10 | 0.21 | 0.0055 | 0.0363 | 1.41 |
| RABL2B | ENSG00000079974 | 51.33 | 0.20 | 0.0080 | 0.0389 | 1.36 |
| RAC1 | ENSG00000136238 | 243.16 | 0.22 | 0.0036 | 0.0342 | 1.97 |
| RAD18 | ENSG00000070950 | 51.59 | 0.19 | 0.0135 | 0.0464 | 2.60 |
| RAD21 | ENSG00000164754 | 236.41 | 0.21 | 0.0037 | 0.0342 | 2.50 |
| RAD23A | ENSG00000179262 | 106.10 | 0.19 | 0.0096 | 0.0414 | 2.11 |
| RAD23B | ENSG00000119318 | 190.06 | 0.22 | 0.0035 | 0.0342 | 2.34 |
| RAD50 | ENSG00000113522 | 211.06 | 0.19 | 0.0077 | 0.0384 | 2.08 |
| RAD52 | ENSG00000002016 | 58.01 | 0.22 | 0.0031 | 0.0342 | 1.27 |
| RAD9A | ENSG00000172613 | 71.31 | 0.21 | 0.0055 | 0.0363 | 1.30 |
| RAE1 | ENSG00000101146 | 41.98 | 0.22 | 0.0022 | 0.0342 | 2.80 |
| RAG1 | ENSG00000166349 | 8.81 | 0.19 | 0.0139 | 0.0469 | 2.20 |
| RAI1 | ENSG00000108557 | 187.49 | 0.19 | 0.0152 | 0.0489 | 1.75 |
| RALA | ENSG00000006451 | 87.87 | 0.21 | 0.0048 | 0.0354 | 2.36 |
| RALB | ENSG00000144118 | 114.04 | 0.23 | 0.0014 | 0.0342 | 1.55 |
| RALBP1 | ENSG00000017797 | 151.79 | 0.27 | 0.0004 | 0.0342 | 1.23 |
| RALGAPA2 | ENSG00000188559 | 573.82 | 0.24 | 0.0010 | 0.0342 | 1.69 |
| RALGPS1 | ENSG00000136828 | 182.05 | 0.23 | 0.0022 | 0.0342 | 1.10 |
| RAMP1 | ENSG00000132329 | 6.81 | 0.20 | 0.0154 | 0.0491 | 1.95 |
| RAN | ENSG00000132341 | 110.95 | 0.19 | 0.0121 | 0.0445 | 3.15 |
| RANBP10 | ENSG00000141084 | 113.15 | 0.19 | 0.0104 | 0.0424 | 1.67 |
| RANBP9 | ENSG00000010017 | 99.09 | 0.19 | 0.0095 | 0.0413 | 1.79 |
| RANGAP1 | ENSG00000100401 | 119.46 | 0.19 | 0.0135 | 0.0464 | 2.26 |
| RANGRF | ENSG00000108961 | 15.75 | 0.22 | 0.0049 | 0.0355 | 1.46 |
| RAP1A | ENSG00000116473 | 123.57 | 0.20 | 0.0058 | 0.0364 | 1.05 |
| RAP1GAP | ENSG00000076864 | 165.83 | 0.20 | 0.0102 | 0.0422 | 0.68 |
| RAP1GAP2 | ENSG00000132359 | 192.58 | 0.23 | 0.0018 | 0.0342 | 1.30 |
| RAP1GDS1 | ENSG00000138698 | 65.71 | 0.19 | 0.0092 | 0.0408 | 1.73 |
| RAP2C | ENSG00000123728 | 55.17 | 0.18 | 0.0158 | 0.0497 | 1.75 |
| RAPGEF3 | ENSG00000079337 | 92.99 | 0.19 | 0.0121 | 0.0445 | 0.88 |
| RAPGEF5 | ENSG00000136237 | 260.48 | 0.23 | 0.0018 | 0.0342 | 1.32 |
| RAPGEFL1 | ENSG00000108352 | 394.14 | 0.21 | 0.0039 | 0.0345 | 0.85 |
| RARG | ENSG00000172819 | 50.43 | 0.20 | 0.0054 | 0.0362 | 2.02 |
| RARRES3 | ENSG00000133321 | 20.44 | 0.24 | 0.0038 | 0.0342 | 1.31 |
| RARS | ENSG00000113643 | 91.89 | 0.20 | 0.0055 | 0.0363 | 2.15 |
| RARS2 | ENSG00000146282 | 64.06 | 0.19 | 0.0087 | 0.0401 | 1.81 |
| RASAL2 | ENSG00000075391 | 165.42 | 0.25 | 0.0011 | 0.0342 | 2.07 |
| RASEF | ENSG00000165105 | 234.23 | 0.20 | 0.0063 | 0.0370 | 1.07 |
| RASSF3 | ENSG00000153179 | 166.26 | 0.22 | 0.0024 | 0.0342 | 1.61 |
| RASSF6 | ENSG00000169435 | 156.81 | 0.19 | 0.0116 | 0.0439 | 0.77 |
| RASSF7 | ENSG00000099849 | 107.90 | 0.27 | 0.0004 | 0.0342 | 1.07 |
| RAVER2 | ENSG00000162437 | 207.55 | 0.23 | 0.0027 | 0.0342 | 0.83 |
| RB1 | ENSG00000139687 | 91.36 | 0.22 | 0.0052 | 0.0359 | 2.68 |
| RB1CC1 | ENSG00000023287 | 185.57 | 0.20 | 0.0070 | 0.0376 | 2.23 |
| RBBP4 | ENSG00000162521 | 147.11 | 0.18 | 0.0109 | 0.0431 | 1.79 |
| RBBP5 | ENSG00000117222 | 58.45 | 0.19 | 0.0099 | 0.0417 | 1.86 |
| RBBP9 | ENSG00000089050 | 36.68 | 0.24 | 0.0021 | 0.0342 | 1.95 |
| RBCK1 | ENSG00000125826 | 164.82 | 0.24 | 0.0010 | 0.0342 | 2.41 |
| RBFOX2 | ENSG00000100320 | 317.70 | 0.21 | 0.0044 | 0.0351 | 1.40 |
| RBL2 | ENSG00000103479 | 281.02 | 0.22 | 0.0021 | 0.0342 | 1.44 |
| RBM12 | ENSG00000244462 | 229.15 | 0.19 | 0.0139 | 0.0469 | 2.31 |
| RBM12B | ENSG00000183808 | 147.02 | 0.23 | 0.0016 | 0.0342 | 1.94 |
| RBM14 | ENSG00000239306 | 213.74 | 0.18 | 0.0115 | 0.0438 | 1.35 |
| RBM15 | ENSG00000162775 | 159.74 | 0.21 | 0.0043 | 0.0351 | 1.21 |
| RBM15B | ENSG00000179837 | 187.58 | 0.21 | 0.0043 | 0.0351 | 1.74 |
| RBM18 | ENSG00000119446 | 30.13 | 0.26 | 0.0015 | 0.0342 | 1.96 |
| RBM22 | ENSG00000086589 | 74.98 | 0.18 | 0.0152 | 0.0489 | 1.55 |
| RBM24 | ENSG00000112183 | 22.95 | 0.25 | 0.0009 | 0.0342 | 0.19 |
| RBM3 | ENSG00000102317 | 209.44 | 0.18 | 0.0151 | 0.0487 | 2.13 |
| RBM34 | ENSG00000188739 | 41.01 | 0.22 | 0.0045 | 0.0352 | 2.03 |
| RBM41 | ENSG00000089682 | 73.38 | 0.18 | 0.0158 | 0.0497 | 1.93 |
| RBM45 | ENSG00000155636 | 16.92 | 0.20 | 0.0065 | 0.0371 | 1.48 |
| RBM47 | ENSG00000163694 | 560.41 | 0.22 | 0.0028 | 0.0342 | 0.82 |
| RBM7 | ENSG00000076053 | 50.94 | 0.18 | 0.0157 | 0.0496 | 1.64 |
| RBM8A | ENSG00000131795 | 56.42 | 0.21 | 0.0043 | 0.0351 | 1.72 |
| RBMS2 | ENSG00000076067 | 251.31 | 0.22 | 0.0022 | 0.0342 | 1.39 |
| RBPJ | ENSG00000168214 | 169.66 | 0.19 | 0.0124 | 0.0450 | 1.67 |
| RC3H2 | ENSG00000056586 | 218.51 | 0.21 | 0.0051 | 0.0358 | 1.81 |
| RCBTB1 | ENSG00000136144 | 45.35 | 0.19 | 0.0104 | 0.0424 | 2.75 |
| RCC1 | ENSG00000180198 | 69.98 | 0.21 | 0.0083 | 0.0395 | 2.51 |
| RCC2 | ENSG00000179051 | 210.83 | 0.20 | 0.0070 | 0.0376 | 2.22 |
| RCCD1 | ENSG00000166965 | 24.84 | 0.19 | 0.0116 | 0.0439 | 1.82 |
| RCE1 | ENSG00000173653 | 48.76 | 0.19 | 0.0098 | 0.0416 | 1.34 |
| RCHY1 | ENSG00000163743 | 57.41 | 0.21 | 0.0055 | 0.0363 | 1.08 |
| RCN1 | ENSG00000049449 | 100.27 | 0.20 | 0.0071 | 0.0376 | 3.16 |
| RCN2 | ENSG00000117906 | 28.87 | 0.19 | 0.0102 | 0.0422 | 2.74 |
| RDH11 | ENSG00000072042 | 63.34 | 0.22 | 0.0034 | 0.0342 | 1.85 |
| RDH13 | ENSG00000160439 | 82.76 | 0.20 | 0.0064 | 0.0370 | 1.06 |
| RDH14 | ENSG00000240857 | 20.48 | 0.18 | 0.0117 | 0.0439 | 1.69 |
| RECQL | ENSG00000004700 | 38.62 | 0.19 | 0.0089 | 0.0403 | 2.12 |
| RECQL4 | ENSG00000160957 | 63.81 | 0.21 | 0.0079 | 0.0387 | 2.26 |
| REEP3 | ENSG00000165476 | 103.36 | 0.24 | 0.0009 | 0.0342 | 1.38 |
| REEP4 | ENSG00000168476 | 34.36 | 0.20 | 0.0070 | 0.0376 | 1.24 |
| REEP5 | ENSG00000129625 | 134.41 | 0.20 | 0.0053 | 0.0361 | 1.50 |
| REG4 | ENSG00000134193 | 264.31 | 0.21 | 0.0109 | 0.0431 | 1.01 |
| RELA | ENSG00000173039 | 136.40 | 0.19 | 0.0116 | 0.0439 | 1.55 |
| REM1 | ENSG00000088320 | 5.38 | 0.20 | 0.0112 | 0.0434 | 0.70 |
| RENBP | ENSG00000102032 | 9.36 | 0.23 | 0.0036 | 0.0342 | 1.37 |
| REP15 | ENSG00000174236 | 31.07 | 0.22 | 0.0043 | 0.0351 | 0.50 |
| REPIN1 | ENSG00000214022 | 315.07 | 0.24 | 0.0010 | 0.0342 | 1.91 |
| REPS2 | ENSG00000169891 | 102.11 | 0.22 | 0.0023 | 0.0342 | 1.90 |
| RER1 | ENSG00000157916 | 206.88 | 0.21 | 0.0035 | 0.0342 | 1.10 |
| RERE | ENSG00000142599 | 475.80 | 0.19 | 0.0097 | 0.0415 | 1.27 |
| REST | ENSG00000084093 | 146.61 | 0.18 | 0.0150 | 0.0486 | 1.91 |
| RETSAT | ENSG00000042445 | 235.13 | 0.23 | 0.0023 | 0.0342 | 0.76 |
| REV1 | ENSG00000135945 | 163.75 | 0.18 | 0.0144 | 0.0477 | 1.34 |
| REXO1 | ENSG00000079313 | 165.94 | 0.18 | 0.0157 | 0.0496 | 1.19 |
| REXO2 | ENSG00000076043 | 46.70 | 0.20 | 0.0067 | 0.0373 | 2.21 |
| RFNG | ENSG00000169733 | 30.25 | 0.24 | 0.0017 | 0.0342 | 1.97 |
| RFX7 | ENSG00000181827 | 102.71 | 0.19 | 0.0132 | 0.0460 | 1.59 |
| RFXANK | ENSG00000064490 | 24.71 | 0.18 | 0.0149 | 0.0485 | 2.14 |
| RG9MTD3 | ENSG00000165275 | 51.32 | 0.21 | 0.0043 | 0.0351 | 1.46 |
| RGL3 | ENSG00000205517 | 46.12 | 0.21 | 0.0057 | 0.0363 | 0.96 |
| RGMB | ENSG00000174136 | 253.15 | 0.22 | 0.0025 | 0.0342 | 1.48 |
| RGP1 | ENSG00000107185 | 268.23 | 0.21 | 0.0045 | 0.0352 | 1.02 |
| RGS12 | ENSG00000159788 | 98.75 | 0.20 | 0.0065 | 0.0371 | 1.71 |
| RGS13 | ENSG00000127074 | 12.91 | -0.31 | 0.0017 | 0.0342 | 1.16 |
| RGS3 | ENSG00000138835 | 140.86 | 0.28 | 0.0004 | 0.0342 | 1.31 |
| RHBDD3 | ENSG00000100263 | 31.64 | 0.19 | 0.0107 | 0.0428 | 1.59 |
| RHBDF1 | ENSG00000007384 | 76.36 | 0.18 | 0.0143 | 0.0476 | 2.15 |
| RHBDL2 | ENSG00000158315 | 65.09 | 0.24 | 0.0009 | 0.0342 | 0.47 |
| RHOA | ENSG00000067560 | 394.10 | 0.20 | 0.0058 | 0.0364 | 1.77 |
| RHOBTB1 | ENSG00000072422 | 43.40 | 0.18 | 0.0125 | 0.0451 | 2.53 |
| RHOC | ENSG00000155366 | 253.80 | 0.21 | 0.0054 | 0.0362 | 1.00 |
| RHOV | ENSG00000104140 | 4.32 | 0.24 | 0.0040 | 0.0346 | 2.63 |
| RIC8A | ENSG00000177963 | 115.78 | 0.18 | 0.0119 | 0.0442 | 1.72 |
| RIC8B | ENSG00000111785 | 53.86 | 0.19 | 0.0123 | 0.0448 | 1.33 |
| RILP | ENSG00000167705 | 38.82 | 0.21 | 0.0049 | 0.0355 | 0.58 |
| RIN1 | ENSG00000174791 | 26.28 | 0.22 | 0.0051 | 0.0358 | 1.79 |
| RIN2 | ENSG00000132669 | 181.75 | 0.22 | 0.0026 | 0.0342 | 1.34 |
| RIOK3 | ENSG00000101782 | 205.06 | 0.21 | 0.0061 | 0.0368 | 0.82 |
| RIPK3 | ENSG00000129465 | 45.58 | 0.18 | 0.0147 | 0.0482 | 0.90 |
| RIPK4 | ENSG00000183421 | 40.58 | 0.19 | 0.0139 | 0.0469 | 1.51 |
| RIT1 | ENSG00000143622 | 72.26 | 0.18 | 0.0155 | 0.0492 | 1.28 |
| RMND5A | ENSG00000153561 | 234.89 | 0.23 | 0.0016 | 0.0342 | 1.67 |
| RNASE4 | ENSG00000181784 | 76.38 | 0.25 | 0.0010 | 0.0342 | 1.00 |
| RNASEH1 | ENSG00000171865 | 27.05 | 0.20 | 0.0071 | 0.0376 | 2.05 |
| RNASEH2B | ENSG00000136104 | 38.90 | 0.20 | 0.0068 | 0.0374 | 2.52 |
| RNASEH2C | ENSG00000172922 | 70.08 | 0.20 | 0.0061 | 0.0368 | 1.26 |
| RNASEL | ENSG00000135828 | 78.00 | 0.25 | 0.0003 | 0.0342 | 0.82 |
| RNF10 | ENSG00000022840 | 318.29 | 0.19 | 0.0108 | 0.0429 | 1.29 |
| RNF103-VPS24 | ENSG00000249884 | 60.42 | 0.18 | 0.0161 | 0.0500 | 1.38 |
| RNF114 | ENSG00000124226 | 138.38 | 0.21 | 0.0042 | 0.0350 | 2.17 |
| RNF121 | ENSG00000137522 | 50.55 | 0.18 | 0.0134 | 0.0463 | 1.42 |
| RNF123 | ENSG00000164068 | 149.90 | 0.19 | 0.0111 | 0.0433 | 1.29 |
| RNF125 | ENSG00000101695 | 77.76 | 0.19 | 0.0082 | 0.0393 | 0.40 |
| RNF126 | ENSG00000070423 | 50.91 | 0.19 | 0.0130 | 0.0458 | 1.76 |
| RNF128 | ENSG00000133135 | 36.84 | 0.24 | 0.0008 | 0.0342 | 2.71 |
| RNF13 | ENSG00000082996 | 67.24 | 0.19 | 0.0097 | 0.0415 | 1.75 |
| RNF14 | ENSG00000013561 | 80.53 | 0.26 | 0.0003 | 0.0342 | 1.27 |
| RNF141 | ENSG00000110315 | 93.30 | 0.19 | 0.0112 | 0.0434 | 1.44 |
| RNF144A | ENSG00000151692 | 78.89 | 0.21 | 0.0057 | 0.0363 | 1.28 |
| RNF145 | ENSG00000145860 | 134.33 | 0.18 | 0.0134 | 0.0463 | 1.28 |
| RNF152 | ENSG00000176641 | 224.98 | 0.23 | 0.0018 | 0.0342 | 0.36 |
| RNF167 | ENSG00000108523 | 89.07 | 0.19 | 0.0083 | 0.0395 | 1.21 |
| RNF168 | ENSG00000163961 | 102.29 | 0.18 | 0.0148 | 0.0484 | 1.66 |
| RNF170 | ENSG00000120925 | 70.43 | 0.24 | 0.0008 | 0.0342 | 1.77 |
| RNF185 | ENSG00000138942 | 60.18 | 0.22 | 0.0036 | 0.0342 | 1.07 |
| RNF187 | ENSG00000168159 | 77.65 | 0.20 | 0.0078 | 0.0386 | 2.08 |
| RNF20 | ENSG00000155827 | 76.26 | 0.17 | 0.0153 | 0.0489 | 1.87 |
| RNF207 | ENSG00000158286 | 112.57 | 0.25 | 0.0013 | 0.0342 | 0.90 |
| RNF213 | ENSG00000173821 | 1792.43 | 0.23 | 0.0035 | 0.0342 | 1.87 |
| RNF214 | ENSG00000167257 | 60.41 | 0.21 | 0.0030 | 0.0342 | 1.59 |
| RNF215 | ENSG00000099999 | 18.33 | 0.18 | 0.0151 | 0.0487 | 1.55 |
| RNF219 | ENSG00000152193 | 22.59 | 0.24 | 0.0012 | 0.0342 | 3.43 |
| RNF220 | ENSG00000187147 | 136.53 | 0.20 | 0.0086 | 0.0399 | 1.47 |
| RNF26 | ENSG00000173456 | 43.49 | 0.19 | 0.0110 | 0.0432 | 2.00 |
| RNF31 | ENSG00000092098 | 87.86 | 0.19 | 0.0121 | 0.0445 | 1.40 |
| RNF34 | ENSG00000170633 | 59.79 | 0.18 | 0.0121 | 0.0445 | 1.42 |
| RNF38 | ENSG00000137075 | 145.61 | 0.21 | 0.0049 | 0.0355 | 1.58 |
| RNF4 | ENSG00000063978 | 142.27 | 0.19 | 0.0085 | 0.0399 | 1.46 |
| RNF40 | ENSG00000103549 | 167.61 | 0.18 | 0.0111 | 0.0433 | 1.56 |
| RNF41 | ENSG00000181852 | 62.55 | 0.20 | 0.0086 | 0.0399 | 1.67 |
| RNF43 | ENSG00000108375 | 262.76 | 0.24 | 0.0017 | 0.0342 | 6.00 |
| RNF5 | ENSG00000204308 | 45.63 | 0.21 | 0.0049 | 0.0355 | 1.24 |
| RNF7 | ENSG00000114125 | 53.34 | 0.22 | 0.0033 | 0.0342 | 1.58 |
| RNH1 | ENSG00000023191 | 136.49 | 0.19 | 0.0105 | 0.0426 | 1.55 |
| RNPC3 | ENSG00000185946 | 171.18 | 0.20 | 0.0077 | 0.0384 | 0.88 |
| RNPEP | ENSG00000176393 | 158.14 | 0.23 | 0.0021 | 0.0342 | 1.35 |
| RNPEPL1 | ENSG00000142327 | 198.72 | 0.20 | 0.0104 | 0.0424 | 1.38 |
| ROCK2 | ENSG00000134318 | 484.36 | 0.24 | 0.0018 | 0.0342 | 2.16 |
| ROD1 | ENSG00000119314 | 511.46 | 0.23 | 0.0045 | 0.0352 | 2.59 |
| ROGDI | ENSG00000067836 | 87.34 | 0.20 | 0.0078 | 0.0386 | 0.94 |
| ROS1 | ENSG00000047936 | 15.70 | 0.19 | 0.0155 | 0.0492 | 0.74 |
| RP1-130H16.18 | ENSG00000248751 | 16.84 | 0.19 | 0.0149 | 0.0485 | 1.06 |
| RP1-199H16.5 | ENSG00000184949 | 18.01 | 0.21 | 0.0056 | 0.0363 | 2.72 |
| RP11-111K18.1 | ENSG00000256646 | 42.88 | 0.21 | 0.0039 | 0.0345 | 2.98 |
| RP11-1280I22.1 | ENSG00000170476 | 103.49 | 0.20 | 0.0103 | 0.0423 | 0.36 |
| RP11-140L24.4 | ENSG00000256206 | 44.15 | 0.18 | 0.0123 | 0.0448 | 2.49 |
| RP11-146D12.2 | ENSG00000240240 | 15.24 | 0.21 | 0.0062 | 0.0368 | 0.16 |
| RP11-155D18.11 | ENSG00000114786 | 45.65 | 0.20 | 0.0071 | 0.0376 | 1.84 |
| RP11-176H8.1 | ENSG00000203546 | 161.25 | 0.20 | 0.0048 | 0.0354 | 1.16 |
| RP11-212D19.4 | ENSG00000255663 | 14.37 | 0.23 | 0.0024 | 0.0342 | 2.19 |
| RP11-234B24.6 | ENSG00000255639 | 45.19 | 0.23 | 0.0027 | 0.0342 | 1.65 |
| RP11-295K3.1 | ENSG00000250644 | 174.48 | 0.22 | 0.0036 | 0.0342 | 1.18 |
| RP11-366L20.2 | ENSG00000197301 | 44.47 | 0.18 | 0.0145 | 0.0479 | 2.13 |
| RP11-382J12.1 | ENSG00000246366 | 17.72 | 0.28 | 0.0011 | 0.0342 | 1.91 |
| RP11-392O18.1 | ENSG00000198843 | 73.78 | 0.18 | 0.0140 | 0.0471 | 1.40 |
| RP11-428C6.1 | ENSG00000214944 | 112.98 | 0.26 | 0.0005 | 0.0342 | 1.59 |
| RP11-432B6.3 | ENSG00000248710 | 36.55 | 0.21 | 0.0048 | 0.0354 | 1.65 |
| RP11-466G12.4 | ENSG00000255072 | 6.03 | 0.19 | 0.0159 | 0.0498 | 2.36 |
| RP11-468E2.1 | ENSG00000254692 | 75.63 | 0.20 | 0.0072 | 0.0377 | 1.43 |
| RP11-512M8.5 | ENSG00000256861 | 50.98 | 0.22 | 0.0022 | 0.0342 | 1.89 |
| RP11-514O12.4 | ENSG00000249141 | 27.81 | 0.17 | 0.0147 | 0.0482 | 1.95 |
| RP11-598P20.5 | ENSG00000254673 | 17.97 | 0.20 | 0.0069 | 0.0375 | 2.46 |
| RP11-691N7.6 | ENSG00000254732 | 66.47 | 0.21 | 0.0054 | 0.0362 | 1.41 |
| RP11-770G2.3 | ENSG00000254470 | 65.87 | 0.21 | 0.0052 | 0.0359 | 1.32 |
| RP11-831H9.11 | ENSG00000255432 | 12.80 | 0.22 | 0.0036 | 0.0342 | 1.84 |
| RP11-831H9.16 | ENSG00000234857 | 263.39 | 0.20 | 0.0064 | 0.0370 | 1.62 |
| RP11-867G23.8 | ENSG00000255468 | 19.25 | 0.24 | 0.0020 | 0.0342 | 1.25 |
| RP11-87C12.2 | ENSG00000256950 | 23.24 | 0.18 | 0.0156 | 0.0494 | 1.67 |
| RP11-977G19.10 | ENSG00000144785 | 39.74 | 0.24 | 0.0010 | 0.0342 | 2.05 |
| RP13-672B3.2 | ENSG00000256632 | 32.72 | 0.18 | 0.0121 | 0.0445 | 1.50 |
| RP3-402G11.5 | ENSG00000073169 | 48.79 | 0.20 | 0.0097 | 0.0415 | 1.16 |
| RP4-539M6.19 | ENSG00000249590 | 27.44 | 0.21 | 0.0034 | 0.0342 | 1.55 |
| RP4-559A3.7 | ENSG00000255835 | 74.13 | 0.23 | 0.0044 | 0.0351 | 1.15 |
| RP4-604K5.1 | ENSG00000183291 | 81.65 | 0.22 | 0.0041 | 0.0349 | 1.86 |
| RP4-811H24.6 | ENSG00000225828 | 46.15 | 0.23 | 0.0018 | 0.0342 | 0.86 |
| RP5-1180C10.2 | ENSG00000230124 | 30.42 | 0.21 | 0.0039 | 0.0345 | 1.41 |
| RP5-1187M17.10 | ENSG00000088899 | 29.69 | 0.24 | 0.0024 | 0.0342 | 5.29 |
| RP5-862P8.2 | ENSG00000143674 | 92.99 | 0.19 | 0.0110 | 0.0432 | 1.70 |
| RP5-874C20.3 | ENSG00000197062 | 23.07 | 0.19 | 0.0114 | 0.0437 | 1.32 |
| RPA1 | ENSG00000132383 | 67.07 | 0.18 | 0.0112 | 0.0434 | 1.81 |
| RPA3 | ENSG00000106399 | 14.26 | 0.19 | 0.0141 | 0.0472 | 2.22 |
| RPAP1 | ENSG00000103932 | 86.33 | 0.25 | 0.0005 | 0.0342 | 1.31 |
| RPH3AL | ENSG00000181031 | 47.40 | 0.22 | 0.0036 | 0.0342 | 1.00 |
| RPL10 | ENSG00000147403 | 279.22 | 0.18 | 0.0119 | 0.0442 | 2.23 |
| RPL10A | ENSG00000198755 | 184.31 | 0.19 | 0.0095 | 0.0413 | 2.22 |
| RPL11 | ENSG00000142676 | 487.52 | 0.20 | 0.0047 | 0.0353 | 2.20 |
| RPL12 | ENSG00000197958 | 233.33 | 0.21 | 0.0043 | 0.0351 | 2.67 |
| RPL13 | ENSG00000167526 | 418.08 | 0.18 | 0.0151 | 0.0487 | 2.35 |
| RPL13A | ENSG00000142541 | 460.86 | 0.19 | 0.0093 | 0.0409 | 2.03 |
| RPL15 | ENSG00000174748 | 405.38 | 0.19 | 0.0106 | 0.0427 | 2.13 |
| RPL18 | ENSG00000063177 | 353.99 | 0.23 | 0.0021 | 0.0342 | 2.33 |
| RPL19 | ENSG00000108298 | 459.26 | 0.20 | 0.0074 | 0.0380 | 2.23 |
| RPL23 | ENSG00000125691 | 277.57 | 0.19 | 0.0115 | 0.0438 | 2.96 |
| RPL23A | ENSG00000198242 | 132.55 | 0.19 | 0.0099 | 0.0417 | 2.49 |
| RPL24 | ENSG00000114391 | 145.23 | 0.20 | 0.0076 | 0.0382 | 2.24 |
| RPL26 | ENSG00000161970 | 216.57 | 0.20 | 0.0085 | 0.0399 | 2.53 |
| RPL27 | ENSG00000131469 | 166.00 | 0.20 | 0.0052 | 0.0359 | 2.70 |
| RPL3 | ENSG00000100316 | 806.40 | 0.20 | 0.0068 | 0.0374 | 1.97 |
| RPL34 | ENSG00000109475 | 174.01 | 0.19 | 0.0101 | 0.0421 | 2.11 |
| RPL35 | ENSG00000136942 | 143.27 | 0.19 | 0.0079 | 0.0387 | 2.76 |
| RPL36 | ENSG00000130255 | 203.93 | 0.20 | 0.0065 | 0.0371 | 2.68 |
| RPL36AL | ENSG00000165502 | 62.61 | 0.20 | 0.0069 | 0.0375 | 1.59 |
| RPL37 | ENSG00000145592 | 339.47 | 0.21 | 0.0061 | 0.0368 | 2.42 |
| RPL37A | ENSG00000197756 | 304.34 | 0.19 | 0.0086 | 0.0399 | 2.50 |
| RPL38 | ENSG00000172809 | 180.16 | 0.20 | 0.0066 | 0.0373 | 2.50 |
| RPL5 | ENSG00000122406 | 394.86 | 0.18 | 0.0120 | 0.0444 | 2.34 |
| RPL7L1 | ENSG00000146223 | 121.29 | 0.18 | 0.0140 | 0.0471 | 2.26 |
| RPL8 | ENSG00000161016 | 564.83 | 0.20 | 0.0062 | 0.0368 | 2.81 |
| RPLP0 | ENSG00000089157 | 504.16 | 0.18 | 0.0131 | 0.0460 | 2.27 |
| RPN1 | ENSG00000163902 | 245.10 | 0.20 | 0.0051 | 0.0358 | 1.76 |
| RPP21 | ENSG00000241370 | 15.77 | 0.24 | 0.0036 | 0.0342 | 1.85 |
| RPP38 | ENSG00000152464 | 21.04 | 0.18 | 0.0149 | 0.0485 | 1.46 |
| RPRD1A | ENSG00000141425 | 66.15 | 0.21 | 0.0055 | 0.0363 | 1.60 |
| RPRD1B | ENSG00000101413 | 85.11 | 0.19 | 0.0113 | 0.0435 | 2.21 |
| RPRD2 | ENSG00000163125 | 200.18 | 0.20 | 0.0058 | 0.0364 | 1.49 |
| RPS11 | ENSG00000142534 | 579.51 | 0.18 | 0.0135 | 0.0464 | 2.23 |
| RPS12 | ENSG00000112306 | 200.94 | 0.19 | 0.0099 | 0.0417 | 2.52 |
| RPS13 | ENSG00000110700 | 118.15 | 0.18 | 0.0126 | 0.0452 | 2.39 |
| RPS15 | ENSG00000115268 | 78.12 | 0.18 | 0.0160 | 0.0499 | 2.57 |
| RPS15A | ENSG00000134419 | 132.03 | 0.21 | 0.0038 | 0.0342 | 2.83 |
| RPS18 | ENSG00000231500 | 426.84 | 0.18 | 0.0136 | 0.0466 | 2.60 |
| RPS19 | ENSG00000105372 | 329.41 | 0.19 | 0.0117 | 0.0439 | 2.56 |
| RPS19BP1 | ENSG00000187051 | 43.79 | 0.18 | 0.0140 | 0.0471 | 1.47 |
| RPS20 | ENSG00000008988 | 143.94 | 0.18 | 0.0123 | 0.0448 | 2.65 |
| RPS21 | ENSG00000171858 | 98.85 | 0.18 | 0.0161 | 0.0500 | 3.89 |
| RPS24 | ENSG00000138326 | 502.33 | 0.20 | 0.0072 | 0.0377 | 2.76 |
| RPS25 | ENSG00000118181 | 103.65 | 0.18 | 0.0158 | 0.0497 | 2.39 |
| RPS27L | ENSG00000185088 | 43.20 | 0.23 | 0.0017 | 0.0342 | 1.58 |
| RPS29 | ENSG00000213741 | 96.93 | 0.19 | 0.0080 | 0.0389 | 1.97 |
| RPS3 | ENSG00000149273 | 468.43 | 0.21 | 0.0045 | 0.0352 | 2.63 |
| RPS4X | ENSG00000198034 | 518.16 | 0.23 | 0.0030 | 0.0342 | 2.92 |
| RPS5 | ENSG00000083845 | 178.71 | 0.21 | 0.0036 | 0.0342 | 2.51 |
| RPS6 | ENSG00000137154 | 854.73 | 0.21 | 0.0038 | 0.0342 | 2.60 |
| RPS6KA1 | ENSG00000117676 | 271.90 | 0.18 | 0.0136 | 0.0466 | 0.86 |
| RPS6KA3 | ENSG00000177189 | 371.29 | 0.22 | 0.0036 | 0.0342 | 1.60 |
| RPS6KL1 | ENSG00000198208 | 21.75 | 0.20 | 0.0120 | 0.0444 | 1.73 |
| RPS9 | ENSG00000170889 | 240.07 | 0.20 | 0.0057 | 0.0363 | 1.92 |
| RPTOR | ENSG00000141564 | 159.52 | 0.20 | 0.0071 | 0.0376 | 1.49 |
| RPUSD2 | ENSG00000166133 | 10.41 | 0.19 | 0.0129 | 0.0457 | 1.87 |
| RPUSD3 | ENSG00000156990 | 46.97 | 0.26 | 0.0005 | 0.0342 | 1.58 |
| RQCD1 | ENSG00000144580 | 133.36 | 0.18 | 0.0161 | 0.0500 | 1.82 |
| RRAS | ENSG00000126458 | 33.64 | 0.20 | 0.0086 | 0.0399 | 1.72 |
| RRBP1 | ENSG00000125844 | 2060.66 | 0.20 | 0.0088 | 0.0402 | 1.21 |
| RREB1 | ENSG00000124782 | 782.81 | 0.20 | 0.0062 | 0.0368 | 1.10 |
| RRN3 | ENSG00000085721 | 72.81 | 0.19 | 0.0099 | 0.0417 | 1.83 |
| RRNAD1 | ENSG00000143303 | 46.03 | 0.19 | 0.0103 | 0.0423 | 1.31 |
| RRP1B | ENSG00000160208 | 127.25 | 0.18 | 0.0137 | 0.0467 | 2.68 |
| RSBN1 | ENSG00000081019 | 92.58 | 0.24 | 0.0010 | 0.0342 | 1.25 |
| RSBN1L | ENSG00000187257 | 66.05 | 0.18 | 0.0147 | 0.0482 | 1.58 |
| RSC1A1 | ENSG00000215695 | 44.94 | 0.22 | 0.0036 | 0.0342 | 1.76 |
| RSF1 | ENSG00000048649 | 221.41 | 0.19 | 0.0081 | 0.0391 | 1.64 |
| RSL1D1 | ENSG00000171490 | 109.71 | 0.20 | 0.0094 | 0.0411 | 3.34 |
| RSL24D1 | ENSG00000137876 | 35.88 | 0.21 | 0.0063 | 0.0370 | 2.71 |
| RSPH1 | ENSG00000160188 | 9.48 | 0.29 | 0.0019 | 0.0342 | 1.40 |
| RSPH10B2 | ENSG00000169402 | 10.50 | 0.18 | 0.0102 | 0.0422 | 2.10 |
| RSPH3 | ENSG00000130363 | 41.44 | 0.21 | 0.0045 | 0.0352 | 1.08 |
| RSPRY1 | ENSG00000159579 | 104.82 | 0.20 | 0.0039 | 0.0345 | 1.55 |
| RSRC1 | ENSG00000174891 | 42.69 | 0.19 | 0.0093 | 0.0409 | 2.23 |
| RSU1 | ENSG00000148484 | 68.08 | 0.18 | 0.0120 | 0.0444 | 1.86 |
| RTEL1 | ENSG00000026036 | 230.54 | 0.22 | 0.0027 | 0.0342 | 2.36 |
| RTF1 | ENSG00000137815 | 121.90 | 0.21 | 0.0042 | 0.0350 | 1.53 |
| RTN3 | ENSG00000133318 | 162.13 | 0.21 | 0.0042 | 0.0350 | 1.78 |
| RTN4IP1 | ENSG00000130347 | 18.59 | 0.19 | 0.0158 | 0.0497 | 1.43 |
| RUFY1 | ENSG00000176783 | 106.59 | 0.19 | 0.0118 | 0.0441 | 1.27 |
| RUFY2 | ENSG00000204130 | 92.58 | 0.19 | 0.0128 | 0.0456 | 1.25 |
| RUNDC1 | ENSG00000198863 | 82.15 | 0.20 | 0.0076 | 0.0382 | 1.38 |
| RUSC1 | ENSG00000160753 | 110.33 | 0.18 | 0.0114 | 0.0437 | 1.07 |
| RWDD2A | ENSG00000013392 | 9.95 | 0.21 | 0.0075 | 0.0382 | 1.49 |
| RWDD2B | ENSG00000156253 | 16.95 | 0.23 | 0.0032 | 0.0342 | 1.51 |
| RXRA | ENSG00000186350 | 267.47 | 0.22 | 0.0048 | 0.0354 | 1.09 |
| RXRB | ENSG00000204231 | 103.32 | 0.21 | 0.0041 | 0.0349 | 1.32 |
| RYK | ENSG00000163785 | 129.89 | 0.20 | 0.0086 | 0.0399 | 1.93 |
| S100A10 | ENSG00000197747 | 341.90 | 0.18 | 0.0137 | 0.0467 | 1.42 |
| S100A11 | ENSG00000163191 | 55.52 | 0.21 | 0.0098 | 0.0416 | 4.50 |
| S100A6 | ENSG00000197956 | 351.03 | 0.21 | 0.0044 | 0.0351 | 2.28 |
| SACM1L | ENSG00000211456 | 128.29 | 0.18 | 0.0135 | 0.0464 | 1.37 |
| SAE1 | ENSG00000142230 | 80.11 | 0.20 | 0.0093 | 0.0409 | 2.42 |
| SALL1 | ENSG00000103449 | 30.52 | 0.21 | 0.0075 | 0.0382 | 0.65 |
| SAMD1 | ENSG00000141858 | 75.21 | 0.20 | 0.0070 | 0.0376 | 1.65 |
| SAMD12 | ENSG00000177570 | 171.79 | 0.21 | 0.0036 | 0.0342 | 1.47 |
| SAMD13 | ENSG00000203943 | 27.02 | 0.25 | 0.0012 | 0.0342 | 0.76 |
| SAMD14 | ENSG00000167100 | 47.97 | 0.23 | 0.0037 | 0.0342 | 1.16 |
| SAMD5 | ENSG00000203727 | 144.40 | 0.24 | 0.0054 | 0.0362 | 2.13 |
| SAMD9L | ENSG00000177409 | 162.41 | 0.20 | 0.0138 | 0.0468 | 0.91 |
| SAP18 | ENSG00000150459 | 79.55 | 0.19 | 0.0105 | 0.0426 | 2.62 |
| SAP30 | ENSG00000164105 | 6.44 | 0.18 | 0.0154 | 0.0491 | 2.63 |
| SAP30L | ENSG00000164576 | 25.25 | 0.28 | 0.0002 | 0.0342 | 1.48 |
| SARM1 | ENSG00000004139 | 132.06 | 0.23 | 0.0017 | 0.0342 | 1.33 |
| SART1 | ENSG00000175467 | 77.85 | 0.18 | 0.0151 | 0.0487 | 1.55 |
| SART3 | ENSG00000075856 | 95.35 | 0.20 | 0.0070 | 0.0376 | 1.85 |
| SAT1 | ENSG00000130066 | 417.19 | 0.21 | 0.0061 | 0.0368 | 1.51 |
| SAT2 | ENSG00000141504 | 27.22 | 0.22 | 0.0049 | 0.0355 | 1.22 |
| SATB2 | ENSG00000119042 | 645.39 | 0.20 | 0.0057 | 0.0363 | 1.06 |
| SATL1 | ENSG00000184788 | 8.42 | 0.21 | 0.0055 | 0.0363 | 1.77 |
| SBF1 | ENSG00000100241 | 310.82 | 0.19 | 0.0143 | 0.0476 | 1.60 |
| SBF2 | ENSG00000133812 | 311.43 | 0.20 | 0.0065 | 0.0371 | 1.43 |
| SBNO1 | ENSG00000139697 | 260.79 | 0.20 | 0.0068 | 0.0374 | 2.00 |
| SCAF8 | ENSG00000213079 | 301.29 | 0.18 | 0.0104 | 0.0424 | 1.30 |
| SCAMP1 | ENSG00000085365 | 75.02 | 0.21 | 0.0046 | 0.0352 | 1.83 |
| SCAMP2 | ENSG00000140497 | 266.22 | 0.21 | 0.0031 | 0.0342 | 0.89 |
| SCAMP3 | ENSG00000116521 | 39.21 | 0.18 | 0.0149 | 0.0485 | 1.93 |
| SCAMP4 | ENSG00000227500 | 100.79 | 0.21 | 0.0068 | 0.0374 | 1.54 |
| SCAP | ENSG00000114650 | 242.73 | 0.20 | 0.0064 | 0.0370 | 1.40 |
| SCARB1 | ENSG00000073060 | 113.91 | 0.24 | 0.0022 | 0.0342 | 2.26 |
| SCARB2 | ENSG00000138760 | 362.83 | 0.26 | 0.0003 | 0.0342 | 1.34 |
| SCARF1 | ENSG00000074660 | 41.66 | 0.20 | 0.0104 | 0.0424 | 0.79 |
| SCCPDH | ENSG00000143653 | 41.83 | 0.21 | 0.0036 | 0.0342 | 1.98 |
| SCD | ENSG00000099194 | 183.96 | 0.25 | 0.0013 | 0.0342 | 7.78 |
| SCFD1 | ENSG00000092108 | 74.41 | 0.21 | 0.0049 | 0.0355 | 1.39 |
| SCIN | ENSG00000006747 | 71.07 | 0.24 | 0.0018 | 0.0342 | 0.48 |
| SCNN1A | ENSG00000111319 | 550.74 | 0.24 | 0.0013 | 0.0342 | 1.03 |
| SCNN1B | ENSG00000168447 | 138.99 | 0.21 | 0.0056 | 0.0363 | 0.05 |
| SCNN1D | ENSG00000162572 | 14.23 | 0.22 | 0.0064 | 0.0370 | 0.90 |
| SCOC | ENSG00000153130 | 51.57 | 0.19 | 0.0134 | 0.0463 | 1.84 |
| SCP2 | ENSG00000116171 | 177.66 | 0.22 | 0.0021 | 0.0342 | 1.10 |
| SCRIB | ENSG00000180900 | 222.95 | 0.21 | 0.0062 | 0.0368 | 2.14 |
| SCRN2 | ENSG00000141295 | 33.11 | 0.21 | 0.0067 | 0.0373 | 1.35 |
| SCYL1 | ENSG00000142186 | 82.42 | 0.21 | 0.0061 | 0.0368 | 1.49 |
| SCYL2 | ENSG00000136021 | 149.77 | 0.18 | 0.0125 | 0.0451 | 1.27 |
| SCYL3 | ENSG00000000457 | 53.89 | 0.19 | 0.0083 | 0.0395 | 1.36 |
| SDAD1 | ENSG00000198301 | 63.75 | 0.18 | 0.0161 | 0.0500 | 2.15 |
| SDC1 | ENSG00000115884 | 340.87 | 0.26 | 0.0008 | 0.0342 | 1.59 |
| SDC4 | ENSG00000124145 | 189.62 | 0.21 | 0.0053 | 0.0361 | 2.77 |
| SDCCAG3 | ENSG00000165689 | 45.52 | 0.20 | 0.0081 | 0.0391 | 2.64 |
| SDF4 | ENSG00000078808 | 246.67 | 0.21 | 0.0029 | 0.0342 | 1.09 |
| SDHA | ENSG00000073578 | 244.06 | 0.20 | 0.0048 | 0.0354 | 0.98 |
| SDHAF1 | ENSG00000205138 | 14.33 | 0.21 | 0.0059 | 0.0365 | 1.43 |
| SDHAF2 | ENSG00000167985 | 51.92 | 0.19 | 0.0118 | 0.0441 | 1.13 |
| SDHB | ENSG00000117118 | 54.01 | 0.20 | 0.0077 | 0.0384 | 1.31 |
| SDHD | ENSG00000204370 | 61.44 | 0.22 | 0.0033 | 0.0342 | 1.12 |
| SDR39U1 | ENSG00000100445 | 100.94 | 0.21 | 0.0045 | 0.0352 | 1.15 |
| SDSL | ENSG00000139410 | 13.64 | 0.23 | 0.0034 | 0.0342 | 1.01 |
| SEC11A | ENSG00000140612 | 70.79 | 0.25 | 0.0010 | 0.0342 | 1.61 |
| SEC14L2 | ENSG00000100003 | 70.92 | 0.23 | 0.0026 | 0.0342 | 1.25 |
| SEC16A | ENSG00000148396 | 636.57 | 0.20 | 0.0064 | 0.0370 | 1.35 |
| SEC16B | ENSG00000120341 | 96.19 | 0.27 | 0.0008 | 0.0342 | 2.97 |
| SEC16B | ENSG00000254154 | 98.80 | 0.26 | 0.0012 | 0.0342 | 2.96 |
| SEC22B | ENSG00000223380 | 118.20 | 0.23 | 0.0015 | 0.0342 | 1.34 |
| SEC23IP | ENSG00000107651 | 138.41 | 0.21 | 0.0042 | 0.0350 | 1.51 |
| SEC24A | ENSG00000113615 | 178.30 | 0.18 | 0.0104 | 0.0424 | 1.18 |
| SEC24C | ENSG00000176986 | 248.33 | 0.20 | 0.0063 | 0.0370 | 1.47 |
| SEC24D | ENSG00000150961 | 205.73 | 0.19 | 0.0116 | 0.0439 | 1.09 |
| SEC31A | ENSG00000138674 | 439.70 | 0.19 | 0.0095 | 0.0413 | 1.27 |
| SEC61A1 | ENSG00000058262 | 384.82 | 0.22 | 0.0034 | 0.0342 | 1.75 |
| SEC62 | ENSG00000008952 | 167.90 | 0.20 | 0.0043 | 0.0351 | 1.37 |
| SEL1L | ENSG00000071537 | 359.51 | 0.23 | 0.0015 | 0.0342 | 1.07 |
| SEL1L3 | ENSG00000091490 | 456.85 | 0.18 | 0.0109 | 0.0431 | 1.32 |
| SELENBP1 | ENSG00000143416 | 855.43 | 0.19 | 0.0088 | 0.0402 | 0.59 |
| SEMA3C | ENSG00000075223 | 146.46 | 0.26 | 0.0004 | 0.0342 | 1.92 |
| SEMA3F | ENSG00000001617 | 47.11 | 0.20 | 0.0094 | 0.0411 | 2.11 |
| SEMA4G | ENSG00000095539 | 446.35 | 0.27 | 0.0006 | 0.0342 | 0.55 |
| SENP2 | ENSG00000163904 | 90.89 | 0.18 | 0.0106 | 0.0427 | 1.61 |
| SENP3 | ENSG00000161956 | 76.39 | 0.20 | 0.0082 | 0.0393 | 1.31 |
| SEPHS1 | ENSG00000086475 | 43.77 | 0.19 | 0.0102 | 0.0422 | 2.27 |
| SEPHS2 | ENSG00000179918 | 99.41 | 0.22 | 0.0034 | 0.0342 | 1.56 |
| SEPN1 | ENSG00000162430 | 194.39 | 0.21 | 0.0046 | 0.0352 | 1.71 |
| SEPP1 | ENSG00000250722 | 805.33 | 0.24 | 0.0022 | 0.0342 | 0.25 |
| SEPSECS | ENSG00000109618 | 62.18 | 0.22 | 0.0047 | 0.0353 | 1.25 |
| SEPT10 | ENSG00000186522 | 80.43 | 0.22 | 0.0029 | 0.0342 | 1.79 |
| SEPT11 | ENSG00000138758 | 394.09 | 0.18 | 0.0139 | 0.0469 | 1.41 |
| SEPT2 | ENSG00000168385 | 475.57 | 0.18 | 0.0138 | 0.0468 | 1.81 |
| SEPT8 | ENSG00000164402 | 188.26 | 0.23 | 0.0018 | 0.0342 | 1.76 |
| SEPW1 | ENSG00000178980 | 132.93 | 0.18 | 0.0135 | 0.0464 | 1.42 |
| SERAC1 | ENSG00000122335 | 26.88 | 0.21 | 0.0041 | 0.0349 | 1.42 |
| SERBP1 | ENSG00000142864 | 282.02 | 0.20 | 0.0091 | 0.0407 | 2.11 |
| SERF2 | ENSG00000140264 | 401.18 | 0.20 | 0.0048 | 0.0354 | 0.97 |
| SERINC1 | ENSG00000111897 | 212.54 | 0.20 | 0.0062 | 0.0368 | 1.38 |
| SERINC2 | ENSG00000168528 | 326.19 | 0.19 | 0.0089 | 0.0403 | 0.90 |
| SERINC3 | ENSG00000132824 | 285.43 | 0.20 | 0.0062 | 0.0368 | 2.44 |
| SERINC5 | ENSG00000164300 | 202.47 | 0.19 | 0.0075 | 0.0382 | 1.53 |
| SERPINA1 | ENSG00000197249 | 153.28 | 0.21 | 0.0060 | 0.0366 | 1.44 |
| SERPINA9 | ENSG00000170054 | 6.01 | -0.31 | 0.0005 | 0.0342 | 0.28 |
| SERPINB6 | ENSG00000124570 | 168.34 | 0.23 | 0.0033 | 0.0342 | 1.55 |
| SERTAD3 | ENSG00000167565 | 44.77 | 0.19 | 0.0116 | 0.0439 | 1.17 |
| SERTAD4 | ENSG00000082497 | 20.58 | 0.20 | 0.0075 | 0.0382 | 1.05 |
| SESN2 | ENSG00000130766 | 57.21 | 0.25 | 0.0008 | 0.0342 | 0.81 |
| SESTD1 | ENSG00000187231 | 109.82 | 0.23 | 0.0023 | 0.0342 | 2.27 |
| SET | ENSG00000119335 | 197.52 | 0.20 | 0.0097 | 0.0415 | 2.93 |
| SETD3 | ENSG00000183576 | 121.78 | 0.21 | 0.0029 | 0.0342 | 1.43 |
| SETD4 | ENSG00000185917 | 67.33 | 0.23 | 0.0029 | 0.0342 | 1.32 |
| SETD7 | ENSG00000145391 | 155.10 | 0.21 | 0.0040 | 0.0346 | 1.93 |
| SETDB1 | ENSG00000143379 | 133.43 | 0.21 | 0.0034 | 0.0342 | 1.53 |
| SETDB2 | ENSG00000136169 | 69.27 | 0.20 | 0.0071 | 0.0376 | 1.59 |
| SEZ6L2 | ENSG00000174938 | 32.42 | 0.23 | 0.0040 | 0.0346 | 2.26 |
| SF3A1 | ENSG00000099995 | 196.12 | 0.19 | 0.0105 | 0.0426 | 1.56 |
| SF3B2 | ENSG00000087365 | 244.24 | 0.18 | 0.0092 | 0.0408 | 1.77 |
| SF3B4 | ENSG00000143368 | 90.02 | 0.19 | 0.0132 | 0.0460 | 1.21 |
| SF3B5 | ENSG00000169976 | 30.36 | 0.24 | 0.0020 | 0.0342 | 1.85 |
| SFMBT1 | ENSG00000163935 | 78.17 | 0.21 | 0.0028 | 0.0342 | 1.52 |
| SFT2D1 | ENSG00000198818 | 52.27 | 0.22 | 0.0030 | 0.0342 | 1.46 |
| SFT2D2 | ENSG00000213064 | 52.61 | 0.20 | 0.0069 | 0.0375 | 1.54 |
| SFT2D3 | ENSG00000173349 | 24.64 | 0.20 | 0.0088 | 0.0402 | 1.85 |
| SGCB | ENSG00000163069 | 48.33 | 0.24 | 0.0016 | 0.0342 | 1.49 |
| SGK2 | ENSG00000101049 | 121.63 | 0.18 | 0.0157 | 0.0496 | 0.94 |
| SGMS1 | ENSG00000198964 | 51.37 | 0.20 | 0.0064 | 0.0370 | 1.19 |
| SGOL2 | ENSG00000163535 | 17.32 | 0.20 | 0.0127 | 0.0454 | 3.12 |
| SGPL1 | ENSG00000166224 | 216.66 | 0.22 | 0.0032 | 0.0342 | 1.52 |
| SGPP2 | ENSG00000163082 | 133.35 | 0.30 | 0.0002 | 0.0342 | 1.06 |
| SGSH | ENSG00000181523 | 89.21 | 0.24 | 0.0019 | 0.0342 | 1.17 |
| SGSM1 | ENSG00000167037 | 51.29 | 0.23 | 0.0028 | 0.0342 | 0.40 |
| SGTA | ENSG00000104969 | 67.18 | 0.18 | 0.0154 | 0.0491 | 1.86 |
| SH2B1 | ENSG00000178188 | 212.45 | 0.21 | 0.0069 | 0.0375 | 1.08 |
| SH2D3A | ENSG00000125731 | 71.84 | 0.23 | 0.0024 | 0.0342 | 1.27 |
| SH2D4A | ENSG00000104611 | 57.09 | 0.24 | 0.0024 | 0.0342 | 1.55 |
| SH3BGRL2 | ENSG00000198478 | 366.94 | 0.24 | 0.0018 | 0.0342 | 1.08 |
| SH3BGRL3 | ENSG00000142669 | 245.05 | 0.19 | 0.0099 | 0.0417 | 1.00 |
| SH3BP2 | ENSG00000087266 | 357.71 | 0.24 | 0.0009 | 0.0342 | 1.32 |
| SH3BP4 | ENSG00000130147 | 138.89 | 0.23 | 0.0014 | 0.0342 | 2.27 |
| SH3BP5L | ENSG00000175137 | 76.67 | 0.25 | 0.0011 | 0.0342 | 1.32 |
| SH3D19 | ENSG00000109686 | 558.47 | 0.25 | 0.0007 | 0.0342 | 1.30 |
| SH3D21 | ENSG00000214193 | 70.71 | 0.25 | 0.0011 | 0.0342 | 1.90 |
| SH3GLB1 | ENSG00000097033 | 253.04 | 0.21 | 0.0047 | 0.0353 | 1.27 |
| SH3GLB2 | ENSG00000148341 | 294.77 | 0.25 | 0.0011 | 0.0342 | 1.18 |
| SH3PXD2A | ENSG00000107957 | 822.11 | 0.21 | 0.0047 | 0.0353 | 1.03 |
| SH3RF1 | ENSG00000154447 | 221.79 | 0.20 | 0.0072 | 0.0377 | 0.85 |
| SH3TC2 | ENSG00000169247 | 20.34 | 0.20 | 0.0114 | 0.0437 | 7.04 |
| SH3YL1 | ENSG00000035115 | 164.86 | 0.23 | 0.0011 | 0.0342 | 1.89 |
| SHANK2 | ENSG00000162105 | 177.06 | 0.18 | 0.0127 | 0.0454 | 1.43 |
| SHARPIN | ENSG00000179526 | 61.98 | 0.22 | 0.0034 | 0.0342 | 1.80 |
| SHC1 | ENSG00000160691 | 245.47 | 0.19 | 0.0116 | 0.0439 | 1.43 |
| SHC2 | ENSG00000129946 | 17.18 | 0.24 | 0.0034 | 0.0342 | 2.25 |
| SHKBP1 | ENSG00000160410 | 68.41 | 0.19 | 0.0149 | 0.0485 | 1.98 |
| SHMT1 | ENSG00000176974 | 48.07 | 0.20 | 0.0089 | 0.0403 | 1.66 |
| SHMT2 | ENSG00000182199 | 42.25 | 0.24 | 0.0011 | 0.0342 | 3.62 |
| SHOC2 | ENSG00000108061 | 139.78 | 0.19 | 0.0099 | 0.0417 | 1.20 |
| SHPK | ENSG00000197417 | 94.42 | 0.22 | 0.0033 | 0.0342 | 0.97 |
| SHROOM1 | ENSG00000164403 | 168.53 | 0.26 | 0.0005 | 0.0342 | 1.09 |
| SHROOM3 | ENSG00000138771 | 801.32 | 0.20 | 0.0043 | 0.0351 | 0.84 |
| SI | ENSG00000090402 | 260.16 | 0.21 | 0.0054 | 0.0362 | 0.10 |
| SIAE | ENSG00000110013 | 316.22 | 0.26 | 0.0005 | 0.0342 | 0.65 |
| SIDT1 | ENSG00000072858 | 214.90 | 0.20 | 0.0051 | 0.0358 | 0.55 |
| SIGLEC11 | ENSG00000161640 | 16.02 | 0.19 | 0.0103 | 0.0423 | 1.91 |
| SIGMAR1 | ENSG00000147955 | 61.38 | 0.25 | 0.0018 | 0.0342 | 3.24 |
| SIKE1 | ENSG00000052723 | 92.32 | 0.19 | 0.0135 | 0.0464 | 1.57 |
| SIL1 | ENSG00000120725 | 57.76 | 0.20 | 0.0084 | 0.0397 | 1.65 |
| SIM2 | ENSG00000159263 | 11.84 | 0.23 | 0.0068 | 0.0374 | 9.23 |
| SIPA1L2 | ENSG00000116991 | 372.34 | 0.20 | 0.0077 | 0.0384 | 0.67 |
| SIPA1L3 | ENSG00000105738 | 547.63 | 0.22 | 0.0044 | 0.0351 | 0.95 |
| SIRT3 | ENSG00000142082 | 51.09 | 0.22 | 0.0041 | 0.0349 | 1.19 |
| SIRT5 | ENSG00000124523 | 33.51 | 0.26 | 0.0002 | 0.0342 | 1.66 |
| SIX5 | ENSG00000177045 | 32.11 | 0.22 | 0.0063 | 0.0370 | 1.41 |
| SKA3 | ENSG00000165480 | 12.50 | 0.21 | 0.0110 | 0.0432 | 5.38 |
| SKIV2L | ENSG00000204351 | 108.71 | 0.20 | 0.0067 | 0.0373 | 1.62 |
| SKP1 | ENSG00000113558 | 144.62 | 0.18 | 0.0128 | 0.0456 | 1.53 |
| SLAIN2 | ENSG00000109171 | 196.14 | 0.18 | 0.0121 | 0.0445 | 1.20 |
| SLC10A3 | ENSG00000126903 | 31.59 | 0.22 | 0.0038 | 0.0342 | 1.80 |
| SLC10A5 | ENSG00000253598 | 22.85 | 0.31 | <.0001 | 0.0342 | 0.77 |
| SLC10A7 | ENSG00000120519 | 48.76 | 0.21 | 0.0065 | 0.0371 | 1.10 |
| SLC11A2 | ENSG00000110911 | 117.71 | 0.29 | 0.0002 | 0.0342 | 2.76 |
| SLC12A2 | ENSG00000064651 | 829.56 | 0.26 | 0.0034 | 0.0342 | 3.77 |
| SLC12A7 | ENSG00000113504 | 287.29 | 0.20 | 0.0091 | 0.0407 | 1.87 |
| SLC12A8 | ENSG00000221955 | 58.35 | 0.19 | 0.0111 | 0.0433 | 1.62 |
| SLC12A9 | ENSG00000146828 | 123.09 | 0.21 | 0.0044 | 0.0351 | 2.42 |
| SLC13A2 | ENSG00000007216 | 35.31 | 0.20 | 0.0090 | 0.0405 | 0.36 |
| SLC16A14 | ENSG00000163053 | 26.14 | 0.30 | <.0001 | 0.0342 | 0.71 |
| SLC16A3 | ENSG00000141526 | 90.02 | 0.20 | 0.0107 | 0.0428 | 1.72 |
| SLC16A5 | ENSG00000170190 | 67.13 | 0.24 | 0.0020 | 0.0342 | 1.19 |
| SLC17A5 | ENSG00000119899 | 71.82 | 0.23 | 0.0015 | 0.0342 | 0.99 |
| SLC1A1 | ENSG00000106688 | 64.06 | 0.24 | 0.0032 | 0.0342 | 1.00 |
| SLC1A5 | ENSG00000105281 | 179.81 | 0.20 | 0.0046 | 0.0352 | 2.02 |
| SLC22A15 | ENSG00000163393 | 21.82 | 0.26 | 0.0017 | 0.0342 | 1.76 |
| SLC22A23 | ENSG00000137266 | 494.83 | 0.25 | 0.0010 | 0.0342 | 0.65 |
| SLC22A4 | ENSG00000197208 | 35.99 | 0.20 | 0.0059 | 0.0365 | 0.70 |
| SLC23A3 | ENSG00000213901 | 91.19 | 0.23 | 0.0026 | 0.0342 | 1.24 |
| SLC24A6 | ENSG00000089060 | 96.26 | 0.20 | 0.0092 | 0.0408 | 1.08 |
| SLC25A1 | ENSG00000100075 | 81.05 | 0.21 | 0.0045 | 0.0352 | 1.39 |
| SLC25A10 | ENSG00000183048 | 81.10 | 0.21 | 0.0057 | 0.0363 | 1.94 |
| SLC25A11 | ENSG00000108528 | 54.13 | 0.19 | 0.0094 | 0.0411 | 1.14 |
| SLC25A12 | ENSG00000115840 | 60.92 | 0.23 | 0.0022 | 0.0342 | 1.11 |
| SLC25A13 | ENSG00000004864 | 68.46 | 0.19 | 0.0074 | 0.0380 | 1.93 |
| SLC25A23 | ENSG00000125648 | 204.42 | 0.21 | 0.0052 | 0.0359 | 0.77 |
| SLC25A24 | ENSG00000085491 | 124.38 | 0.24 | 0.0014 | 0.0342 | 1.24 |
| SLC25A27 | ENSG00000153291 | 45.31 | 0.20 | 0.0096 | 0.0414 | 1.78 |
| SLC25A29 | ENSG00000197119 | 66.58 | 0.22 | 0.0058 | 0.0364 | 1.77 |
| SLC25A3 | ENSG00000075415 | 283.60 | 0.19 | 0.0088 | 0.0402 | 1.79 |
| SLC25A30 | ENSG00000174032 | 43.13 | 0.20 | 0.0071 | 0.0376 | 1.12 |
| SLC25A35 | ENSG00000125434 | 23.55 | 0.22 | 0.0048 | 0.0354 | 1.24 |
| SLC25A37 | ENSG00000147454 | 226.79 | 0.18 | 0.0131 | 0.0460 | 1.08 |
| SLC25A38 | ENSG00000144659 | 48.16 | 0.20 | 0.0081 | 0.0391 | 1.23 |
| SLC25A4 | ENSG00000151729 | 21.98 | 0.22 | 0.0052 | 0.0359 | 1.12 |
| SLC25A43 | ENSG00000077713 | 52.45 | 0.20 | 0.0055 | 0.0363 | 1.87 |
| SLC25A46 | ENSG00000164209 | 128.78 | 0.18 | 0.0143 | 0.0476 | 1.34 |
| SLC25A5 | ENSG00000005022 | 209.60 | 0.21 | 0.0048 | 0.0354 | 1.93 |
| SLC26A1 | ENSG00000145217 | 59.40 | 0.20 | 0.0064 | 0.0370 | 1.49 |
| SLC26A11 | ENSG00000181045 | 30.23 | 0.19 | 0.0161 | 0.0500 | 1.25 |
| SLC26A2 | ENSG00000155850 | 2486.27 | 0.20 | 0.0114 | 0.0437 | 0.38 |
| SLC26A6 | ENSG00000225697 | 186.94 | 0.27 | 0.0004 | 0.0342 | 1.33 |
| SLC27A1 | ENSG00000130304 | 42.08 | 0.18 | 0.0154 | 0.0491 | 1.54 |
| SLC28A3 | ENSG00000197506 | 25.37 | 0.22 | 0.0083 | 0.0395 | 1.81 |
| SLC29A1 | ENSG00000112759 | 34.44 | 0.22 | 0.0058 | 0.0364 | 4.24 |
| SLC29A2 | ENSG00000174669 | 95.55 | 0.26 | 0.0009 | 0.0342 | 1.50 |
| SLC29A3 | ENSG00000198246 | 14.11 | 0.20 | 0.0079 | 0.0387 | 2.48 |
| SLC2A10 | ENSG00000197496 | 81.60 | 0.19 | 0.0078 | 0.0386 | 1.17 |
| SLC2A8 | ENSG00000136856 | 37.69 | 0.19 | 0.0105 | 0.0426 | 2.74 |
| SLC30A1 | ENSG00000170385 | 124.21 | 0.21 | 0.0050 | 0.0356 | 1.54 |
| SLC30A4 | ENSG00000104154 | 250.41 | 0.33 | <.0001 | 0.0342 | 0.60 |
| SLC30A6 | ENSG00000152683 | 72.87 | 0.18 | 0.0154 | 0.0491 | 1.90 |
| SLC30A7 | ENSG00000162695 | 172.22 | 0.22 | 0.0035 | 0.0342 | 1.43 |
| SLC30A9 | ENSG00000014824 | 152.54 | 0.24 | 0.0017 | 0.0342 | 1.70 |
| SLC31A1 | ENSG00000136868 | 94.54 | 0.26 | 0.0011 | 0.0342 | 1.68 |
| SLC33A1 | ENSG00000169359 | 63.38 | 0.19 | 0.0097 | 0.0415 | 1.67 |
| SLC34A3 | ENSG00000198569 | 10.69 | 0.23 | 0.0062 | 0.0368 | 1.83 |
| SLC35A1 | ENSG00000164414 | 92.05 | 0.23 | 0.0015 | 0.0342 | 0.89 |
| SLC35A3 | ENSG00000117620 | 305.85 | 0.25 | 0.0009 | 0.0342 | 1.04 |
| SLC35A4 | ENSG00000176087 | 126.91 | 0.21 | 0.0028 | 0.0342 | 1.11 |
| SLC35B1 | ENSG00000121073 | 48.13 | 0.19 | 0.0105 | 0.0426 | 1.55 |
| SLC35B3 | ENSG00000124786 | 67.66 | 0.22 | 0.0027 | 0.0342 | 1.22 |
| SLC35C1 | ENSG00000181830 | 137.17 | 0.21 | 0.0034 | 0.0342 | 1.01 |
| SLC35D1 | ENSG00000116704 | 324.23 | 0.23 | 0.0020 | 0.0342 | 0.78 |
| SLC35D2 | ENSG00000130958 | 81.05 | 0.26 | 0.0003 | 0.0342 | 1.32 |
| SLC35E1 | ENSG00000127526 | 135.00 | 0.20 | 0.0084 | 0.0397 | 1.62 |
| SLC35E2 | ENSG00000215790 | 36.05 | 0.23 | 0.0025 | 0.0342 | 1.30 |
| SLC35E2B | ENSG00000189339 | 259.32 | 0.23 | 0.0024 | 0.0342 | 1.29 |
| SLC35E4 | ENSG00000100036 | 50.58 | 0.19 | 0.0105 | 0.0426 | 2.37 |
| SLC35F2 | ENSG00000110660 | 78.20 | 0.19 | 0.0094 | 0.0411 | 1.51 |
| SLC35F5 | ENSG00000115084 | 216.81 | 0.22 | 0.0023 | 0.0342 | 1.65 |
| SLC36A1 | ENSG00000123643 | 200.58 | 0.23 | 0.0034 | 0.0342 | 0.82 |
| SLC37A1 | ENSG00000160190 | 202.55 | 0.27 | 0.0003 | 0.0342 | 0.98 |
| SLC37A3 | ENSG00000157800 | 112.30 | 0.20 | 0.0055 | 0.0363 | 1.80 |
| SLC37A4 | ENSG00000137700 | 79.66 | 0.30 | <.0001 | 0.0342 | 1.42 |
| SLC38A1 | ENSG00000111371 | 599.35 | 0.21 | 0.0054 | 0.0362 | 1.98 |
| SLC38A10 | ENSG00000157637 | 342.12 | 0.21 | 0.0047 | 0.0353 | 1.31 |
| SLC39A1 | ENSG00000143570 | 92.37 | 0.21 | 0.0045 | 0.0352 | 1.96 |
| SLC39A11 | ENSG00000133195 | 78.74 | 0.22 | 0.0024 | 0.0342 | 1.45 |
| SLC39A14 | ENSG00000104635 | 328.39 | 0.25 | 0.0007 | 0.0342 | 1.30 |
| SLC39A3 | ENSG00000141873 | 21.89 | 0.20 | 0.0075 | 0.0382 | 1.65 |
| SLC39A4 | ENSG00000147804 | 38.65 | 0.23 | 0.0039 | 0.0345 | 2.56 |
| SLC39A5 | ENSG00000139540 | 158.83 | 0.20 | 0.0057 | 0.0363 | 0.91 |
| SLC39A7 | ENSG00000112473 | 182.95 | 0.23 | 0.0025 | 0.0342 | 1.19 |
| SLC39A8 | ENSG00000138821 | 162.38 | 0.23 | 0.0027 | 0.0342 | 0.82 |
| SLC39A9 | ENSG00000029364 | 198.33 | 0.22 | 0.0022 | 0.0342 | 1.48 |
| SLC3A1 | ENSG00000138079 | 58.05 | 0.21 | 0.0059 | 0.0365 | 1.52 |
| SLC3A2 | ENSG00000168003 | 93.36 | 0.18 | 0.0159 | 0.0498 | 2.70 |
| SLC40A1 | ENSG00000138449 | 314.48 | 0.33 | 0.0002 | 0.0342 | 1.38 |
| SLC41A2 | ENSG00000136052 | 107.02 | 0.24 | 0.0016 | 0.0342 | 0.79 |
| SLC44A1 | ENSG00000070214 | 598.33 | 0.24 | 0.0010 | 0.0342 | 1.17 |
| SLC44A2 | ENSG00000129353 | 210.03 | 0.20 | 0.0061 | 0.0368 | 1.18 |
| SLC44A3 | ENSG00000143036 | 83.69 | 0.25 | 0.0007 | 0.0342 | 1.59 |
| SLC44A4 | ENSG00000204385 | 654.67 | 0.23 | 0.0018 | 0.0342 | 0.61 |
| SLC45A3 | ENSG00000158715 | 76.69 | 0.19 | 0.0104 | 0.0424 | 0.88 |
| SLC45A4 | ENSG00000022567 | 89.39 | 0.20 | 0.0078 | 0.0386 | 1.90 |
| SLC46A1 | ENSG00000076351 | 136.79 | 0.22 | 0.0031 | 0.0342 | 1.31 |
| SLC46A3 | ENSG00000139508 | 64.33 | 0.18 | 0.0161 | 0.0500 | 1.05 |
| SLC48A1 | ENSG00000211584 | 52.47 | 0.23 | 0.0028 | 0.0342 | 1.46 |
| SLC4A11 | ENSG00000088836 | 4.96 | 0.20 | 0.0116 | 0.0439 | 8.05 |
| SLC4A2 | ENSG00000164889 | 109.81 | 0.22 | 0.0049 | 0.0355 | 2.76 |
| SLC4A4 | ENSG00000080493 | 664.69 | 0.20 | 0.0071 | 0.0376 | 0.06 |
| SLC50A1 | ENSG00000169241 | 52.75 | 0.19 | 0.0106 | 0.0427 | 1.08 |
| SLC5A2 | ENSG00000140675 | 50.79 | 0.21 | 0.0059 | 0.0365 | 1.48 |
| SLC5A3 | ENSG00000198743 | 310.79 | 0.22 | 0.0030 | 0.0342 | 1.58 |
| SLC5A6 | ENSG00000138074 | 67.92 | 0.19 | 0.0117 | 0.0439 | 3.55 |
| SLC6A7 | ENSG00000011083 | 101.82 | 0.23 | 0.0043 | 0.0351 | 0.43 |
| SLC6A8 | ENSG00000130821 | 225.94 | 0.26 | 0.0023 | 0.0342 | 1.00 |
| SLC7A8 | ENSG00000092068 | 78.87 | 0.19 | 0.0106 | 0.0427 | 2.03 |
| SLC9A1 | ENSG00000090020 | 227.01 | 0.21 | 0.0065 | 0.0371 | 0.64 |
| SLC9A10 | ENSG00000172139 | 31.92 | 0.20 | 0.0079 | 0.0387 | 0.76 |
| SLC9A3R2 | ENSG00000065054 | 63.51 | 0.22 | 0.0037 | 0.0342 | 1.38 |
| SLC9A6 | ENSG00000198689 | 38.89 | 0.21 | 0.0036 | 0.0342 | 1.65 |
| SLC9A8 | ENSG00000197818 | 148.91 | 0.20 | 0.0046 | 0.0352 | 1.52 |
| SLCO2B1 | ENSG00000137491 | 266.50 | 0.20 | 0.0063 | 0.0370 | 0.97 |
| SLMAP | ENSG00000163681 | 359.10 | 0.18 | 0.0129 | 0.0457 | 1.29 |
| SLMO2 | ENSG00000101166 | 111.31 | 0.18 | 0.0139 | 0.0469 | 3.04 |
| SLPI | ENSG00000124107 | 39.88 | 0.26 | 0.0009 | 0.0342 | 1.03 |
| SLU7 | ENSG00000164609 | 77.91 | 0.18 | 0.0156 | 0.0494 | 1.62 |
| SMAD1 | ENSG00000170365 | 62.96 | 0.21 | 0.0044 | 0.0351 | 1.02 |
| SMAD2 | ENSG00000175387 | 330.35 | 0.17 | 0.0160 | 0.0499 | 1.10 |
| SMAD3 | ENSG00000166949 | 219.35 | 0.20 | 0.0068 | 0.0374 | 1.21 |
| SMAD4 | ENSG00000141646 | 195.18 | 0.18 | 0.0138 | 0.0468 | 1.05 |
| SMAD5 | ENSG00000113658 | 151.00 | 0.26 | 0.0007 | 0.0342 | 1.89 |
| SMAD6 | ENSG00000137834 | 28.34 | 0.21 | 0.0073 | 0.0379 | 2.03 |
| SMAGP | ENSG00000170545 | 64.07 | 0.21 | 0.0045 | 0.0352 | 1.51 |
| SMAP1 | ENSG00000112305 | 93.50 | 0.18 | 0.0127 | 0.0454 | 1.32 |
| SMARCA2 | ENSG00000080503 | 249.98 | 0.21 | 0.0038 | 0.0342 | 1.27 |
| SMARCA4 | ENSG00000127616 | 304.35 | 0.18 | 0.0147 | 0.0482 | 2.03 |
| SMARCA5 | ENSG00000153147 | 214.23 | 0.19 | 0.0088 | 0.0402 | 1.94 |
| SMARCB1 | ENSG00000099956 | 60.78 | 0.18 | 0.0117 | 0.0439 | 1.61 |
| SMARCC2 | ENSG00000139613 | 343.78 | 0.20 | 0.0044 | 0.0351 | 1.43 |
| SMARCD1 | ENSG00000066117 | 98.14 | 0.21 | 0.0031 | 0.0342 | 1.88 |
| SMARCD2 | ENSG00000108604 | 108.58 | 0.19 | 0.0095 | 0.0413 | 2.31 |
| SMARCD3 | ENSG00000082014 | 35.13 | 0.20 | 0.0060 | 0.0366 | 1.17 |
| SMARCE1 | ENSG00000073584 | 158.50 | 0.19 | 0.0091 | 0.0407 | 1.73 |
| SMC1A | ENSG00000072501 | 250.33 | 0.19 | 0.0104 | 0.0424 | 2.06 |
| SMC6 | ENSG00000163029 | 122.57 | 0.19 | 0.0123 | 0.0448 | 1.68 |
| SMCR7 | ENSG00000177427 | 19.93 | 0.25 | 0.0011 | 0.0342 | 0.98 |
| SMCR7L | ENSG00000100335 | 111.70 | 0.22 | 0.0032 | 0.0342 | 1.69 |
| SMCR8 | ENSG00000176994 | 83.59 | 0.21 | 0.0055 | 0.0363 | 1.18 |
| SMEK2 | ENSG00000138041 | 247.02 | 0.24 | 0.0014 | 0.0342 | 1.78 |
| SMG1 | ENSG00000157106 | 951.17 | 0.22 | 0.0019 | 0.0342 | 1.64 |
| SMG5 | ENSG00000198952 | 259.22 | 0.23 | 0.0030 | 0.0342 | 1.80 |
| SMG6 | ENSG00000070366 | 214.59 | 0.19 | 0.0133 | 0.0462 | 0.89 |
| SMG7 | ENSG00000116698 | 266.45 | 0.20 | 0.0059 | 0.0365 | 2.15 |
| SMG8 | ENSG00000167447 | 17.89 | 0.21 | 0.0050 | 0.0356 | 2.20 |
| SMOC2 | ENSG00000112562 | 63.88 | 0.21 | 0.0059 | 0.0365 | 2.53 |
| SMPD2 | ENSG00000135587 | 33.07 | 0.19 | 0.0151 | 0.0487 | 1.25 |
| SMPD3 | ENSG00000103056 | 259.76 | 0.26 | 0.0004 | 0.0342 | 0.54 |
| SMPD4 | ENSG00000136699 | 131.63 | 0.19 | 0.0132 | 0.0460 | 2.07 |
| SMPDL3A | ENSG00000172594 | 60.24 | 0.21 | 0.0046 | 0.0352 | 0.43 |
| SMPDL3B | ENSG00000130768 | 32.41 | 0.21 | 0.0042 | 0.0350 | 0.85 |
| SMS | ENSG00000102172 | 54.97 | 0.20 | 0.0087 | 0.0401 | 2.82 |
| SMUG1 | ENSG00000123415 | 41.05 | 0.23 | 0.0017 | 0.0342 | 1.39 |
| SMYD2 | ENSG00000143499 | 71.12 | 0.19 | 0.0105 | 0.0426 | 2.08 |
| SNAP23 | ENSG00000092531 | 99.83 | 0.22 | 0.0042 | 0.0350 | 1.23 |
| SNAP29 | ENSG00000099940 | 69.70 | 0.24 | 0.0009 | 0.0342 | 1.29 |
| SNAP47 | ENSG00000143740 | 56.10 | 0.25 | 0.0003 | 0.0342 | 1.62 |
| SNAPC2 | ENSG00000104976 | 11.16 | 0.18 | 0.0161 | 0.0500 | 1.76 |
| SNAPC3 | ENSG00000164975 | 177.33 | 0.24 | 0.0025 | 0.0342 | 1.34 |
| SNAPC4 | ENSG00000165684 | 55.74 | 0.19 | 0.0134 | 0.0463 | 2.16 |
| SNAPIN | ENSG00000143553 | 19.97 | 0.25 | 0.0005 | 0.0342 | 1.49 |
| SNRNP200 | ENSG00000144028 | 465.78 | 0.19 | 0.0089 | 0.0403 | 2.06 |
| SNRNP35 | ENSG00000184209 | 44.51 | 0.22 | 0.0030 | 0.0342 | 1.30 |
| SNRNP48 | ENSG00000168566 | 46.23 | 0.24 | 0.0014 | 0.0342 | 1.93 |
| SNRPA | ENSG00000077312 | 45.58 | 0.20 | 0.0050 | 0.0356 | 2.00 |
| SNRPB2 | ENSG00000125870 | 20.54 | 0.18 | 0.0138 | 0.0468 | 3.39 |
| SNRPD2 | ENSG00000125743 | 26.56 | 0.20 | 0.0089 | 0.0403 | 3.10 |
| SNRPF | ENSG00000139343 | 7.67 | 0.23 | 0.0043 | 0.0351 | 3.48 |
| SNTB1 | ENSG00000172164 | 51.90 | 0.21 | 0.0067 | 0.0373 | 5.36 |
| SNTB2 | ENSG00000168807 | 174.77 | 0.21 | 0.0044 | 0.0351 | 1.73 |
| SNUPN | ENSG00000169371 | 20.22 | 0.21 | 0.0056 | 0.0363 | 1.45 |
| SNW1 | ENSG00000100603 | 66.78 | 0.20 | 0.0057 | 0.0363 | 1.79 |
| SNX1 | ENSG00000028528 | 268.14 | 0.17 | 0.0153 | 0.0489 | 1.13 |
| SNX12 | ENSG00000147164 | 61.43 | 0.19 | 0.0100 | 0.0419 | 2.00 |
| SNX14 | ENSG00000135317 | 130.92 | 0.18 | 0.0129 | 0.0457 | 1.58 |
| SNX15 | ENSG00000110025 | 57.16 | 0.23 | 0.0017 | 0.0342 | 1.34 |
| SNX16 | ENSG00000104497 | 16.90 | 0.19 | 0.0123 | 0.0448 | 1.66 |
| SNX19 | ENSG00000120451 | 321.41 | 0.19 | 0.0088 | 0.0402 | 1.19 |
| SNX2 | ENSG00000205302 | 63.62 | 0.18 | 0.0138 | 0.0468 | 1.43 |
| SNX21 | ENSG00000124104 | 82.16 | 0.21 | 0.0048 | 0.0354 | 1.60 |
| SNX24 | ENSG00000064652 | 39.22 | 0.21 | 0.0038 | 0.0342 | 0.77 |
| SNX27 | ENSG00000143376 | 159.79 | 0.19 | 0.0071 | 0.0376 | 1.72 |
| SNX3 | ENSG00000112335 | 67.53 | 0.19 | 0.0081 | 0.0391 | 1.84 |
| SNX33 | ENSG00000173548 | 209.35 | 0.20 | 0.0086 | 0.0399 | 1.23 |
| SNX4 | ENSG00000114520 | 49.16 | 0.26 | 0.0003 | 0.0342 | 1.53 |
| SNX6 | ENSG00000129515 | 80.47 | 0.20 | 0.0077 | 0.0384 | 1.40 |
| SNX7 | ENSG00000162627 | 44.94 | 0.18 | 0.0132 | 0.0460 | 1.64 |
| SNX8 | ENSG00000106266 | 27.15 | 0.18 | 0.0160 | 0.0499 | 1.71 |
| SOAT1 | ENSG00000057252 | 95.77 | 0.19 | 0.0112 | 0.0434 | 1.12 |
| SOCS6 | ENSG00000170677 | 116.24 | 0.21 | 0.0075 | 0.0382 | 0.90 |
| SOD1 | ENSG00000142168 | 71.79 | 0.20 | 0.0071 | 0.0376 | 2.06 |
| SORD | ENSG00000140263 | 24.12 | 0.23 | 0.0043 | 0.0351 | 3.80 |
| SORL1 | ENSG00000137642 | 731.61 | 0.28 | <.0001 | 0.0342 | 1.58 |
| SORT1 | ENSG00000134243 | 407.03 | 0.25 | 0.0008 | 0.0342 | 1.46 |
| SOS2 | ENSG00000100485 | 209.92 | 0.18 | 0.0132 | 0.0460 | 0.99 |
| SOSTDC1 | ENSG00000171243 | 19.07 | 0.21 | 0.0051 | 0.0358 | 0.46 |
| SOX13 | ENSG00000143842 | 92.22 | 0.25 | 0.0004 | 0.0342 | 1.64 |
| SOX4 | ENSG00000124766 | 121.44 | 0.29 | 0.0003 | 0.0342 | 4.89 |
| SOX9 | ENSG00000125398 | 186.11 | 0.25 | 0.0071 | 0.0376 | 3.46 |
| SP1 | ENSG00000185591 | 474.45 | 0.24 | 0.0013 | 0.0342 | 1.51 |
| SP140L | ENSG00000185404 | 80.28 | 0.23 | 0.0017 | 0.0342 | 0.98 |
| SP3 | ENSG00000172845 | 256.89 | 0.19 | 0.0086 | 0.0399 | 1.71 |
| SP5 | ENSG00000204335 | 3.75 | 0.24 | 0.0069 | 0.0375 | 5.85 |
| SP6 | ENSG00000189120 | 11.36 | 0.24 | 0.0057 | 0.0363 | 5.62 |
| SPACA4 | ENSG00000177202 | 30.15 | 0.28 | 0.0002 | 0.0342 | 0.67 |
| SPAG1 | ENSG00000104450 | 87.87 | 0.20 | 0.0053 | 0.0361 | 1.81 |
| SPAG7 | ENSG00000091640 | 30.55 | 0.20 | 0.0061 | 0.0368 | 1.14 |
| SPATA12 | ENSG00000186451 | 11.17 | 0.19 | 0.0110 | 0.0432 | 2.54 |
| SPATA13 | ENSG00000182957 | 286.25 | 0.22 | 0.0027 | 0.0342 | 2.60 |
| SPATA18 | ENSG00000163071 | 34.20 | 0.21 | 0.0044 | 0.0351 | 0.93 |
| SPATA20 | ENSG00000006282 | 122.40 | 0.19 | 0.0086 | 0.0399 | 1.05 |
| SPATA24 | ENSG00000170469 | 32.25 | 0.26 | 0.0005 | 0.0342 | 0.64 |
| SPATA5L1 | ENSG00000171763 | 20.42 | 0.21 | 0.0048 | 0.0354 | 1.70 |
| SPATA7 | ENSG00000042317 | 12.07 | 0.18 | 0.0150 | 0.0486 | 1.55 |
| SPATS2L | ENSG00000196141 | 219.10 | 0.19 | 0.0117 | 0.0439 | 1.34 |
| SPCS1 | ENSG00000114902 | 47.22 | 0.19 | 0.0094 | 0.0411 | 1.66 |
| SPCS2 | ENSG00000118363 | 43.23 | 0.18 | 0.0137 | 0.0467 | 1.30 |
| SPDYE3 | ENSG00000214300 | 42.30 | 0.19 | 0.0125 | 0.0451 | 1.55 |
| SPDYE6 | ENSG00000173678 | 71.56 | 0.19 | 0.0129 | 0.0457 | 1.56 |
| SPECC1 | ENSG00000128487 | 265.33 | 0.21 | 0.0045 | 0.0352 | 0.63 |
| SPECC1L | ENSG00000100014 | 299.36 | 0.21 | 0.0032 | 0.0342 | 0.87 |
| SPG11 | ENSG00000104133 | 425.47 | 0.22 | 0.0014 | 0.0342 | 1.14 |
| SPG21 | ENSG00000090487 | 79.34 | 0.19 | 0.0086 | 0.0399 | 2.00 |
| SPG7 | ENSG00000197912 | 233.45 | 0.20 | 0.0057 | 0.0363 | 1.16 |
| SPHK2 | ENSG00000063176 | 54.49 | 0.20 | 0.0108 | 0.0429 | 1.45 |
| SPICE1 | ENSG00000163611 | 151.87 | 0.24 | 0.0015 | 0.0342 | 1.30 |
| SPIN1 | ENSG00000106723 | 172.18 | 0.23 | 0.0022 | 0.0342 | 1.75 |
| SPIN2A | ENSG00000147059 | 18.19 | 0.19 | 0.0097 | 0.0415 | 1.89 |
| SPIN4 | ENSG00000186767 | 21.32 | 0.22 | 0.0047 | 0.0353 | 2.61 |
| SPINK5 | ENSG00000133710 | 57.15 | 0.19 | 0.0126 | 0.0452 | 0.39 |
| SPINT1 | ENSG00000166145 | 284.27 | 0.22 | 0.0025 | 0.0342 | 1.05 |
| SPINT2 | ENSG00000167642 | 244.27 | 0.21 | 0.0034 | 0.0342 | 1.53 |
| SPIRE1 | ENSG00000134278 | 36.57 | 0.24 | 0.0020 | 0.0342 | 2.25 |
| SPON1 | ENSG00000152268 | 286.07 | 0.18 | 0.0135 | 0.0464 | 0.42 |
| SPON2 | ENSG00000159674 | 101.54 | 0.22 | 0.0035 | 0.0342 | 2.19 |
| SPOPL | ENSG00000144228 | 105.49 | 0.20 | 0.0071 | 0.0376 | 1.53 |
| SPPL3 | ENSG00000157837 | 136.63 | 0.21 | 0.0040 | 0.0346 | 1.58 |
| SPR | ENSG00000116096 | 41.06 | 0.26 | 0.0005 | 0.0342 | 1.67 |
| SPRED1 | ENSG00000166068 | 144.39 | 0.20 | 0.0054 | 0.0362 | 1.56 |
| SPRED2 | ENSG00000198369 | 166.78 | 0.19 | 0.0101 | 0.0421 | 1.31 |
| SPRY4 | ENSG00000187678 | 118.22 | 0.19 | 0.0112 | 0.0434 | 2.23 |
| SPRYD3 | ENSG00000167778 | 69.36 | 0.22 | 0.0029 | 0.0342 | 1.57 |
| SPRYD7 | ENSG00000123178 | 21.96 | 0.23 | 0.0027 | 0.0342 | 1.96 |
| SPSB3 | ENSG00000162032 | 85.58 | 0.20 | 0.0111 | 0.0433 | 1.18 |
| SPTAN1 | ENSG00000197694 | 907.54 | 0.20 | 0.0064 | 0.0370 | 1.66 |
| SPTB | ENSG00000070182 | 68.36 | 0.23 | 0.0022 | 0.0342 | 1.23 |
| SPTBN1 | ENSG00000115306 | 2676.58 | 0.21 | 0.0030 | 0.0342 | 1.42 |
| SPTBN2 | ENSG00000173898 | 38.78 | 0.22 | 0.0047 | 0.0353 | 5.89 |
| SPTLC1 | ENSG00000090054 | 85.62 | 0.22 | 0.0042 | 0.0350 | 1.82 |
| SPTLC2 | ENSG00000100596 | 255.24 | 0.25 | 0.0004 | 0.0342 | 1.44 |
| SPTLC3 | ENSG00000172296 | 38.40 | 0.18 | 0.0161 | 0.0500 | 0.63 |
| SPTSSA | ENSG00000165389 | 54.54 | 0.27 | 0.0004 | 0.0342 | 1.31 |
| SRC | ENSG00000197122 | 305.61 | 0.25 | 0.0007 | 0.0342 | 1.48 |
| SRCAP | ENSG00000080603 | 706.31 | 0.19 | 0.0103 | 0.0423 | 1.53 |
| SRD5A3 | ENSG00000128039 | 23.90 | 0.25 | 0.0017 | 0.0342 | 1.72 |
| SREBF1 | ENSG00000072310 | 200.74 | 0.19 | 0.0112 | 0.0434 | 1.47 |
| SREBF2 | ENSG00000198911 | 463.94 | 0.21 | 0.0043 | 0.0351 | 1.32 |
| SREK1IP1 | ENSG00000153006 | 51.32 | 0.18 | 0.0156 | 0.0494 | 1.64 |
| SRGAP1 | ENSG00000196935 | 194.71 | 0.23 | 0.0017 | 0.0342 | 1.01 |
| SRGAP3 | ENSG00000196220 | 68.39 | 0.18 | 0.0148 | 0.0484 | 1.27 |
| SRMS | ENSG00000125508 | 4.49 | 0.21 | 0.0100 | 0.0419 | 5.45 |
| SRP68 | ENSG00000167881 | 74.59 | 0.20 | 0.0058 | 0.0364 | 1.86 |
| SRP72 | ENSG00000174780 | 104.47 | 0.22 | 0.0046 | 0.0352 | 2.08 |
| SRP9 | ENSG00000143742 | 96.64 | 0.24 | 0.0025 | 0.0342 | 2.27 |
| SRPK1 | ENSG00000096063 | 271.55 | 0.22 | 0.0041 | 0.0349 | 2.83 |
| SRPR | ENSG00000182934 | 249.35 | 0.19 | 0.0116 | 0.0439 | 1.25 |
| SRPRB | ENSG00000144867 | 56.78 | 0.20 | 0.0068 | 0.0374 | 1.99 |
| SRRM2 | ENSG00000167978 | 5166.21 | 0.20 | 0.0063 | 0.0370 | 1.01 |
| SRSF1 | ENSG00000136450 | 504.62 | 0.18 | 0.0157 | 0.0496 | 1.81 |
| SRSF9 | ENSG00000111786 | 81.78 | 0.19 | 0.0084 | 0.0397 | 2.00 |
| SS18 | ENSG00000141380 | 138.05 | 0.18 | 0.0130 | 0.0458 | 1.24 |
| SS18L1 | ENSG00000184402 | 58.84 | 0.20 | 0.0057 | 0.0363 | 2.34 |
| SSBP3 | ENSG00000157216 | 91.84 | 0.20 | 0.0051 | 0.0358 | 1.26 |
| SSBP4 | ENSG00000130511 | 57.68 | 0.19 | 0.0105 | 0.0426 | 2.05 |
| SSH3 | ENSG00000172830 | 93.33 | 0.22 | 0.0035 | 0.0342 | 1.32 |
| SSNA1 | ENSG00000176101 | 28.25 | 0.23 | 0.0030 | 0.0342 | 1.78 |
| SSR1 | ENSG00000124783 | 358.85 | 0.20 | 0.0067 | 0.0373 | 1.85 |
| SSR2 | ENSG00000163479 | 169.83 | 0.19 | 0.0072 | 0.0377 | 1.67 |
| SSR3 | ENSG00000114850 | 213.78 | 0.24 | 0.0027 | 0.0342 | 1.75 |
| SSTR1 | ENSG00000139874 | 17.09 | 0.26 | 0.0006 | 0.0342 | 0.66 |
| SSU72 | ENSG00000160075 | 141.49 | 0.19 | 0.0099 | 0.0417 | 1.48 |
| ST13 | ENSG00000100380 | 85.86 | 0.20 | 0.0054 | 0.0362 | 2.22 |
| ST14 | ENSG00000149418 | 746.23 | 0.21 | 0.0034 | 0.0342 | 1.26 |
| ST3GAL6 | ENSG00000064225 | 20.73 | 0.21 | 0.0057 | 0.0363 | 1.20 |
| ST6GALNAC1 | ENSG00000070526 | 533.90 | 0.21 | 0.0050 | 0.0356 | 0.45 |
| ST6GALNAC6 | ENSG00000160408 | 685.17 | 0.19 | 0.0091 | 0.0407 | 0.14 |
| STAG1 | ENSG00000118007 | 191.37 | 0.18 | 0.0105 | 0.0426 | 1.33 |
| STAM2 | ENSG00000115145 | 123.11 | 0.22 | 0.0020 | 0.0342 | 1.15 |
| STAMBP | ENSG00000124356 | 105.27 | 0.22 | 0.0030 | 0.0342 | 1.51 |
| STAP2 | ENSG00000178078 | 101.90 | 0.21 | 0.0038 | 0.0342 | 0.81 |
| STARD10 | ENSG00000214530 | 250.91 | 0.24 | 0.0015 | 0.0342 | 0.78 |
| STARD3NL | ENSG00000010270 | 43.44 | 0.19 | 0.0106 | 0.0427 | 1.90 |
| STARD4 | ENSG00000164211 | 101.79 | 0.19 | 0.0152 | 0.0489 | 1.31 |
| STARD5 | ENSG00000172345 | 97.86 | 0.18 | 0.0136 | 0.0466 | 0.64 |
| STARD7 | ENSG00000084090 | 289.79 | 0.21 | 0.0040 | 0.0346 | 1.86 |
| STAT1 | ENSG00000115415 | 373.42 | 0.20 | 0.0155 | 0.0492 | 2.09 |
| STAT2 | ENSG00000170581 | 285.55 | 0.19 | 0.0118 | 0.0441 | 1.40 |
| STAT5A | ENSG00000126561 | 69.82 | 0.19 | 0.0112 | 0.0434 | 1.49 |
| STAT6 | ENSG00000166888 | 492.41 | 0.22 | 0.0026 | 0.0342 | 1.37 |
| STAU1 | ENSG00000124214 | 197.14 | 0.21 | 0.0053 | 0.0361 | 2.80 |
| STAU2 | ENSG00000040341 | 102.27 | 0.23 | 0.0023 | 0.0342 | 1.70 |
| STBD1 | ENSG00000118804 | 46.13 | 0.22 | 0.0035 | 0.0342 | 0.94 |
| STEAP2 | ENSG00000157214 | 118.05 | 0.19 | 0.0103 | 0.0423 | 1.69 |
| STIL | ENSG00000123473 | 59.99 | 0.20 | 0.0103 | 0.0423 | 2.22 |
| STIM2 | ENSG00000109689 | 90.96 | 0.20 | 0.0081 | 0.0391 | 1.17 |
| STK11 | ENSG00000118046 | 110.74 | 0.22 | 0.0045 | 0.0352 | 1.62 |
| STK24 | ENSG00000102572 | 382.49 | 0.20 | 0.0075 | 0.0382 | 1.75 |
| STK25 | ENSG00000115694 | 207.55 | 0.22 | 0.0037 | 0.0342 | 1.47 |
| STK36 | ENSG00000163482 | 72.16 | 0.25 | 0.0006 | 0.0342 | 2.24 |
| STK38 | ENSG00000112079 | 243.09 | 0.20 | 0.0072 | 0.0377 | 1.67 |
| STK38L | ENSG00000211455 | 129.90 | 0.18 | 0.0160 | 0.0499 | 2.42 |
| STK39 | ENSG00000198648 | 143.50 | 0.26 | 0.0004 | 0.0342 | 1.60 |
| STOML1 | ENSG00000067221 | 12.40 | 0.24 | 0.0020 | 0.0342 | 1.69 |
| STOML2 | ENSG00000165283 | 51.72 | 0.21 | 0.0046 | 0.0352 | 2.27 |
| STON2 | ENSG00000140022 | 44.10 | 0.20 | 0.0069 | 0.0375 | 0.99 |
| STRA13 | ENSG00000169689 | 28.26 | 0.19 | 0.0112 | 0.0434 | 1.81 |
| STRADB | ENSG00000082146 | 59.94 | 0.24 | 0.0014 | 0.0342 | 0.95 |
| STRN | ENSG00000115808 | 210.67 | 0.26 | 0.0007 | 0.0342 | 1.76 |
| STS | ENSG00000101846 | 82.33 | 0.23 | 0.0044 | 0.0351 | 0.92 |
| STT3B | ENSG00000163527 | 261.17 | 0.20 | 0.0078 | 0.0386 | 2.66 |
| STUB1 | ENSG00000103266 | 62.53 | 0.20 | 0.0064 | 0.0370 | 1.78 |
| STX10 | ENSG00000104915 | 41.53 | 0.26 | 0.0004 | 0.0342 | 1.82 |
| STX12 | ENSG00000117758 | 103.84 | 0.21 | 0.0048 | 0.0354 | 1.01 |
| STX16 | ENSG00000124222 | 341.50 | 0.19 | 0.0074 | 0.0380 | 2.05 |
| STX18 | ENSG00000168818 | 86.16 | 0.20 | 0.0067 | 0.0373 | 1.29 |
| STX3 | ENSG00000166900 | 207.49 | 0.21 | 0.0044 | 0.0351 | 1.47 |
| STX5 | ENSG00000162236 | 65.55 | 0.22 | 0.0026 | 0.0342 | 1.13 |
| STX6 | ENSG00000135823 | 96.11 | 0.19 | 0.0085 | 0.0399 | 1.97 |
| STXBP2 | ENSG00000076944 | 128.35 | 0.19 | 0.0088 | 0.0402 | 1.20 |
| STXBP5 | ENSG00000164506 | 280.74 | 0.19 | 0.0098 | 0.0416 | 1.03 |
| STXBP6 | ENSG00000168952 | 47.93 | 0.21 | 0.0054 | 0.0362 | 1.44 |
| STYK1 | ENSG00000060140 | 81.17 | 0.26 | 0.0004 | 0.0342 | 0.42 |
| SUCLA2 | ENSG00000136143 | 44.69 | 0.23 | 0.0017 | 0.0342 | 2.25 |
| SUCLG2 | ENSG00000172340 | 154.70 | 0.19 | 0.0091 | 0.0407 | 0.96 |
| SUDS3 | ENSG00000111707 | 190.46 | 0.19 | 0.0076 | 0.0382 | 1.60 |
| SUGP1 | ENSG00000105705 | 36.79 | 0.21 | 0.0064 | 0.0370 | 1.63 |
| SUGT1 | ENSG00000165416 | 32.97 | 0.28 | 0.0005 | 0.0342 | 3.02 |
| SULF1 | ENSG00000137573 | 153.08 | 0.25 | 0.0022 | 0.0342 | 6.87 |
| SULF2 | ENSG00000196562 | 249.40 | 0.21 | 0.0039 | 0.0345 | 2.08 |
| SULT1A1 | ENSG00000196502 | 39.00 | 0.20 | 0.0103 | 0.0423 | 0.87 |
| SULT1B1 | ENSG00000173597 | 94.80 | 0.24 | 0.0014 | 0.0342 | 0.39 |
| SULT1C2 | ENSG00000198203 | 78.87 | 0.34 | 0.0022 | 0.0342 | 3.21 |
| SULT1C3 | ENSG00000196228 | 5.42 | 0.23 | 0.0078 | 0.0386 | 6.06 |
| SUMF2 | ENSG00000129103 | 156.70 | 0.20 | 0.0086 | 0.0399 | 1.91 |
| SUMO1 | ENSG00000116030 | 48.84 | 0.19 | 0.0097 | 0.0415 | 2.36 |
| SUMO2 | ENSG00000188612 | 43.61 | 0.20 | 0.0047 | 0.0353 | 2.33 |
| SUMO3 | ENSG00000184900 | 84.30 | 0.22 | 0.0028 | 0.0342 | 1.80 |
| SUN1 | ENSG00000164828 | 409.95 | 0.22 | 0.0024 | 0.0342 | 1.72 |
| SUOX | ENSG00000139531 | 65.12 | 0.21 | 0.0052 | 0.0359 | 1.40 |
| SUPT16H | ENSG00000092201 | 141.94 | 0.20 | 0.0062 | 0.0368 | 2.26 |
| SUPT4H1 | ENSG00000213246 | 61.09 | 0.22 | 0.0071 | 0.0376 | 2.65 |
| SUPT5H | ENSG00000196235 | 202.49 | 0.19 | 0.0111 | 0.0433 | 1.48 |
| SURF1 | ENSG00000148290 | 28.01 | 0.18 | 0.0089 | 0.0403 | 1.39 |
| SURF4 | ENSG00000148248 | 333.55 | 0.21 | 0.0041 | 0.0349 | 1.62 |
| SURF6 | ENSG00000148296 | 59.00 | 0.20 | 0.0063 | 0.0370 | 1.81 |
| SUSD1 | ENSG00000106868 | 64.31 | 0.20 | 0.0076 | 0.0382 | 1.97 |
| SUV420H1 | ENSG00000110066 | 190.72 | 0.24 | 0.0014 | 0.0342 | 1.17 |
| SUV420H2 | ENSG00000133247 | 45.10 | 0.25 | 0.0020 | 0.0342 | 1.47 |
| SYAP1 | ENSG00000169895 | 160.09 | 0.19 | 0.0130 | 0.0458 | 1.86 |
| SYBU | ENSG00000147642 | 50.02 | 0.29 | 0.0033 | 0.0342 | 2.27 |
| SYDE2 | ENSG00000097096 | 34.99 | 0.20 | 0.0074 | 0.0380 | 1.87 |
| SYF2 | ENSG00000117614 | 46.11 | 0.19 | 0.0099 | 0.0417 | 1.47 |
| SYNGR2 | ENSG00000108639 | 165.63 | 0.21 | 0.0036 | 0.0342 | 1.84 |
| SYNJ2 | ENSG00000078269 | 211.55 | 0.20 | 0.0047 | 0.0353 | 1.66 |
| SYNJ2BP | ENSG00000213463 | 263.44 | 0.23 | 0.0020 | 0.0342 | 1.03 |
| SYS1-DBNDD2 | ENSG00000254806 | 70.34 | 0.20 | 0.0056 | 0.0363 | 1.83 |
| SYT10 | ENSG00000110975 | 10.24 | 0.21 | 0.0060 | 0.0366 | 0.18 |
| SYT13 | ENSG00000019505 | 101.54 | 0.19 | 0.0071 | 0.0376 | 1.07 |
| SYT7 | ENSG00000011347 | 81.70 | 0.21 | 0.0041 | 0.0349 | 2.61 |
| SYTL2 | ENSG00000137501 | 1083.34 | 0.26 | 0.0007 | 0.0342 | 0.50 |
| SYTL4 | ENSG00000102362 | 155.91 | 0.23 | 0.0017 | 0.0342 | 0.95 |
| SYTL5 | ENSG00000147041 | 34.89 | 0.24 | 0.0025 | 0.0342 | 0.96 |
| SZT2 | ENSG00000198198 | 408.18 | 0.21 | 0.0059 | 0.0365 | 1.07 |
| TAB1 | ENSG00000100324 | 55.58 | 0.21 | 0.0060 | 0.0366 | 1.34 |
| TAB2 | ENSG00000055208 | 247.21 | 0.18 | 0.0146 | 0.0481 | 1.26 |
| TACC2 | ENSG00000138162 | 527.97 | 0.20 | 0.0076 | 0.0382 | 1.17 |
| TACO1 | ENSG00000136463 | 15.19 | 0.24 | 0.0008 | 0.0342 | 1.59 |
| TADA3 | ENSG00000171148 | 96.63 | 0.22 | 0.0032 | 0.0342 | 1.42 |
| TAF15 | ENSG00000172660 | 111.61 | 0.18 | 0.0146 | 0.0481 | 1.93 |
| TAF4 | ENSG00000130699 | 73.68 | 0.23 | 0.0019 | 0.0342 | 2.60 |
| TAF5 | ENSG00000148835 | 28.14 | 0.19 | 0.0091 | 0.0407 | 1.39 |
| TAF6 | ENSG00000106290 | 62.33 | 0.20 | 0.0080 | 0.0389 | 2.06 |
| TAF7 | ENSG00000178913 | 89.33 | 0.19 | 0.0097 | 0.0415 | 1.81 |
| TALDO1 | ENSG00000177156 | 61.22 | 0.18 | 0.0126 | 0.0452 | 2.49 |
| TAMM41 | ENSG00000144559 | 63.18 | 0.19 | 0.0089 | 0.0403 | 1.58 |
| TANC1 | ENSG00000115183 | 352.55 | 0.20 | 0.0063 | 0.0370 | 1.40 |
| TANC2 | ENSG00000170921 | 107.30 | 0.22 | 0.0038 | 0.0342 | 1.28 |
| TAOK3 | ENSG00000135090 | 217.93 | 0.20 | 0.0091 | 0.0407 | 1.22 |
| TAP1 | ENSG00000168394 | 148.01 | 0.24 | 0.0055 | 0.0363 | 2.40 |
| TAPBP | ENSG00000231925 | 579.39 | 0.22 | 0.0027 | 0.0342 | 1.45 |
| TAPBPL | ENSG00000139192 | 90.96 | 0.22 | 0.0026 | 0.0342 | 1.10 |
| TARS | ENSG00000113407 | 105.06 | 0.19 | 0.0079 | 0.0387 | 2.68 |
| TAS2R31 | ENSG00000256436 | 11.49 | 0.24 | 0.0015 | 0.0342 | 1.55 |
| TATDN1 | ENSG00000147687 | 29.98 | 0.20 | 0.0087 | 0.0401 | 2.25 |
| TATDN3 | ENSG00000203705 | 15.81 | 0.20 | 0.0087 | 0.0401 | 1.24 |
| TAX1BP1 | ENSG00000106052 | 236.42 | 0.24 | 0.0010 | 0.0342 | 1.99 |
| TAX1BP3 | ENSG00000213977 | 156.18 | 0.20 | 0.0070 | 0.0376 | 1.04 |
| TAZ | ENSG00000102125 | 68.01 | 0.19 | 0.0103 | 0.0423 | 1.62 |
| TBC1D10B | ENSG00000169221 | 131.70 | 0.19 | 0.0122 | 0.0446 | 1.66 |
| TBC1D13 | ENSG00000107021 | 53.10 | 0.20 | 0.0077 | 0.0384 | 1.50 |
| TBC1D14 | ENSG00000132405 | 247.27 | 0.22 | 0.0023 | 0.0342 | 1.40 |
| TBC1D17 | ENSG00000104946 | 52.86 | 0.21 | 0.0055 | 0.0363 | 1.34 |
| TBC1D23 | ENSG00000036054 | 64.45 | 0.21 | 0.0040 | 0.0346 | 1.65 |
| TBC1D24 | ENSG00000162065 | 33.24 | 0.24 | 0.0016 | 0.0342 | 2.49 |
| TBC1D2B | ENSG00000167202 | 224.31 | 0.23 | 0.0012 | 0.0342 | 1.15 |
| TBC1D8B | ENSG00000133138 | 56.45 | 0.32 | 0.0002 | 0.0342 | 1.37 |
| TBC1D9B | ENSG00000197226 | 392.13 | 0.23 | 0.0015 | 0.0342 | 1.49 |
| TBCB | ENSG00000105254 | 35.22 | 0.22 | 0.0047 | 0.0353 | 2.04 |
| TBL1X | ENSG00000101849 | 63.60 | 0.21 | 0.0046 | 0.0352 | 1.96 |
| TBL1XR1 | ENSG00000177565 | 487.75 | 0.23 | 0.0023 | 0.0342 | 1.77 |
| TBL2 | ENSG00000106638 | 57.06 | 0.19 | 0.0100 | 0.0419 | 2.05 |
| TBL3 | ENSG00000183751 | 32.43 | 0.19 | 0.0122 | 0.0446 | 2.26 |
| TBRG4 | ENSG00000136270 | 108.17 | 0.19 | 0.0150 | 0.0486 | 2.45 |
| TBX3 | ENSG00000135111 | 57.06 | 0.22 | 0.0057 | 0.0363 | 2.20 |
| TCEA3 | ENSG00000204219 | 186.96 | 0.22 | 0.0035 | 0.0342 | 0.63 |
| TCEAL1 | ENSG00000172465 | 12.62 | 0.19 | 0.0128 | 0.0456 | 2.18 |
| TCEANC | ENSG00000176896 | 12.05 | 0.20 | 0.0076 | 0.0382 | 1.28 |
| TCEB3 | ENSG00000011007 | 149.25 | 0.19 | 0.0083 | 0.0395 | 1.30 |
| TCF12 | ENSG00000140262 | 219.72 | 0.19 | 0.0096 | 0.0414 | 1.92 |
| TCF20 | ENSG00000100207 | 359.54 | 0.21 | 0.0070 | 0.0376 | 2.01 |
| TCF7L2 | ENSG00000148737 | 304.21 | 0.20 | 0.0074 | 0.0380 | 0.95 |
| TCL1A | ENSG00000100721 | 10.05 | -0.21 | 0.0096 | 0.0414 | 0.15 |
| TCN2 | ENSG00000185339 | 27.53 | 0.22 | 0.0030 | 0.0342 | 0.79 |
| TCP10 | ENSG00000203690 | 6.07 | 0.19 | 0.0136 | 0.0466 | 1.01 |
| TCP11L1 | ENSG00000176148 | 73.64 | 0.22 | 0.0032 | 0.0342 | 0.94 |
| TCTA | ENSG00000145022 | 60.84 | 0.24 | 0.0006 | 0.0342 | 1.29 |
| TCTN3 | ENSG00000119977 | 57.52 | 0.20 | 0.0047 | 0.0353 | 1.67 |
| TDP1 | ENSG00000042088 | 38.47 | 0.18 | 0.0138 | 0.0468 | 1.71 |
| TDP2 | ENSG00000111802 | 200.25 | 0.26 | 0.0009 | 0.0342 | 0.92 |
| TDRD3 | ENSG00000083544 | 66.37 | 0.19 | 0.0092 | 0.0408 | 1.52 |
| TDRD5 | ENSG00000162782 | 7.21 | 0.20 | 0.0121 | 0.0445 | 2.03 |
| TDRKH | ENSG00000182134 | 16.89 | 0.23 | 0.0034 | 0.0342 | 2.26 |
| TEAD3 | ENSG00000007866 | 128.30 | 0.21 | 0.0048 | 0.0354 | 1.25 |
| TECPR2 | ENSG00000196663 | 80.26 | 0.21 | 0.0046 | 0.0352 | 1.23 |
| TEF | ENSG00000167074 | 66.28 | 0.21 | 0.0053 | 0.0361 | 0.71 |
| TEN1 | ENSG00000108504 | 47.16 | 0.20 | 0.0079 | 0.0387 | 1.44 |
| TEP1 | ENSG00000129566 | 535.68 | 0.24 | 0.0011 | 0.0342 | 0.79 |
| TET3 | ENSG00000187605 | 166.69 | 0.19 | 0.0095 | 0.0413 | 1.56 |
| TEX2 | ENSG00000136478 | 120.77 | 0.17 | 0.0114 | 0.0437 | 1.57 |
| TEX261 | ENSG00000144043 | 120.19 | 0.18 | 0.0124 | 0.0450 | 1.70 |
| TEX264 | ENSG00000164081 | 69.82 | 0.21 | 0.0042 | 0.0350 | 1.32 |
| TFAP4 | ENSG00000090447 | 19.31 | 0.22 | 0.0044 | 0.0351 | 3.40 |
| TFB1M | ENSG00000029639 | 22.87 | 0.19 | 0.0099 | 0.0417 | 1.69 |
| TFB2M | ENSG00000162851 | 12.05 | 0.20 | 0.0123 | 0.0448 | 2.29 |
| TFCP2 | ENSG00000135457 | 93.18 | 0.19 | 0.0100 | 0.0419 | 1.78 |
| TFDP2 | ENSG00000114126 | 177.77 | 0.27 | 0.0002 | 0.0342 | 1.62 |
| TFF1 | ENSG00000160182 | 52.06 | 0.20 | 0.0148 | 0.0484 | 1.29 |
| TFG | ENSG00000114354 | 181.27 | 0.19 | 0.0117 | 0.0439 | 1.53 |
| TFR2 | ENSG00000106327 | 5.14 | 0.20 | 0.0107 | 0.0428 | 2.67 |
| TGFBR1 | ENSG00000106799 | 118.18 | 0.22 | 0.0028 | 0.0342 | 2.07 |
| TGFBRAP1 | ENSG00000135966 | 88.61 | 0.18 | 0.0129 | 0.0457 | 2.01 |
| TGOLN2 | ENSG00000152291 | 794.18 | 0.23 | 0.0016 | 0.0342 | 1.51 |
| THAP3 | ENSG00000041988 | 85.76 | 0.22 | 0.0020 | 0.0342 | 1.20 |
| THAP4 | ENSG00000176946 | 60.81 | 0.21 | 0.0032 | 0.0342 | 1.70 |
| THAP8 | ENSG00000161277 | 9.16 | 0.26 | 0.0012 | 0.0342 | 2.02 |
| THG1L | ENSG00000113272 | 30.14 | 0.20 | 0.0071 | 0.0376 | 1.44 |
| THOC3 | ENSG00000051596 | 17.98 | 0.18 | 0.0141 | 0.0472 | 2.45 |
| THOC6 | ENSG00000131652 | 45.39 | 0.18 | 0.0143 | 0.0476 | 1.43 |
| THOC7 | ENSG00000163634 | 31.17 | 0.19 | 0.0100 | 0.0419 | 2.77 |
| THRA | ENSG00000126351 | 138.98 | 0.18 | 0.0132 | 0.0460 | 1.86 |
| THRAP3 | ENSG00000054118 | 300.23 | 0.19 | 0.0098 | 0.0416 | 1.65 |
| THSD4 | ENSG00000187720 | 195.94 | 0.18 | 0.0139 | 0.0469 | 0.83 |
| THUMPD1 | ENSG00000066654 | 115.80 | 0.22 | 0.0025 | 0.0342 | 1.56 |
| THYN1 | ENSG00000151500 | 21.32 | 0.19 | 0.0134 | 0.0463 | 1.67 |
| TIA1 | ENSG00000116001 | 244.13 | 0.21 | 0.0065 | 0.0371 | 1.45 |
| TIAF1 | ENSG00000221995 | 170.65 | 0.22 | 0.0041 | 0.0349 | 1.22 |
| TIAL1 | ENSG00000151923 | 125.43 | 0.22 | 0.0023 | 0.0342 | 1.68 |
| TIGD2 | ENSG00000180346 | 12.37 | 0.24 | 0.0037 | 0.0342 | 2.24 |
| TIGD5 | ENSG00000179886 | 18.17 | 0.22 | 0.0062 | 0.0368 | 1.93 |
| TIGD6 | ENSG00000164296 | 33.73 | 0.20 | 0.0089 | 0.0403 | 0.91 |
| TIMM13 | ENSG00000099800 | 45.70 | 0.20 | 0.0089 | 0.0403 | 1.84 |
| TIMMDC1 | ENSG00000113845 | 53.77 | 0.21 | 0.0037 | 0.0342 | 1.63 |
| TIPRL | ENSG00000143155 | 35.69 | 0.18 | 0.0128 | 0.0456 | 1.76 |
| TIRAP | ENSG00000150455 | 48.32 | 0.20 | 0.0055 | 0.0363 | 1.12 |
| TJP2 | ENSG00000119139 | 282.19 | 0.25 | 0.0010 | 0.0342 | 2.05 |
| TJP3 | ENSG00000105289 | 264.20 | 0.22 | 0.0032 | 0.0342 | 0.74 |
| TK1 | ENSG00000167900 | 27.68 | 0.19 | 0.0148 | 0.0484 | 2.12 |
| TK2 | ENSG00000166548 | 67.41 | 0.18 | 0.0143 | 0.0476 | 1.42 |
| TKT | ENSG00000163931 | 174.71 | 0.20 | 0.0077 | 0.0384 | 3.27 |
| TLE1 | ENSG00000196781 | 123.13 | 0.18 | 0.0117 | 0.0439 | 0.98 |
| TLE2 | ENSG00000065717 | 80.38 | 0.18 | 0.0157 | 0.0496 | 1.53 |
| TLK1 | ENSG00000198586 | 269.18 | 0.19 | 0.0064 | 0.0370 | 1.38 |
| TLK2 | ENSG00000146872 | 84.20 | 0.19 | 0.0082 | 0.0393 | 1.42 |
| TLN2 | ENSG00000171914 | 543.31 | 0.21 | 0.0039 | 0.0345 | 0.69 |
| TLR3 | ENSG00000164342 | 27.16 | 0.30 | 0.0004 | 0.0342 | 0.72 |
| TLX1 | ENSG00000107807 | 3.59 | 0.21 | 0.0144 | 0.0477 | 6.21 |
| TM2D1 | ENSG00000162604 | 52.00 | 0.23 | 0.0028 | 0.0342 | 1.19 |
| TM7SF2 | ENSG00000149809 | 24.36 | 0.20 | 0.0092 | 0.0408 | 1.62 |
| TM7SF3 | ENSG00000064115 | 88.11 | 0.24 | 0.0008 | 0.0342 | 1.71 |
| TM9SF1 | ENSG00000100926 | 86.36 | 0.18 | 0.0139 | 0.0469 | 1.42 |
| TM9SF2 | ENSG00000125304 | 275.39 | 0.23 | 0.0014 | 0.0342 | 2.18 |
| TM9SF3 | ENSG00000077147 | 685.21 | 0.25 | 0.0016 | 0.0342 | 1.45 |
| TM9SF4 | ENSG00000101337 | 190.66 | 0.22 | 0.0026 | 0.0342 | 2.32 |
| TMBIM1 | ENSG00000135926 | 396.59 | 0.20 | 0.0076 | 0.0382 | 1.26 |
| TMBIM6 | ENSG00000139644 | 1030.53 | 0.24 | 0.0005 | 0.0342 | 1.47 |
| TMC4 | ENSG00000167608 | 199.81 | 0.25 | 0.0009 | 0.0342 | 1.00 |
| TMC5 | ENSG00000103534 | 374.80 | 0.25 | 0.0017 | 0.0342 | 1.26 |
| TMCC1 | ENSG00000172765 | 100.90 | 0.19 | 0.0075 | 0.0382 | 1.78 |
| TMCC3 | ENSG00000057704 | 133.74 | 0.19 | 0.0093 | 0.0409 | 0.61 |
| TMCO1 | ENSG00000143183 | 108.74 | 0.27 | 0.0002 | 0.0342 | 1.32 |
| TMCO3 | ENSG00000150403 | 89.71 | 0.21 | 0.0052 | 0.0359 | 1.71 |
| TMCO4 | ENSG00000162542 | 81.31 | 0.22 | 0.0037 | 0.0342 | 1.09 |
| TMED1 | ENSG00000099203 | 44.07 | 0.22 | 0.0036 | 0.0342 | 1.20 |
| TMED10 | ENSG00000170348 | 422.93 | 0.22 | 0.0024 | 0.0342 | 1.50 |
| TMED2 | ENSG00000086598 | 272.54 | 0.20 | 0.0064 | 0.0370 | 2.05 |
| TMED3 | ENSG00000166557 | 58.52 | 0.24 | 0.0019 | 0.0342 | 1.60 |
| TMED4 | ENSG00000158604 | 175.77 | 0.21 | 0.0053 | 0.0361 | 1.64 |
| TMED7 | ENSG00000134970 | 135.67 | 0.19 | 0.0142 | 0.0474 | 1.61 |
| TMED7-TICAM2 | ENSG00000251201 | 38.65 | 0.19 | 0.0134 | 0.0463 | 1.52 |
| TMED9 | ENSG00000184840 | 133.03 | 0.18 | 0.0141 | 0.0472 | 1.53 |
| TMEM101 | ENSG00000091947 | 17.24 | 0.19 | 0.0093 | 0.0409 | 1.93 |
| TMEM105 | ENSG00000185332 | 3.43 | 0.19 | 0.0161 | 0.0500 | 2.61 |
| TMEM106B | ENSG00000106460 | 122.54 | 0.24 | 0.0022 | 0.0342 | 1.95 |
| TMEM106C | ENSG00000134291 | 106.87 | 0.22 | 0.0027 | 0.0342 | 1.50 |
| TMEM11 | ENSG00000178307 | 19.36 | 0.20 | 0.0098 | 0.0416 | 1.28 |
| TMEM111 | ENSG00000125037 | 66.07 | 0.17 | 0.0157 | 0.0496 | 1.49 |
| TMEM115 | ENSG00000126062 | 59.59 | 0.22 | 0.0029 | 0.0342 | 1.34 |
| TMEM117 | ENSG00000139173 | 18.46 | 0.19 | 0.0158 | 0.0497 | 1.50 |
| TMEM120A | ENSG00000189077 | 41.92 | 0.19 | 0.0112 | 0.0434 | 1.35 |
| TMEM123 | ENSG00000152558 | 320.41 | 0.21 | 0.0062 | 0.0368 | 2.88 |
| TMEM125 | ENSG00000179178 | 47.79 | 0.25 | 0.0010 | 0.0342 | 1.08 |
| TMEM126A | ENSG00000171202 | 11.86 | 0.21 | 0.0044 | 0.0351 | 1.71 |
| TMEM127 | ENSG00000135956 | 231.97 | 0.21 | 0.0050 | 0.0356 | 1.17 |
| TMEM128 | ENSG00000132406 | 19.92 | 0.22 | 0.0032 | 0.0342 | 1.45 |
| TMEM129 | ENSG00000168936 | 98.65 | 0.22 | 0.0026 | 0.0342 | 1.19 |
| TMEM131 | ENSG00000075568 | 432.65 | 0.19 | 0.0067 | 0.0373 | 0.98 |
| TMEM134 | ENSG00000172663 | 44.61 | 0.21 | 0.0072 | 0.0377 | 1.28 |
| TMEM135 | ENSG00000166575 | 48.30 | 0.18 | 0.0150 | 0.0486 | 1.37 |
| TMEM139 | ENSG00000178826 | 19.08 | 0.23 | 0.0050 | 0.0356 | 2.43 |
| TMEM141 | ENSG00000244187 | 96.57 | 0.26 | 0.0005 | 0.0342 | 1.59 |
| TMEM144 | ENSG00000164124 | 80.85 | 0.24 | 0.0012 | 0.0342 | 1.18 |
| TMEM14B | ENSG00000137210 | 41.52 | 0.21 | 0.0049 | 0.0355 | 1.76 |
| TMEM14C | ENSG00000111843 | 39.55 | 0.20 | 0.0059 | 0.0365 | 2.04 |
| TMEM150A | ENSG00000168890 | 18.95 | 0.18 | 0.0159 | 0.0498 | 1.66 |
| TMEM150B | ENSG00000180061 | 10.25 | 0.20 | 0.0108 | 0.0429 | 2.50 |
| TMEM159 | ENSG00000011638 | 27.20 | 0.28 | 0.0004 | 0.0342 | 1.98 |
| TMEM163 | ENSG00000152128 | 10.85 | -0.20 | 0.0104 | 0.0424 | 1.29 |
| TMEM164 | ENSG00000157600 | 240.29 | 0.21 | 0.0051 | 0.0358 | 1.21 |
| TMEM167A | ENSG00000174695 | 138.26 | 0.20 | 0.0069 | 0.0375 | 1.45 |
| TMEM167B | ENSG00000215717 | 61.30 | 0.24 | 0.0012 | 0.0342 | 1.38 |
| TMEM168 | ENSG00000146802 | 148.68 | 0.19 | 0.0116 | 0.0439 | 1.39 |
| TMEM170A | ENSG00000166822 | 126.30 | 0.23 | 0.0016 | 0.0342 | 1.13 |
| TMEM171 | ENSG00000157111 | 43.90 | 0.20 | 0.0068 | 0.0374 | 0.50 |
| TMEM173 | ENSG00000184584 | 98.93 | 0.22 | 0.0036 | 0.0342 | 1.18 |
| TMEM176B | ENSG00000106565 | 166.14 | 0.21 | 0.0050 | 0.0356 | 1.38 |
| TMEM177 | ENSG00000144120 | 23.75 | 0.22 | 0.0032 | 0.0342 | 1.89 |
| TMEM179B | ENSG00000185475 | 42.98 | 0.24 | 0.0009 | 0.0342 | 1.39 |
| TMEM180 | ENSG00000138111 | 29.22 | 0.21 | 0.0060 | 0.0366 | 1.53 |
| TMEM181 | ENSG00000146433 | 265.94 | 0.23 | 0.0017 | 0.0342 | 1.44 |
| TMEM182 | ENSG00000170417 | 9.36 | 0.23 | 0.0046 | 0.0352 | 2.23 |
| TMEM183A | ENSG00000163444 | 74.22 | 0.18 | 0.0122 | 0.0446 | 1.41 |
| TMEM184A | ENSG00000164855 | 127.35 | 0.23 | 0.0028 | 0.0342 | 1.00 |
| TMEM184B | ENSG00000198792 | 145.21 | 0.22 | 0.0040 | 0.0346 | 1.72 |
| TMEM184C | ENSG00000164168 | 85.20 | 0.22 | 0.0021 | 0.0342 | 1.42 |
| TMEM187 | ENSG00000177854 | 7.14 | 0.24 | 0.0019 | 0.0342 | 2.19 |
| TMEM188 | ENSG00000205423 | 20.69 | 0.23 | 0.0026 | 0.0342 | 1.45 |
| TMEM189 | ENSG00000240849 | 180.84 | 0.21 | 0.0057 | 0.0363 | 2.60 |
| TMEM189-UBE2V1 | ENSG00000124208 | 133.71 | 0.21 | 0.0035 | 0.0342 | 2.60 |
| TMEM192 | ENSG00000170088 | 47.53 | 0.22 | 0.0026 | 0.0342 | 1.57 |
| TMEM200A | ENSG00000164484 | 57.50 | 0.21 | 0.0066 | 0.0373 | 1.47 |
| TMEM203 | ENSG00000187713 | 28.06 | 0.24 | 0.0014 | 0.0342 | 1.76 |
| TMEM205 | ENSG00000105518 | 26.79 | 0.20 | 0.0114 | 0.0437 | 1.60 |
| TMEM208 | ENSG00000168701 | 14.11 | 0.19 | 0.0146 | 0.0481 | 1.97 |
| TMEM219 | ENSG00000149932 | 71.91 | 0.23 | 0.0012 | 0.0342 | 1.40 |
| TMEM223 | ENSG00000168569 | 44.57 | 0.25 | 0.0014 | 0.0342 | 1.34 |
| TMEM236 | ENSG00000148483 | 59.55 | 0.20 | 0.0087 | 0.0401 | 0.28 |
| TMEM30A | ENSG00000112697 | 202.47 | 0.21 | 0.0056 | 0.0363 | 1.63 |
| TMEM30B | ENSG00000182107 | 154.52 | 0.21 | 0.0032 | 0.0342 | 0.73 |
| TMEM33 | ENSG00000109133 | 186.67 | 0.21 | 0.0057 | 0.0363 | 1.99 |
| TMEM37 | ENSG00000171227 | 43.70 | 0.21 | 0.0061 | 0.0368 | 0.60 |
| TMEM39B | ENSG00000121775 | 20.56 | 0.22 | 0.0046 | 0.0352 | 1.44 |
| TMEM42 | ENSG00000169964 | 18.89 | 0.18 | 0.0127 | 0.0454 | 1.57 |
| TMEM44 | ENSG00000145014 | 104.41 | 0.22 | 0.0028 | 0.0342 | 1.11 |
| TMEM45A | ENSG00000181458 | 8.75 | 0.22 | 0.0036 | 0.0342 | 1.42 |
| TMEM45B | ENSG00000151715 | 234.95 | 0.28 | 0.0002 | 0.0342 | 0.84 |
| TMEM48 | ENSG00000058804 | 68.29 | 0.19 | 0.0141 | 0.0472 | 2.49 |
| TMEM5 | ENSG00000118600 | 22.43 | 0.22 | 0.0027 | 0.0342 | 1.95 |
| TMEM50A | ENSG00000183726 | 99.16 | 0.23 | 0.0020 | 0.0342 | 1.36 |
| TMEM50B | ENSG00000142188 | 101.22 | 0.18 | 0.0154 | 0.0491 | 1.14 |
| TMEM51 | ENSG00000171729 | 37.03 | 0.22 | 0.0026 | 0.0342 | 1.42 |
| TMEM53 | ENSG00000126106 | 46.63 | 0.20 | 0.0077 | 0.0384 | 1.29 |
| TMEM54 | ENSG00000121900 | 219.93 | 0.18 | 0.0138 | 0.0468 | 0.68 |
| TMEM57 | ENSG00000204178 | 78.36 | 0.19 | 0.0077 | 0.0384 | 1.27 |
| TMEM59 | ENSG00000116209 | 214.41 | 0.24 | 0.0008 | 0.0342 | 1.00 |
| TMEM62 | ENSG00000137842 | 52.10 | 0.24 | 0.0018 | 0.0342 | 1.69 |
| TMEM63A | ENSG00000196187 | 709.17 | 0.26 | 0.0006 | 0.0342 | 2.37 |
| TMEM63B | ENSG00000137216 | 141.46 | 0.22 | 0.0031 | 0.0342 | 1.10 |
| TMEM63C | ENSG00000165548 | 25.11 | 0.22 | 0.0062 | 0.0368 | 1.61 |
| TMEM66 | ENSG00000133872 | 237.47 | 0.19 | 0.0095 | 0.0413 | 1.27 |
| TMEM68 | ENSG00000167904 | 43.09 | 0.19 | 0.0094 | 0.0411 | 2.03 |
| TMEM69 | ENSG00000159596 | 31.42 | 0.21 | 0.0059 | 0.0365 | 1.66 |
| TMEM72 | ENSG00000187783 | 37.98 | 0.21 | 0.0066 | 0.0373 | 0.17 |
| TMEM79 | ENSG00000163472 | 25.63 | 0.25 | 0.0009 | 0.0342 | 1.57 |
| TMEM80 | ENSG00000177042 | 75.19 | 0.27 | 0.0004 | 0.0342 | 1.22 |
| TMEM85 | ENSG00000128463 | 28.20 | 0.21 | 0.0040 | 0.0346 | 1.74 |
| TMEM87B | ENSG00000153214 | 202.39 | 0.26 | 0.0008 | 0.0342 | 1.51 |
| TMEM8A | ENSG00000129925 | 168.46 | 0.20 | 0.0073 | 0.0379 | 1.52 |
| TMEM8B | ENSG00000137103 | 68.94 | 0.24 | 0.0029 | 0.0342 | 1.11 |
| TMEM92 | ENSG00000167105 | 17.08 | 0.18 | 0.0151 | 0.0487 | 1.80 |
| TMEM98 | ENSG00000006042 | 80.21 | 0.23 | 0.0016 | 0.0342 | 1.18 |
| TMEM9B | ENSG00000175348 | 71.13 | 0.20 | 0.0053 | 0.0361 | 1.01 |
| TMLHE | ENSG00000185973 | 27.70 | 0.21 | 0.0033 | 0.0342 | 1.74 |
| TMOD3 | ENSG00000138594 | 217.27 | 0.20 | 0.0040 | 0.0346 | 1.24 |
| TMPO | ENSG00000120802 | 207.55 | 0.22 | 0.0054 | 0.0362 | 2.22 |
| TMPPE | ENSG00000188167 | 52.02 | 0.20 | 0.0065 | 0.0371 | 1.47 |
| TMPRSS4 | ENSG00000137648 | 610.66 | 0.21 | 0.0047 | 0.0353 | 1.34 |
| TMSB10 | ENSG00000034510 | 448.23 | 0.20 | 0.0062 | 0.0368 | 2.28 |
| TMTC2 | ENSG00000179104 | 93.42 | 0.18 | 0.0131 | 0.0460 | 1.26 |
| TMTC3 | ENSG00000139324 | 56.51 | 0.23 | 0.0022 | 0.0342 | 1.84 |
| TMTC4 | ENSG00000125247 | 49.98 | 0.26 | 0.0009 | 0.0342 | 3.09 |
| TMUB2 | ENSG00000168591 | 76.74 | 0.25 | 0.0006 | 0.0342 | 1.14 |
| TMX2 | ENSG00000213593 | 89.56 | 0.18 | 0.0115 | 0.0438 | 1.41 |
| TNFAIP8L1 | ENSG00000185361 | 44.17 | 0.19 | 0.0111 | 0.0433 | 1.71 |
| TNFRSF10D | ENSG00000173530 | 42.60 | 0.21 | 0.0049 | 0.0355 | 1.28 |
| TNFRSF11A | ENSG00000141655 | 231.84 | 0.26 | 0.0005 | 0.0342 | 0.52 |
| TNFRSF14 | ENSG00000157873 | 352.07 | 0.23 | 0.0040 | 0.0346 | 0.95 |
| TNFRSF25 | ENSG00000215788 | 87.70 | 0.22 | 0.0023 | 0.0342 | 1.69 |
| TNFRSF6B | ENSG00000243509 | 39.87 | 0.22 | 0.0085 | 0.0399 | 2.83 |
| TNFSF10 | ENSG00000121858 | 128.90 | 0.28 | 0.0004 | 0.0342 | 0.89 |
| TNFSF12 | ENSG00000239697 | 94.55 | 0.25 | 0.0009 | 0.0342 | 0.88 |
| TNFSF12-TNFSF13 | ENSG00000248871 | 80.31 | 0.26 | 0.0005 | 0.0342 | 0.82 |
| TNFSF13 | ENSG00000161955 | 83.61 | 0.26 | 0.0004 | 0.0342 | 0.81 |
| TNFSF15 | ENSG00000181634 | 14.19 | 0.25 | 0.0042 | 0.0350 | 2.46 |
| TNIP1 | ENSG00000145901 | 294.81 | 0.24 | 0.0011 | 0.0342 | 1.25 |
| TNK1 | ENSG00000174292 | 37.44 | 0.22 | 0.0031 | 0.0342 | 1.13 |
| TNPO1 | ENSG00000083312 | 297.68 | 0.22 | 0.0031 | 0.0342 | 1.77 |
| TNPO2 | ENSG00000105576 | 168.94 | 0.19 | 0.0092 | 0.0408 | 1.98 |
| TNPO3 | ENSG00000064419 | 121.21 | 0.22 | 0.0019 | 0.0342 | 2.08 |
| TNRC6A | ENSG00000090905 | 347.50 | 0.19 | 0.0104 | 0.0424 | 1.18 |
| TNRC6B | ENSG00000100354 | 788.23 | 0.19 | 0.0094 | 0.0411 | 1.00 |
| TNS3 | ENSG00000136205 | 425.91 | 0.20 | 0.0071 | 0.0376 | 2.28 |
| TOB1 | ENSG00000141232 | 157.38 | 0.23 | 0.0016 | 0.0342 | 1.76 |
| TOLLIP | ENSG00000078902 | 91.22 | 0.21 | 0.0031 | 0.0342 | 1.42 |
| TOM1 | ENSG00000100284 | 82.71 | 0.25 | 0.0015 | 0.0342 | 1.42 |
| TOM1L1 | ENSG00000141198 | 70.38 | 0.23 | 0.0026 | 0.0342 | 1.71 |
| TOM1L2 | ENSG00000175662 | 111.02 | 0.19 | 0.0097 | 0.0415 | 0.97 |
| TOMM20 | ENSG00000173726 | 152.58 | 0.22 | 0.0039 | 0.0345 | 2.78 |
| TOMM40 | ENSG00000130204 | 48.16 | 0.18 | 0.0140 | 0.0471 | 2.42 |
| TOMM40L | ENSG00000158882 | 34.33 | 0.24 | 0.0015 | 0.0342 | 1.17 |
| TOMM70A | ENSG00000154174 | 141.62 | 0.20 | 0.0076 | 0.0382 | 2.03 |
| TONSL | ENSG00000160949 | 64.60 | 0.21 | 0.0046 | 0.0352 | 2.49 |
| TOP2A | ENSG00000131747 | 111.38 | 0.23 | 0.0046 | 0.0352 | 4.85 |
| TOP2B | ENSG00000077097 | 323.01 | 0.18 | 0.0137 | 0.0467 | 1.85 |
| TOP3A | ENSG00000177302 | 73.57 | 0.20 | 0.0056 | 0.0363 | 1.53 |
| TOP3B | ENSG00000100038 | 59.67 | 0.18 | 0.0159 | 0.0498 | 1.30 |
| TOPBP1 | ENSG00000163781 | 105.84 | 0.20 | 0.0058 | 0.0364 | 2.25 |
| TOPORS | ENSG00000197579 | 58.87 | 0.22 | 0.0024 | 0.0342 | 1.71 |
| TOR1A | ENSG00000136827 | 57.45 | 0.21 | 0.0034 | 0.0342 | 1.56 |
| TOR1AIP2 | ENSG00000169905 | 317.86 | 0.24 | 0.0010 | 0.0342 | 1.76 |
| TOR1B | ENSG00000136816 | 89.00 | 0.25 | 0.0021 | 0.0342 | 1.09 |
| TOX3 | ENSG00000103460 | 93.37 | 0.29 | 0.0002 | 0.0342 | 1.67 |
| TP53 | ENSG00000141510 | 107.86 | 0.21 | 0.0058 | 0.0364 | 2.45 |
| TP53I11 | ENSG00000175274 | 270.27 | 0.19 | 0.0100 | 0.0419 | 1.24 |
| TP53I13 | ENSG00000167543 | 48.01 | 0.23 | 0.0029 | 0.0342 | 1.27 |
| TP53I3 | ENSG00000115129 | 114.04 | 0.19 | 0.0100 | 0.0419 | 0.63 |
| TPBG | ENSG00000146242 | 46.83 | 0.21 | 0.0044 | 0.0351 | 1.31 |
| TPCN1 | ENSG00000186815 | 385.67 | 0.25 | 0.0009 | 0.0342 | 1.03 |
| TPD52 | ENSG00000076554 | 417.03 | 0.20 | 0.0072 | 0.0377 | 1.12 |
| TPD52L2 | ENSG00000101150 | 118.71 | 0.20 | 0.0051 | 0.0358 | 3.19 |
| TPI1 | ENSG00000111669 | 237.40 | 0.21 | 0.0047 | 0.0353 | 1.88 |
| TPK1 | ENSG00000196511 | 46.77 | 0.20 | 0.0073 | 0.0379 | 0.74 |
| TPM1 | ENSG00000140416 | 608.16 | 0.19 | 0.0070 | 0.0376 | 1.32 |
| TPMT | ENSG00000137364 | 67.67 | 0.23 | 0.0019 | 0.0342 | 1.39 |
| TPR | ENSG00000047410 | 454.81 | 0.18 | 0.0145 | 0.0479 | 1.88 |
| TPRG1L | ENSG00000158109 | 106.05 | 0.22 | 0.0012 | 0.0342 | 1.16 |
| TPRKB | ENSG00000144034 | 13.78 | 0.23 | 0.0043 | 0.0351 | 1.78 |
| TPRN | ENSG00000176058 | 148.74 | 0.24 | 0.0038 | 0.0342 | 1.42 |
| TPSG1 | ENSG00000116176 | 44.22 | 0.19 | 0.0150 | 0.0486 | 0.37 |
| TPST2 | ENSG00000128294 | 37.88 | 0.19 | 0.0143 | 0.0476 | 1.50 |
| TPT1 | ENSG00000133112 | 1558.67 | 0.19 | 0.0081 | 0.0391 | 2.20 |
| TPX2 | ENSG00000088325 | 45.43 | 0.22 | 0.0057 | 0.0363 | 6.89 |
| TRABD | ENSG00000170638 | 102.32 | 0.20 | 0.0097 | 0.0415 | 1.37 |
| TRADD | ENSG00000102871 | 53.22 | 0.21 | 0.0056 | 0.0363 | 1.38 |
| TRAF2 | ENSG00000127191 | 51.59 | 0.22 | 0.0039 | 0.0345 | 2.15 |
| TRAF3IP2 | ENSG00000056972 | 121.39 | 0.19 | 0.0073 | 0.0379 | 0.96 |
| TRAF7 | ENSG00000131653 | 162.15 | 0.20 | 0.0079 | 0.0387 | 1.89 |
| TRAK1 | ENSG00000182606 | 504.82 | 0.22 | 0.0020 | 0.0342 | 1.09 |
| TRAK2 | ENSG00000115993 | 213.64 | 0.18 | 0.0126 | 0.0452 | 1.06 |
| TRAM1 | ENSG00000067167 | 158.54 | 0.25 | 0.0013 | 0.0342 | 2.04 |
| TRANK1 | ENSG00000168016 | 458.40 | 0.19 | 0.0102 | 0.0422 | 0.72 |
| TRAPPC10 | ENSG00000160218 | 317.21 | 0.19 | 0.0089 | 0.0403 | 1.21 |
| TRAPPC2L | ENSG00000167515 | 13.16 | 0.22 | 0.0029 | 0.0342 | 2.34 |
| TRAPPC3 | ENSG00000054116 | 56.63 | 0.23 | 0.0037 | 0.0342 | 1.51 |
| TRAPPC4 | ENSG00000196655 | 51.52 | 0.27 | 0.0003 | 0.0342 | 1.33 |
| TRAPPC5 | ENSG00000181029 | 15.75 | 0.25 | 0.0014 | 0.0342 | 2.05 |
| TRAPPC8 | ENSG00000153339 | 128.10 | 0.19 | 0.0068 | 0.0374 | 1.07 |
| TREX1 | ENSG00000213689 | 21.05 | 0.19 | 0.0126 | 0.0452 | 1.33 |
| TRIB3 | ENSG00000101255 | 9.24 | 0.23 | 0.0069 | 0.0375 | 11.80 |
| TRIL | ENSG00000176734 | 19.53 | 0.20 | 0.0098 | 0.0416 | 1.52 |
| TRIM11 | ENSG00000154370 | 75.94 | 0.23 | 0.0022 | 0.0342 | 1.63 |
| TRIM14 | ENSG00000106785 | 303.33 | 0.24 | 0.0009 | 0.0342 | 1.09 |
| TRIM16 | ENSG00000221926 | 37.37 | 0.20 | 0.0082 | 0.0393 | 2.16 |
| TRIM17 | ENSG00000162931 | 32.94 | 0.20 | 0.0109 | 0.0431 | 1.33 |
| TRIM2 | ENSG00000109654 | 471.84 | 0.28 | 0.0003 | 0.0342 | 2.18 |
| TRIM21 | ENSG00000132109 | 39.23 | 0.23 | 0.0014 | 0.0342 | 1.48 |
| TRIM23 | ENSG00000113595 | 26.94 | 0.19 | 0.0114 | 0.0437 | 1.28 |
| TRIM26 | ENSG00000234127 | 151.13 | 0.19 | 0.0128 | 0.0456 | 1.48 |
| TRIM27 | ENSG00000204713 | 192.61 | 0.24 | 0.0010 | 0.0342 | 1.83 |
| TRIM28 | ENSG00000130726 | 204.15 | 0.17 | 0.0160 | 0.0499 | 2.54 |
| TRIM3 | ENSG00000110171 | 109.54 | 0.23 | 0.0016 | 0.0342 | 0.95 |
| TRIM32 | ENSG00000119401 | 38.27 | 0.25 | 0.0005 | 0.0342 | 1.76 |
| TRIM37 | ENSG00000108395 | 49.24 | 0.22 | 0.0019 | 0.0342 | 2.13 |
| TRIM38 | ENSG00000112343 | 136.42 | 0.18 | 0.0160 | 0.0499 | 0.99 |
| TRIM39 | ENSG00000204599 | 57.25 | 0.19 | 0.0112 | 0.0434 | 1.45 |
| TRIM39-RPP21 | ENSG00000248167 | 19.27 | 0.23 | 0.0058 | 0.0364 | 1.83 |
| TRIM4 | ENSG00000146833 | 83.69 | 0.20 | 0.0063 | 0.0370 | 1.76 |
| TRIM41 | ENSG00000146063 | 123.01 | 0.20 | 0.0072 | 0.0377 | 1.26 |
| TRIM44 | ENSG00000166326 | 137.03 | 0.20 | 0.0067 | 0.0373 | 1.79 |
| TRIM45 | ENSG00000134253 | 15.23 | 0.18 | 0.0145 | 0.0479 | 1.42 |
| TRIM52 | ENSG00000183718 | 67.81 | 0.23 | 0.0022 | 0.0342 | 1.36 |
| TRIM56 | ENSG00000169871 | 160.31 | 0.20 | 0.0060 | 0.0366 | 1.60 |
| TRIM65 | ENSG00000141569 | 56.66 | 0.23 | 0.0026 | 0.0342 | 2.02 |
| TRIM66 | ENSG00000166436 | 132.02 | 0.20 | 0.0065 | 0.0371 | 1.32 |
| TRIM68 | ENSG00000167333 | 35.04 | 0.26 | 0.0006 | 0.0342 | 1.62 |
| TRIM7 | ENSG00000146054 | 15.85 | 0.24 | 0.0036 | 0.0342 | 1.59 |
| TRIM73 | ENSG00000178809 | 31.05 | 0.20 | 0.0098 | 0.0416 | 1.45 |
| TRIO | ENSG00000038382 | 709.67 | 0.18 | 0.0120 | 0.0444 | 1.75 |
| TRIP11 | ENSG00000100815 | 225.69 | 0.20 | 0.0073 | 0.0379 | 1.27 |
| TRIP13 | ENSG00000071539 | 11.47 | 0.21 | 0.0110 | 0.0432 | 4.93 |
| TRIT1 | ENSG00000043514 | 32.91 | 0.21 | 0.0049 | 0.0355 | 2.17 |
| TRMT112 | ENSG00000173113 | 45.25 | 0.21 | 0.0044 | 0.0351 | 1.93 |
| TRMT2B | ENSG00000188917 | 105.91 | 0.25 | 0.0008 | 0.0342 | 1.66 |
| TRMT5 | ENSG00000126814 | 30.02 | 0.21 | 0.0044 | 0.0351 | 1.86 |
| TRMU | ENSG00000100416 | 100.00 | 0.20 | 0.0069 | 0.0375 | 1.52 |
| TROAP | ENSG00000135451 | 20.71 | 0.20 | 0.0120 | 0.0444 | 3.20 |
| TROVE2 | ENSG00000116747 | 161.83 | 0.19 | 0.0072 | 0.0377 | 1.68 |
| TRPC4AP | ENSG00000100991 | 170.55 | 0.19 | 0.0083 | 0.0395 | 2.13 |
| TRPM4 | ENSG00000130529 | 586.57 | 0.22 | 0.0027 | 0.0342 | 0.62 |
| TRPM6 | ENSG00000119121 | 352.12 | 0.24 | 0.0032 | 0.0342 | 0.18 |
| TRPV1 | ENSG00000196689 | 52.08 | 0.20 | 0.0102 | 0.0422 | 0.85 |
| TRRAP | ENSG00000196367 | 486.70 | 0.18 | 0.0132 | 0.0460 | 1.71 |
| TRUB2 | ENSG00000167112 | 29.92 | 0.19 | 0.0096 | 0.0414 | 1.98 |
| TSC2 | ENSG00000103197 | 283.01 | 0.24 | 0.0023 | 0.0342 | 1.53 |
| TSC22D1 | ENSG00000102804 | 496.28 | 0.22 | 0.0029 | 0.0342 | 1.67 |
| TSC22D4 | ENSG00000166925 | 99.66 | 0.21 | 0.0068 | 0.0374 | 1.55 |
| TSFM | ENSG00000123297 | 25.69 | 0.18 | 0.0138 | 0.0468 | 1.94 |
| TSG101 | ENSG00000074319 | 51.84 | 0.21 | 0.0030 | 0.0342 | 1.76 |
| TSKU | ENSG00000182704 | 58.34 | 0.24 | 0.0017 | 0.0342 | 2.09 |
| TSN | ENSG00000211460 | 84.52 | 0.20 | 0.0085 | 0.0399 | 2.57 |
| TSNARE1 | ENSG00000171045 | 44.91 | 0.21 | 0.0057 | 0.0363 | 2.30 |
| TSPAN1 | ENSG00000117472 | 592.17 | 0.20 | 0.0064 | 0.0370 | 0.33 |
| TSPAN12 | ENSG00000106025 | 48.53 | 0.34 | <.0001 | 0.0342 | 1.61 |
| TSPAN13 | ENSG00000106537 | 108.14 | 0.23 | 0.0041 | 0.0349 | 1.34 |
| TSPAN15 | ENSG00000099282 | 144.99 | 0.23 | 0.0018 | 0.0342 | 1.27 |
| TSPAN17 | ENSG00000048140 | 76.83 | 0.20 | 0.0120 | 0.0444 | 1.58 |
| TSPAN3 | ENSG00000140391 | 639.70 | 0.26 | 0.0010 | 0.0342 | 0.78 |
| TSPAN31 | ENSG00000135452 | 39.89 | 0.19 | 0.0114 | 0.0437 | 1.55 |
| TSPAN6 | ENSG00000000003 | 77.56 | 0.23 | 0.0019 | 0.0342 | 2.26 |
| TSPAN8 | ENSG00000127324 | 336.82 | 0.26 | 0.0007 | 0.0342 | 1.52 |
| TSPO | ENSG00000100300 | 60.18 | 0.18 | 0.0149 | 0.0485 | 1.47 |
| TSPYL1 | ENSG00000189241 | 157.42 | 0.18 | 0.0129 | 0.0457 | 1.38 |
| TSR2 | ENSG00000158526 | 35.59 | 0.21 | 0.0056 | 0.0363 | 2.07 |
| TSSK3 | ENSG00000162526 | 25.55 | 0.19 | 0.0134 | 0.0463 | 0.85 |
| TST | ENSG00000128311 | 104.99 | 0.19 | 0.0113 | 0.0435 | 0.81 |
| TSTA3 | ENSG00000104522 | 71.26 | 0.21 | 0.0056 | 0.0363 | 2.02 |
| TSTD1 | ENSG00000215845 | 27.31 | 0.19 | 0.0090 | 0.0405 | 1.44 |
| TTC1 | ENSG00000113312 | 42.09 | 0.23 | 0.0015 | 0.0342 | 1.70 |
| TTC13 | ENSG00000143643 | 99.93 | 0.21 | 0.0034 | 0.0342 | 0.91 |
| TTC15 | ENSG00000171853 | 183.07 | 0.18 | 0.0115 | 0.0438 | 1.36 |
| TTC17 | ENSG00000052841 | 323.97 | 0.18 | 0.0108 | 0.0429 | 1.49 |
| TTC22 | ENSG00000006555 | 123.96 | 0.24 | 0.0021 | 0.0342 | 0.74 |
| TTC3 | ENSG00000182670 | 817.44 | 0.19 | 0.0087 | 0.0401 | 1.59 |
| TTC30A | ENSG00000197557 | 16.26 | 0.19 | 0.0090 | 0.0405 | 1.37 |
| TTC35 | ENSG00000104412 | 47.67 | 0.20 | 0.0084 | 0.0397 | 1.77 |
| TTC37 | ENSG00000198677 | 195.32 | 0.19 | 0.0094 | 0.0411 | 1.68 |
| TTC38 | ENSG00000075234 | 140.93 | 0.27 | 0.0003 | 0.0342 | 1.05 |
| TTC39A | ENSG00000085831 | 216.65 | 0.20 | 0.0051 | 0.0358 | 0.72 |
| TTC39B | ENSG00000155158 | 96.79 | 0.20 | 0.0082 | 0.0393 | 1.33 |
| TTC4 | ENSG00000243725 | 35.78 | 0.18 | 0.0145 | 0.0479 | 1.41 |
| TTC5 | ENSG00000136319 | 17.76 | 0.19 | 0.0100 | 0.0419 | 1.44 |
| TTC7A | ENSG00000068724 | 115.59 | 0.20 | 0.0057 | 0.0363 | 1.54 |
| TTC8 | ENSG00000165533 | 16.64 | 0.20 | 0.0070 | 0.0376 | 1.71 |
| TTF2 | ENSG00000116830 | 96.05 | 0.19 | 0.0135 | 0.0464 | 1.96 |
| TTLL12 | ENSG00000100304 | 111.83 | 0.23 | 0.0022 | 0.0342 | 1.81 |
| TTLL5 | ENSG00000119685 | 99.44 | 0.19 | 0.0114 | 0.0437 | 1.48 |
| TTLL6 | ENSG00000170703 | 82.78 | 0.23 | 0.0028 | 0.0342 | 0.16 |
| TTYH1 | ENSG00000167614 | 38.64 | 0.23 | 0.0024 | 0.0342 | 1.68 |
| TUBA4A | ENSG00000127824 | 40.73 | 0.18 | 0.0152 | 0.0489 | 2.39 |
| TUBAL3 | ENSG00000178462 | 17.13 | 0.22 | 0.0053 | 0.0361 | 0.52 |
| TUBGCP2 | ENSG00000130640 | 138.01 | 0.20 | 0.0078 | 0.0386 | 1.36 |
| TUBGCP4 | ENSG00000137822 | 47.18 | 0.21 | 0.0032 | 0.0342 | 1.73 |
| TUBGCP6 | ENSG00000128159 | 274.90 | 0.19 | 0.0115 | 0.0438 | 1.15 |
| TUFM | ENSG00000178952 | 157.77 | 0.20 | 0.0062 | 0.0368 | 1.92 |
| TUFT1 | ENSG00000143367 | 70.66 | 0.20 | 0.0064 | 0.0370 | 2.04 |
| TULP3 | ENSG00000078246 | 43.84 | 0.19 | 0.0093 | 0.0409 | 1.90 |
| TULP4 | ENSG00000130338 | 240.48 | 0.20 | 0.0058 | 0.0364 | 2.04 |
| TUSC1 | ENSG00000198680 | 19.60 | 0.20 | 0.0069 | 0.0375 | 1.34 |
| TUSC2 | ENSG00000114383 | 40.02 | 0.21 | 0.0045 | 0.0352 | 1.66 |
| TUT1 | ENSG00000149016 | 47.69 | 0.20 | 0.0071 | 0.0376 | 1.44 |
| TWF1 | ENSG00000151239 | 70.34 | 0.24 | 0.0032 | 0.0342 | 1.87 |
| TWSG1 | ENSG00000128791 | 51.37 | 0.22 | 0.0021 | 0.0342 | 1.39 |
| TXLNA | ENSG00000084652 | 204.86 | 0.19 | 0.0085 | 0.0399 | 1.57 |
| TXN | ENSG00000136810 | 63.57 | 0.19 | 0.0092 | 0.0408 | 2.27 |
| TXN2 | ENSG00000100348 | 64.50 | 0.20 | 0.0045 | 0.0352 | 1.32 |
| TXNDC11 | ENSG00000153066 | 166.05 | 0.22 | 0.0035 | 0.0342 | 0.85 |
| TXNDC12 | ENSG00000117862 | 61.72 | 0.23 | 0.0012 | 0.0342 | 1.86 |
| TXNDC17 | ENSG00000129235 | 32.56 | 0.19 | 0.0083 | 0.0395 | 1.29 |
| TXNDC5 | ENSG00000239264 | 826.97 | 0.20 | 0.0067 | 0.0373 | 0.86 |
| TXNIP | ENSG00000117289 | 1218.80 | 0.20 | 0.0067 | 0.0373 | 0.96 |
| TXNL1 | ENSG00000091164 | 46.92 | 0.24 | 0.0011 | 0.0342 | 1.49 |
| TXNRD2 | ENSG00000184470 | 45.34 | 0.18 | 0.0158 | 0.0497 | 1.53 |
| TYK2 | ENSG00000105397 | 256.66 | 0.19 | 0.0088 | 0.0402 | 1.28 |
| TYSND1 | ENSG00000156521 | 67.48 | 0.21 | 0.0052 | 0.0359 | 1.62 |
| UACA | ENSG00000137831 | 164.78 | 0.20 | 0.0059 | 0.0365 | 1.69 |
| UAP1 | ENSG00000117143 | 174.41 | 0.18 | 0.0139 | 0.0469 | 0.91 |
| UAP1L1 | ENSG00000197355 | 51.21 | 0.22 | 0.0042 | 0.0350 | 1.70 |
| UBA1 | ENSG00000130985 | 394.53 | 0.20 | 0.0066 | 0.0373 | 1.83 |
| UBA2 | ENSG00000126261 | 77.65 | 0.20 | 0.0086 | 0.0399 | 2.95 |
| UBA3 | ENSG00000144744 | 57.05 | 0.19 | 0.0093 | 0.0409 | 1.91 |
| UBA52 | ENSG00000221983 | 161.62 | 0.20 | 0.0052 | 0.0359 | 2.26 |
| UBA7 | ENSG00000182179 | 174.02 | 0.23 | 0.0025 | 0.0342 | 0.87 |
| UBAC1 | ENSG00000130560 | 57.28 | 0.21 | 0.0039 | 0.0345 | 1.26 |
| UBAC2 | ENSG00000134882 | 98.90 | 0.23 | 0.0012 | 0.0342 | 2.31 |
| UBAP2 | ENSG00000137073 | 196.34 | 0.21 | 0.0042 | 0.0350 | 1.94 |
| UBB | ENSG00000170315 | 147.11 | 0.18 | 0.0118 | 0.0441 | 1.51 |
| UBE2A | ENSG00000077721 | 77.64 | 0.20 | 0.0101 | 0.0421 | 1.74 |
| UBE2C | ENSG00000175063 | 15.07 | 0.20 | 0.0109 | 0.0431 | 6.95 |
| UBE2E3 | ENSG00000170035 | 45.71 | 0.21 | 0.0051 | 0.0358 | 1.68 |
| UBE2F | ENSG00000184182 | 64.98 | 0.19 | 0.0075 | 0.0382 | 1.98 |
| UBE2J2 | ENSG00000160087 | 87.69 | 0.21 | 0.0033 | 0.0342 | 1.35 |
| UBE2K | ENSG00000078140 | 163.29 | 0.18 | 0.0124 | 0.0450 | 1.68 |
| UBE2L3 | ENSG00000185651 | 87.68 | 0.21 | 0.0029 | 0.0342 | 1.53 |
| UBE2L6 | ENSG00000156587 | 43.19 | 0.21 | 0.0097 | 0.0415 | 2.28 |
| UBE2M | ENSG00000130725 | 35.49 | 0.20 | 0.0045 | 0.0352 | 1.82 |
| UBE2Q1 | ENSG00000160714 | 102.01 | 0.18 | 0.0123 | 0.0448 | 1.71 |
| UBE2R2 | ENSG00000107341 | 133.23 | 0.20 | 0.0077 | 0.0384 | 1.90 |
| UBE2T | ENSG00000077152 | 6.16 | 0.21 | 0.0114 | 0.0437 | 4.03 |
| UBE2V1 | ENSG00000244687 | 181.16 | 0.19 | 0.0112 | 0.0434 | 2.32 |
| UBE2Z | ENSG00000159202 | 222.50 | 0.19 | 0.0065 | 0.0371 | 1.64 |
| UBE3A | ENSG00000114062 | 204.53 | 0.19 | 0.0065 | 0.0371 | 1.44 |
| UBE3B | ENSG00000151148 | 214.34 | 0.20 | 0.0052 | 0.0359 | 1.25 |
| UBE3C | ENSG00000009335 | 296.08 | 0.20 | 0.0062 | 0.0368 | 2.04 |
| UBE4A | ENSG00000110344 | 262.25 | 0.19 | 0.0060 | 0.0366 | 1.38 |
| UBE4B | ENSG00000130939 | 182.42 | 0.20 | 0.0053 | 0.0361 | 1.43 |
| UBFD1 | ENSG00000103353 | 80.89 | 0.26 | 0.0004 | 0.0342 | 2.61 |
| UBL3 | ENSG00000122042 | 125.99 | 0.22 | 0.0023 | 0.0342 | 1.29 |
| UBL4A | ENSG00000102178 | 33.83 | 0.19 | 0.0115 | 0.0438 | 2.55 |
| UBL5 | ENSG00000198258 | 31.30 | 0.19 | 0.0078 | 0.0386 | 1.93 |
| UBLCP1 | ENSG00000164332 | 27.94 | 0.19 | 0.0119 | 0.0442 | 2.06 |
| UBN1 | ENSG00000118900 | 207.95 | 0.20 | 0.0059 | 0.0365 | 1.45 |
| UBOX5 | ENSG00000185019 | 42.50 | 0.26 | 0.0005 | 0.0342 | 1.38 |
| UBQLN1 | ENSG00000135018 | 212.71 | 0.20 | 0.0114 | 0.0437 | 1.91 |
| UBQLN2 | ENSG00000188021 | 61.31 | 0.18 | 0.0112 | 0.0434 | 1.91 |
| UBR2 | ENSG00000024048 | 306.38 | 0.21 | 0.0021 | 0.0342 | 1.26 |
| UBR3 | ENSG00000144357 | 199.37 | 0.18 | 0.0151 | 0.0487 | 1.50 |
| UBR4 | ENSG00000127481 | 1217.11 | 0.18 | 0.0151 | 0.0487 | 1.36 |
| UBR7 | ENSG00000012963 | 62.69 | 0.20 | 0.0044 | 0.0351 | 1.45 |
| UBTD2 | ENSG00000168246 | 53.74 | 0.20 | 0.0057 | 0.0363 | 1.66 |
| UBTF | ENSG00000108312 | 248.09 | 0.18 | 0.0109 | 0.0431 | 1.27 |
| UBXN4 | ENSG00000144224 | 305.99 | 0.21 | 0.0038 | 0.0342 | 1.68 |
| UBXN6 | ENSG00000167671 | 100.38 | 0.19 | 0.0117 | 0.0439 | 1.47 |
| UBXN7 | ENSG00000163960 | 164.54 | 0.20 | 0.0037 | 0.0342 | 2.02 |
| UCHL5 | ENSG00000116750 | 44.17 | 0.21 | 0.0067 | 0.0373 | 2.08 |
| UCK1 | ENSG00000130717 | 34.22 | 0.23 | 0.0016 | 0.0342 | 1.43 |
| UCK2 | ENSG00000143179 | 132.06 | 0.25 | 0.0008 | 0.0342 | 1.72 |
| UEVLD | ENSG00000151116 | 48.06 | 0.24 | 0.0011 | 0.0342 | 1.39 |
| UFC1 | ENSG00000143222 | 67.28 | 0.19 | 0.0084 | 0.0397 | 1.87 |
| UFL1 | ENSG00000014123 | 77.93 | 0.21 | 0.0045 | 0.0352 | 1.65 |
| UFSP2 | ENSG00000109775 | 33.85 | 0.20 | 0.0062 | 0.0368 | 1.29 |
| UGGT1 | ENSG00000136731 | 348.08 | 0.20 | 0.0064 | 0.0370 | 1.87 |
| UGP2 | ENSG00000169764 | 297.23 | 0.21 | 0.0036 | 0.0342 | 0.76 |
| UGT8 | ENSG00000174607 | 112.08 | 0.22 | 0.0056 | 0.0363 | 1.41 |
| UHMK1 | ENSG00000152332 | 326.17 | 0.24 | 0.0014 | 0.0342 | 1.79 |
| UHRF1BP1 | ENSG00000065060 | 159.50 | 0.19 | 0.0104 | 0.0424 | 1.66 |
| UIMC1 | ENSG00000087206 | 81.78 | 0.20 | 0.0051 | 0.0358 | 1.46 |
| ULK1 | ENSG00000177169 | 135.88 | 0.19 | 0.0132 | 0.0460 | 1.48 |
| ULK3 | ENSG00000140474 | 127.53 | 0.23 | 0.0024 | 0.0342 | 0.93 |
| UMPS | ENSG00000114491 | 34.77 | 0.18 | 0.0125 | 0.0451 | 2.28 |
| UNC119 | ENSG00000109103 | 45.03 | 0.25 | 0.0011 | 0.0342 | 1.87 |
| UNC119B | ENSG00000175970 | 67.43 | 0.25 | 0.0009 | 0.0342 | 1.83 |
| UNC13B | ENSG00000198722 | 451.93 | 0.18 | 0.0144 | 0.0477 | 0.83 |
| UNC45A | ENSG00000140553 | 75.32 | 0.18 | 0.0129 | 0.0457 | 1.51 |
| UNC50 | ENSG00000115446 | 37.06 | 0.22 | 0.0053 | 0.0361 | 1.45 |
| UNC5CL | ENSG00000124602 | 20.96 | 0.23 | 0.0031 | 0.0342 | 2.95 |
| UNC93B1 | ENSG00000110057 | 48.40 | 0.22 | 0.0041 | 0.0349 | 1.36 |
| UNK | ENSG00000132478 | 75.46 | 0.21 | 0.0051 | 0.0358 | 1.41 |
| UNKL | ENSG00000059145 | 94.71 | 0.18 | 0.0151 | 0.0487 | 1.43 |
| UPF1 | ENSG00000005007 | 249.77 | 0.18 | 0.0143 | 0.0476 | 1.58 |
| UPF3A | ENSG00000169062 | 35.55 | 0.21 | 0.0049 | 0.0355 | 2.15 |
| UPK3B | ENSG00000243566 | 21.75 | 0.23 | 0.0037 | 0.0342 | 1.44 |
| UPK3BL | ENSG00000205236 | 84.46 | 0.18 | 0.0155 | 0.0492 | 1.36 |
| UQCR10 | ENSG00000184076 | 36.30 | 0.18 | 0.0104 | 0.0424 | 1.12 |
| UQCRC1 | ENSG00000010256 | 166.04 | 0.21 | 0.0038 | 0.0342 | 1.24 |
| UQCRC2 | ENSG00000140740 | 173.47 | 0.23 | 0.0021 | 0.0342 | 1.51 |
| UQCRFS1 | ENSG00000169021 | 63.02 | 0.18 | 0.0105 | 0.0426 | 1.25 |
| UQCRH | ENSG00000173660 | 85.28 | 0.18 | 0.0114 | 0.0437 | 1.65 |
| UQCRQ | ENSG00000164405 | 50.16 | 0.19 | 0.0081 | 0.0391 | 1.44 |
| URGCP | ENSG00000106608 | 202.82 | 0.22 | 0.0037 | 0.0342 | 1.24 |
| URM1 | ENSG00000167118 | 50.56 | 0.19 | 0.0105 | 0.0426 | 1.77 |
| UROD | ENSG00000126088 | 27.20 | 0.22 | 0.0037 | 0.0342 | 1.31 |
| USH1C | ENSG00000006611 | 252.60 | 0.24 | 0.0008 | 0.0342 | 1.43 |
| USMG5 | ENSG00000173915 | 34.62 | 0.21 | 0.0073 | 0.0379 | 1.56 |
| USO1 | ENSG00000138768 | 194.24 | 0.19 | 0.0076 | 0.0382 | 1.55 |
| USP13 | ENSG00000058056 | 39.29 | 0.18 | 0.0158 | 0.0497 | 1.65 |
| USP19 | ENSG00000172046 | 151.71 | 0.19 | 0.0101 | 0.0421 | 1.34 |
| USP20 | ENSG00000136878 | 80.61 | 0.20 | 0.0080 | 0.0389 | 1.34 |
| USP21 | ENSG00000143258 | 42.83 | 0.20 | 0.0086 | 0.0399 | 1.77 |
| USP22 | ENSG00000124422 | 401.65 | 0.20 | 0.0046 | 0.0352 | 1.62 |
| USP25 | ENSG00000155313 | 152.97 | 0.21 | 0.0049 | 0.0355 | 1.54 |
| USP28 | ENSG00000048028 | 61.37 | 0.20 | 0.0034 | 0.0342 | 1.60 |
| USP3 | ENSG00000140455 | 100.68 | 0.18 | 0.0119 | 0.0442 | 1.37 |
| USP30 | ENSG00000135093 | 53.80 | 0.26 | 0.0003 | 0.0342 | 1.27 |
| USP32 | ENSG00000170832 | 102.13 | 0.22 | 0.0026 | 0.0342 | 1.73 |
| USP33 | ENSG00000077254 | 149.26 | 0.18 | 0.0102 | 0.0422 | 1.36 |
| USP37 | ENSG00000135913 | 140.26 | 0.21 | 0.0044 | 0.0351 | 1.70 |
| USP38 | ENSG00000170185 | 111.23 | 0.22 | 0.0026 | 0.0342 | 1.30 |
| USP40 | ENSG00000085982 | 142.42 | 0.26 | 0.0005 | 0.0342 | 1.67 |
| USP5 | ENSG00000111667 | 70.94 | 0.17 | 0.0160 | 0.0499 | 2.07 |
| USP54 | ENSG00000166348 | 391.99 | 0.24 | 0.0012 | 0.0342 | 1.22 |
| USP6NL | ENSG00000148429 | 78.51 | 0.19 | 0.0098 | 0.0416 | 2.44 |
| USP7 | ENSG00000187555 | 392.90 | 0.18 | 0.0087 | 0.0401 | 2.08 |
| USP9X | ENSG00000124486 | 574.57 | 0.25 | 0.0014 | 0.0342 | 2.15 |
| UTP14A | ENSG00000156697 | 36.18 | 0.20 | 0.0068 | 0.0374 | 3.52 |
| UTP14C | ENSG00000253797 | 130.01 | 0.23 | 0.0019 | 0.0342 | 1.88 |
| UTP3 | ENSG00000132467 | 33.12 | 0.18 | 0.0105 | 0.0426 | 1.80 |
| UTP6 | ENSG00000108651 | 57.35 | 0.23 | 0.0023 | 0.0342 | 1.98 |
| VAC14 | ENSG00000103043 | 80.80 | 0.22 | 0.0029 | 0.0342 | 1.83 |
| VAMP3 | ENSG00000049245 | 122.85 | 0.23 | 0.0032 | 0.0342 | 1.52 |
| VAMP7 | ENSG00000124333 | 18.75 | 0.19 | 0.0109 | 0.0431 | 1.83 |
| VAMP8 | ENSG00000118640 | 72.30 | 0.20 | 0.0072 | 0.0377 | 1.09 |
| VANGL1 | ENSG00000173218 | 96.42 | 0.22 | 0.0028 | 0.0342 | 1.68 |
| VANGL2 | ENSG00000162738 | 64.76 | 0.20 | 0.0092 | 0.0408 | 1.40 |
| VAPB | ENSG00000124164 | 157.97 | 0.22 | 0.0032 | 0.0342 | 2.40 |
| VARS | ENSG00000204394 | 100.00 | 0.22 | 0.0036 | 0.0342 | 2.62 |
| VARS2 | ENSG00000137411 | 89.35 | 0.23 | 0.0017 | 0.0342 | 1.80 |
| VASH1 | ENSG00000071246 | 93.24 | 0.18 | 0.0153 | 0.0489 | 1.60 |
| VAV2 | ENSG00000160293 | 118.29 | 0.21 | 0.0044 | 0.0351 | 1.96 |
| VAV3 | ENSG00000134215 | 178.90 | 0.22 | 0.0035 | 0.0342 | 1.60 |
| VBP1 | ENSG00000155959 | 24.38 | 0.21 | 0.0081 | 0.0391 | 2.79 |
| VCP | ENSG00000165280 | 304.66 | 0.20 | 0.0089 | 0.0403 | 2.00 |
| VCPIP1 | ENSG00000175073 | 166.45 | 0.22 | 0.0033 | 0.0342 | 1.74 |
| VDAC1 | ENSG00000213585 | 173.80 | 0.20 | 0.0051 | 0.0358 | 2.84 |
| VDAC3 | ENSG00000078668 | 79.92 | 0.22 | 0.0026 | 0.0342 | 1.94 |
| VDR | ENSG00000111424 | 300.22 | 0.25 | 0.0008 | 0.0342 | 0.98 |
| VEZF1 | ENSG00000136451 | 124.43 | 0.19 | 0.0092 | 0.0408 | 1.70 |
| VEZT | ENSG00000028203 | 113.62 | 0.18 | 0.0120 | 0.0444 | 1.84 |
| VGLL4 | ENSG00000144560 | 153.15 | 0.18 | 0.0140 | 0.0471 | 1.82 |
| VHL | ENSG00000134086 | 123.99 | 0.22 | 0.0023 | 0.0342 | 1.71 |
| VIL1 | ENSG00000127831 | 419.90 | 0.24 | 0.0011 | 0.0342 | 2.05 |
| VILL | ENSG00000136059 | 209.43 | 0.23 | 0.0031 | 0.0342 | 0.66 |
| VIPR1 | ENSG00000114812 | 418.69 | 0.27 | 0.0005 | 0.0342 | 0.48 |
| VKORC1L1 | ENSG00000196715 | 104.43 | 0.20 | 0.0059 | 0.0365 | 2.35 |
| VMA21 | ENSG00000160131 | 62.81 | 0.21 | 0.0068 | 0.0374 | 3.08 |
| VNN1 | ENSG00000112299 | 14.42 | 0.31 | 0.0012 | 0.0342 | 1.13 |
| VPRBP | ENSG00000145041 | 129.08 | 0.19 | 0.0105 | 0.0426 | 1.61 |
| VPS11 | ENSG00000160695 | 57.32 | 0.19 | 0.0086 | 0.0399 | 1.44 |
| VPS13A | ENSG00000197969 | 673.38 | 0.25 | 0.0004 | 0.0342 | 1.13 |
| VPS13C | ENSG00000129003 | 786.33 | 0.19 | 0.0100 | 0.0419 | 1.68 |
| VPS13D | ENSG00000048707 | 1051.54 | 0.19 | 0.0108 | 0.0429 | 0.84 |
| VPS18 | ENSG00000104142 | 39.60 | 0.21 | 0.0071 | 0.0376 | 1.51 |
| VPS25 | ENSG00000131475 | 42.17 | 0.20 | 0.0063 | 0.0370 | 1.85 |
| VPS26B | ENSG00000151502 | 122.92 | 0.26 | 0.0003 | 0.0342 | 1.46 |
| VPS28 | ENSG00000160948 | 75.29 | 0.24 | 0.0014 | 0.0342 | 1.78 |
| VPS35 | ENSG00000069329 | 148.79 | 0.19 | 0.0103 | 0.0423 | 2.33 |
| VPS36 | ENSG00000136100 | 228.59 | 0.23 | 0.0016 | 0.0342 | 1.79 |
| VPS37A | ENSG00000155975 | 89.70 | 0.20 | 0.0065 | 0.0371 | 1.22 |
| VPS37C | ENSG00000167987 | 81.78 | 0.19 | 0.0115 | 0.0438 | 1.23 |
| VPS39 | ENSG00000166887 | 164.24 | 0.21 | 0.0050 | 0.0356 | 1.20 |
| VPS45 | ENSG00000136631 | 63.82 | 0.20 | 0.0088 | 0.0402 | 1.21 |
| VPS4A | ENSG00000132612 | 127.26 | 0.22 | 0.0037 | 0.0342 | 1.62 |
| VPS4B | ENSG00000119541 | 104.81 | 0.21 | 0.0033 | 0.0342 | 1.11 |
| VPS52 | ENSG00000223501 | 114.67 | 0.22 | 0.0034 | 0.0342 | 1.31 |
| VPS54 | ENSG00000143952 | 104.97 | 0.20 | 0.0056 | 0.0363 | 1.67 |
| VPS72 | ENSG00000163159 | 35.76 | 0.20 | 0.0084 | 0.0397 | 1.73 |
| VPS8 | ENSG00000156931 | 191.44 | 0.18 | 0.0136 | 0.0466 | 1.42 |
| VRK3 | ENSG00000105053 | 56.57 | 0.18 | 0.0143 | 0.0476 | 1.21 |
| VSIG10 | ENSG00000176834 | 210.07 | 0.26 | 0.0004 | 0.0342 | 1.28 |
| VSIG2 | ENSG00000019102 | 170.19 | 0.21 | 0.0041 | 0.0349 | 0.17 |
| VSTM5 | ENSG00000214376 | 17.92 | 0.18 | 0.0158 | 0.0497 | 0.59 |
| VTA1 | ENSG00000009844 | 48.91 | 0.20 | 0.0069 | 0.0375 | 1.89 |
| VTI1B | ENSG00000100568 | 64.22 | 0.25 | 0.0006 | 0.0342 | 1.34 |
| VWA2 | ENSG00000165816 | 16.57 | 0.25 | 0.0019 | 0.0342 | 8.01 |
| VWA5A | ENSG00000110002 | 117.85 | 0.22 | 0.0029 | 0.0342 | 0.76 |
| WAC | ENSG00000095787 | 335.14 | 0.19 | 0.0094 | 0.0411 | 1.51 |
| WAPAL | ENSG00000062650 | 214.02 | 0.20 | 0.0066 | 0.0373 | 1.55 |
| WASL | ENSG00000106299 | 238.41 | 0.23 | 0.0022 | 0.0342 | 1.19 |
| WBP1 | ENSG00000239779 | 64.51 | 0.19 | 0.0125 | 0.0451 | 1.14 |
| WBP11 | ENSG00000084463 | 107.77 | 0.18 | 0.0139 | 0.0469 | 2.00 |
| WBP4 | ENSG00000120688 | 26.98 | 0.20 | 0.0080 | 0.0389 | 2.10 |
| WBSCR16 | ENSG00000174374 | 30.98 | 0.22 | 0.0033 | 0.0342 | 1.83 |
| WDFY1 | ENSG00000085449 | 212.97 | 0.23 | 0.0020 | 0.0342 | 1.69 |
| WDFY3 | ENSG00000163625 | 342.74 | 0.21 | 0.0046 | 0.0352 | 1.36 |
| WDR11 | ENSG00000120008 | 144.43 | 0.19 | 0.0083 | 0.0395 | 1.44 |
| WDR12 | ENSG00000138442 | 28.13 | 0.19 | 0.0132 | 0.0460 | 2.83 |
| WDR13 | ENSG00000101940 | 106.05 | 0.18 | 0.0144 | 0.0477 | 1.40 |
| WDR24 | ENSG00000127580 | 43.23 | 0.21 | 0.0037 | 0.0342 | 1.80 |
| WDR26 | ENSG00000162923 | 372.17 | 0.19 | 0.0089 | 0.0403 | 1.31 |
| WDR27 | ENSG00000184465 | 100.45 | 0.20 | 0.0087 | 0.0401 | 1.61 |
| WDR3 | ENSG00000065183 | 46.62 | 0.20 | 0.0075 | 0.0382 | 3.06 |
| WDR34 | ENSG00000119333 | 45.23 | 0.22 | 0.0063 | 0.0370 | 2.56 |
| WDR45L | ENSG00000141580 | 134.13 | 0.21 | 0.0053 | 0.0361 | 1.98 |
| WDR5 | ENSG00000196363 | 70.33 | 0.22 | 0.0043 | 0.0351 | 2.37 |
| WDR52 | ENSG00000206530 | 192.55 | 0.23 | 0.0016 | 0.0342 | 1.06 |
| WDR55 | ENSG00000120314 | 161.27 | 0.17 | 0.0147 | 0.0482 | 1.18 |
| WDR59 | ENSG00000103091 | 89.07 | 0.20 | 0.0069 | 0.0375 | 1.77 |
| WDR60 | ENSG00000126870 | 95.01 | 0.21 | 0.0041 | 0.0349 | 1.50 |
| WDR73 | ENSG00000177082 | 49.49 | 0.19 | 0.0080 | 0.0389 | 1.36 |
| WDR81 | ENSG00000167716 | 119.93 | 0.23 | 0.0038 | 0.0342 | 1.37 |
| WDR83 | ENSG00000123154 | 25.05 | 0.18 | 0.0126 | 0.0452 | 1.62 |
| WDR85 | ENSG00000148399 | 69.62 | 0.19 | 0.0144 | 0.0477 | 1.67 |
| WDR86 | ENSG00000187260 | 20.63 | 0.23 | 0.0029 | 0.0342 | 1.56 |
| WDR90 | ENSG00000161996 | 111.49 | 0.21 | 0.0071 | 0.0376 | 2.82 |
| WDSUB1 | ENSG00000196151 | 18.73 | 0.23 | 0.0012 | 0.0342 | 1.83 |
| WDTC1 | ENSG00000142784 | 150.94 | 0.20 | 0.0084 | 0.0397 | 1.23 |
| WFDC1 | ENSG00000103175 | 20.90 | 0.19 | 0.0073 | 0.0379 | 1.08 |
| WHAMM | ENSG00000156232 | 47.57 | 0.20 | 0.0045 | 0.0352 | 1.25 |
| WHSC1 | ENSG00000109685 | 477.41 | 0.20 | 0.0094 | 0.0411 | 1.55 |
| WHSC2 | ENSG00000185049 | 67.45 | 0.19 | 0.0125 | 0.0451 | 1.69 |
| WIPF2 | ENSG00000171475 | 213.05 | 0.18 | 0.0144 | 0.0477 | 1.22 |
| WIPI2 | ENSG00000157954 | 107.94 | 0.19 | 0.0070 | 0.0376 | 1.94 |
| WNK1 | ENSG00000060237 | 692.00 | 0.18 | 0.0135 | 0.0464 | 1.77 |
| WNK2 | ENSG00000165238 | 1066.80 | 0.22 | 0.0024 | 0.0342 | 0.99 |
| WNK4 | ENSG00000126562 | 205.51 | 0.19 | 0.0091 | 0.0407 | 0.92 |
| WRAP73 | ENSG00000116213 | 50.69 | 0.18 | 0.0150 | 0.0486 | 1.41 |
| WSB2 | ENSG00000176871 | 148.09 | 0.20 | 0.0062 | 0.0368 | 1.46 |
| WWC1 | ENSG00000113645 | 95.86 | 0.22 | 0.0026 | 0.0342 | 1.69 |
| WWP1 | ENSG00000123124 | 188.13 | 0.22 | 0.0039 | 0.0345 | 1.70 |
| WWP2 | ENSG00000198373 | 199.77 | 0.22 | 0.0020 | 0.0342 | 1.08 |
| XBP1 | ENSG00000100219 | 625.19 | 0.19 | 0.0100 | 0.0419 | 1.00 |
| XDH | ENSG00000158125 | 166.05 | 0.21 | 0.0052 | 0.0359 | 0.50 |
| XIAP | ENSG00000101966 | 258.47 | 0.22 | 0.0037 | 0.0342 | 1.65 |
| XK | ENSG00000047597 | 103.21 | 0.21 | 0.0051 | 0.0358 | 1.13 |
| XKR6 | ENSG00000171044 | 24.62 | 0.26 | 0.0014 | 0.0342 | 1.01 |
| XKR8 | ENSG00000158156 | 26.69 | 0.21 | 0.0045 | 0.0352 | 0.90 |
| XKR9 | ENSG00000221947 | 10.63 | 0.23 | 0.0068 | 0.0374 | 1.40 |
| XPNPEP1 | ENSG00000108039 | 226.81 | 0.20 | 0.0059 | 0.0365 | 1.10 |
| XPO1 | ENSG00000082898 | 380.32 | 0.22 | 0.0030 | 0.0342 | 2.19 |
| XPO7 | ENSG00000130227 | 162.10 | 0.18 | 0.0115 | 0.0438 | 1.50 |
| XPOT | ENSG00000184575 | 104.94 | 0.25 | 0.0015 | 0.0342 | 3.52 |
| XPR1 | ENSG00000143324 | 102.83 | 0.24 | 0.0005 | 0.0342 | 1.36 |
| XRCC5 | ENSG00000079246 | 321.69 | 0.19 | 0.0109 | 0.0431 | 2.23 |
| XRCC6 | ENSG00000196419 | 146.33 | 0.18 | 0.0130 | 0.0458 | 2.05 |
| XRN1 | ENSG00000114127 | 334.88 | 0.20 | 0.0077 | 0.0384 | 1.20 |
| XRN2 | ENSG00000088930 | 114.44 | 0.18 | 0.0115 | 0.0438 | 3.05 |
| XXbac-BPG116M5.17 | ENSG00000244255 | 75.95 | 0.19 | 0.0137 | 0.0467 | 3.41 |
| XYLB | ENSG00000093217 | 58.27 | 0.22 | 0.0022 | 0.0342 | 1.46 |
| YAP1 | ENSG00000137693 | 202.40 | 0.21 | 0.0040 | 0.0346 | 2.55 |
| YARS2 | ENSG00000139131 | 22.17 | 0.22 | 0.0023 | 0.0342 | 1.74 |
| YBEY | ENSG00000182362 | 16.44 | 0.20 | 0.0068 | 0.0374 | 1.26 |
| YEATS2 | ENSG00000163872 | 148.35 | 0.21 | 0.0046 | 0.0352 | 2.32 |
| YES1 | ENSG00000176105 | 157.99 | 0.21 | 0.0032 | 0.0342 | 1.50 |
| YIF1A | ENSG00000174851 | 39.85 | 0.26 | 0.0007 | 0.0342 | 1.55 |
| YIF1B | ENSG00000167645 | 80.02 | 0.22 | 0.0020 | 0.0342 | 1.04 |
| YIPF1 | ENSG00000058799 | 35.47 | 0.21 | 0.0049 | 0.0355 | 1.18 |
| YIPF2 | ENSG00000130733 | 51.64 | 0.19 | 0.0105 | 0.0426 | 1.70 |
| YIPF3 | ENSG00000137207 | 100.98 | 0.19 | 0.0083 | 0.0395 | 1.46 |
| YIPF4 | ENSG00000119820 | 157.43 | 0.22 | 0.0024 | 0.0342 | 1.58 |
| YIPF5 | ENSG00000145817 | 46.80 | 0.26 | 0.0004 | 0.0342 | 1.47 |
| YIPF6 | ENSG00000181704 | 135.33 | 0.18 | 0.0131 | 0.0460 | 1.76 |
| YKT6 | ENSG00000106636 | 102.51 | 0.23 | 0.0019 | 0.0342 | 1.98 |
| YLPM1 | ENSG00000119596 | 316.89 | 0.19 | 0.0112 | 0.0434 | 1.28 |
| YME1L1 | ENSG00000136758 | 224.25 | 0.21 | 0.0041 | 0.0349 | 1.81 |
| YPEL1 | ENSG00000100027 | 57.03 | 0.21 | 0.0044 | 0.0351 | 1.11 |
| YPEL2 | ENSG00000175155 | 114.25 | 0.26 | 0.0004 | 0.0342 | 1.25 |
| YTHDF1 | ENSG00000149658 | 103.71 | 0.19 | 0.0087 | 0.0401 | 2.60 |
| YTHDF2 | ENSG00000198492 | 104.16 | 0.18 | 0.0157 | 0.0496 | 1.56 |
| YWHAB | ENSG00000166913 | 387.50 | 0.18 | 0.0153 | 0.0489 | 2.55 |
| YWHAE | ENSG00000108953 | 249.46 | 0.20 | 0.0073 | 0.0379 | 1.99 |
| YWHAG | ENSG00000170027 | 215.16 | 0.20 | 0.0093 | 0.0409 | 2.73 |
| YWHAH | ENSG00000128245 | 124.43 | 0.21 | 0.0062 | 0.0368 | 2.03 |
| YWHAQ | ENSG00000134308 | 135.90 | 0.19 | 0.0103 | 0.0423 | 2.35 |
| YY1 | ENSG00000100811 | 196.19 | 0.21 | 0.0025 | 0.0342 | 1.59 |
| Z84479.1 | ENSG00000130731 | 20.45 | 0.20 | 0.0106 | 0.0427 | 3.12 |
| ZACN | ENSG00000186919 | 71.18 | 0.18 | 0.0159 | 0.0498 | 1.29 |
| ZADH2 | ENSG00000180011 | 157.47 | 0.20 | 0.0063 | 0.0370 | 1.00 |
| ZBED4 | ENSG00000100426 | 118.69 | 0.19 | 0.0099 | 0.0417 | 1.60 |
| ZBTB22 | ENSG00000236104 | 47.11 | 0.22 | 0.0026 | 0.0342 | 1.05 |
| ZBTB3 | ENSG00000185670 | 23.11 | 0.24 | 0.0013 | 0.0342 | 1.36 |
| ZBTB33 | ENSG00000177485 | 50.67 | 0.22 | 0.0067 | 0.0373 | 2.93 |
| ZBTB34 | ENSG00000177125 | 60.35 | 0.24 | 0.0013 | 0.0342 | 1.47 |
| ZBTB38 | ENSG00000177311 | 494.45 | 0.21 | 0.0030 | 0.0342 | 1.65 |
| ZBTB40 | ENSG00000184677 | 211.55 | 0.18 | 0.0143 | 0.0476 | 1.12 |
| ZBTB41 | ENSG00000177888 | 34.13 | 0.21 | 0.0068 | 0.0374 | 2.12 |
| ZBTB42 | ENSG00000179627 | 27.70 | 0.19 | 0.0112 | 0.0434 | 1.39 |
| ZBTB48 | ENSG00000204859 | 74.30 | 0.22 | 0.0022 | 0.0342 | 0.87 |
| ZBTB7B | ENSG00000160685 | 310.79 | 0.22 | 0.0038 | 0.0342 | 0.76 |
| ZBTB7C | ENSG00000184828 | 144.80 | 0.19 | 0.0082 | 0.0393 | 0.34 |
| ZBTB8OS | ENSG00000176261 | 50.49 | 0.19 | 0.0087 | 0.0401 | 1.25 |
| ZBTB9 | ENSG00000213588 | 13.95 | 0.18 | 0.0144 | 0.0477 | 2.58 |
| ZC3H11A | ENSG00000058673 | 509.92 | 0.20 | 0.0061 | 0.0368 | 1.54 |
| ZC3H13 | ENSG00000123200 | 215.66 | 0.20 | 0.0064 | 0.0370 | 2.08 |
| ZC3H14 | ENSG00000100722 | 147.35 | 0.20 | 0.0059 | 0.0365 | 1.37 |
| ZC3H15 | ENSG00000065548 | 86.01 | 0.22 | 0.0038 | 0.0342 | 2.68 |
| ZC3H4 | ENSG00000130749 | 177.57 | 0.19 | 0.0101 | 0.0421 | 1.68 |
| ZC3H6 | ENSG00000188177 | 98.33 | 0.21 | 0.0037 | 0.0342 | 1.27 |
| ZC3H7B | ENSG00000100403 | 306.43 | 0.20 | 0.0075 | 0.0382 | 1.53 |
| ZC3HAV1L | ENSG00000146858 | 19.00 | 0.20 | 0.0085 | 0.0399 | 3.25 |
| ZCCHC10 | ENSG00000155329 | 30.21 | 0.22 | 0.0035 | 0.0342 | 1.26 |
| ZCCHC14 | ENSG00000140948 | 158.99 | 0.21 | 0.0044 | 0.0351 | 1.96 |
| ZCCHC2 | ENSG00000141664 | 101.51 | 0.19 | 0.0125 | 0.0451 | 0.91 |
| ZCCHC3 | ENSG00000177764 | 41.06 | 0.20 | 0.0092 | 0.0408 | 1.75 |
| ZCCHC6 | ENSG00000083223 | 206.99 | 0.18 | 0.0107 | 0.0428 | 1.39 |
| ZCCHC8 | ENSG00000033030 | 77.62 | 0.19 | 0.0105 | 0.0426 | 1.26 |
| ZCRB1 | ENSG00000139168 | 27.02 | 0.21 | 0.0040 | 0.0346 | 2.34 |
| ZDHHC12 | ENSG00000160446 | 46.84 | 0.24 | 0.0013 | 0.0342 | 1.28 |
| ZDHHC20 | ENSG00000180776 | 46.92 | 0.26 | 0.0009 | 0.0342 | 3.13 |
| ZDHHC21 | ENSG00000175893 | 88.78 | 0.18 | 0.0158 | 0.0497 | 1.43 |
| ZDHHC23 | ENSG00000184307 | 181.36 | 0.20 | 0.0061 | 0.0368 | 1.04 |
| ZDHHC24 | ENSG00000174165 | 29.61 | 0.21 | 0.0050 | 0.0356 | 1.51 |
| ZDHHC3 | ENSG00000163812 | 312.82 | 0.21 | 0.0038 | 0.0342 | 1.38 |
| ZDHHC5 | ENSG00000156599 | 276.15 | 0.19 | 0.0071 | 0.0376 | 1.46 |
| ZDHHC6 | ENSG00000023041 | 76.39 | 0.21 | 0.0048 | 0.0354 | 1.61 |
| ZDHHC7 | ENSG00000153786 | 223.75 | 0.26 | 0.0002 | 0.0342 | 1.13 |
| ZDHHC9 | ENSG00000188706 | 104.97 | 0.23 | 0.0015 | 0.0342 | 3.50 |
| ZER1 | ENSG00000160445 | 123.93 | 0.23 | 0.0023 | 0.0342 | 0.96 |
| ZFAND1 | ENSG00000104231 | 51.73 | 0.21 | 0.0056 | 0.0363 | 2.38 |
| ZFAND3 | ENSG00000156639 | 131.05 | 0.22 | 0.0030 | 0.0342 | 1.43 |
| ZFHX2 | ENSG00000136367 | 35.59 | 0.21 | 0.0058 | 0.0364 | 1.64 |
| ZFP106 | ENSG00000103994 | 333.17 | 0.21 | 0.0040 | 0.0346 | 1.26 |
| ZFP161 | ENSG00000198081 | 28.28 | 0.20 | 0.0069 | 0.0375 | 1.38 |
| ZFP41 | ENSG00000181638 | 84.45 | 0.25 | 0.0009 | 0.0342 | 1.79 |
| ZFP62 | ENSG00000196670 | 119.27 | 0.21 | 0.0058 | 0.0364 | 1.52 |
| ZFP64 | ENSG00000020256 | 48.76 | 0.26 | 0.0002 | 0.0342 | 2.20 |
| ZFP91 | ENSG00000186660 | 309.31 | 0.21 | 0.0067 | 0.0373 | 1.22 |
| ZFP91-CNTF | ENSG00000255073 | 89.20 | 0.19 | 0.0083 | 0.0395 | 1.22 |
| ZFPL1 | ENSG00000162300 | 44.83 | 0.21 | 0.0071 | 0.0376 | 1.19 |
| ZFYVE1 | ENSG00000165861 | 53.60 | 0.19 | 0.0080 | 0.0389 | 0.97 |
| ZFYVE19 | ENSG00000166140 | 46.88 | 0.21 | 0.0059 | 0.0365 | 1.09 |
| ZFYVE21 | ENSG00000100711 | 42.11 | 0.25 | 0.0004 | 0.0342 | 1.17 |
| ZFYVE26 | ENSG00000072121 | 200.97 | 0.20 | 0.0084 | 0.0397 | 1.26 |
| ZFYVE27 | ENSG00000155256 | 131.80 | 0.22 | 0.0034 | 0.0342 | 1.22 |
| ZFYVE28 | ENSG00000159733 | 101.54 | 0.22 | 0.0037 | 0.0342 | 0.61 |
| ZFYVE9 | ENSG00000157077 | 115.82 | 0.19 | 0.0095 | 0.0413 | 1.07 |
| ZG16B | ENSG00000162078 | 24.90 | 0.18 | 0.0161 | 0.0500 | 1.09 |
| ZGLP1 | ENSG00000220201 | 6.09 | 0.18 | 0.0152 | 0.0489 | 1.42 |
| ZHX1 | ENSG00000165156 | 93.53 | 0.21 | 0.0027 | 0.0342 | 1.43 |
| ZKSCAN1 | ENSG00000106261 | 465.78 | 0.25 | 0.0005 | 0.0342 | 1.82 |
| ZKSCAN3 | ENSG00000189298 | 25.24 | 0.24 | 0.0017 | 0.0342 | 1.44 |
| ZKSCAN5 | ENSG00000196652 | 40.70 | 0.19 | 0.0095 | 0.0413 | 1.69 |
| ZMAT3 | ENSG00000172667 | 83.34 | 0.25 | 0.0018 | 0.0342 | 2.16 |
| ZMIZ1 | ENSG00000108175 | 448.84 | 0.18 | 0.0149 | 0.0485 | 1.43 |
| ZMYM1 | ENSG00000197056 | 37.52 | 0.24 | 0.0016 | 0.0342 | 1.65 |
| ZMYM3 | ENSG00000147130 | 76.93 | 0.26 | 0.0007 | 0.0342 | 1.92 |
| ZMYM6 | ENSG00000163867 | 117.46 | 0.18 | 0.0143 | 0.0476 | 1.10 |
| ZMYM6NB | ENSG00000243749 | 13.02 | 0.18 | 0.0122 | 0.0446 | 1.41 |
| ZMYND11 | ENSG00000015171 | 142.94 | 0.19 | 0.0088 | 0.0402 | 1.55 |
| ZMYND12 | ENSG00000066185 | 4.11 | 0.22 | 0.0105 | 0.0426 | 0.70 |
| ZMYND19 | ENSG00000165724 | 24.46 | 0.22 | 0.0055 | 0.0363 | 2.29 |
| ZMYND8 | ENSG00000101040 | 230.53 | 0.26 | 0.0003 | 0.0342 | 2.40 |
| ZNF12 | ENSG00000164631 | 80.65 | 0.18 | 0.0134 | 0.0463 | 2.03 |
| ZNF133 | ENSG00000125846 | 46.89 | 0.18 | 0.0158 | 0.0497 | 1.64 |
| ZNF142 | ENSG00000115568 | 119.55 | 0.24 | 0.0010 | 0.0342 | 1.67 |
| ZNF146 | ENSG00000167635 | 133.40 | 0.19 | 0.0096 | 0.0414 | 2.37 |
| ZNF148 | ENSG00000163848 | 171.87 | 0.19 | 0.0086 | 0.0399 | 1.55 |
| ZNF160 | ENSG00000170949 | 100.25 | 0.20 | 0.0058 | 0.0364 | 1.37 |
| ZNF169 | ENSG00000175787 | 38.32 | 0.23 | 0.0025 | 0.0342 | 1.14 |
| ZNF174 | ENSG00000103343 | 13.77 | 0.22 | 0.0043 | 0.0351 | 1.72 |
| ZNF175 | ENSG00000105497 | 27.54 | 0.22 | 0.0028 | 0.0342 | 0.97 |
| ZNF177 | ENSG00000188629 | 16.99 | 0.23 | 0.0031 | 0.0342 | 1.40 |
| ZNF18 | ENSG00000154957 | 26.51 | 0.19 | 0.0087 | 0.0401 | 1.05 |
| ZNF181 | ENSG00000197841 | 24.72 | 0.25 | 0.0005 | 0.0342 | 1.38 |
| ZNF185 | ENSG00000147394 | 23.11 | 0.19 | 0.0113 | 0.0435 | 2.92 |
| ZNF189 | ENSG00000136870 | 64.30 | 0.23 | 0.0034 | 0.0342 | 1.62 |
| ZNF193 | ENSG00000137185 | 41.16 | 0.20 | 0.0083 | 0.0395 | 1.14 |
| ZNF195 | ENSG00000005801 | 89.33 | 0.22 | 0.0046 | 0.0352 | 1.86 |
| ZNF197 | ENSG00000186448 | 64.91 | 0.19 | 0.0065 | 0.0371 | 1.36 |
| ZNF200 | ENSG00000010539 | 15.59 | 0.19 | 0.0138 | 0.0468 | 2.55 |
| ZNF205 | ENSG00000122386 | 19.76 | 0.23 | 0.0043 | 0.0351 | 1.94 |
| ZNF212 | ENSG00000170260 | 26.92 | 0.21 | 0.0059 | 0.0365 | 1.66 |
| ZNF213 | ENSG00000085644 | 31.57 | 0.20 | 0.0093 | 0.0409 | 1.47 |
| ZNF217 | ENSG00000171940 | 229.72 | 0.19 | 0.0096 | 0.0414 | 1.79 |
| ZNF219 | ENSG00000165804 | 84.31 | 0.19 | 0.0140 | 0.0471 | 1.19 |
| ZNF221 | ENSG00000159905 | 10.71 | 0.19 | 0.0141 | 0.0472 | 1.21 |
| ZNF223 | ENSG00000178386 | 17.11 | 0.23 | 0.0027 | 0.0342 | 1.07 |
| ZNF224 | ENSG00000186019 | 60.50 | 0.21 | 0.0039 | 0.0345 | 1.04 |
| ZNF226 | ENSG00000167380 | 61.80 | 0.23 | 0.0022 | 0.0342 | 1.41 |
| ZNF230 | ENSG00000159882 | 10.39 | 0.22 | 0.0061 | 0.0368 | 1.21 |
| ZNF233 | ENSG00000159915 | 20.83 | 0.29 | 0.0018 | 0.0342 | 1.53 |
| ZNF235 | ENSG00000159917 | 35.05 | 0.21 | 0.0052 | 0.0359 | 1.16 |
| ZNF236 | ENSG00000130856 | 98.19 | 0.18 | 0.0152 | 0.0489 | 1.19 |
| ZNF24 | ENSG00000172466 | 190.62 | 0.18 | 0.0139 | 0.0469 | 1.21 |
| ZNF264 | ENSG00000083844 | 145.07 | 0.19 | 0.0061 | 0.0368 | 1.17 |
| ZNF266 | ENSG00000174652 | 107.91 | 0.21 | 0.0063 | 0.0370 | 1.11 |
| ZNF28 | ENSG00000198538 | 53.38 | 0.25 | 0.0005 | 0.0342 | 1.74 |
| ZNF282 | ENSG00000170265 | 75.23 | 0.20 | 0.0086 | 0.0399 | 1.76 |
| ZNF283 | ENSG00000167637 | 36.97 | 0.24 | 0.0010 | 0.0342 | 1.58 |
| ZNF292 | ENSG00000188994 | 336.99 | 0.18 | 0.0125 | 0.0451 | 1.41 |
| ZNF3 | ENSG00000166526 | 120.70 | 0.25 | 0.0019 | 0.0342 | 2.04 |
| ZNF311 | ENSG00000197935 | 10.41 | 0.23 | 0.0016 | 0.0342 | 1.50 |
| ZNF317 | ENSG00000130803 | 72.30 | 0.20 | 0.0074 | 0.0380 | 1.74 |
| ZNF320 | ENSG00000182986 | 98.26 | 0.30 | <.0001 | 0.0342 | 1.48 |
| ZNF321P | ENSG00000221874 | 70.78 | 0.21 | 0.0038 | 0.0342 | 1.23 |
| ZNF322 | ENSG00000181315 | 20.38 | 0.19 | 0.0118 | 0.0441 | 1.62 |
| ZNF323 | ENSG00000235109 | 24.01 | 0.19 | 0.0121 | 0.0445 | 1.47 |
| ZNF324 | ENSG00000083812 | 48.88 | 0.19 | 0.0138 | 0.0468 | 1.24 |
| ZNF326 | ENSG00000162664 | 78.25 | 0.22 | 0.0026 | 0.0342 | 1.37 |
| ZNF33B | ENSG00000196693 | 138.03 | 0.23 | 0.0021 | 0.0342 | 1.64 |
| ZNF350 | ENSG00000256683 | 27.27 | 0.19 | 0.0112 | 0.0434 | 1.12 |
| ZNF358 | ENSG00000198816 | 48.88 | 0.21 | 0.0050 | 0.0356 | 1.69 |
| ZNF383 | ENSG00000188283 | 15.53 | 0.20 | 0.0081 | 0.0391 | 1.34 |
| ZNF384 | ENSG00000126746 | 121.43 | 0.20 | 0.0062 | 0.0368 | 1.51 |
| ZNF395 | ENSG00000186918 | 192.93 | 0.20 | 0.0089 | 0.0403 | 1.35 |
| ZNF396 | ENSG00000186496 | 6.26 | 0.24 | 0.0021 | 0.0342 | 0.89 |
| ZNF397 | ENSG00000186812 | 105.02 | 0.19 | 0.0110 | 0.0432 | 1.01 |
| ZNF408 | ENSG00000175213 | 19.43 | 0.23 | 0.0030 | 0.0342 | 1.55 |
| ZNF415 | ENSG00000170954 | 15.47 | 0.19 | 0.0115 | 0.0438 | 0.87 |
| ZNF417 | ENSG00000173480 | 109.91 | 0.19 | 0.0092 | 0.0408 | 1.34 |
| ZNF420 | ENSG00000197050 | 12.84 | 0.18 | 0.0159 | 0.0498 | 1.90 |
| ZNF432 | ENSG00000256087 | 31.64 | 0.19 | 0.0129 | 0.0457 | 1.44 |
| ZNF434 | ENSG00000140987 | 31.81 | 0.18 | 0.0160 | 0.0499 | 1.69 |
| ZNF443 | ENSG00000180855 | 20.63 | 0.22 | 0.0035 | 0.0342 | 2.18 |
| ZNF445 | ENSG00000185219 | 175.96 | 0.18 | 0.0145 | 0.0479 | 1.41 |
| ZNF449 | ENSG00000173275 | 25.99 | 0.20 | 0.0113 | 0.0435 | 1.67 |
| ZNF45 | ENSG00000124459 | 37.25 | 0.20 | 0.0067 | 0.0373 | 1.22 |
| ZNF462 | ENSG00000148143 | 221.24 | 0.22 | 0.0036 | 0.0342 | 1.45 |
| ZNF468 | ENSG00000204604 | 35.86 | 0.22 | 0.0029 | 0.0342 | 1.89 |
| ZNF48 | ENSG00000180035 | 21.83 | 0.19 | 0.0115 | 0.0438 | 2.13 |
| ZNF488 | ENSG00000165388 | 5.89 | 0.20 | 0.0151 | 0.0487 | 1.93 |
| ZNF490 | ENSG00000188033 | 36.36 | 0.20 | 0.0071 | 0.0376 | 1.62 |
| ZNF498 | ENSG00000197037 | 76.13 | 0.18 | 0.0119 | 0.0442 | 1.49 |
| ZNF503 | ENSG00000165655 | 21.63 | 0.20 | 0.0081 | 0.0391 | 3.13 |
| ZNF512 | ENSG00000243943 | 91.17 | 0.22 | 0.0032 | 0.0342 | 1.43 |
| ZNF513 | ENSG00000163795 | 61.51 | 0.19 | 0.0127 | 0.0454 | 0.96 |
| ZNF514 | ENSG00000144026 | 111.97 | 0.23 | 0.0023 | 0.0342 | 1.87 |
| ZNF517 | ENSG00000197363 | 28.33 | 0.19 | 0.0129 | 0.0457 | 1.91 |
| ZNF524 | ENSG00000171443 | 19.46 | 0.24 | 0.0015 | 0.0342 | 1.49 |
| ZNF526 | ENSG00000167625 | 45.14 | 0.23 | 0.0026 | 0.0342 | 1.80 |
| ZNF528 | ENSG00000167555 | 59.48 | 0.21 | 0.0047 | 0.0353 | 0.94 |
| ZNF543 | ENSG00000178229 | 17.81 | 0.20 | 0.0067 | 0.0373 | 1.69 |
| ZNF544 | ENSG00000198131 | 78.27 | 0.22 | 0.0018 | 0.0342 | 1.69 |
| ZNF547 | ENSG00000152433 | 6.41 | 0.21 | 0.0057 | 0.0363 | 1.54 |
| ZNF552 | ENSG00000178935 | 101.96 | 0.23 | 0.0023 | 0.0342 | 1.06 |
| ZNF557 | ENSG00000130544 | 53.40 | 0.18 | 0.0148 | 0.0484 | 1.43 |
| ZNF558 | ENSG00000167785 | 41.15 | 0.19 | 0.0092 | 0.0408 | 1.64 |
| ZNF559 | ENSG00000188321 | 31.59 | 0.18 | 0.0161 | 0.0500 | 1.38 |
| ZNF561 | ENSG00000171469 | 67.26 | 0.19 | 0.0093 | 0.0409 | 1.52 |
| ZNF564 | ENSG00000249709 | 66.05 | 0.19 | 0.0089 | 0.0403 | 1.22 |
| ZNF574 | ENSG00000105732 | 31.10 | 0.18 | 0.0156 | 0.0494 | 1.83 |
| ZNF580 | ENSG00000213015 | 41.54 | 0.19 | 0.0148 | 0.0484 | 1.03 |
| ZNF585A | ENSG00000196967 | 22.72 | 0.18 | 0.0144 | 0.0477 | 1.50 |
| ZNF585B | ENSG00000245680 | 31.42 | 0.19 | 0.0114 | 0.0437 | 1.32 |
| ZNF587 | ENSG00000198466 | 542.67 | 0.23 | 0.0014 | 0.0342 | 1.41 |
| ZNF592 | ENSG00000166716 | 208.65 | 0.18 | 0.0132 | 0.0460 | 1.22 |
| ZNF600 | ENSG00000189190 | 41.19 | 0.23 | 0.0012 | 0.0342 | 1.25 |
| ZNF605 | ENSG00000196458 | 64.84 | 0.23 | 0.0026 | 0.0342 | 1.55 |
| ZNF606 | ENSG00000166704 | 25.54 | 0.20 | 0.0090 | 0.0405 | 1.26 |
| ZNF608 | ENSG00000168916 | 113.73 | 0.18 | 0.0129 | 0.0457 | 1.06 |
| ZNF609 | ENSG00000180357 | 307.43 | 0.18 | 0.0156 | 0.0494 | 1.22 |
| ZNF613 | ENSG00000176024 | 18.87 | 0.18 | 0.0136 | 0.0466 | 1.26 |
| ZNF615 | ENSG00000197619 | 23.42 | 0.18 | 0.0105 | 0.0426 | 1.33 |
| ZNF616 | ENSG00000204611 | 18.94 | 0.27 | 0.0005 | 0.0342 | 1.67 |
| ZNF618 | ENSG00000157657 | 61.93 | 0.21 | 0.0070 | 0.0376 | 1.84 |
| ZNF623 | ENSG00000183309 | 89.44 | 0.20 | 0.0066 | 0.0373 | 2.10 |
| ZNF624 | ENSG00000197566 | 14.32 | 0.20 | 0.0076 | 0.0382 | 1.16 |
| ZNF627 | ENSG00000198551 | 32.04 | 0.17 | 0.0156 | 0.0494 | 1.91 |
| ZNF628 | ENSG00000197483 | 17.40 | 0.19 | 0.0130 | 0.0458 | 1.56 |
| ZNF629 | ENSG00000102870 | 101.13 | 0.22 | 0.0021 | 0.0342 | 1.81 |
| ZNF630 | ENSG00000221994 | 19.23 | 0.26 | 0.0018 | 0.0342 | 1.61 |
| ZNF638 | ENSG00000075292 | 435.23 | 0.18 | 0.0098 | 0.0416 | 1.39 |
| ZNF646 | ENSG00000167395 | 127.14 | 0.21 | 0.0070 | 0.0376 | 1.62 |
| ZNF652 | ENSG00000198740 | 247.82 | 0.20 | 0.0066 | 0.0373 | 1.30 |
| ZNF654 | ENSG00000175105 | 52.22 | 0.19 | 0.0112 | 0.0434 | 1.40 |
| ZNF664 | ENSG00000179195 | 403.79 | 0.24 | 0.0008 | 0.0342 | 1.84 |
| ZNF673 | ENSG00000147121 | 24.50 | 0.20 | 0.0074 | 0.0380 | 1.81 |
| ZNF683 | ENSG00000176083 | 3.14 | 0.26 | 0.0043 | 0.0351 | 1.39 |
| ZNF687 | ENSG00000143373 | 95.13 | 0.21 | 0.0033 | 0.0342 | 1.53 |
| ZNF688 | ENSG00000229809 | 26.08 | 0.20 | 0.0061 | 0.0368 | 1.15 |
| ZNF69 | ENSG00000198429 | 68.09 | 0.22 | 0.0043 | 0.0351 | 1.27 |
| ZNF692 | ENSG00000171163 | 82.24 | 0.23 | 0.0028 | 0.0342 | 1.45 |
| ZNF7 | ENSG00000147789 | 102.36 | 0.19 | 0.0098 | 0.0416 | 1.59 |
| ZNF70 | ENSG00000187792 | 42.07 | 0.19 | 0.0126 | 0.0452 | 1.13 |
| ZNF701 | ENSG00000167562 | 56.36 | 0.18 | 0.0144 | 0.0477 | 1.22 |
| ZNF704 | ENSG00000164684 | 331.28 | 0.19 | 0.0097 | 0.0415 | 1.18 |
| ZNF711 | ENSG00000147180 | 52.54 | 0.19 | 0.0135 | 0.0464 | 1.18 |
| ZNF720 | ENSG00000197302 | 71.41 | 0.18 | 0.0150 | 0.0486 | 1.53 |
| ZNF74 | ENSG00000185252 | 27.34 | 0.23 | 0.0031 | 0.0342 | 2.67 |
| ZNF747 | ENSG00000169955 | 51.28 | 0.21 | 0.0038 | 0.0342 | 1.19 |
| ZNF750 | ENSG00000141579 | 16.61 | 0.19 | 0.0095 | 0.0413 | 2.00 |
| ZNF75D | ENSG00000186376 | 81.73 | 0.18 | 0.0152 | 0.0489 | 1.45 |
| ZNF761 | ENSG00000160336 | 51.55 | 0.20 | 0.0084 | 0.0397 | 1.84 |
| ZNF765 | ENSG00000196417 | 44.66 | 0.23 | 0.0014 | 0.0342 | 1.72 |
| ZNF766 | ENSG00000196214 | 37.30 | 0.18 | 0.0145 | 0.0479 | 2.01 |
| ZNF768 | ENSG00000169957 | 79.84 | 0.23 | 0.0027 | 0.0342 | 1.52 |
| ZNF771 | ENSG00000179965 | 4.51 | 0.20 | 0.0106 | 0.0427 | 1.94 |
| ZNF774 | ENSG00000196391 | 45.51 | 0.27 | 0.0006 | 0.0342 | 0.93 |
| ZNF775 | ENSG00000196456 | 52.77 | 0.22 | 0.0047 | 0.0353 | 1.65 |
| ZNF776 | ENSG00000152443 | 64.53 | 0.20 | 0.0067 | 0.0373 | 1.32 |
| ZNF780A | ENSG00000197782 | 69.41 | 0.20 | 0.0076 | 0.0382 | 1.38 |
| ZNF785 | ENSG00000197162 | 71.46 | 0.22 | 0.0029 | 0.0342 | 1.58 |
| ZNF787 | ENSG00000142409 | 45.09 | 0.19 | 0.0145 | 0.0479 | 1.29 |
| ZNF789 | ENSG00000198556 | 143.11 | 0.21 | 0.0048 | 0.0354 | 1.46 |
| ZNF8 | ENSG00000083842 | 35.59 | 0.19 | 0.0091 | 0.0407 | 1.60 |
| ZNF808 | ENSG00000198482 | 142.37 | 0.18 | 0.0122 | 0.0446 | 1.00 |
| ZNF81 | ENSG00000197779 | 84.60 | 0.20 | 0.0073 | 0.0379 | 1.23 |
| ZNF813 | ENSG00000198346 | 25.58 | 0.18 | 0.0104 | 0.0424 | 1.52 |
| ZNF827 | ENSG00000151612 | 159.33 | 0.23 | 0.0015 | 0.0342 | 1.25 |
| ZNF839 | ENSG00000022976 | 47.94 | 0.18 | 0.0159 | 0.0498 | 1.11 |
| ZNF844 | ENSG00000223547 | 56.40 | 0.24 | 0.0013 | 0.0342 | 1.18 |
| ZNF845 | ENSG00000213799 | 23.47 | 0.28 | 0.0002 | 0.0342 | 1.76 |
| ZNF846 | ENSG00000196605 | 27.66 | 0.21 | 0.0037 | 0.0342 | 1.12 |
| ZNF862 | ENSG00000106479 | 110.41 | 0.20 | 0.0071 | 0.0376 | 1.15 |
| ZNF891 | ENSG00000214029 | 33.29 | 0.20 | 0.0076 | 0.0382 | 1.59 |
| ZNF91 | ENSG00000167232 | 307.17 | 0.22 | 0.0032 | 0.0342 | 1.05 |
| ZNFX1 | ENSG00000124201 | 253.51 | 0.21 | 0.0044 | 0.0351 | 1.93 |
| ZNHIT1 | ENSG00000106400 | 88.18 | 0.20 | 0.0053 | 0.0361 | 1.61 |
| ZNHIT2 | ENSG00000174276 | 6.52 | 0.24 | 0.0036 | 0.0342 | 1.96 |
| ZNRF1 | ENSG00000186187 | 50.29 | 0.19 | 0.0090 | 0.0405 | 1.29 |
| ZNRF2 | ENSG00000180233 | 114.77 | 0.20 | 0.0064 | 0.0370 | 1.35 |
| ZSCAN16 | ENSG00000196812 | 13.31 | 0.19 | 0.0106 | 0.0427 | 1.64 |
| ZSCAN2 | ENSG00000176371 | 25.51 | 0.21 | 0.0049 | 0.0355 | 1.60 |
| ZSCAN21 | ENSG00000166529 | 20.46 | 0.24 | 0.0014 | 0.0342 | 1.83 |
| ZSCAN29 | ENSG00000140265 | 96.69 | 0.22 | 0.0043 | 0.0351 | 1.31 |
| ZSWIM1 | ENSG00000168612 | 34.45 | 0.20 | 0.0074 | 0.0380 | 2.52 |
| ZSWIM5 | ENSG00000162415 | 61.85 | 0.21 | 0.0043 | 0.0351 | 0.91 |
| ZXDC | ENSG00000070476 | 170.90 | 0.18 | 0.0130 | 0.0458 | 1.30 |
| ZYG11B | ENSG00000162378 | 135.47 | 0.19 | 0.0100 | 0.0419 | 1.31 |
| ZZEF1 | ENSG00000074755 | 886.46 | 0.19 | 0.0082 | 0.0393 | 0.62 |
| ZZZ3 | ENSG00000036549 | 160.14 | 0.19 | 0.0126 | 0.0452 | 1.73 |

^1^Refers to the association of mRNA expression association with miRNA expression in normal colorectal mucosa.
